# Evaluation of ultra-deep targeted sequencing for personalized breast cancer care

**DOI:** 10.1186/bcr3584

**Published:** 2013-12-10

**Authors:** Olivier Harismendy, Richard B Schwab, Hakan Alakus, Shawn E Yost, Hiroko Matsui, Farnaz Hasteh, Anne M Wallace, Hannah L Park, Lisa Madlensky, Barbara Parker, Philip M Carpenter, Kristen Jepsen, Hoda Anton-Culver, Kelly A Frazer

**Affiliations:** 1 Division of Genome Information Sciences, Department of Pediatrics and Rady Children’s Hospital, University of California San Diego, 9500 Gilman Drive, La Jolla CA 92093, USA; 2 Moores UCSD Cancer Center, School of Medicine, University of California San Diego, 3855 Health Science Drive, La Jolla CA 92093, USA; 3 Clinical and Translational Science Institute, University of California San Diego, 9500 Gilman Drive, La Jolla CA 92093, USA; 4 Department of Medicine, School of Medicine, University of California San Diego, 9500 Gilman Drive, La Jolla CA 92093, USA; 5 Department of Pathology, School of Medicine, University of California San Diego, 9500 Gilman Drive, La Jolla CA 92093, USA; 6 Department of Surgery, School of Medicine, University of California San Diego, 9500 Gilman Drive, La Jolla CA 92093, USA; 7 Bioinformatics Graduate Program, University of California San Diego, 9500 Gilman Drive, La Jolla CA 92093, USA; 8 Department of Family and Preventive Medicine, School of Medicine, University of California San Diego, La Jolla CA, USA; 9 Institute for Genomic Medicine, University of California San Diego, 9500 Gilman Drive, La Jolla CA 92093, USA; 10 Department of Epidemiology, School of Medicine, University of California Irvine, 252 Irvine Hall, Irvine CA 92697, USA; 11 Department of Pathology and Laboratory Medicine, School of Medicine, University of California Irvine, 252 Irvine Hall, Irvine CA 92697, USA; 12 Department of General, Visceral and Cancer Surgery, University of Cologne, Frangenheimstraße 4, 50931, Köln Germany

## Abstract

**Introduction:**

The increasing number of targeted therapies, together with a deeper understanding of cancer genetics and drug response, have prompted major healthcare centers to implement personalized treatment approaches relying on high-throughput tumor DNA sequencing. However, the optimal way to implement this transformative methodology is not yet clear. Current assays may miss important clinical information such as the mutation allelic fraction, the presence of sub-clones or chromosomal rearrangements, or the distinction between inherited variants and somatic mutations. Here, we present the evaluation of ultra-deep targeted sequencing (UDT-Seq) to generate and interpret the molecular profile of 38 breast cancer patients from two academic medical centers.

**Methods:**

We sequenced 47 genes in matched germline and tumor DNA samples from 38 breast cancer patients. The selected genes, or the pathways they belong to, can be targeted by drugs or are important in familial cancer risk or drug metabolism.

**Results:**

Relying on the added value of sequencing matched tumor and germline DNA and using a dedicated analysis, UDT-Seq has a high sensitivity to identify mutations in tumors with low malignant cell content. Applying UDT-Seq to matched tumor and germline specimens from the 38 patients resulted in a proposal for at least one targeted therapy for 22 patients, the identification of tumor sub-clones in 3 patients, the suggestion of potential adverse drug effects in 3 patients and a recommendation for genetic counseling for 2 patients.

**Conclusion:**

Overall our study highlights the additional benefits of a sequencing strategy, which includes germline DNA and is optimized for heterogeneous tumor tissues.

## Introduction

The use of highly effective targeted therapies in cancer frequently depends on the specific mutational profile of the tumor. As an increasing number of targeted therapies become available, determining the comprehensive genetic profile of a tumor is critical in understanding the response to targeted drugs for cancer treatment. Indeed, this genetic profile can help predict sensitivity or resistance to particular therapies and can therefore offer new, tailored treatment options to patients with late-stage or recurrent disease. In breast cancer, for example, trastuzumab has been used for Her2 amplified or overexpressing breast cancer. Notably, this strategy may suggest the use of a drug indicated for another anatomic cancer type, or the use of an investigational drug. Measuring the true clinical benefit of this tailored strategy is difficult, however, because targeted therapy frequently leads to drug resistance, the mechanisms of which are often not well understood. Nevertheless, this area of research is developing rapidly and some preliminary studies matching therapy to the tumor mutational profile across many clinical trials show an improved response rate [1].

Traditionally, several types of molecular assays are available to identify somatic DNA mutations in tumors. Such assays analyze single positions, single exons, or whole genes using mass spectrometry [2], allele-specific polymerase chain reaction (PCR) [3] or Sanger sequencing. These assays are, however, limited in scope – looking only at specific genes or mutations – and limited in sensitivity – usually dependent on the fraction of tumor cells contained in the tissue specimen. More recently, high-throughput sequencing of candidate genes has extended the breadth and sensitivity of this approach, overcoming some of these drawbacks [4-7]. Some major clinical centers are now starting to use more comprehensive molecular profiling in clinical care. However, these assays differ with regards to breadth (number of genes), depth (number of independent DNA molecules sampled) and design – selection of the genes or inclusion of a matched germline control. As a consequence, the clinical utility may vary. The Cancer Genome Atlas (TCGA) [8], a consortium focused on research and discovery, sequenced the entire exome of tumors but at limited coverage depth, rejecting specimens with less than 60% cellularity and preventing the reliable identification of subclonal mutations. More targeted commercial assays such as Foundation One (Foundation Medicine, Cambridge, MA) may generate increased coverage depth of a smaller set of genes but do not always report the mutant allelic fraction [9]. Such diagnostic services also omit the comparison with a matched germline control, which is essential to increase the analytical sensitivity and distinguish between inherited variants and somatic mutations.

Ultra-deep targeted sequencing (UDT-Seq) [5,10] of matched tumor–germline specimens has not yet been evaluated in a clinical setting. The sequencing of matched tumor–germline samples is crucial to distinguish somatic mutations from sequencing artifacts; it is also critical to establish with certainty that a variant identified in the tumor is somatic rather than inherited since filtering against polymorphism databases can eliminate real mutations [11]. In the absence of a matched germline DNA sequence, the misinterpretation of an inherited variant for a somatic mutation could potentially prevent a patient from getting appropriate genetic counseling. Additionally, inherited variation in metabolism genes such as *DPYD* or *CYP2D6* has been associated with 5-fluorouracil toxicity and possibly tamoxifen efficacy [12], respectively, and, although the variants are rare, a more systematic clinical screening would provide important benefits. The simultaneous sequencing of the germline DNA along with the tumor DNA therefore offers technical advantages to identify somatic mutations at low allelic fraction and increases the opportunity to identify actionable inherited variants.

Here, we evaluate a targeted sequencing assay for its use in a cancer clinical setting. Specifically, we performed UDT-Seq of 47 genes that are clinically actionable or important for patient care. We show that potentially important information is gained by sequencing at high depth, including identification of subclonal mutations. Additional information is also gained from the sequencing of matched germline DNA and from the inference of tumor DNA copy number alterations. We therefore demonstrate that in comparison with other high-throughput sequencing methods, UDT-Seq of matched tumor–germline DNA used in a clinical setting generates more potentially actionable findings for a greater number of patients.

## Methods

### Clinical specimens

All University of California, San Diego and University of California, Irvine patients were consented in accordance with the protocols approved by their respective Institutional Review Board of the university (Table S1 in Additional file 1 and Additional file 2). Snap-frozen tissue samples were subjected to mechanical pulverization, followed by disruption of the tissue in lysis buffer and DNA/RNA extraction using AllPrep DNA extraction kits (Qiagen GmbH, Hilden, Germany) according to the manufacturer’s recommendation. Germline DNA was extracted from blood clots using Qiagen Clotspin Baskets and DNA QIAmp DNA Blood maxi kits (Qiagen Inc., Valencia, CA, USA) and from saliva samples according to the respective manufacturer’s protocol.

### Data generation

The data were generated according to our published UDT-Seq method [5,10]. Briefly, the genomic DNA samples were fragmented to an average size of 3 kb. To prepare the input DNA template mixture for targeted amplification, 1.5 μg of the purified genomic DNA fragmentation reaction was added to 9.4 μl of 10× High-Fidelity Buffer (11304–029; Invitrogen (Carlsbad, CA, USA)), 2.5 μl of 50 mM MgSO_4_, 2.5 μl of 10 mM dNTP, 7.2 μl of 4 M Betaine, 7.2 μl RDT Droplet Stabilizer, 3.6 μl dimethyl sulfoxide and 1.4 μl of 5 units/μl Platinum High-Fidelity Taq (Invitrogen), and the samples were brought to a final volume of 50 μl with nuclease-free water. The primer droplets (Table S2 in Additional file 1 and Additional file 2) were merged with the sample droplets on the RDT1000 (RainDance Technologies (Billerica, MA, USA)).

The PCR reactions were carried out as follows: initial denaturation at 94°C for 2 minutes; 55 cycles at 94°C for 30 seconds, 54°C for 30 seconds and 68°C for 60 seconds; and final extension at 68°C for 10 minutes, followed by a 4°C hold. After breaking the emulsion and purification of the amplicons, the samples were subjected to the secondary PCR using 0.5 μM final concentration of a universal forward primer and an index-specific reverse primer (Table S3 in Additional file 1). Samples were amplified as follows: initial denaturation at 94°C for 2 minutes; 10 cycles at 94°C for 30 seconds, 56°C for 30 seconds and 68°C for 1 minute; and final extension at 68°C for 10 minutes, followed by a 4°C hold. The purified amplified library was then analyzed on an Agilent Bioanalyzer to quantify final amplicon yield and pooled in equimolar amounts. The pool was loaded at between 8 and 11 pM (depending on the run) and sequenced on the Illumina (San Diego, CA, USA) MiSeq sequencing instrument for 2 × 150 cycles using custom sequencing primers (Table S3 in Additional file 1). The resulting reads were deconvoluted based on their index sequence. The raw reads are publically available through the Short Reads Archive at the NCBI: SRA067610 and SRA067611. The libraries were sequenced to an average of 3.1 million 151 bp long paired-end reads per sample (Table S4 in Additional file 1).

### Data analysis

#### Mutascope

The analysis was performed using Mutascope capable of detecting mutations at 1% allelic fraction with high sensitivity [10]. We first identified potential false positive variants (module makeBlackList). We then aligned the reads to the human genome (modules runBWA, refinement, groupRealign, and xpileup). Mutascope calculates the error rate for each position/substitution/strand group (module calcErrorRates) at positions that are not database of single nucleotide polymorphisms (dbSNP) positions and uses this to calculate the binomial probability of mutations in the tumor (module callSomatic) distinguishing somatic from germline using an additional Fisher exact test. Finally, likely false positive mutations were filtered out using coverage bias, read-group bias, ambiguity of alternate allele, mapping quality, alternate allele quality, proximity to an indel, or to a homopolymer.

#### Copy number alterations

The average number of reads per gene was calculated for each sample sequenced. We then computed the mean and standard deviation of the normalized coverage in the germline DNA for each patient at each gene. The significance of amplification or deletion of a specific gene in the tumor DNA was estimated by comparing the tumor normalized coverage to the distribution of normal normalized coverage at this gene for all patients, using the R function *pnorm*. Following the Bonferroni correction for multiple testing, we reported amplifications (log*R* >1) and deletion (log*R* < -1) events with *P* <5.6 × 10^–6^ (Table S5 in Additional file 1).

#### Variant annotation

Variants were queried against dbSNP135 to determine novel or known variants. We next used snpEff [13] version 2.0.5 in combination with GATK VariantAnnotator (Broad Institute, Cambridge MA, USA), both with default parameters, to identify the different functional impacts on coding genes. We enriched this annotation by cross-referencing the list of variants to the dbNSFP database [14], which provides conservation (PhyloP), functional prediction (SIFT, PolyPhen and MutationTaster), as well as Uniprot codon change information. Finally, we annotated the variants for presence in Catalogue of Somatic Mutations in Cancer v61 (Welcome Trust Sanger Institute, Hinxton, UK) based on coordinate and genotype. Notably, we used Catalogue of Somatic Mutations in Cancer codon numbering when discordant numbering was reported between databases.

## Results

**Figure 1:**
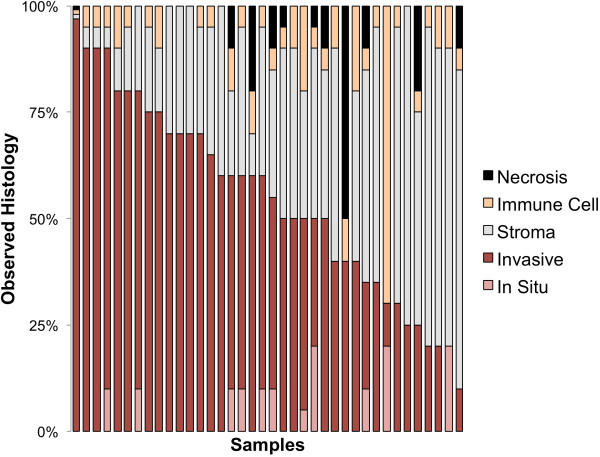
**Histology examination.** For each sample, the proportion of necrosis, immune cells, stromal cells, *in situ* tumor and invasive tumor is indicated.

We collected 38 tumors, including two lobular invasive carcinoma, 35 ductal invasive carcinoma (six of which showed lobular features) and one Ductal Carcinoma In Situ. Notably, four tumors had cellularity lower than 20% (Figure 1) and six tumors were Her2-positive as determined by standard testing (Table S1 in Additional file 1 and Additional file 2). We assembled a panel of 47 genes to analyze these specimens using UDT-Seq. The genes were selected for their clinical importance or their relevance to breast cancer genetics and treatment (Table 1). The coverage resulting from the sequencing of the 1,736 amplicons from 38 pairs of tumor DNA and germline DNA was deep (Table S4 in Additional file 1; with an average of 1,481 reads per amplicon), sensitive (with 92% of the bases covered at 500× or more) and highly uniform (with an average of 92.6% of the bases within twofold of the mean) – in agreement with the published specifications of microdroplet PCR [5,10,15], which provides high-quality data for clinical sequencing.

**Table 1:** Genes included in the breast cancer panel sequenced by UDT-Seq

**Gene**	**Rationale for inclusion**^**a**^	**Molecular eligibility for clinical trial**	**US FDA-approved pathway inhibitor**	**Pathway inhibitor in clinical testing**
*PIK3CA*	S,	Y		Y
*PTEN*	S, G	Y		Y
*BRAF*	S,		Y	Y
*KRAS*	S,			Y
*EGFR*	S,		Y	Y
*ALK*	S,	Y	Y	Y
*ERBB2*	S,	Y	Y	Y
*JAK2*	S,		Y	
*PDGFRB*	S,		Y	
*RET*	S, G		Y	
*JAK1*	S,		Y	
*RARA*	S,		Y	
*TP53*	S, G			
*CDH1*	S,			
*GATA3*	S,			
*CTNNA1*	S,			
*RB1*	S, G			
*CDKN2A*	S,			Y
*AKT1*	S,		Y	Y
*APC*	S, G			
*PIK3R1*	S,			
*BRCA1*	S, G			Y
*ERBB3*	S,			
*JAK3*	S,		Y	
*NOTCH1*	S,			Y
*MET*	S, G		Y	Y
*FGFR2*	S,	Y		Y
*ABL2*	S,			
*BRCA2*	S, G			Y
*CTNNB1*	S,			
*ERBB4*	S,			Y
*FGFR1*	S,	Y		Y
*FGFR1OP*	S,			
*PALB2*	S, G			
*TOP1*	S,		Y	
*DPD*	P (capecitabine/5-fluorouracil)			
*TPMT*	P (6-mercaptopurine thioguanine)			
*CYP2D6*	P (tamoxifen (+/-))			
*CYP2C9*	P (warfarin)			
*VKORC1*	P (warfarin)			
*CFTR*	R (cystic fibrosis)			
*MLH1*	G			
*MSH2*	G			
*MSH6*	G			
*PMS2*	G			
*CHK2*	G			
*ATM*	G			

FDA, Food and Drug Administration; UDT-Seq, ultra-deep targeted sequencing. ^a^S somatic mutations; G, germline cancer risk; P, pharmacogenetic risk; R, reproductive significance.

### Chromosomal alterations

The precise allelic fraction measured at each sequenced position by UDT-Seq can be reflective of the prevalence of a mutated clone in the tumor sample, but can also result from chromosomal losses or gains. Therefore it is important to first identify these chromosomal alterations to interpret the mutations’ allelic fraction but also to reveal potential actionable events such as the amplification of a targetable oncogene.

**Figure 2:**
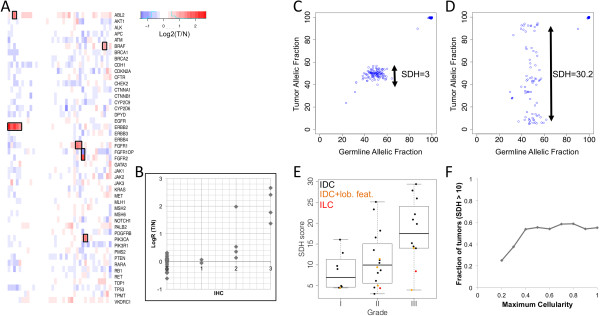
**Somatic rearrangements. (A)** Heatmap representing the average log*R* ratio of tumor/germline coverage depth observed on all amplicons of a given gene (rows) in the sequenced samples (columns). Red, gains; blue, losses. Black frames indicate significant changes (*P* <5.6 × 10^–6^). **(B)** The log*R* ratios of tumor/germline coverage depth of the *Her2* gene correlate with the results of immunohistochemistry. **(C)**, **(D)** Scatterplot representing the allelic fraction of the germline variants in the germline DNA (*x* axis) and tumor DNA (y-axis) for tumors showing a low **(C)** or high **(D)** level of chromosomal instability. The standard deviation of heterozygotes (SDH) score, calculated from the standard deviation of the allelic fraction of heterozygous single nucleotide polymorphisms in the tumor, is indicated. **(E)** Distribution of SDH scores in the sequenced cohort as a function of histological grade (*x* axis). Invasive lobular carcinoma (ILC; red) and invasive ductal carcinoma (IDC) showing lobular features (orange) are indicated. **(F)** Cumulative fraction of tumors with high SDH score (*y* axis), at increasing tumor cellularity (*x* axis). IHC, immunohistochemistry.

As shown previously, the distribution of the fractions of reads per amplicon generated by UDT-Seq is highly reproducible from sample to sample [5]. As a result, the difference in coverage depth of an amplicon between tumor and germline can be indicative of chromosome copy number gains or losses. Indeed, we noticed that five of the six samples determined by traditional methods (immunohistochemistry or fluorescent *in situ* hybridization) to have *Her2* amplification show a higher coverage depth at *ERBB2* amplicons, the gene coding for *Her2* (Figure 2A). The immunohistochemistry or fluorescent *in situ* hybridization score is correlated with the level of amplification determined by this approach (*r*^2^ = 0.70; Figure 2B). We also identified potential copy number gains of *ABL2*, *BRAF*, *FGFR2* and *PIK3CA* in one sample, *FGFR1* in two samples, as well as a loss of *FGFR1OP* in one sample (Figure 2A; Table S5 in Additional file 1). Despite the high coverage depth generated, the low tumor cell content and overall level of gene amplification in a sample can reduce the sensitivity of this approach, as illustrated by a false negative *Her2*-amplified sample, which had low *in situ* hybridization ratio (2.8) and 50% tumor cell content. Nevertheless, this inference of copy number alterations can identify *bona-fide* actionable events.

The high depth of sequencing of both tumor and germline also facilitates the identification of loss of heterozygosity events, by measuring the allelic fraction of heterozygous polymorphisms in the tumor (Figure 2C,D). This observed effect on allelic fraction is, however, a combination of tumor purity and ploidy that is difficult to separate using only ~150 germline variants per patient. We can summarize this instability using the standard deviation of the allelic fraction of the heterozygous single nucleotide polymorphisms observed in the tumor (standard deviation of heterozygotes (SDH) score; Figure 2E). The SDH score was correlated with the Nottingham grade (*P* <0.005, Student’s *t* test), indicating that high-grade tumors have more chromosomal rearrangements, especially for ductal carcinomas *in situ*. Similarly, for highly cellular tumors, a high SDH score is indicative of a high chromosomal instability. As expected, a higher fraction of elevated SDH score was observed in high cellularity samples (Figure 2F), indicating that chromosomal instability is more difficult to identify in heterogeneous samples using our approach. As described below, the identification of loss of heterozygosity events is important for the interpretation of the allelic fraction at somatic mutations.

### Tumors’ mutational landscape

We identified somatic variants, substitutions and insertion/deletions in the sequenced samples using Mutascope [10]. Four patients had no mutations, and 34 patients had between one and 12 nonsilent mutations (one to 16 total mutations). In total, we identified 76 somatic variants across the 34 cases, of which 62 were nonsilent, resulting in a coding change in 28 genes (Table S6 in Additional file 1).

**Figure 3:**
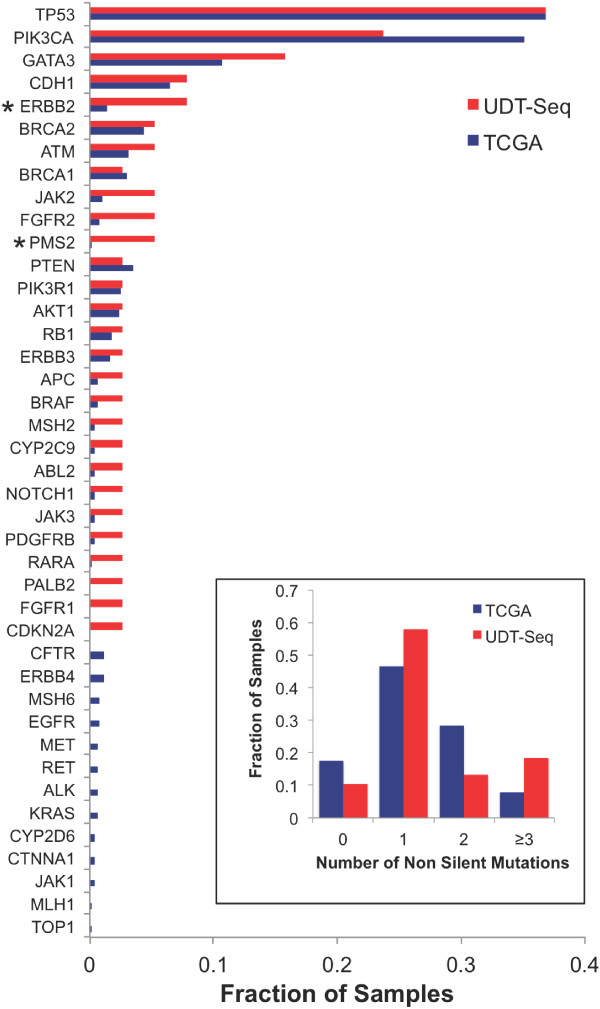
**Comparison with The Cancer Genome Atlas cohort.** Bar graph representing the fraction of samples with nonsilent somatic mutations in The Cancer Genome Atlas (TCGA) cohort (*n* = 507, blue) and the studied cohort (*n* = 38, red). *Statistically significant difference (Fisher test *P* <0.05). Inset: bar graph indicating the fraction of samples with none, one, two, or three or more nonsilent mutations over the entire TCGA cohort (blue) or studied cohort (red). UDT-Seq, ultra-deep targeted sequencing.

To highlight the specificities of the patient cohort and the sequencing assay, we compared our results with those obtained from a large TCGA cohort of 507 breast invasive carcinomas that were sequenced at all coding genes [8]. We observed that 17% of the TCGA samples had no detectable mutations in the 47 genes of our panel, as compared with the 10% of samples with no detectable mutations determined by our approach (Figure 3 inset). Similarly, there were three or more somatic mutations in 18% of the samples in our study compared with only 8% in the TCGA dataset. Thirty-nine of the 41 genes mutated either in our study or in the TCGA dataset were mutated in the same fraction of samples (*P* ≥0.05; Figure 3). Only *ERBB2* and *PMS2* showed a significant difference (*P* <0.05), although the large difference in sample size could weaken this comparison. Altogether, these observations suggest our approach has a greater sensitivity to detect mutations in potentially clinically actionable genes.

**Figure 4:**
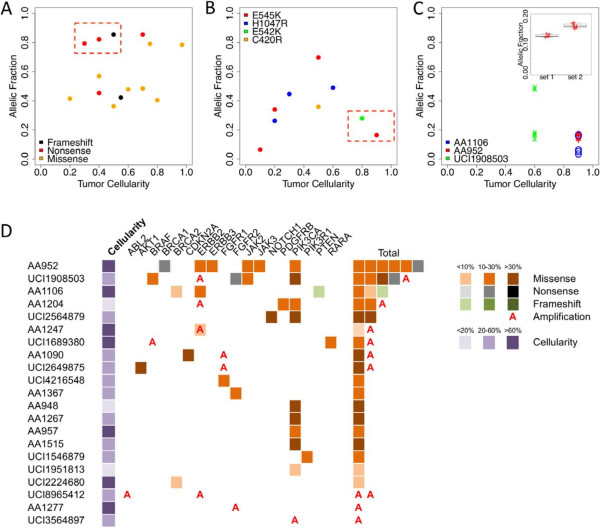
**Patterns and actionability of somatic mutations. (A), (B)** The allelic fraction of all *TP53***(A)** and *PIK3CA***(B)** nonsilent somatic mutants (*y* axis) is displayed as a function of the cellularity of the tumor (*x* axis). Red boxes indicate samples where the allelic fraction deviates from tumor cellularity. **(C)** The allelic fraction of the nonsilent somatic mutations in the three tumors showing evidence of two subclones is displayed as a function of the tumor cellularity (*x* axis). Inset: highlighting the distribution of allelic fraction of the mutations identified in the two clones of AA952. **(D)** Schematic representation of the type of somatic variation identified in the genes actionable for their somatic status. The tumor cellularity is displayed in a purple gradient color. The samples are ranked by decreasing number of actionable somatic mutations.

The most frequently mutated gene, *TP53*, was altered in 37% (14/38) of the patients. In six patients, the mutation was homozygous, leading to a frameshift (*n* = 1), a nonsense (*n* = 3) or a missense (*n* = 2), supporting the total loss of function of *TP53* in these cases. In one patient, three missense mutations (P142L, P158L and R158C) were present on the same DNA strand, indicating that one *TP53* allele remained wild-type. The remaining seven patients had heterozygous mutations, which were all predicted to be deleterious. Interestingly, we noticed *TP53* mutations with high allelic fraction in low cellularity tumors (Figure 4A, red box). Assuming that the adjacent tissue sections used for histology and sequencing have comparable cellularity, this suggests that *TP53* mutations may be present in the surrounding stroma, consistent with previous observations [16-19].

The second most frequently mutated gene, *PIK3CA*, was mutated in 24% (9/38) of the patients. All of the mutations occurred in mutational hotspots known to result in a phosphoinositide-3 kinase (PI3K) gain of function: E545K (*n* = 4), H1047R (*n* = 3), E542K (*n* = 1) and C420R (*n* = 1) [20]. In contrast to *TP53*, the allelic fraction of *PIK3CA* mutants was proportional to the tumor cellularity (Figure 4B), with the exception of two tumors (Figure 4B, red box) of high cellularity (>80%) and lower *PIK3CA* mutant allelic fraction (<30%), indicating that the mutations may have been present in only a subset of the tumor cells.

*GATA3* was found mutated in 16% (6/38) of the patients. Interestingly, five out of the six mutations led to a frameshift, consistent with the findings of the TCGA (88%, 38/43) and much higher than the initial *GATA3* mutational analysis performed by Sanger sequencing in breast cancer (30%, 2/6) [21]. The frameshift mutations in this transcription factor occurred in the vicinity of the Zn Finger domain (residues 263 to 313), which also surrounds the nuclear localization signal [22]. The mutations may therefore result in a loss of function by preventing DNA binding or nuclear import. The unique mutational profile of *GATA3*, dominated by frameshift mutations, may prompt further investigations about their mechanism of onset and significance.

We also identified less frequently mutated genes with potential value in the clinic. One patient’s tumor was determined to harbor a *PIK3R1*-K567E mutation, which has been observed in endometrial cancer [23]. Although the significance of this particular substitution is not known, loss of function mutations of the regulatory subunit of the PI3K complex can contribute to the activation of PI3K pathway [24]. Similarly the *PTEN* frameshift mutation identified in another patient’s tumor may result in partial *PTEN* loss of function and subsequent PI3K activation. Three patients carried missense mutations in *ERBB2*, all predicted to affect its function. Two of these mutations were located in the kinase domain and are known to mediate resistance to lapatinib (L755S) [25] or to activate Her2 (D769H) [26]. Finally, we identified four mutations in *CDH1* in three tumors. Interestingly, two tumors were diagnosed as lobular cancer and one had lobular features, in agreement with the increased prevalence of E-cadherin loss (encoded by *CDH1*) in lobular breast cancer [27].

### Tumor subclonal populations

While 35/38 patients had between zero and three somatic mutations, three patients had more than three mutations. Because of the high sequencing coverage depth (>1,000-fold), we were able to identify subclonal cell populations in these tumors (Table S7 in Additional file 1; Figure 4C). We identified one patient with 12 nonsilent mutations, which corresponds to about 10 times the average mutation rate observed in breast cancer [8]. Although this hypermutated tumor had a cellularity of 90%, we observed a set of seven mutations at 17% and a set of five mutations at 13% allelic fraction, with both sets representing statistically different populations (*P* <10^–5^, Student’s *t* test; Figure 4C, inset). One possible explanation is the presence of two subclones: assuming the seven mutations at higher allelic fraction are present in a heterozygous sate in a major founder clone (28% of the cells, 14% of the DNA) from which a minor clone arose, adding five heterozygous mutations (26% of the cells, 13% of the DNA). Among the founder clone mutations, we noticed a *BRCA1* nonsense mutation, which may explain the high mutation rate observed in this sample.

The last two patients carried six mutations each. One patient with lobular carcinoma had two *CDH1* mutations and one *ERBB2* mutation at ~16% allelic fraction, as well as a distinct set of mutations in *PTEN*, *BRCA2* and *PMS2* at ~5% allelic fraction. The observed allelic fractions are in contrast with the high cellularity (90%) and absence of strong rearrangement (SDH = 8.5) in this lobular tumor. Assuming that the mutations are not mutually exclusive, this observation implies that the loss of a *PTEN* allele only appeared recently in the tumor and that the majority of the tumor cells had no detectable somatic events in the panel of genes investigated. Finally, the tumor of one patient, also with low SDH and high cellularity, harbored two hallmark mutations at ~50% allelic fraction (*PIK3CA* and *TP53*) probably driving the initial tumor, but carried four mutations at ~16% allelic fraction, suggesting the presence of a subclone consisting of 32% of cells. This study highlights how the differences in allelic fraction observed within tumors can reveal subclonal populations and genetic drivers, and could be used to monitor treatment and possibly prevent future resistance.

### Importance of the germline variants

Our approach identified 586 inherited germline variants, with a median of 140 per patient, 85% of them present in dbSNP (Table S8 in Additional file 1 and Additional file 2). We first investigated the presence of deleterious variants in *BRCA1/2*, which are the most actionable genes in the clinical setting. We identified three patients with a predicted deleterious mutation in one of these genes, of which only one seems truly deleterious [28-30] (Table S9 in Additional file 1). The BRCA1-Q1355_E1356fs frameshift mutation is a previously reported deleterious mutation [30] and is clinically actionable. Interestingly, the mutant allele was selected for in the tumor (allelic fraction 94%), indicating a selective advantage. This germline finding was later confirmed by a Clinical Laboratory Improvement Amendments-approved assay after the patient consulted with a clinical genetic counselor.

Inherited variants in *DPYD* have been associated with toxicity to 5-fluorouracil or capecitabine chemotherapy [12], which is commonly used in breast cancer treatment. We identified six patients carrying three variants in *DPYD* with predicted deleterious effects. Three patients were heterozygous for rs1801160 (Minor Allele Frequency = 0.04). This single nucleotide polymorphism defines the DPYD*6 haplotype, which has been associated with increased toxicity [31]. Two novel missense variants (K259E and V944A) identified in three patients have an unknown significance. Interestingly, a recent study indicates that variants in *DPYD* can actually increase its metabolic activity, therefore protecting against toxicity and decreasing drug efficiency [32]. Until more functional experiments are performed, it will be challenging to unambiguously determine the clinical relevance of most inherited *DPYD* variants. We also identified two patients carrying one inactive allele of the gene (CYP2D6*6). However, it is not clear whether this particular allele, in a heterozygous state, is associated with a reduced metabolism of tamoxifen; therefore, a change in drug dosage is not justified.

More generally, our approach identified many inherited variants of unknown significance, which should be cautiously interpreted. Importantly, in the absence of a matched germline sample, some of these variants might have been misidentified as tumor-specific events potentially confounding the rationale for targeted therapy, therefore highlighting the importance of sequencing matched germline DNA.

### Clinical implications

Out of the 47 genes sequenced, 24 are classified as actionable based on their somatic status (Table 1). These genes or the pathway they belong to could be targeted by a specific inhibitor, commercially available or under investigation (*PIK3CA*, *ERBB2*), or are predictive biomarkers for targeted therapies that are approved or in clinical trials (*BRCA1/2*, *PTEN*). There were 21 patients whose tumors carried nonsilent mutations or copy number alterations in 17 of these 24 genes (Figure 4D). Importantly, three of the patients had tumors with less than 20% cellularity and in four patients we identified mutations at an allelic fraction of 10% or lower. We can establish the added benefit of our strategy in such cases: if we had limited our analysis to the samples with cellularity higher than 60% (19 samples), which is the inclusion criteria used by the TCGA, we would have identified mutations in only six patients for an overall sensitivity of only 31% (6/19 cases). However, by using the UDT-Seq approach, we identified mutations in actionable genes in 21 of the 38 patients studied for an overall sensitivity of 55% (21/38 cases), combining the benefits of less stringent inclusion criteria and higher assay sensitivity.

Based on these molecular findings, we then summarized the most likely clinical course of action (Table 2). Looking at somatic mutations and amplification, we would have proposed the use of trastuzumab for seven patients based on *ERBB2* status. Notably, for one of them the *ERBB2* gene is not amplified but carried an activating mutation, which would have been missed through standard Her2 testing. We would have further recommended the enrollment of 12 patients in a *PIK3CA* inhibitor clinical study due to a mutation in the PIK3/AKT/mTOR pathway. Four other patients may have been considered as candidates for the clinical testing of an *FGFR* inhibitor. Finally, for seven patients, the molecular testing suggests that they could each have benefited from *PARP*, *CDK4/6*, *AKT*, *ABL2*, *BRAF*, *JAK* or *RARA* inhibitors. Importantly, we were able to identify 18 patients who might specifically benefit from the advantages of our approach (Table 2). Regarding germline mutations, one patient carrying a germline *BRCA1* mutation underwent genetic counseling and had her mutation confirmed in a Clinical Laboratory Improvement Amendments-certified setting. One patient carried a germline *CFTR* deleterious mutation. These types of incidental findings, not related to breast cancer treatment, should be returned to the patient according to recent guidelines of the American College of Medical Genetics [33]. Overall, combining both somatic and germline discoveries, 25 patients had genetic results potentially informative for their care, of which 19 would not have been identified through routine testing.

**Table 2:** Summary of the primary course of action likely to result from the molecular testing

**Patient**	**SNP or mutation (allelic fraction)**	**Proposed action**	**UDT-Seq advantages**^**a**^
AA1025	rs113993959 (Het)	CFTR genetic counseling	Germline
AA1090	CDKN2A-A85D (66%)	CDK4/6 inhibitor	
	FGFR1 amplification	FGFR1/2 inhibitor	CNA
AA1106	ERBB2-L755S (17%)	Trastuzumab	
	PTEN-frameshift (5%)	PIK3CA inhibitor	Depth
	BRCA2-I1418T (4%)	PARP inhibitor	Depth
AA1204^b^	PIK3CA-H1047R (26%)	PIK3CA inhibitor	Sensitivity
	Her2 amplification	Trastuzumab	CNA
AA1222^b^	Her2 amplification	Trastuzumab	CNA
AA1247^b^	Her2 amplification	Trastuzumab	CNA
	ERBB2-D769H (5%)		Depth
AA1267	PIK3CA-H1047R (45%)	PIK3CA inhibitor	
AA1277	rs80357508 (Het)	BRCA1 genetic counseling	Germline
	FGFR2 amplification	FGFR1/2 inhibitor	CNA
AA948	PIK3CA-E545K (34%)	PIK3CA inhibitor	Sensitivity
AA952	PIK3CA-E545K (16%)	PIK3CA inhibitor	
	BRCA1-W306* (18%)	PARP inhibitor	
	BRCA1-E550K (13%)		
	JAK2-S131L (16%)	JAK inhibitor	
	JAK3-I386M (13%)		
	rs1801160 (Het)	5-FU toxicity	Germline
AA957	PIK3CA-E542K (28%)	PIK3CA inhibitor	
AA1515	PIK3CA-E545K (70%)	PIK3CA inhibitor	
UCI1546879	PIK3R1-K204E (30%)	PIK3CA inhibitor	
UCI1689380	RARA-337 T (14%)	RARA inhibitor	
	BRAF amplification	Vemurafenib	
UCI1908503^b^	PIK3CA-H1047R (40%)	PIK3CA inhibitor	
	Her2 amplification	Trastuzumab	CNA
UCI1951813	PIK3CA-E545K (7%)	PIK3CA inhibitor	Sensitivity
UCI2076630^b^	Her2 amplification	Trastuzumab	CNA
UCI2224680	BRCA2-L1829F (2%)	PARP inhibitor	Depth
UCI2564879	PIK3R1-K204E (30%)	PIK3CA inhibitor	
UCI2649875	AKT1-L52R (63%)	AKT inhibitor	
	FGFR1 amplification	FGFR1/2 inhibitor	CNA
UCI4216548	FGFR1-D683H (13%)	FGFR1/2 inhibitor	
UCI8965412^b^	Her2 amplification	Trastuzumab	CNA
	ABL2 amplification	Imatinib	CNA
UCI1804937	rs1801160 (Het)	5-FU toxicity	Germline
UCI2008866	rs1801160 (Het)	5-FU toxicity	Germline
UCI3564897	PIK3CA amplification	PIK3CA inhibitor	CNA

5-FU, 5-fluorouracil; SNP, single nucleotide polymorphism. ^a^Depth, accurate calls at low allelic fraction (<10%); sensitivity, accurate calls in heterogeneous samples; CNA, inference of copy number alterations; germline, inclusion of a matched germline DNA. ^b^Her2-positive determined through standard of care.

## Discussion

An increasing number of diagnostic companies and healthcare centers are proposing to perform tumor genetic profiling to support precision cancer care. Assays providing both deep and genome-wide or broad coverage are not yet available or currently justified in a clinical setting. Therefore, one should look directly at patient benefit and clinical utility to select an appropriate strategy. We still have a limited understanding of the role of most proteins even in pathways deemed actionable. Therefore, until more clinical evidence is provided, broad or genome-wide sequencing is likely to unveil mutations for which a clear therapeutic rationale is not yet available or misunderstood. In contrast, the use of deep sequencing of a restricted panel of genes increases the sensitivity to detect well-known and actionable mutations, which can have a greater impact in the clinic. For these reasons, deep sequencing of a restricted gene panel is likely to benefit the greatest number of patients today. Using our UDT-Seq approach, we identified potentially actionable mutations in 14/19 patients whose tumor samples had less than 60% cellularity and discovered actionable mutations present at 10% allelic fraction or less in four patients, some of whom had tumors with high malignant cellularity. UDT-Seq offers a very quantitative measurement of the allelic fraction of the mutations providing information about the biology of the tumor. For example, we observed a field effect in tumors harboring *TP53* mutations and the presence of subclonal *PIK3CA* mutations or of multiple mutated clones in three tumors, probably resulting from their evolution. Clinical utility of these new data will require specific trials to show that targeting resistant subclones or field effects is likely to improve outcomes in both the curative and palliative setting.

Traditionally, tumor-specific markers are investigated in the tumor specimen only. While this may be sufficient for protein markers, a DNA mutation is identified as a mismatch to the reference human genome and could correspond either to an inherited variant or somatically acquired mutation in the tumor. Only the sequencing of matched germline DNA can confirm that the variant is somatic, providing a better rationale for the use of targeted therapy, or inherited, providing important information for the care of the patient and their relatives. Finally, the use of matched germline DNA sequencing facilitates the detection of mutations at low allelic fraction [10,34], which, as discussed above, is likely to be extremely important for optimal implementation in clinical care. It is typically feasible to obtain a blood or buccal sample along with the tumor or biopsy sample being investigated, without excessive burden.

Importantly, the adoption of such transformative diagnostic assays in the clinic needs to include physician education and training and be associated with the establishment of molecular tumor boards in academic centers. These molecular tumor boards are not focused on a particular cancer by site of origin, but rather on the molecular markers identified. The presence of basic scientists with expertise in the altered pathways also improves the clinical interpretation. Indeed, the role and clinical significance of mutations located in less commonly mutated exons, genes or in the noncoding portions of the genome [35] remain to be established. Interpreting these variants of unknown significance, whether inherited or somatic, is the most controversial and difficult aspect of clinical sequencing. Despite attempts to consolidate variants, mutations, and clinical information in public databases, molecular tumor board members must currently perform extensive literature searches to predict the impact of a mutation. In our study, missense mutations in *ERBB2* were reported as activating by only a few published studies, suggesting their relevance for trastuzumab or lapatinib treatment. A similar challenge exists for the interpretation of polymorphisms in drug metabolizing genes, which will benefit from the efforts of the pharmacogenomics research network [36]. Finally, such precision medicine strategy is sensible only if it benefits the patients. For inherited variants, access to clinical genetic counseling is critical to interpret the results in the context of a complete family history. Similarly, targeting genes with somatic mutations using an investigational drug, requires access to a clinical trial or reimbursement for off-label use of targeted drugs with clinical outcome captured in a clinical registry study.

## Conclusion

Our study evaluates the potential benefits of the UDT-Seq of 47 selected genes for breast cancer care. We show that our assay identifies actionable findings, both inherited variants and somatic mutations, in 25 out of 38 samples. In particular, the specificities of our assay – inclusion of germline DNA, identification of copy number variants, high coverage depth and sensitivity to identify somatic mutations at low allelic fraction – would have been directly beneficial to 18 patients. As high-throughput sequencing starts to be used in clinical care, its establishment as a routine diagnostic assay will require progress on many fronts: demonstration of technical validity and clinical utility, education of physicians and trainees, and cooperation with pharmaceutical and insurance companies to increase drug accessibility.

## Abbreviations

CFTR: Cysitc Fibrosis Transmembrane Conductance Regulator; dbSNP: Database of single nucleotide polymorphisms; JAK: Janus kinase; PARP: Poly-ADP ribose polymerase; PCR: Polymerase chain reaction; PI3K: Phosphoinositide-3 kinase; SDH: Standard deviation of heterozygotes; TCGA: The Cancer Genome Atlas; UDT-Seq: Ultra-deep targeted sequencing.

## Competing interest

The authors declare that they have no competing interests.

## Authors’ contributions

OH, SEY, HM and LM analyzed the data. HA and KJ generated the data. RBS, FH, AMW, HLP and PMC collected and analyzed the samples and clinical information. OH, KAF, HA-C, BP and RBS designed the study. OH, LM, RBS and KAF wrote the manuscript. All authors read and approved the final manuscript.

## Supplementary Material

Additional file 1: Table S1 Presenting the source and histopathologic description of the 38 specimens studied, **Table S2.** Presenting the list of primers used in the study, **Table S3.** Presenting the universal PCR primers, **Table S4.** Presenting the significant copy number gains and losses, **Table S5.** Presenting the read alignment and coverage statistics, **Table S6.** Presenting the list of nonsilent somatic mutations, **Table S7.** Presenting the list of mutations that segregate into two significantly different groups of allelic fractions in the tumors of three patients, **Table S8.** Presenting the list of nonsilent germline variants, and **Table S9.** Presenting the list of predicted deleterious inherited variants in BRCA1 or BRCA2. Click here for file

Additional file 2 Supplemental text. Click here for file
