# Supplementary material for: Evaluation of ultra-deep targeted sequencing for personalized breast cancer care
Source: Breast Cancer Res. 2013 Dec 10;15(6):R115. doi: 10.1186/bcr3584 (PMC3978701; doi:10.1186/bcr3584)
Supplement: Additional file 1: Table S1 — Presenting the source and histopathologic description of the 38 specimens studied, Table S2. Presenting the list of primers used in the study, Table S3. Presenting the universal PCR primers, Table S4. Presenting the significant copy number gains and losses, Table S5. Presenting the read alignment and coverage statistics, Table S6. Presenting the list of nonsilent somatic mutations, Table S7. Presenting the list of mutations that segregate into two significantly different groups of allelic fractions in the tumors of three patients, Table S8. Presenting the list of nonsilent germline variants, and Table S9. Presenting the list of predicted deleterious inherited variants in BRCA1 or BRCA2. [file bcr3584-S1.pdf]

**Table S1: Source and histo-pathologic description of the 38 specimens studied**

| Sample ID | Sample source | ER | PR | Her2 | | Histology of primary site* | Notthingham grade | Histology | | | | |
|---|---|---|---|---|---|---|---|---|---|---|---|---|
| | | | | ISH ratio | IHC | | | In Situ | Invasive | Stroma | Immune Cell | Necrosis |
| AA0948 | Lumpectomy | >80 | <5 | 1.05 | 1+ | IDC | I | 0% | 20% | 70% | 10% | 0% |
| AA0930 | Lumpectomy | 90 | 50 | NA | 0 | IDC + LF | I | 0% | 25% | 75% | 0% | 0% |
| UCI2219330 | Lumpectomy | 100 | 0 | NA | | IDC | I | 0% | 35% | 60% | 5% | 0% |
| UCI2076630 | Mastectomy | 95 | 20 | 2.8 | | IDC | I | 5% | 45% | 30% | 20% | 0% |
| AA0960 | Lumpectomy | 80 | <10 | | 0 | IDC | I | 0% | 60% | 40% | 0% | 0% |
| UCI1804937 | Mastectomy | 100 | 50 | 1.2 | | IDC | I | 0% | 65% | 30% | 5% | 0% |
| AA0957 | Mastectomy | 100 | 50 | | 0 | IDC +DCIS | I | 10% | 70% | 20% | 0% | 0% |
| AA0926 | Lumpectomy | 90 | 50 | 1.47 | | IDC | I | 0% | 90% | 5% | 5% | 0% |
| UCI1951813 | Mastectomy | 100 | 0 | 1 | | IDC | II | 0% | 10% | 75% | 5% | 10% |
| UCI2649875 | Lumpectomy | 95 | 40 | 1.1 | | IDC | II | 0% | 25% | 50% | 5% | 20% |
| AA1267 | Lumpectomy | 100 | 95 | 1.05 | 2+ | IDC+LF | II | 0% | 30% | 65% | 5% | 0% |
| UCI1546879 | Mastectomy | 70 | 0 | 1.5 | | IDC | II | 10% | 25% | 50% | 5% | 10% |
| AA1090 | Lumpectomy | 100 | <5 | | 0 | IDC | II | 0% | 40% | 50% | 10% | 0% |
| UCI2564879 | Mastectomy | 90 | 90 | 1.2 | | IDC | II | 20% | 30% | 40% | 5% | 5% |
| UCI8965412 | Mastectomy | 80 | 90 | 6.7 | | IDC | II | 10% | 45% | 30% | 5% | 10% |
| UCI1908503 | Lymphadenectomy | 0 | 0 | 7.7 | | IDC | II | 0% | 60% | 10% | 10% | 20% |
| UCI9135402 | Mastectomy | 90 | 90 | 1.4 | | IDC+LF | II | 10% | 50% | 35% | 5% | 0% |
| AA1025 | Mastectomy | 100 | 30 | | 0 | IDC+LF+ DCIS | II | 10% | 50% | 20% | 10% | 10% |
| AA1188 | Mastectomy | 85 | 25 | | 0 | ILC +LCIS | II | 10% | 50% | 35% | 5% | 0% |
| UCI2224680 | Mastectomy | 90 | 80 | 1.2 | | IDC | II | 0% | 70% | 25% | 5% | 0% |
| UCI1689380 | Lumpectomy | 100 | 70 | 1.3 | | IDC | II | 0% | 75% | 20% | 5% | 0% |
| UCI3165498 | Mastectomy | 100 | 95 | 1.4 | | IDC | II | 0% | 75% | 15% | 10% | 0% |
| UCI2231576 | Mastectomy | 100 | 100 | 1.4 | | IDC | II | 0% | 80% | 15% | 5% | 0% |
| AA0952 | Lumpectomy | 95 | 30 | | 0 | IDC | II | 0% | 90% | 5% | 5% | 0% |
| AA0943 | Lumpectomy | >90 | 30 | 1.4 | | IDC+LF | III | 0% | 20% | 75% | 5% | 0% |
| AA1204 | Mastectomy | 0 | 0 | | 3+ | DCIS | III | 20% | 0% | 70% | 10% | 0% |
| UCI3564897 | Mastectomy | 0 | 0 | 2.3 | | IDC | III | 20% | 10% | 0% | 70% | 0% |
| UCI1821517 | Lumpectomy | 0 | 0 | 1.1 | | IDC | III | 0% | 40% | 40% | 20% | 0% |
| AA1307 | Lumpectomy | 0 | 0 | | 0 | IDC | III | 0% | 40% | 0% | 10% | 50% |
| AA1367 | Mastectomy | 0 | 0 | 1.3 | 1+ | IDC | III | 0% | 50% | 40% | 5% | 5% |
| AA1077 | Mastectomy | 100 | 0 | | 0 | IDC | III | 0% | 70% | 30% | 0% | 0% |
| AA1247 | Mastectomy | 0 | 0 | | 3+ | IDC | III | 0% | 70% | 30% | 0% | 0% |
| AA1277 | Mastectomy | 0 | 0 | | 0 | IDC | III | 0% | 70% | 30% | 0% | 0% |
| AA1222 | Lumpectomy | 90 | 60 | 3.5 | 2+ | IDC+LF | III | 0% | 80% | 10% | 10% | 0% |
| AA1106 | Mastectomy | 100 | 5 | | 0 | ILC +LCIS | III | 10% | 80% | 5% | 5% | 0% |
| UCI2008866 | Mastectomy | 0 | 0 | 1.4 | | IDC | III | 0% | 97% | 1% | 1% | 1% |
| UCI4216548 | Brain metastasis | 0 | 0 | 1.1 | | IDC | NA | 0% | 50% | 35% | 5% | 10% |
| AA1515 | Liver metastasis | 100 | 30 | 1 | 1+ | IDC+DCIS | NA | 0% | 50% | 40% | 10% | 0% |

* IDC: Invasise Ductal Carcinoma; DCIS: Ductal Carcinoma In Situ; LF: Lobular features; ILC: Invasive Lobular Carconima; LCIS, Lobular Carcinoma In Situ

**Table S2: List of Primers used in the study**

| Primer ID | Amplicons coodrinates (hg19) | Primer sequence (without UDT-Seq tail) |
|---|---|---|
| RB1.11.14.RB1.1_F | chr13:48942526-48942776 | GCAGCAGCTGGGTCATCTAT |
| RB1.11.14.RB1.1_R | chr13:48942526-48942776 | TGAAACACTATAAAGCCATGAATAACA |
| RB1.7.14.RB1.1_F | chr13:48934101-48934338 | TCTACCCTGCGATTTTCTCTCA |
| RB1.7.14.RB1.1_R | chr13:48934101-48934338 | TCCTGTCAGCCTTAGAACCA |
| RB1.26.1.RB1.1_F | chr13:49051432-49051585 | CGAAAGCATCATAGTTACTGGA |
| RB1.26.1.RB1.1_R | chr13:49051432-49051585 | CGAAAAGACTTCTTGCAGTGT |
| RB1.2.14.RB1.1_F | chr13:48881426-48881667 | TTTTCATTTGGTAGGCTTGAGTTT |
| RB1.2.14.RB1.1_R | chr13:48881426-48881667 | AGCAGAGGTAAATTTCCTCTGGG |
| RB1.2.15.RB1.2_F | chr13:48881341-48881484 | CACAGTAGTGTTATGTGCAAAC |
| RB1.2.15.RB1.2_R | chr13:48881341-48881484 | ACCAAGCTCTCTCTCTGACA |
| RB1.17.5.RB1.1_F | chr13:48955446-48955604 | CCCATGGATTCTGAATGTGC |
| RB1.17.5.RB1.1_R | chr13:48955446-48955604 | AGCCATATGCACATGAATGAA |
| RB1.17.6.RB1.2_F | chr13:48955344-48955495 | AAAAATACCTAGCTCAAGGGTT |
| RB1.17.6.RB1.2_R | chr13:48955344-48955495 | CTTGTCAAGTTGCCTTCTGC |
| RB1.22.1.RB1.1_F | chr13:49039141-49039283 | ACTGTTCTTCCTCAGACATTCA |
| RB1.22.1.RB1.1_R | chr13:49039141-49039283 | GTTTTGGTGGACCCATTACA |
| RB1.22.11.RB1.2_F | chr13:49039023-49039149 | TGTGCTTCTTACCAGTCAAAA |
| RB1.22.11.RB1.2_R | chr13:49039023-49039149 | GAATCATACTCCTCTTCTTTGATCA |
| RB1.1.7.RB1.1_F | chr13:48878026-48878180 | CTCCTCCACAGCTCGCT |
| RB1.1.7.RB1.1_R | chr13:48878026-48878180 | CTGCTCGCTCACCTGAC |
| RB1.1.8.RB1.2_F | chr13:48878026-48878185 | CTCCTCCACAGCTCGCT |
| RB1.1.8.RB1.2_R | chr13:48878026-48878185 | GGCTCTGCTCGCTCAC |
| RB1.18.9.RB1.1_F | chr13:49027137-49027266 | CATCATGTTTCATATAGGATTCACC |
| RB1.18.9.RB1.1_R | chr13:49027137-49027266 | TGCAGTTTGAATGGTCAACA |
| RB1.18.10.RB1.2_F | chr13:49027026-49027173 | ATTGCCACTGTCAATTGTGC |
| RB1.18.10.RB1.2_R | chr13:49027026-49027173 | TCAAGGTGATCAGTTGGTCC |
| RB1.16.14.RB1.1_F | chr13:48954201-48954434 | TCTGTTTCAGGAAGAAGAACGA |
| RB1.16.14.RB1.1_R | chr13:48954201-48954434 | CTCCCCCGACCAAAGAAACA |
| RB1.23.1.RB1.1_F | chr13:49039349-49039504 | TTTTTGCTCTAGCCCCCTAC |
| RB1.23.1.RB1.1_R | chr13:49039349-49039504 | TCCCTAAAGAGAAAACACACAC |
| RB1.23.2.RB1.2_F | chr13:49039216-49039368 | GGTCTTCATGCAGAGACTGA |
| RB1.23.2.RB1.2_R | chr13:49039216-49039368 | CTTGTAAGGGCTTCGAGGAA |
| RB1.13.1.RB1.1_F | chr13:48951063-48951217 | ACCTCCTAAAGAACTGCACA |
| RB1.13.1.RB1.1_R | chr13:48951063-48951217 | ACTGGAAAGATGCTGCTTTT |
| RB1.13.4.RB1.2_F | chr13:48951028-48951153 | TGATTACACAGTATCCTCGACA |
| RB1.13.4.RB1.2_R | chr13:48951028-48951153 | GTTACCTGTGATCCAATTTCGA |
| RB1.25.1.RB1.1_F | chr13:49050856-49051014 | AGACTTCTGAGAAGTTCCAGA |
| RB1.25.1.RB1.1_R | chr13:49050856-49051014 | GGATTCCCCAGATGACCATC |
| RB1.25.2.RB1.2_F | chr13:49050759-49050897 | TGCCTGATTTTTGACACACC |
| RB1.25.2.RB1.2_R | chr13:49050759-49050897 | GTTGCTTCCTTCAGCACTTC |
| RB1.27.1.RB1.1_F | chr13:49054098-49054228 | TTGCCAACTTACCCAGTACC |
| RB1.27.1.RB1.1_R | chr13:49054098-49054228 | ATCCAGAGGTGTACACAGTG |
| RB1.6.12.RB1.1_F | chr13:48923063-48923208 | TCTTTCAGTGATACATTTTTCCTGT |
| RB1.6.12.RB1.1_R | chr13:48923063-48923208 | AGTCCAAAGGAATGCCAATT |
| RB1.3.1.RB1.1_F | chr13:48916742-48916872 | TTTTTGTTCCCAGGGAGGTT |
| RB1.3.1.RB1.1_R | chr13:48916742-48916872 | TCCTTTTATGGCAGAGGCTT |
| RB1.3.2.RB1.2_F | chr13:48916603-48916755 | TGCCATCAGAAGGATGTGTT |
| RB1.3.2.RB1.2_R | chr13:48916603-48916755 | CAGATTCCCCACAGTTCCTT |
| RB1.9.12.RB1.1_F | chr13:48938984-48939139 | TGTTCAAGAGTCAAGAGATTAGAT |
| RB1.9.12.RB1.1_R | chr13:48938984-48939139 | CCCTCCACAGTCTCAAAACA |
| RB1.12.12.RB1.1_F | chr13:48947517-48947662 | CTCCCTTCATTGCTTAACACA |
| RB1.12.12.RB1.1_R | chr13:48947517-48947662 | AATTTCAAGTTTCTTTGCCAAGA |
| RB1.20.1.RB1.1_F | chr13:49033833-49033987 | TATTCCCACAGTGTATCGGC |
| RB1.20.1.RB1.1_R | chr13:49033833-49033987 | GTAGGGAGGAGAGAAGGTGA |
| RB1.20.2.RB1.2_F | chr13:49033696-49033855 | TGGGGGAAAGAAAAGAGTGG |
| RB1.20.2.RB1.2_R | chr13:49033696-49033855 | GACAGAAGGCGTTCACAAAG |
| RB1.14.1.RB1.1_F | chr13:48953649-48953806 | CTGGGCAAAACAGTGAGACT |
| RB1.14.1.RB1.1_R | chr13:48953649-48953806 | CGCCCGGCTGAAATTTTT |
| RB1.15.5.RB1.1_F | chr13:48954147-48954298 | TGCTGACACAAATAAGGTTTCA |
| RB1.15.5.RB1.1_R | chr13:48954147-48954298 | TGTTGTCATTCAGAAGTTTGCT |
| RB1.8.9.RB1.1_F | chr13:48936977-48937128 | CAGCTGTTATACCCATTAATGGT |
| RB1.8.9.RB1.1_R | chr13:48936977-48937128 | GGGAGAACTTACATCTAAATCTACT |
| RB1.8.12.RB1.2_F | chr13:48936932-48937053 | TGATGGATGTACAATTGTTCTTATC |
| RB1.8.12.RB1.2_R | chr13:48936932-48937053 | TCATGTTCTTTACAGAGAACTTCA |

| | | |
|---|---|---|
| RB1.4.12.RB1.1_F | chr13:48919225-48919376 | TCTTTCCTTTGTAGTGTCCATAA |
| RB1.4.12.RB1.1_R | chr13:48919225-48919376 | CAGAATCTAATTGTGAACAATGACA |
| RB1.4.13.RB1.2_F | chr13:48919122-48919272 | AGTAGTGATTTGATGTAGAGCTGA |
| RB1.4.13.RB1.2_R | chr13:48919122-48919272 | CTTCAACAGTCTTGACATAGCA |
| RB1.24.9.RB1.1_F | chr13:49047451-49047606 | TGCTCATCTCTGCAAAATTGT |
| RB1.24.9.RB1.1_R | chr13:49047451-49047606 | GGTGTTTGAATAACTGCATTTGG |
| RB1.19.1.RB1.1_F | chr13:49030369-49030524 | TCTCCTGTAAGATCTCCAAAGA |
| RB1.19.1.RB1.1_R | chr13:49030369-49030524 | TTTGAACCCAGTCAGCCTAG |
| RB1.19.2.RB1.2_F | chr13:49030277-49030434 | TGGGTGTACAACCTTGAAGT |
| RB1.19.2.RB1.2_R | chr13:49030277-49030434 | TTTCAATGGCTTCTGGGTCT |
| RB1.10.3.RB1.1_F | chr13:48941574-48941733 | TGAAATCTGTGCCTCTGTGT |
| RB1.10.3.RB1.1_R | chr13:48941574-48941733 | ACCATGTGCAATACCTGTCT |
| RB1.10.12.RB1.2_F | chr13:48941593-48941744 | TGTGCTGAGAGATGTAATGACA |
| RB1.10.12.RB1.2_R | chr13:48941593-48941744 | CAAATCAATCAAATATACCATGTGC |
| RB1.5.9.RB1.1_F | chr13:48921916-48922057 | GCATGAGAAAACTACTATGACTTCT |
| RB1.5.9.RB1.1_R | chr13:48921916-48922057 | ACCCTAACTATCAAGATGTTTGAG |
| CFTR.11.1.CFTR.1_F | chr7:117199584-117199739 | GGGTAAAATTAAGCACAGTGGA |
| CFTR.11.1.CFTR.1_R | chr7:117199584-117199739 | AGTGTGAAGGGTTCATATGCA |
| CFTR.11.2.CFTR.2_F | chr7:117199482-117199616 | AGTGAATCCTGAGCGTGATT |
| CFTR.11.2.CFTR.2_R | chr7:117199482-117199616 | TCTTTAATGGTGCCAGGCAT |
| CFTR.21.9.CFTR.1_F | chr7:117254659-117254805 | GGTTCATTTACGTCTTTTGTGC |
| CFTR.21.9.CFTR.1_R | chr7:117254659-117254805 | ACTCATACTTTGTTACTTGTCTGA |
| CFTR.7.10.CFTR.1_F | chr7:117176539-117176793 | GACTTAAAACCTTGAGCAGTTCTT |
| CFTR.7.10.CFTR.1_R | chr7:117176539-117176793 | TGAGGTGGAAGTCTACCATGA |
| CFTR.26.6.CFTR.1_F | chr7:117305495-117305636 | CTGTTCTGTGATATTATGTGTGGT |
| CFTR.26.6.CFTR.1_R | chr7:117305495-117305636 | CAAGGGCAATGAGATCTTAAGT |
| CFTR.2.7.CFTR.1_F | chr7:117144269-117144411 | TCCAAATCTGTATGGAGACCA |
| CFTR.2.7.CFTR.1_R | chr7:117144269-117144411 | CAATGTACATGAACATACCTTTCC |
| CFTR.2.8.CFTR.2_F | chr7:117144392-117144527 | TCCCTTCTGTTGATTCTGCT |
| CFTR.2.8.CFTR.2_R | chr7:117144392-117144527 | GCATTAGCCACCATACTTGG |
| CFTR.17.1.CFTR.1_F | chr7:117243693-117243847 | GTGTTTTACATTTACGTGGGAG |
| CFTR.17.1.CFTR.1_R | chr7:117243693-117243847 | CAGCAGTTTCATTTCTTAGACC |
| CFTR.17.2.CFTR.2_F | chr7:117243566-117243697 | ACTTTGGCTGCCAAATAACG |
| CFTR.17.2.CFTR.2_R | chr7:117243566-117243697 | CCCATAGCAAGCAAAGTGTC |
| CFTR.22.1.CFTR.1_F | chr7:117267594-117267755 | TGCGATCTGTGAGCCGA |
| CFTR.22.1.CFTR.1_R | chr7:117267594-117267755 | TCCACCTTCTGTGTATTTTGC |
| CFTR.22.2.CFTR.2_F | chr7:117267748-117267877 | GGGCCAAATGACTGTCAAAG |
| CFTR.22.2.CFTR.2_R | chr7:117267748-117267877 | ACACATTGCTTCAGGCTACT |
| CFTR.22.2.CFTR.3_F | chr7:117267468-117267612 | GCCCGACAAATAACCAAGTG |
| CFTR.22.2.CFTR.3_R | chr7:117267468-117267612 | AGGTTTACCTTCTGTTGGCA |
| CFTR.1.1.CFTR.1_F | chr7:117120105-117120257 | GGTCTTTGGCATTAGGAGCT |
| CFTR.1.1.CFTR.1_R | chr7:117120105-117120257 | CAAACCCAACCCATACACAC |
| CFTR.18.10.CFTR.1_F | chr7:117246643-117246899 | TGGGTTCTGAATGCGTCTACT |
| CFTR.18.10.CFTR.1_R | chr7:117246643-117246899 | AGACAGGACTTCAACCCTCA |
| CFTR.23.1.CFTR.1_F | chr7:117282505-117282648 | CTTATAGGTGGGCCTCTTGG |
| CFTR.23.1.CFTR.1_R | chr7:117282505-117282648 | TGGCTAAGTCCTTTTGCTCA |
| CFTR.23.2.CFTR.2_F | chr7:117282478-117282612 | TCACAGAAGTGATCCCATCA |
| CFTR.23.2.CFTR.2_R | chr7:117282478-117282612 | AAAGGCTTTCCTCCACTGTT |
| CFTR.16.1.CFTR.1_F | chr7:117242879-117243021 | ACTGTGTCTTGTTCCATTCCA |
| CFTR.16.1.CFTR.1_R | chr7:117242879-117243021 | CCACTACCATAATGCTTGGGA |
| CFTR.13.1.CFTR.1_F | chr7:117230382-117230538 | CCAGGAAATAGAGAGGAAATGT |
| CFTR.13.1.CFTR.1_R | chr7:117230382-117230538 | TGGGACAGTCTGTCTTTCTT |
| CFTR.6.1.CFTR.1_F | chr7:117175360-117175517 | CAAGTGGCACTCCTCATGG |
| CFTR.6.1.CFTR.1_R | chr7:117175360-117175517 | CCTGGTTTTACTAAAGTGGGC |
| CFTR.6.2.CFTR.2_F | chr7:117175288-117175434 | TGACACCTGTTTTTGCTGTG |
| CFTR.6.2.CFTR.2_R | chr7:117175288-117175434 | CATCATTCTCCCTAGCCCAG |
| CFTR.27.1.CFTR.1_F | chr7:117306992-117307152 | GAACAAAGTGCGGCAGTAC |
| CFTR.27.1.CFTR.1_R | chr7:117306992-117307152 | ATGCTGCTCTCTAAAGCCTT |
| CFTR.27.2.CFTR.2_F | chr7:117307024-117307182 | AAACTGCTGAACGAGAGGAG |
| CFTR.27.2.CFTR.2_R | chr7:117307024-117307182 | TCCATGAGCAAATGTCCCAT |
| CFTR.27.2.CFTR.3_F | chr7:117306853-117306997 | TTCAAAATGCAAGGCTCTGG |
| CFTR.27.2.CFTR.3_R | chr7:117306853-117306997 | TCGTTCAGCAGTTTCTGGAT |
| CFTR.25.1.CFTR.1_F | chr7:117304751-117304911 | AACTGCAGGTTGGGCTC |
| CFTR.25.1.CFTR.1_R | chr7:117304751-117304911 | CAGAACATCTGAAACTCACACT |
| CFTR.25.2.CFTR.2_F | chr7:117304805-117304963 | ACTTTGTCCTTGTGGATGGG |
| CFTR.25.2.CFTR.2_R | chr7:117304805-117304963 | TCACCATGAAGCAGGCATAA |
| CFTR.25.2.CFTR.3_F | chr7:117304621-117304779 | TTCAAATGGTGGCAGGTAGT |

| | | |
|---|---|---|
| CFTR.25.2.CFTR.3_R | chr7:117304621-117304779 | CCACAAGGACAAAGTCAAGC |
| CFTR.3.9.CFTR.1_F | chr7:117149072-117149196 | TGCAACTTATTGGTCCCACT |
| CFTR.3.9.CFTR.1_R | chr7:117149072-117149196 | ATGAATGTACAAATGAGATCCTTAC |
| CFTR.9.10.CFTR.1_F | chr7:117182011-117182185 | AGCACAATGAGAGTATAAAGTAGATGT |
| CFTR.9.10.CFTR.1_R | chr7:117182011-117182185 | AGAAAACAGTTAGGTGTTTAGAGCA |
| CFTR.12.9.CFTR.1_F | chr7:117227738-117227894 | GAAGGAAGATGTGCCTTTCA |
| CFTR.12.9.CFTR.1_R | chr7:117227738-117227894 | TGCTTGCTAGACCAATAATTAGT |
| CFTR.20.9.CFTR.1_F | chr7:117251714-117251868 | TTCGGACGGCAGCCTTA |
| CFTR.20.9.CFTR.1_R | chr7:117251714-117251868 | TGCTTAGCTAAAGTTAATGAGTTCA |
| CFTR.20.10.CFTR.2_F | chr7:117251504-117251752 | CAAAGAATGGCACCAGTGTGA |
| CFTR.20.10.CFTR.2_R | chr7:117251504-117251752 | AGGTACAAGAACCAGTTGGCA |
| CFTR.14.1.CFTR.1_F | chr7:117232228-117232387 | ACTGAGACCTTACACCGTTT |
| CFTR.14.1.CFTR.1_R | chr7:117232228-117232387 | TCAGAATCCTCTTCGATGCC |
| CFTR.14.2.CFTR.2_F | chr7:117232408-117232567 | GGCATCGAAGAGGATTCTGA |
| CFTR.14.2.CFTR.2_R | chr7:117232408-117232567 | GCTGTTGTCTTTCGGTGAAT |
| CFTR.14.2.CFTR.3_F | chr7:117232094-117232237 | TTTTGCATGAAGGTAGCAGC |
| CFTR.14.2.CFTR.3_R | chr7:117232094-117232237 | AGGAGACAGGAGCATCTCC |
| CFTR.14.3.CFTR.4_F | chr7:117232376-117232524 | CCATTGTGCAAAAGACTCCC |
| CFTR.14.3.CFTR.4_R | chr7:117232376-117232524 | GTGTCATCAGGTTCAGGACA |
| CFTR.14.3.CFTR.5_F | chr7:117232562-117232718 | ACACACTCAGTTAACCAAGGT |
| CFTR.14.3.CFTR.5_R | chr7:117232562-117232718 | AAATACCCCCAAGCGATGTA |
| CFTR.14.10.CFTR.6_F | chr7:117231942-117232154 | TGCTAAAATACGAGACATATTGCAA |
| CFTR.14.10.CFTR.6_R | chr7:117231942-117232154 | TGGTCGAAAGAATCACATCCCA |
| CFTR.15.4.CFTR.1_F | chr7:117234950-117235102 | TGGTGGCATGAAACTGTACT |
| CFTR.15.4.CFTR.1_R | chr7:117234950-117235102 | ACATTCTTACCTCTGCCAGA |
| CFTR.15.6.CFTR.2_F | chr7:117235034-117235167 | CCAGCAGTGACTACATGGAA |
| CFTR.15.6.CFTR.2_R | chr7:117235034-117235167 | GTGCATATATATGTATACATCCCCA |
| CFTR.8.1.CFTR.1_F | chr7:117180174-117180333 | AGAACAGAACTGAAACTGACTC |
| CFTR.8.1.CFTR.1_R | chr7:117180174-117180333 | CAGGGAAATTGCCGAGTG |
| CFTR.8.2.CFTR.2_F | chr7:117180325-117180466 | TCATTCTGCATTGTTCTGCG |
| CFTR.8.2.CFTR.2_R | chr7:117180325-117180466 | CGCACATTTTTGCAAAGTTC |
| CFTR.8.2.CFTR.3_F | chr7:117180087-117180220 | GACCATGCTCAGATCTTCCA |
| CFTR.8.2.CFTR.3_R | chr7:117180087-117180220 | AACACCACAAAGAACCCTGA |
| CFTR.4.1.CFTR.1_F | chr7:117171013-117171170 | TCATAGCTTCCTATGACCCG |
| CFTR.4.1.CFTR.1_R | chr7:117171013-117171170 | GGCCTGTGCAAGGAAGTATT |
| CFTR.4.2.CFTR.2_F | chr7:117170901-117171036 | CTTGTGTTGAAATTCTCAGGGT |
| CFTR.4.2.CFTR.2_R | chr7:117170901-117171036 | GCCTATGCCTAGATAAATCGC |
| CFTR.24.10.CFTR.1_F | chr7:117292784-117293014 | ACTTGATGGTAAGTACATGGGTGT |
| CFTR.24.10.CFTR.1_R | chr7:117292784-117293014 | TGTGTTGGTATGAGTTACCCCT |
| CFTR.19.1.CFTR.1_F | chr7:117250605-117250730 | TTGTGATTGGAGCTATAGCAGT |
| CFTR.19.1.CFTR.1_R | chr7:117250605-117250730 | AGATGAGTATCGCACATTCACT |
| CFTR.19.2.CFTR.2_F | chr7:117250528-117250686 | CACACTTTGTCCACTTTGCA |
| CFTR.19.2.CFTR.2_R | chr7:117250528-117250686 | TTGAGTTGCTGTGAGGTTTG |
| CFTR.10.4.CFTR.1_F | chr7:117188688-117188848 | GTGTGTGTGTGTGTGTTTTT |
| CFTR.10.4.CFTR.1_R | chr7:117188688-117188848 | AGTGGATCCAGCAACCG |
| CFTR.10.5.CFTR.2_F | chr7:117188810-117188939 | TCTTGGTACTCCTGTCCTGA |
| CFTR.10.5.CFTR.2_R | chr7:117188810-117188939 | ACCTTCCAGCACTACAAACT |
| CFTR.5.10.CFTR.1_F | chr7:117174195-117174425 | TGAACCTGAGAAGATAGTAAGCTAGA |
| CFTR.5.10.CFTR.1_R | chr7:117174195-117174425 | AGTGCCTAAAAGATTAAATCAATAGGT |
| FGFR1OP.6.7.FGFR1OP.1_F | chr6:167424258-167424400 | TGGAGTGTAATTGGTATATTTGTGT |
| FGFR1OP.6.7.FGFR1OP.1_R | chr6:167424258-167424400 | AAGTCCAGACATGACTTCCG |
| FGFR1OP.11.1.FGFR1OP.1_F | chr6:167446056-167446184 | GGTAGGTAAATTGTGACGCAC |
| FGFR1OP.11.1.FGFR1OP.1_R | chr6:167446056-167446184 | TTGTCTTGTCTGTGTTCGGA |
| FGFR1OP.3.5.FGFR1OP.1_F | chr6:167416662-167416789 | AGGGTAAACACATGACACTTAA |
| FGFR1OP.3.5.FGFR1OP.1_R | chr6:167416662-167416789 | TCCTGATTTCTCCCAAATGGT |
| FGFR1OP.7.1.FGFR1OP.1_F | chr6:167426903-167427056 | AGATGTTCCACTTCTGAGACA |
| FGFR1OP.7.1.FGFR1OP.1_R | chr6:167426903-167427056 | CTCTGCCCTCATGCATGTTA |
| FGFR1OP.9.1.FGFR1OP.1_F | chr6:167438257-167438398 | GAGCACCGATTAACTGTCCT |
| FGFR1OP.9.1.FGFR1OP.1_R | chr6:167438257-167438398 | TCCCGAGTATTGAAGTCCAC |
| FGFR1OP.12.8.FGFR1OP.1_F | chr6:167447203-167447461 | GCTGCTGCCCTTGCTACATT |
| FGFR1OP.12.8.FGFR1OP.1_R | chr6:167447203-167447461 | TGGGAAATGCAGAACACCAT |
| FGFR1OP.2.5.FGFR1OP.1_F | chr6:167413479-167413611 | TTGCTCGTGTTTGTTGCTG |
| FGFR1OP.2.5.FGFR1OP.1_R | chr6:167413479-167413611 | ACAGCCATGTTAAAATCTGGA |
| FGFR1OP.14.1.FGFR1OP.1_F | chr6:167453512-167453647 | AACAAGGACAGAGGACTGAC |
| FGFR1OP.14.1.FGFR1OP.1_R | chr6:167453512-167453647 | AAATCTTCCACACATGGGCT |
| FGFR1OP.8.1.FGFR1OP.1_F | chr6:167435927-167436086 | ATGAGGCCAATCAGAGTGAT |
| FGFR1OP.8.1.FGFR1OP.1_R | chr6:167435927-167436086 | TGGCTTAGGAATGGGATCAT |

| | | |
|---|---|---|
| FGFR1OP.8.2.FGFR1OP.2_F | chr6:167435984-167436138 | GCCTTCACTTACTGTCCCAT |
| FGFR1OP.8.2.FGFR1OP.2_R | chr6:167435984-167436138 | CCCCTAGTCTCATAGCTCAA |
| FGFR1OP.8.2.FGFR1OP.3_F | chr6:167435811-167435963 | GCACACACACAGGTTTGATT |
| FGFR1OP.8.2.FGFR1OP.3_R | chr6:167435811-167435963 | ATGGGACAGTAAGTGAAGGC |
| FGFR1OP.4.8.FGFR1OP.1_F | chr6:167417206-167417458 | TGCTTACTTACCTTTTTCTTGATCTT |
| FGFR1OP.4.8.FGFR1OP.1_R | chr6:167417206-167417458 | ACACTGGTGCCAGCTATCTG |
| FGFR1OP.1.5.FGFR1OP.1_F | chr6:167412901-167413040 | GGTTGTCTTGGAGAAGCAAG |
| FGFR1OP.1.5.FGFR1OP.1_R | chr6:167412901-167413040 | TGTCCACGCAAGCCCT |
| FGFR1OP.10.6.FGFR1OP.1_F | chr6:167439963-167440114 | AGCCCCATATTGAACAACGA |
| FGFR1OP.10.6.FGFR1OP.1_R | chr6:167439963-167440114 | ACGATCAGCACAAAAACTAGA |
| FGFR1OP.13.1.FGFR1OP.1_F | chr6:167453362-167453491 | AGACTACTCACATTCTGACACT |
| FGFR1OP.13.1.FGFR1OP.1_R | chr6:167453362-167453491 | GTCAGTCCTCTGTCCTTGTT |
| FGFR1OP.5.3.FGFR1OP.1_F | chr6:167417715-167417874 | CTCCTTGAGAATAACTTCTGCT |
| FGFR1OP.5.3.FGFR1OP.1_R | chr6:167417715-167417874 | TACTTCCCCAGTGGTTGG |
| FGFR1OP.5.4.FGFR1OP.2_F | chr6:167417788-167417942 | GTCGAGAGAATTTAGCCCGA |
| FGFR1OP.5.4.FGFR1OP.2_R | chr6:167417788-167417942 | TGCACTAATCTGCATGGCTA |
| BRCA1.11.18.BRCA1.1_F | chr17:41228514-41228663 | TGATGTTTCTTACCTTTCCACT |
| BRCA1.11.18.BRCA1.1_R | chr17:41228514-41228663 | ATCACTATCAGAACAAAGCAGT |
| BRCA1.11.20.BRCA1.2_F | chr17:41228407-41228543 | AGTGTATAAATGCCTGTATGCA |
| BRCA1.11.20.BRCA1.2_R | chr17:41228407-41228543 | AAGTTTGAGGTGTCTGCAGA |
| BRCA1.21.22.BRCA1.1_F | chr17:41258445-41258595 | TTCCTACTGTGGTTGCTTCC |
| BRCA1.21.22.BRCA1.1_R | chr17:41258445-41258595 | TGCCTTTTGAGTATTCTTTCTACA |
| BRCA1.7.1.BRCA1.1_F | chr17:41215879-41216031 | AGGGAGGAGGGGAGAAATAG |
| BRCA1.7.1.BRCA1.1_R | chr17:41215879-41216031 | ACTGAGGCTCTTTAGCTTCT |
| BRCA1.2.1.BRCA1.1_F | chr17:41199580-41199723 | ATGTGCCAAGAACTGTGCTA |
| BRCA1.2.1.BRCA1.1_R | chr17:41199580-41199723 | CTCTTTCCTTCCTGGGGATC |
| BRCA1.17.1.BRCA1.1_F | chr17:41249252-41249408 | GCTTCATAGACAAAGGTTCTCT |
| BRCA1.17.1.BRCA1.1_R | chr17:41249252-41249408 | GCCACAGTAGATGCTCAGTA |
| BRCA1.22.1.BRCA1.1_F | chr17:41267736-41267886 | TGGAGCCACATAACACATTCA |
| BRCA1.22.1.BRCA1.1_R | chr17:41267736-41267886 | TGACTCAGTCATAACAGCTCA |
| BRCA1.1.1.BRCA1.1_F | chr17:41197684-41197820 | CTGTGGCTCTGTACCTGTG |
| BRCA1.1.1.BRCA1.1_R | chr17:41197684-41197820 | GCTTGTGTTCTCTGTCTCCA |
| BRCA1.18.21.BRCA1.1_F | chr17:41251751-41251901 | GGCAAAACTATAAGATAAGGAATCC |
| BRCA1.18.21.BRCA1.1_R | chr17:41251751-41251901 | CTTGACTGTTCTTTACCATACTGT |
| BRCA1.23.23.BRCA1.1_F | chr17:41276029-41276253 | CCCAAATTAATACACTCTTGTGCTGA |
| BRCA1.23.23.BRCA1.1_R | chr17:41276029-41276253 | GGACGTTGTCATTAGTTCTTTGGT |
| BRCA1.13.1.BRCA1.1_F | chr17:41234447-41234599 | CTGATGTGCTTTGTTCTGGA |
| BRCA1.13.1.BRCA1.1_R | chr17:41234447-41234599 | TCTTGGTGCCATTTATCGTT |
| BRCA1.13.2.BRCA1.2_F | chr17:41234327-41234486 | TGTGCTGAGCAAGGATCATA |
| BRCA1.13.2.BRCA1.2_R | chr17:41234327-41234486 | GCCTTCTAACAGCTACCCTT |
| BRCA1.16.1.BRCA1.1_F | chr17:41247852-41247986 | TGCCTGTTAAGTTGGCAAAC |
| BRCA1.16.1.BRCA1.1_R | chr17:41247852-41247986 | GGTCATTTGACAGTTCTGCA |
| BRCA1.6.1.BRCA1.1_F | chr17:41215277-41215418 | GGAAAGTGGTGCATTGATGG |
| BRCA1.6.1.BRCA1.1_R | chr17:41215277-41215418 | GCACGTTCTTCTGCTGTATG |
| BRCA1.3.1.BRCA1.1_F | chr17:41201098-41201248 | GTCTTGCTCACAGGAGAGAA |
| BRCA1.3.1.BRCA1.1_R | chr17:41201098-41201248 | GCCTGGGTTAAGTATGCAGA |
| BRCA1.9.1.BRCA1.1_F | chr17:41222965-41223125 | TTCTTCTGGGGTCAGGC |
| BRCA1.9.1.BRCA1.1_R | chr17:41222965-41223125 | CTTCAACCTCTGCATTGAAAGT |
| BRCA1.9.16.BRCA1.2_F | chr17:41223089-41223248 | TCAGTAGTATGAGCAGCAGC |
| BRCA1.9.16.BRCA1.2_R | chr17:41223089-41223248 | CCATTCCTTTCAGAGGGAAC |
| BRCA1.9.17.BRCA1.3_F | chr17:41223244-41223377 | GCTGATTCCAGATTCCAGGT |
| BRCA1.9.17.BRCA1.3_R | chr17:41223244-41223377 | TGGATTTCCACCAACACTGT |
| BRCA1.9.21.BRCA1.4_F | chr17:41222917-41223068 | CCAGAATGTTGTTAAGTCTTAGTCA |
| BRCA1.9.21.BRCA1.4_R | chr17:41222917-41223068 | GCTGCTGCTCATACTACTGA |
| BRCA1.12.1.BRCA1.1_F | chr17:41231293-41231434 | ATTCCTCCTTTTGGCCAGAA |
| BRCA1.12.1.BRCA1.1_R | chr17:41231293-41231434 | CCGTTCCCTTGATGTCTACA |
| BRCA1.15.1.BRCA1.1_F | chr17:41243387-41243547 | TGTAAAATGTGCTCCCCAAA |
| BRCA1.15.1.BRCA1.1_R | chr17:41243387-41243547 | TCTGAAAGCCAGGGAGTTG |
| BRCA1.15.2.BRCA1.2_F | chr17:41243282-41243415 | AAGATGACGTCCTAGCTGTG |
| BRCA1.15.2.BRCA1.2_R | chr17:41243282-41243415 | TTTGTGTTTGCCCCAGTCTA |
| BRCA1.15.2.BRCA1.3_F | chr17:41243558-41243717 | ACTCAGACCAACTCCCTG |
| BRCA1.15.2.BRCA1.3_R | chr17:41243558-41243717 | GCAGTAACCAGGTAATATTGGC |
| BRCA1.15.3.BRCA1.4_F | chr17:41243438-41243596 | GACTGGGGCAAACACAAAAA |
| BRCA1.15.3.BRCA1.4_R | chr17:41243438-41243596 | ACACCCAGGATCCTTTCTTG |
| BRCA1.15.3.BRCA1.5_F | chr17:41243808-41243967 | TAGACAGACACTCGGTAGCA |
| BRCA1.15.3.BRCA1.5_R | chr17:41243808-41243967 | CAGGAGTCCTAGCCCTTTCA |
| BRCA1.15.4.BRCA1.6_F | chr17:41243657-41243808 | TCCAATTCACTGCACTGTGA |

| | | |
|---|---|---|
| BRCA1.15.4.BRCA1.6_R | chr17:41243657-41243808 | GTCTACTAGGCATAGCACCG |
| BRCA1.15.4.BRCA1.7_F | chr17:41244108-41244267 | ACAGGTCATCAGGTGTCTCA |
| BRCA1.15.4.BRCA1.7_R | chr17:41244108-41244267 | GGTTTTGCAACCTGAGGTCT |
| BRCA1.15.5.BRCA1.8_F | chr17:41243946-41244107 | TGGCCCCTCTTCGGTAA |
| BRCA1.15.5.BRCA1.8_R | chr17:41243946-41244107 | TCATGCATCTCAGGTTTGTTC |
| BRCA1.15.5.BRCA1.9_F | chr17:41244708-41244867 | GACAAAACCTAGAGCCTCCT |
| BRCA1.15.5.BRCA1.9_R | chr17:41244708-41244867 | CCACTCTGGGTCCTTAAAGA |
| BRCA1.15.6.BRCA1.10_F | chr17:41244957-41245117 | GCAAATGACTGGCGCTTT |
| BRCA1.15.6.BRCA1.10_R | chr17:41244957-41245117 | CAGTGTGCAGCATTTGAAAAC |
| BRCA1.15.6.BRCA1.11_F | chr17:41244608-41244740 | TTGATGGGAAAAAGTGGTGG |
| BRCA1.15.6.BRCA1.11_R | chr17:41244608-41244740 | CCTGTGGTTGGTCAGAAAGA |
| BRCA1.15.7.BRCA1.12_F | chr17:41245148-41245307 | TGCACACTGACTCACACA |
| BRCA1.15.7.BRCA1.12_R | chr17:41245148-41245307 | TGTCTAATAATGCTGAAGACCC |
| BRCA1.15.7.BRCA1.13_F | chr17:41244487-41244644 | GGCTAATTGTGCTCACTGTAC |
| BRCA1.15.7.BRCA1.13_R | chr17:41244487-41244644 | CGAAACTGGACTCATTACTCC |
| BRCA1.15.8.BRCA1.14_F | chr17:41245033-41245166 | TATGCTTGTTTCCCGACTGT |
| BRCA1.15.8.BRCA1.14_R | chr17:41245033-41245166 | GTTAGCACTCTAGGGAAGGC |
| BRCA1.15.8.BRCA1.15_F | chr17:41244348-41244502 | TCTACCTAGTTCTGCTTGAATG |
| BRCA1.15.8.BRCA1.15_R | chr17:41244348-41244502 | TCACCTGAAAGAGAAATGGGA |
| BRCA1.15.8.BRCA1.16_F | chr17:41245387-41245547 | TGGAAGGCTAGGATTGACAA |
| BRCA1.15.8.BRCA1.16_R | chr17:41245387-41245547 | GGCACAGCAGAAACCTACA |
| BRCA1.15.9.BRCA1.17_F | chr17:41245292-41245442 | TGCAAAACCCTTTCTCCACT |
| BRCA1.15.9.BRCA1.17_R | chr17:41245292-41245442 | ACTTTCCCAGAGCTGAAGTT |
| BRCA1.15.9.BRCA1.18_F | chr17:41245568-41245729 | TAGGTTTCTGCTGTGCCT |
| BRCA1.15.9.BRCA1.18_R | chr17:41245568-41245729 | TCCACAATTCAAAAGCACCT |
| BRCA1.15.10.BRCA1.19_F | chr17:41245538-41245692 | CCAGTTGCAGGTTCTTTACC |
| BRCA1.15.10.BRCA1.19_R | chr17:41245538-41245692 | GAGGAAGTCTTCTACCAGGC |
| BRCA1.15.10.BRCA1.20_F | chr17:41245750-41245909 | AGGTGCTTTTGAATTGTGGA |
| BRCA1.15.10.BRCA1.20_R | chr17:41245750-41245909 | AGCAGAATGGTCAAGTGATG |
| BRCA1.15.11.BRCA1.21_F | chr17:41245655-41245789 | TCAGTACAATTAGGTGGGCT |
| BRCA1.15.11.BRCA1.21_R | chr17:41245655-41245789 | TCAAAACGAAAGCTGAACCT |
| BRCA1.15.11.BRCA1.22_F | chr17:41245980-41246139 | CAGGAGTCTTTTGAACTGCC |
| BRCA1.15.11.BRCA1.22_R | chr17:41245980-41246139 | AACCTATCGGAAGAAGGCAA |
| BRCA1.15.12.BRCA1.23_F | chr17:41246139-41246300 | TGGCTTAAGTTGGGGAGG |
| BRCA1.15.12.BRCA1.23_R | chr17:41246139-41246300 | GCTGATGTATTGGACGTTCT |
| BRCA1.15.12.BRCA1.24_F | chr17:41245847-41245997 | CGAGTGATTCTATTGGGTTAGG |
| BRCA1.15.12.BRCA1.24_R | chr17:41245847-41245997 | GGCCTTCATCCTGAGGATTT |
| BRCA1.15.13.BRCA1.25_F | chr17:41246341-41246501 | GCTACTTTGGCATTTGATTCAG |
| BRCA1.15.13.BRCA1.25_R | chr17:41246341-41246501 | GCTGATCCCCTGTGTGA |
| BRCA1.15.14.BRCA1.26_F | chr17:41246208-41246343 | ACTCTCTACTGATTTGGAGTGA |
| BRCA1.15.14.BRCA1.26_R | chr17:41246208-41246343 | TGATGACTCACATGATGGGG |
| BRCA1.15.14.BRCA1.27_F | chr17:41246522-41246681 | CACACAGGGGATCAGCATT |
| BRCA1.15.14.BRCA1.27_R | chr17:41246522-41246681 | TTACAGCATGAGAACAGCAG |
| BRCA1.15.15.BRCA1.28_F | chr17:41246479-41246611 | ATTCTCTGAGCATGGCAGTT |
| BRCA1.15.15.BRCA1.28_R | chr17:41246479-41246611 | ATAAAAGCAAACAGCCTGGC |
| BRCA1.15.16.BRCA1.29_F | chr17:41246632-41246791 | GCCAGGCTGTTTGCTTTTAT |
| BRCA1.15.16.BRCA1.29_R | chr17:41246632-41246791 | GAACACCACTGAGAAGCGT |
| BRCA1.15.17.BRCA1.30_F | chr17:41246789-41246949 | TTTCTGGATGCCTCTCAGC |
| BRCA1.15.17.BRCA1.30_R | chr17:41246789-41246949 | TGGTTGATTTCCACCTCCAA |
| BRCA1.15.18.BRCA1.31_F | chr17:41244256-41244382 | TGCTTACAATTACTTCCAGGAA |
| BRCA1.15.18.BRCA1.31_R | chr17:41244256-41244382 | TCCAGTACTAATGAAGTGGGC |
| BRCA1.15.21.BRCA1.32_F | chr17:41244862-41245012 | CACATTCAAAAGTGACTTTTGGAC |
| BRCA1.15.21.BRCA1.32_R | chr17:41244862-41245012 | ACAGTCGGGAAACAAGCATA |
| BRCA1.14.1.BRCA1.1_F | chr17:41242926-41243082 | CTGAATGCAAAGGACACCAC |
| BRCA1.14.1.BRCA1.1_R | chr17:41242926-41243082 | TGCAGCGTTTATAGTCTGCT |
| BRCA1.20.1.BRCA1.1_F | chr17:41256858-41256991 | TTCATGGACAGCACTTGAGT |
| BRCA1.20.1.BRCA1.1_R | chr17:41256858-41256991 | CACTTGCTGAGTGTGTTTCT |
| BRCA1.8.1.BRCA1.1_F | chr17:41219588-41219743 | GCCTCATGTGGTTTTATGCA |
| BRCA1.8.1.BRCA1.1_R | chr17:41219588-41219743 | GCTGTGTGCTAGAGGTAACT |
| BRCA1.4.1.BRCA1.1_F | chr17:41202997-41203143 | TTCAGCAATCTGAGGAACCC |
| BRCA1.4.1.BRCA1.1_R | chr17:41202997-41203143 | TGTCCCTCTCTCTTCCTCTC |
| BRCA1.24.1.BRCA1.1_F | chr17:41277065-41277207 | TCCAGTTCCTATCACGAGGA |
| BRCA1.24.1.BRCA1.1_R | chr17:41277065-41277207 | GAGAGTGGATTTCCGAAGCT |
| BRCA1.19.18.BRCA1.1_F | chr17:41256158-41256291 | CCAAGGAAGGATTTTCGGGT |
| BRCA1.19.18.BRCA1.1_R | chr17:41256158-41256291 | ACTGCAAACATAATGTTTTCCC |
| BRCA1.19.19.BRCA1.2_F | chr17:41256028-41256167 | CAGCTACTAAGGGGGCTAAG |
| BRCA1.19.19.BRCA1.2_R | chr17:41256028-41256167 | AAACCGTGCCAAAAGACTTC |

| | | |
|---|---|---|
| BRCA1.10.1.BRCA1.1_F | chr17:41226393-41226551 | TGTTTCCGTCAAATCGTGTG |
| BRCA1.10.1.BRCA1.1_R | chr17:41226393-41226551 | GCGATGGTTTTCTCCTTCCA |
| BRCA1.10.2.BRCA1.2_F | chr17:41226337-41226479 | GTGTTTGTTCCAATACAGCAGA |
| BRCA1.10.2.BRCA1.2_R | chr17:41226337-41226479 | GTACATGCACAGTTGCTCTG |
| BRCA1.5.1.BRCA1.1_F | chr17:41209069-41209212 | CTTGAGGGAGGGAGCTTTAC |
| BRCA1.5.1.BRCA1.1_R | chr17:41209069-41209212 | ATATGACGTGTCTGCTCCAC |
| ERBB4.11.11.ERBB4.1_F | chr2:212488516-212488770 | TCATTTGTGCAGCAACTTCTCA |
| ERBB4.11.11.ERBB4.1_R | chr2:212488516-212488770 | CCTTCTTCTTTCCGCTTTGCA |
| ERBB4.21.1.ERBB4.1_F | chr2:212576791-212576918 | CTGACCCATGAATACCAGTGA |
| ERBB4.21.1.ERBB4.1_R | chr2:212576791-212576918 | AGTTTCTCAATCCCCTAACTCT |
| ERBB4.21.2.ERBB4.2_F | chr2:212576694-212576850 | AACTCTTCAGCTTCCAGAGG |
| ERBB4.21.2.ERBB4.2_R | chr2:212576694-212576850 | GTCAGCTCAGACTGTGGATT |
| ERBB4.7.8.ERBB4.1_F | chr2:212293122-212293275 | ACAAGCTTTAATTCGCAAAGAA |
| ERBB4.7.8.ERBB4.1_R | chr2:212293122-212293275 | CCAGCCCAAAGACTCACATT |
| ERBB4.26.4.ERBB4.1_F | chr2:212652718-212652865 | CGCCACATAGGGTAGAACAT |
| ERBB4.26.4.ERBB4.1_R | chr2:212652718-212652865 | CAGAAATCCTAAATGGTGGAGT |
| ERBB4.26.5.ERBB4.2_F | chr2:212652837-212652986 | CCAATGAATGGTGTCTGCAT |
| ERBB4.26.5.ERBB4.2_R | chr2:212652837-212652986 | TGGCTATTAGCAACATGACTCT |
| ERBB4.17.1.ERBB4.1_F | chr2:212543775-212543925 | GCAACCCTAGAAGAAGCCTT |
| ERBB4.17.1.ERBB4.1_R | chr2:212543775-212543925 | CCTCTGTTTTATTTCCCCTTGA |
| ERBB4.2.1.ERBB4.1_F | chr2:212251597-212251757 | CCTTGTTTGGGTTTGTCTCG |
| ERBB4.2.1.ERBB4.1_R | chr2:212251597-212251757 | AGGGTGCTACTGCTGAGA |
| ERBB4.2.2.ERBB4.2_F | chr2:212251752-212251897 | GCCATTACAGCAGGAGTCAT |
| ERBB4.2.2.ERBB4.2_R | chr2:212251752-212251897 | CTAACAACGCCTTCTCTCCA |
| ERBB4.2.2.ERBB4.3_F | chr2:212251469-212251607 | TAGCCTAGGCCTTTACTGGA |
| ERBB4.2.2.ERBB4.3_R | chr2:212251469-212251607 | GCTGGATGAGGAAGGTTACA |
| ERBB4.22.1.ERBB4.1_F | chr2:212578260-212578415 | AATAATCTGAGCTACCACTCAC |
| ERBB4.22.1.ERBB4.1_R | chr2:212578260-212578415 | TGAGCTTGTTTGCTGAATGT |
| ERBB4.1.1.ERBB4.1_F | chr2:212248370-212248529 | GTATTCCGGTGTCTGTAAGG |
| ERBB4.1.1.ERBB4.1_R | chr2:212248370-212248529 | CCTGACTACTGGAACCACAG |
| ERBB4.1.2.ERBB4.2_F | chr2:212248324-212248479 | AGGTGTGTCTCTCCACCTAA |
| ERBB4.1.2.ERBB4.2_R | chr2:212248324-212248479 | AGACTACCTGCAGGAGTACA |
| ERBB4.1.2.ERBB4.3_F | chr2:212248580-212248740 | CTTTCTTGGCCTTCTCTGG |
| ERBB4.1.2.ERBB4.3_R | chr2:212248580-212248740 | CCCTTTTGTTTCTCGGAGAA |
| ERBB4.1.3.ERBB4.4_F | chr2:212248661-212248804 | AGGTACAGTGGCTCATTCAC |
| ERBB4.1.3.ERBB4.4_R | chr2:212248661-212248804 | CCAGAAACTAGAGGTTAGCTGA |
| ERBB4.1.3.ERBB4.5_F | chr2:212248500-212248636 | TGTACTCCTGCAGGTAGTCT |
| ERBB4.1.3.ERBB4.5_R | chr2:212248500-212248636 | ATGAGCCACTGTACCTCAAC |
| ERBB4.18.1.ERBB4.1_F | chr2:212566737-212566892 | CCCGGATTACTATTCTCTGGT |
| ERBB4.18.1.ERBB4.1_R | chr2:212566737-212566892 | TTCTCACTTCCCCCTCCTTA |
| ERBB4.18.2.ERBB4.2_F | chr2:212566593-212566743 | AAGGGTTGGGATAACAGAGC |
| ERBB4.18.2.ERBB4.2_R | chr2:212566593-212566743 | ACTGGACAACACTCTTCAGC |
| ERBB4.13.6.ERBB4.1_F | chr2:212522455-212522584 | AGGAAAGGATTTGAGCGACA |
| ERBB4.13.6.ERBB4.1_R | chr2:212522455-212522584 | CATGTCACTGTGGATGGAGA |
| ERBB4.23.6.ERBB4.1_F | chr2:212587147-212587306 | TCTTGACACAGAATGCTCCA |
| ERBB4.23.6.ERBB4.1_R | chr2:212587147-212587306 | GGCAGCGTTTCATTAACAAG |
| ERBB4.23.7.ERBB4.2_F | chr2:212587007-212587157 | GGGGGCAATAGTATCTATACCC |
| ERBB4.23.7.ERBB4.2_R | chr2:212587007-212587157 | TGGAGCACAATTTCAATGCA |
| ERBB4.16.1.ERBB4.1_F | chr2:212537875-212538009 | TAAGCCAACACACCACAGAT |
| ERBB4.16.1.ERBB4.1_R | chr2:212537875-212538009 | TGACGCTTGTTTGCTTACAC |
| ERBB4.29.6.ERBB4.1_F | chr2:213403104-213403256 | GATGGGTGAAGAGGGCA |
| ERBB4.29.6.ERBB4.1_R | chr2:213403104-213403256 | CGGGATCTGAGACTTCCAAA |
| ERBB4.25.1.ERBB4.1_F | chr2:212615308-212615446 | AGAGGCATGAAATGGACCAA |
| ERBB4.25.1.ERBB4.1_R | chr2:212615308-212615446 | AACGTGCATGTCCTTTTTCC |
| ERBB4.27.1.ERBB4.1_F | chr2:212812194-212812343 | CCAAGTTCTTGAAGTCCAAAGT |
| ERBB4.27.1.ERBB4.1_R | chr2:212812194-212812343 | GGCAACTGTTTGTGTCTTTTC |
| ERBB4.27.6.ERBB4.2_F | chr2:212812068-212812223 | TGCCTTAGAGTGTTCCTCAA |
| ERBB4.27.6.ERBB4.2_R | chr2:212812068-212812223 | TGAGGATCGATATGCCTTGG |
| ERBB4.6.1.ERBB4.1_F | chr2:212288920-212289069 | ACGTCAATAGTGCAGATGGG |
| ERBB4.6.1.ERBB4.1_R | chr2:212288920-212289069 | AGTGCTGGTTTGTTCAACAT |
| ERBB4.6.2.ERBB4.2_F | chr2:212288763-212288922 | GGCGTTCATATGTTCCTTTCA |
| ERBB4.6.2.ERBB4.2_R | chr2:212288763-212288922 | AGGAGAACGTTTGCCTCAG |
| ERBB4.28.1.ERBB4.1_F | chr2:212989472-212989629 | AAGAGAAAGAAAGCCACAGC |
| ERBB4.28.1.ERBB4.1_R | chr2:212989472-212989629 | CGTGTATCTTCTTGTCTTTGCA |
| ERBB4.3.6.ERBB4.1_F | chr2:212252632-212252791 | ACATGGTAAGCAAAGACCGA |
| ERBB4.3.6.ERBB4.1_R | chr2:212252632-212252791 | ACCCATGTGGCTTTTCTTTT |
| ERBB4.9.4.ERBB4.1_F | chr2:212426580-212426738 | TGAAAACTGTTCCAGGTTAGGA |

| | | |
|---|---|---|
| ERBB4.9.4.ERBB4.1_R | chr2:212426580-212426738 | GTGTCTGAGCCCAACCATC |
| ERBB4.9.5.ERBB4.2_F | chr2:212426730-212426874 | CCATGGGGCATAAGTTGAGT |
| ERBB4.9.5.ERBB4.2_R | chr2:212426730-212426874 | TCAGCACCATTAGTACAATCCA |
| ERBB4.12.1.ERBB4.1_F | chr2:212495200-212495356 | ACCTCACCTCTGTTTCCAAG |
| ERBB4.12.1.ERBB4.1_R | chr2:212495200-212495356 | TCCTCTGTCTTCTCTCTCTTCA |
| ERBB4.12.2.ERBB4.2_F | chr2:212495171-212495305 | CCCCAAACACATGAAGAGGA |
| ERBB4.12.2.ERBB4.2_R | chr2:212495171-212495305 | TGTCAGAACTCCCCTGATTG |
| ERBB4.20.9.ERBB4.1_F | chr2:212569963-212570118 | CTGGGATTACTGGTGTGAGC |
| ERBB4.20.9.ERBB4.1_R | chr2:212569963-212570118 | GGCTACATCTCTTCTTGATTTTCT |
| ERBB4.14.1.ERBB4.1_F | chr2:212522675-212522833 | GAAGCAAGCACACCAAGTAC |
| ERBB4.14.1.ERBB4.1_R | chr2:212522675-212522833 | CCTGGCTTGAAATTCATTGTG |
| ERBB4.15.1.ERBB4.1_F | chr2:212530078-212530239 | TTTGGATGGCATGGGTGG |
| ERBB4.15.1.ERBB4.1_R | chr2:212530078-212530239 | TCCAGAGGAAATGACACCAC |
| ERBB4.15.2.ERBB4.2_F | chr2:212530026-212530183 | TAACACATACCAGGTGAGCC |
| ERBB4.15.2.ERBB4.2_R | chr2:212530026-212530183 | GGGTCCTGACAACTGTACAA |
| ERBB4.8.1.ERBB4.1_F | chr2:212295694-212295848 | TCCTCCATCAGCATTGTACTC |
| ERBB4.8.1.ERBB4.1_R | chr2:212295694-212295848 | CCTGGGCATTAACTGAATATCA |
| ERBB4.8.2.ERBB4.2_F | chr2:212295549-212295703 | CAGGAGAAGAGGCAAATGGT |
| ERBB4.8.2.ERBB4.2_R | chr2:212295549-212295703 | CTAGCCAGACTCTTGGAAGG |
| ERBB4.4.1.ERBB4.1_F | chr2:212285186-212285342 | ACCCTATTCGAGTCAATTCTTG |
| ERBB4.4.1.ERBB4.1_R | chr2:212285186-212285342 | GTGTCTGATGGGCAATCTTTC |
| ERBB4.4.2.ERBB4.2_F | chr2:212285039-212285195 | CACATCTTTTGTGGGTATGGT |
| ERBB4.4.2.ERBB4.2_R | chr2:212285039-212285195 | ACATCCCACCTCCCATCTAT |
| ERBB4.24.1.ERBB4.1_F | chr2:212589770-212589921 | TAGGGAAGGAAAGGAGAGCA |
| ERBB4.24.1.ERBB4.1_R | chr2:212589770-212589921 | GGTCCTATCTCTGTCTTCTGC |
| ERBB4.19.10.ERBB4.1_F | chr2:212568820-212568972 | GCCCTGCAGCTTTAAACATA |
| ERBB4.19.10.ERBB4.1_R | chr2:212568820-212568972 | CACCATATTTCAAGAAAAATCAGCA |
| ERBB4.10.10.ERBB4.1_F | chr2:212483891-212484028 | AAAATGTGATTGCCTGGGTG |
| ERBB4.10.10.ERBB4.1_R | chr2:212483891-212484028 | AGAACTTAATAGGTTGAGATGTCA |
| ERBB4.5.10.ERBB4.1_F | chr2:212286707-212286861 | TTTGCCATTGGCCTAGTCTT |
| ERBB4.5.10.ERBB4.1_R | chr2:212286707-212286861 | TTCAACTCCTTTCTAGTTACGAA |
| RARA.6.1.RARA.1_F | chr17:38508633-38508792 | GACATTGACCTCTGGGACAA |
| RARA.6.1.RARA.1_R | chr17:38508633-38508792 | ATCTACCCAGGACACAGGG |
| RARA.6.2.RARA.2_F | chr17:38508504-38508643 | GAAGTGAAGGCTGGGTAGAG |
| RARA.6.2.RARA.2_R | chr17:38508504-38508643 | AATGATGCACTTGGTGGAGA |
| RARA.3.1.RARA.1_F | chr17:38504567-38504721 | CCTCCCCTCTTCTCTCTCTA |
| RARA.3.1.RARA.1_R | chr17:38504567-38504721 | CTTCCCAATGACCCCTTCAA |
| RARA.7.1.RARA.1_F | chr17:38510571-38510735 | ATCCTGCGGATCTGCAC |
| RARA.7.1.RARA.1_R | chr17:38510571-38510735 | AGATGAGGCAGATGGCG |
| RARA.7.2.RARA.2_F | chr17:38510724-38510870 | GAGATGGATGATGCGGAGAC |
| RARA.7.2.RARA.2_R | chr17:38510724-38510870 | CTGTGTAGTTGCAGACCAGA |
| RARA.7.2.RARA.3_F | chr17:38510447-38510586 | TATCAGACAGCATTGCTCCG |
| RARA.7.2.RARA.3_R | chr17:38510447-38510586 | GAGAAGGTCATGGTGTCCTG |
| RARA.9.1.RARA.1_F | chr17:38512319-38512484 | TCCATGCCGCCTCTCAT |
| RARA.9.1.RARA.1_R | chr17:38512319-38512484 | CTGTGTCCATGTGGCGT |
| RARA.9.2.RARA.2_F | chr17:38512190-38512325 | ATTGATCTTCCCACCTCGAG |
| RARA.9.2.RARA.2_R | chr17:38512190-38512325 | CCCTCTGAGTTCTCCAACAT |
| RARA.2.1.RARA.1_F | chr17:38498964-38499124 | TTCGAGGGGAAAGATGTACG |
| RARA.2.1.RARA.1_R | chr17:38498964-38499124 | AGACTGAGAGGCCGGTA |
| RARA.2.2.RARA.2_F | chr17:38498957-38499106 | CTTGCCTTCGAGGGGAAAG |
| RARA.2.2.RARA.2_R | chr17:38498957-38499106 | TACGTACAGTGGTTTGAGCC |
| RARA.8.1.RARA.1_F | chr17:38511515-38511676 | GGTGGTTCTGCTTCCTCAG |
| RARA.8.1.RARA.1_R | chr17:38511515-38511676 | TCCAGGTCTGTGAGCCT |
| RARA.1.3.RARA.1_F | chr17:38487468-38487628 | TTCTGACTGTGGCCGCTT |
| RARA.1.3.RARA.1_R | chr17:38487468-38487628 | CTGGGGATGGTGTGCTA |
| RARA.1.4.RARA.2_F | chr17:38487624-38487757 | GCACCAGCTTCCAGTTAGT |
| RARA.1.4.RARA.2_R | chr17:38487624-38487757 | AACTGGAGAAGACCTTGTCG |
| RARA.4.1.RARA.1_F | chr17:38506046-38506208 | ATACCCAGGGCTTCTTCC |
| RARA.4.1.RARA.1_R | chr17:38506046-38506208 | TCTGAGACCCCAACTGAGG |
| RARA.4.2.RARA.2_F | chr17:38505894-38506048 | GTGAATCATTCTCCCCAGCT |
| RARA.4.2.RARA.2_R | chr17:38505894-38506048 | CATGTTCTTCTGGATGCTGC |
| RARA.10.1.RARA.1_F | chr17:38566870-38567012 | ATGGTGAGAAGCTACTGCAG |
| RARA.10.1.RARA.1_R | chr17:38566870-38567012 | GATTTGCCCATACAAGCAGC |
| RARA.5.1.RARA.1_F | chr17:38508182-38508341 | CTGTGAGAAACGACCGAAAC |
| RARA.5.1.RARA.1_R | chr17:38508182-38508341 | GCAAATCCCACCCTGCA |
| RARA.5.2.RARA.2_F | chr17:38508144-38508300 | TGCAGCTGCCCTCTTAAC |
| RARA.5.2.RARA.2_R | chr17:38508144-38508300 | TAGTGTATTTGCCCAGCTGG |

| | | |
|---|---|---|
| CTNNA1.11.1.CTNNA1.1_F | chr5:138260248-138260404 | TGTGTCATTGCTCTCCAAGA |
| CTNNA1.11.1.CTNNA1.1_R | chr5:138260248-138260404 | CACTGGAAAGGGAGAGTTCC |
| CTNNA1.11.2.CTNNA1.2_F | chr5:138260181-138260310 | AGCCTCCTTCCTCATTCAAC |
| CTNNA1.11.2.CTNNA1.2_R | chr5:138260181-138260310 | TCTCTGAGGTGACTACGTGA |
| CTNNA1.7.3.CTNNA1.1_F | chr5:138221837-138221986 | TTGACATGAGCACAAATGGC |
| CTNNA1.7.3.CTNNA1.1_R | chr5:138221837-138221986 | ACAATCAGCACTTTCATCCAG |
| CTNNA1.17.1.CTNNA1.1_F | chr5:138268565-138268698 | ACCAAGGTCTGTCCATCATC |
| CTNNA1.17.1.CTNNA1.1_R | chr5:138268565-138268698 | CCCAAGTAGCTGGGACTTAC |
| CTNNA1.2.1.CTNNA1.1_F | chr5:138118919-138119073 | GGCCCTCTAATAAGAAGAGAGG |
| CTNNA1.2.1.CTNNA1.1_R | chr5:138118919-138119073 | GCCTCTCTTTACCTAAAAAGCA |
| CTNNA1.2.2.CTNNA1.2_F | chr5:138118772-138118918 | TGTTCAGTGGAATCTCATGTGA |
| CTNNA1.2.2.CTNNA1.2_R | chr5:138118772-138118918 | CATGGGCCTTCTTAGAACGA |
| CTNNA1.1.1.CTNNA1.1_F | chr5:138117595-138117750 | TGATGTTTGCCTGACTGACT |
| CTNNA1.1.1.CTNNA1.1_R | chr5:138117595-138117750 | GGCCTGTGCTATAGAACCAT |
| CTNNA1.18.1.CTNNA1.1_F | chr5:138269521-138269681 | ATGTCCCTGATCCAGGCA |
| CTNNA1.18.1.CTNNA1.1_R | chr5:138269521-138269681 | TCTTGGTCTGTGTCTCATCC |
| CTNNA1.18.2.CTNNA1.2_F | chr5:138269660-138269808 | GAAGGCACCAGAGAAAAAGC |
| CTNNA1.18.2.CTNNA1.2_R | chr5:138269660-138269808 | GACTGATATTCAGGAGCCCC |
| CTNNA1.18.2.CTNNA1.3_F | chr5:138269405-138269564 | CTGGTCTTGCAGAGGAGTAG |
| CTNNA1.18.2.CTNNA1.3_R | chr5:138269405-138269564 | ATTTGGTAGAGGCGACGTAG |
| CTNNA1.16.1.CTNNA1.1_F | chr5:138268263-138268414 | GTGTTAAGCCTGCTCTCTCT |
| CTNNA1.16.1.CTNNA1.1_R | chr5:138268263-138268414 | CACTGGCCCTCTTTTTGTTC |
| CTNNA1.13.1.CTNNA1.1_F | chr5:138264930-138265088 | CCTGTGATCTTTGTCTGGGT |
| CTNNA1.13.1.CTNNA1.1_R | chr5:138264930-138265088 | CAAGAAAGGGGACAGGGAAA |
| CTNNA1.6.1.CTNNA1.1_F | chr5:138163224-138163383 | GAAACAAATCATTGTGGACCC |
| CTNNA1.6.1.CTNNA1.1_R | chr5:138163224-138163383 | CCATGTACTCCGAAAGCAG |
| CTNNA1.6.2.CTNNA1.2_F | chr5:138163363-138163515 | TTGTGGCAGAGTGTAATGCT |
| CTNNA1.6.2.CTNNA1.2_R | chr5:138163363-138163515 | ACCTACTCCCAAAAATCAAGGA |
| CTNNA1.6.2.CTNNA1.3_F | chr5:138163078-138163223 | GCCCCTTACCTGCTAAGAAG |
| CTNNA1.6.2.CTNNA1.3_R | chr5:138163078-138163223 | CTCCTCGCTGAAGCTCAAG |
| CTNNA1.3.1.CTNNA1.1_F | chr5:138145746-138145896 | GGTGATTTGATGAAGGCTGC |
| CTNNA1.3.1.CTNNA1.1_R | chr5:138145746-138145896 | ACAGACATAGGCCTGTATACT |
| CTNNA1.3.2.CTNNA1.2_F | chr5:138145597-138145747 | AGGAACAGCAAACAACAACA |
| CTNNA1.3.2.CTNNA1.2_R | chr5:138145597-138145747 | ATCATCTGCGAACTCTCCTG |
| CTNNA1.9.1.CTNNA1.1_F | chr5:138239983-138240141 | TAGGGCCAGGTGTGTGTTT |
| CTNNA1.9.1.CTNNA1.1_R | chr5:138239983-138240141 | TATGCTGTCACCAGTGTCAG |
| CTNNA1.12.1.CTNNA1.1_F | chr5:138260943-138261102 | TACTGTGCCTCTTTCTCCAC |
| CTNNA1.12.1.CTNNA1.1_R | chr5:138260943-138261102 | AAGACGTCTGAAATGCAGTT |
| CTNNA1.15.1.CTNNA1.1_F | chr5:138266491-138266625 | GTGAGGGGCTTCACATACAA |
| CTNNA1.15.1.CTNNA1.1_R | chr5:138266491-138266625 | CCTGGCAAGTCTGTCACTTA |
| CTNNA1.14.1.CTNNA1.1_F | chr5:138266171-138266331 | TCTGATCACAGGCGATCATG |
| CTNNA1.14.1.CTNNA1.1_R | chr5:138266171-138266331 | TGCTCACCGGGTAAAGTCT |
| CTNNA1.14.2.CTNNA1.2_F | chr5:138266299-138266434 | AATGACATCATTGTGCTGGC |
| CTNNA1.14.2.CTNNA1.2_R | chr5:138266299-138266434 | GAAGATTGTCCACAAAGCCC |
| CTNNA1.14.2.CTNNA1.3_F | chr5:138266045-138266189 | CCTTCAGGCGAAATGAAACC |
| CTNNA1.14.2.CTNNA1.3_R | chr5:138266045-138266189 | TTCCGCAATCTTCGCTTTTT |
| CTNNA1.8.1.CTNNA1.1_F | chr5:138223193-138223353 | ATTAGCTCCGCAAAGCTGT |
| CTNNA1.8.1.CTNNA1.1_R | chr5:138223193-138223353 | ACCAAGTTGCCTACAAGTCA |
| CTNNA1.8.2.CTNNA1.2_F | chr5:138223072-138223215 | TCCTCATGTAAGTGCAGCAA |
| CTNNA1.8.2.CTNNA1.2_R | chr5:138223072-138223215 | TGGAACATTGGTTTCCAGGA |
| CTNNA1.4.1.CTNNA1.1_F | chr5:138147863-138147991 | TGGGTTTTGGGGTCTTTCTT |
| CTNNA1.4.1.CTNNA1.1_R | chr5:138147863-138147991 | ATCCCCAAATCATGACTGTAC |
| CTNNA1.10.1.CTNNA1.1_F | chr5:138253446-138253598 | TGCAGGTTATTAATGCTGCA |
| CTNNA1.10.1.CTNNA1.1_R | chr5:138253446-138253598 | TCACATACTTCTCTGGGGAAC |
| CTNNA1.10.2.CTNNA1.2_F | chr5:138253360-138253518 | CATGTGGTGTGATGTCTCCT |
| CTNNA1.10.2.CTNNA1.2_R | chr5:138253360-138253518 | TGTGAGAACACGGACTTGTT |
| CTNNA1.5.1.CTNNA1.1_F | chr5:138160248-138160411 | ATGTTGGCCATCGTGATCA |
| CTNNA1.5.1.CTNNA1.1_R | chr5:138160248-138160411 | ATCGTCTGAGGCAGTGG |
| CTNNA1.5.2.CTNNA1.2_F | chr5:138160346-138160497 | TGATGTCGCAGCCTATAAGG |
| CTNNA1.5.2.CTNNA1.2_R | chr5:138160346-138160497 | CTGGAAATTTCCACCCAAGC |
| CTNNA1.5.2.CTNNA1.3_F | chr5:138160192-138160323 | AGCCCATATAAAGAGTGCTCC |
| CTNNA1.5.2.CTNNA1.3_R | chr5:138160192-138160323 | TTATAGGCTGCGACATCAGG |
| CDH1.6.1.CDH1.1_F | chr16:68844120-68844278 | TCTTCTCTCACGCTGTGTC |
| CDH1.6.1.CDH1.1_R | chr16:68844120-68844278 | GGTCCAAAGAACCTAAGAGTCT |
| CDH1.6.2.CDH1.2_F | chr16:68844059-68844197 | CCCCTTCTCCCATGTTTTCT |
| CDH1.6.2.CDH1.2_R | chr16:68844059-68844197 | CCTTAAAGACCTCCTGGGTG |
| CDH1.11.1.CDH1.1_F | chr16:68853182-68853337 | AGAGCTTGTCCCCGTTCA |

| | | |
|---|---|---|
| CDH1.11.1.CDH1.1_R | chr16:68853182-68853337 | AGCAAAGGCTCAGATGAGG |
| CDH1.3.1.CDH1.1_F | chr16:68835583-68835742 | TGCCCTGCAGTGAATTTTGA |
| CDH1.3.1.CDH1.1_R | chr16:68835583-68835742 | GTATTCAGCGTGACTTTGGT |
| CDH1.3.2.CDH1.2_F | chr16:68835685-68835824 | AAAAGGCCTCTACGGTTTCA |
| CDH1.3.2.CDH1.2_R | chr16:68835685-68835824 | ACAGCGCACTAAAACAACAG |
| CDH1.3.2.CDH1.3_F | chr16:68835513-68835662 | ACAAGTTCGCTCTTTGGAGA |
| CDH1.3.2.CDH1.3_R | chr16:68835513-68835662 | AAACCGTAGAGGCCTTTTGA |
| CDH1.7.1.CDH1.1_F | chr16:68845604-68845763 | CAGGAACCTCTGTGATGGAG |
| CDH1.7.1.CDH1.1_R | chr16:68845604-68845763 | ATCCTCCTGACCCCTGA |
| CDH1.7.2.CDH1.2_F | chr16:68845505-68845655 | ATGTCCCCTCCTTTATCCCT |
| CDH1.7.2.CDH1.2_R | chr16:68845505-68845655 | TTGGCTGAGGATGGTGTAAG |
| CDH1.9.1.CDH1.1_F | chr16:68847236-68847396 | AAGGGTCAGGTGCCTGA |
| CDH1.9.1.CDH1.1_R | chr16:68847236-68847396 | GCCAGGTACCATACAAACCT |
| CDH1.9.2.CDH1.2_F | chr16:68847266-68847408 | AACGTCGTAATCACCACACT |
| CDH1.9.2.CDH1.2_R | chr16:68847266-68847408 | TTTCTGCATCTTGCCAGGTA |
| CDH1.9.2.CDH1.3_F | chr16:68847097-68847245 | TCAAATACACTCCAGCCTGG |
| CDH1.9.2.CDH1.3_R | chr16:68847097-68847245 | AGTGTGGTGATTACGACGTT |
| CDH1.12.1.CDH1.1_F | chr16:68855944-68856103 | ACAGGGACACTTCTGCTGAT |
| CDH1.12.1.CDH1.1_R | chr16:68855944-68856103 | TCGTTGTACTGAATGGTCCA |
| CDH1.12.2.CDH1.2_F | chr16:68856080-68856223 | CATCTCCCTTCACAGCAGAA |
| CDH1.12.2.CDH1.2_R | chr16:68856080-68856223 | TCAATGGAAGGGGTGACATC |
| CDH1.12.2.CDH1.3_F | chr16:68855844-68855999 | TCTAGACTTGGTCTGGTGGA |
| CDH1.12.2.CDH1.3_R | chr16:68855844-68855999 | AGGCTTTGGATTCCTCTCAC |
| CDH1.2.1.CDH1.1_F | chr16:68772193-68772345 | GTTCCATCTACCTTTCCCCC |
| CDH1.2.1.CDH1.1_R | chr16:68772193-68772345 | TTTCCAACCCCTCCCTACT |
| CDH1.15.1.CDH1.1_F | chr16:68863574-68863730 | AAGGACTTTGACTTGAGCCA |
| CDH1.15.1.CDH1.1_R | chr16:68863574-68863730 | CCTGAGCTTAGAGATGAGCC |
| CDH1.15.2.CDH1.2_F | chr16:68863505-68863664 | CTGAACATAGCCCTGTGTGT |
| CDH1.15.2.CDH1.2_R | chr16:68863505-68863664 | CCAATTTCATCGGGATTGGC |
| CDH1.14.1.CDH1.1_F | chr16:68862064-68862218 | TCAACACTTGCTCTGTCTCC |
| CDH1.14.1.CDH1.1_R | chr16:68862064-68862218 | CCACTGAGCTACCAAGGTTT |
| CDH1.8.1.CDH1.1_F | chr16:68846038-68846184 | TTGTGTCGATCTCTCTGCAG |
| CDH1.8.1.CDH1.1_R | chr16:68846038-68846184 | ACCTTTCTTTGGAAACCCTCT |
| CDH1.4.1.CDH1.1_F | chr16:68842334-68842486 | TCTTTCCTTTTAGGCCTCCG |
| CDH1.4.1.CDH1.1_R | chr16:68842334-68842486 | ATCCCTCCCAGAGAAACAGA |
| CDH1.4.2.CDH1.2_F | chr16:68842229-68842380 | TTCAAACTGTACACTGCCCA |
| CDH1.4.2.CDH1.2_R | chr16:68842229-68842380 | CTCTTCTGTCTTCTGAGGCC |
| CDH1.1.1.CDH1.1_F | chr16:68771230-68771374 | CAAAGCACCTGTGAGCTTG |
| CDH1.1.1.CDH1.1_R | chr16:68771230-68771374 | TCGCAAGTCAGGGGATC |
| CDH1.10.1.CDH1.1_F | chr16:68849438-68849598 | GGCTTGGATTTTGAGGCCAA |
| CDH1.10.1.CDH1.1_R | chr16:68849438-68849598 | GATGTGATTTCCTGGCCCA |
| CDH1.10.2.CDH1.2_F | chr16:68849541-68849678 | TGGATGTGCTGGATGTGAAT |
| CDH1.10.2.CDH1.2_R | chr16:68849541-68849678 | GTTGCTGCAAGTCAGTTGAA |
| CDH1.10.2.CDH1.3_F | chr16:68849308-68849460 | TGGCAGAAACCACAGTTACT |
| CDH1.10.2.CDH1.3_R | chr16:68849308-68849460 | AGGTACCACATTCGTCACTG |
| CDH1.13.1.CDH1.1_F | chr16:68857386-68857544 | TCAAGCTCATGGATAACCAGA |
| CDH1.13.1.CDH1.1_R | chr16:68857386-68857544 | AGTCAAAGGCTGAGTCACTT |
| CDH1.13.2.CDH1.2_F | chr16:68857228-68857387 | GCGGGTGTCTTTAGTTCACT |
| CDH1.13.2.CDH1.2_R | chr16:68857228-68857387 | AAGGTGGTCACTTGGTCTTT |
| CDH1.16.1.CDH1.1_F | chr16:68867272-68867431 | TGACTATGAAGGAAGCGGTT |
| CDH1.16.1.CDH1.1_R | chr16:68867272-68867431 | TTCTGCATTTCCCAGCACAT |
| CDH1.16.2.CDH1.2_F | chr16:68867136-68867281 | ATTGCTAGACTTCTTGCCCC |
| CDH1.16.2.CDH1.2_R | chr16:68867136-68867281 | AGTTCAGGGAGCTCAGACTA |
| CDH1.5.1.CDH1.1_F | chr16:68842624-68842763 | CCAACAAAGACAAAGAAGGCA |
| CDH1.5.1.CDH1.1_R | chr16:68842624-68842763 | CCGGTGTCAACAAGCTTCTA |
| CDH1.5.2.CDH1.2_F | chr16:68842564-68842715 | CCAGTGTTGGGATCCTTCTT |
| CDH1.5.2.CDH1.2_R | chr16:68842564-68842715 | CGTTCTCTATCCAGAGGCTC |
| CDKN2A.1.1.CDKN2A.1_F | chr9:21968220-21968350 | GAGGTTTCTCAGAGCCTCTC |
| CDKN2A.1.1.CDKN2A.1_R | chr9:21968220-21968350 | GAAGCCATTGCGAGAACTTT |
| CDKN2A.4.1.CDKN2A.1_F | chr9:21974496-21974658 | TCATTCCTCTTCCTTGGCTT |
| CDKN2A.4.1.CDKN2A.1_R | chr9:21974496-21974658 | TGGGTAGAGGGTCTGCA |
| CDKN2A.4.2.CDKN2A.2_F | chr9:21974467-21974615 | ATTCGAGAGATCTGTACGCG |
| CDKN2A.4.2.CDKN2A.2_R | chr9:21974467-21974615 | ACTCTGGAGGACGAAGTTTG |
| CDKN2A.4.3.CDKN2A.3_F | chr9:21974636-21974791 | CAAACTTCGTCCTCCAGAGT |
| CDKN2A.4.3.CDKN2A.3_R | chr9:21974636-21974791 | AGCAGCATGGAGCCTTC |
| CDKN2A.4.4.CDKN2A.4_F | chr9:21974759-21974911 | GCACCTCCTCTACCCGA |
| CDKN2A.4.4.CDKN2A.4_R | chr9:21974759-21974911 | GAAGAAAGAGGAGGGGCTG |

| | | |
|---|---|---|
| CDKN2A.3.1.CDKN2A.1_F | chr9:21971091-21971250 | ACCAGCGTGTCCAGGAA |
| CDKN2A.3.1.CDKN2A.1_R | chr9:21971091-21971250 | CAAGCTTCCTTTCCGTCATG |
| CDKN2A.3.4.CDKN2A.2_F | chr9:21970906-21971070 | TCATCAGTCCTCACCTGAG |
| CDKN2A.3.4.CDKN2A.2_R | chr9:21970906-21971070 | TGGACACGCTGGTGGT |
| CDKN2A.3.5.CDKN2A.3_F | chr9:21970763-21970919 | AGGGCGATAGGGAGACTCA |
| CDKN2A.3.5.CDKN2A.3_R | chr9:21970763-21970919 | GTAACCATGCCCGCATAGAT |
| CDKN2A.3.6.CDKN2A.4_F | chr9:21971015-21971160 | CTCCTCAGCCAGGTCCA |
| CDKN2A.3.6.CDKN2A.4_R | chr9:21971015-21971160 | AGCTGCTGCTGCTCCA |
| CDKN2A.2.1.CDKN2A.1_F | chr9:21968680-21968819 | CAGCTTGCGATAACCAAAGG |
| CDKN2A.2.1.CDKN2A.1_R | chr9:21968680-21968819 | ACGCAACGAGATTAGAGAGG |
| CDKN2A.5.1.CDKN2A.1_F | chr9:21994137-21994297 | CTTTTCGAGGGCCTTTCCTA |
| CDKN2A.5.1.CDKN2A.1_R | chr9:21994137-21994297 | TTGGTGACCCTCCGGATT |
| CDKN2A.5.2.CDKN2A.2_F | chr9:21994270-21994411 | GATGTGAACCACGAAAACCC |
| CDKN2A.5.2.CDKN2A.2_R | chr9:21994270-21994411 | GTCCCAGTCTGCAGTTAAGG |
| CYP2D6.6.1.CYP2D6.1_F | chr22:42524813-42524957 | AAGCCCGACTCCTCCTT |
| CYP2D6.6.1.CYP2D6.1_R | chr22:42524813-42524957 | ACCCCTTACCCGCATCT |
| CYP2D6.6.2.CYP2D6.2_F | chr22:42524692-42524851 | AATGTCCTTTCCCAAACCCA |
| CYP2D6.6.2.CYP2D6.2_R | chr22:42524692-42524851 | TTCGAGTACGACGACCCT |
| CYP2D6.3.1.CYP2D6.1_F | chr22:42523458-42523618 | CCAGGCCTACCTTAGGGAT |
| CYP2D6.3.1.CYP2D6.1_R | chr22:42523458-42523618 | CCGTGTCCAACAGGAGA |
| CYP2D6.3.2.CYP2D6.2_F | chr22:42523561-42523717 | AATCACGGCAGTGGTGTAG |
| CYP2D6.3.2.CYP2D6.2_R | chr22:42523561-42523717 | GATCCTGTAAGCCTGACCTC |
| CYP2D6.3.2.CYP2D6.3_F | chr22:42523352-42523482 | GTGGCATTGAGGACTAGGTG |
| CYP2D6.3.2.CYP2D6.3_R | chr22:42523352-42523482 | GTGACCCATATGACATCCCG |
| CYP2D6.7.3.CYP2D6.1_F | chr22:42525026-42525167 | TTTGTGCCCTTCTGCCC |
| CYP2D6.7.3.CYP2D6.1_R | chr22:42525026-42525167 | TGTTCCTGGCGCGCTAT |
| CYP2D6.7.4.CYP2D6.2_F | chr22:42525106-42525247 | TGCTCCAGCGACTTCTTG |
| CYP2D6.7.4.CYP2D6.2_R | chr22:42525106-42525247 | GAGCATAGGGTTGGAGTGG |
| CYP2D6.9.1.CYP2D6.1_F | chr22:42526623-42526784 | TCCCTCACCTGGTCGAA |
| CYP2D6.9.1.CYP2D6.1_R | chr22:42526623-42526784 | AGGCAGGTATGGGGCTA |
| CYP2D6.9.2.CYP2D6.2_F | chr22:42526638-42526793 | TCGAAGCAGTATGGTGTGTT |
| CYP2D6.9.2.CYP2D6.2_R | chr22:42526638-42526793 | CATTTGGTAGTGAGGCAGGT |
| CYP2D6.9.2.CYP2D6.3_F | chr22:42526486-42526627 | AAATGCCCTTCTCCAGGAAG |
| CYP2D6.9.2.CYP2D6.3_R | chr22:42526486-42526627 | GGACTTCCAGAACACACCAT |
| CYP2D6.2.1.CYP2D6.1_F | chr22:42522863-42523023 | ACAGGCACCTGCTGAGAA |
| CYP2D6.2.1.CYP2D6.1_R | chr22:42522863-42523023 | CAGCATCCTAGAGTCCAGTC |
| CYP2D6.2.2.CYP2D6.2_F | chr22:42522815-42522968 | GTGATACCCCTGCAAGACTC |
| CYP2D6.2.2.CYP2D6.2_R | chr22:42522815-42522968 | ACACTCATCACCAACCTGTC |
| CYP2D6.8.1.CYP2D6.1_F | chr22:42525748-42525909 | GCTGCTTGCCTTGGGAA |
| CYP2D6.8.1.CYP2D6.1_R | chr22:42525748-42525909 | ACCCTCCCTCTGCAGTT |
| CYP2D6.8.2.CYP2D6.2_F | chr22:42525863-42526002 | ATTGAGCACGACCACCG |
| CYP2D6.8.2.CYP2D6.2_R | chr22:42525863-42526002 | ACCTGCTCACTCCTGGTAG |
| CYP2D6.8.2.CYP2D6.3_F | chr22:42525724-42525875 | CCACGGAAATCTGTCTCTGT |
| CYP2D6.8.2.CYP2D6.3_R | chr22:42525724-42525875 | ACGTGTTCAGCCTGCAG |
| CYP2D6.4.1.CYP2D6.1_F | chr22:42523838-42523998 | TGCACTGTTTCCCAGATGG |
| CYP2D6.4.1.CYP2D6.1_R | chr22:42523838-42523998 | TTCTGTCCCGAGTATGCTCT |
| CYP2D6.1.5.CYP2D6.1_F | chr22:42522635-42522794 | CACCAGGAAAGCAAAGACAC |
| CYP2D6.1.5.CYP2D6.1_R | chr22:42522635-42522794 | GAGTCTTGCAGGGGTATCAC |
| CYP2D6.1.6.CYP2D6.2_F | chr22:42522516-42522670 | CCGAGTTGGAACTACCACAT |
| CYP2D6.1.6.CYP2D6.2_R | chr22:42522516-42522670 | CTGCAGCACTTCAGCTTCT |
| CYP2D6.5.1.CYP2D6.1_F | chr22:42524186-42524346 | CCACTCTCACCTTCTCCATC |
| CYP2D6.5.1.CYP2D6.1_R | chr22:42524186-42524346 | CTGTCTGGTGTAGGTGCTG |
| CYP2D6.5.2.CYP2D6.2_F | chr22:42524256-42524395 | GGTCATCCTGTGCTCAGTTA |
| CYP2D6.5.2.CYP2D6.2_R | chr22:42524256-42524395 | TGAGACTTGTCCAGGTGAAC |
| CYP2D6.5.2.CYP2D6.3_F | chr22:42524102-42524247 | ATGCACTGGTCCAACCTTTT |
| CYP2D6.5.2.CYP2D6.3_R | chr22:42524102-42524247 | TGGATGAGCTGCTAACTGAG |
| KRAS.1.6.KRAS.1_F | chr12:25362706-25362835 | ACCACTTGTACTAGTATGCCT |
| KRAS.1.6.KRAS.1_R | chr12:25362706-25362835 | TTTATGTATTTCAGGGTGTTGATG |
| KRAS.1.7.KRAS.2_F | chr12:25362761-25362887 | ACACACTTTGTCTTTGACTTCT |
| KRAS.1.7.KRAS.2_R | chr12:25362761-25362887 | TGTTACCTGTACACATGAAGC |
| KRAS.4.1.KRAS.1_F | chr12:25380235-25380366 | TACACAAAGAAAGCCCTCCC |
| KRAS.4.1.KRAS.1_R | chr12:25380235-25380366 | AGGTGCACTGTAATAATCCAGA |
| KRAS.4.4.KRAS.2_F | chr12:25380106-25380250 | TGCATGGCATTAGCAAAGAC |
| KRAS.4.4.KRAS.2_R | chr12:25380106-25380250 | AGTACAGTGCAATGAGGGAC |
| KRAS.3.1.KRAS.1_F | chr12:25378606-25378763 | TCCATAACTTCTTGCTAAGTCC |
| KRAS.3.1.KRAS.1_R | chr12:25378606-25378763 | GACAAAAGTTGTGGACAGGT |
| KRAS.3.5.KRAS.2_F | chr12:25378526-25378660 | TGATTTTGCAGAAAACAGATCTG |

| | | |
|---|---|---|
| KRAS.3.5.KRAS.2_R | chr12:25378526-25378660 | TCTGAAGATGTACCTATGGTCC |
| KRAS.2.1.KRAS.1_F | chr12:25368374-25368522 | GTGCTGAACTTAAACTTACCAG |
| KRAS.2.1.KRAS.1_R | chr12:25368374-25368522 | ACTCTTACCAATGCAACAGAC |
| KRAS.5.2.KRAS.1_F | chr12:25398179-25398312 | TCAAAGAATGGTCCTGCACC |
| KRAS.5.2.KRAS.1_R | chr12:25398179-25398312 | AAGGCCTGCTGAAAATGACT |
| KRAS.5.3.KRAS.2_F | chr12:25398269-25398395 | ATTAGCTGTATCGTCAAGGCA |
| KRAS.5.3.KRAS.2_R | chr12:25398269-25398395 | AAAGGTACTGGTGGAGTATTTG |
| CTNNB1.6.1.CTNNB1.1_F | chr3:41268718-41268865 | AGCTCATCATACTGGCTAGTG |
| CTNNB1.6.1.CTNNB1.1_R | chr3:41268718-41268865 | TGCAGATGCTATACACAAGACT |
| CTNNB1.6.2.CTNNB1.2_F | chr3:41268654-41268809 | TGGCTCTTCTCAGACATGTG |
| CTNNB1.6.2.CTNNB1.2_R | chr3:41268654-41268809 | GCCGGCTTATTACTAGAGCA |
| CTNNB1.11.1.CTNNB1.1_F | chr3:41277844-41278000 | TGTGTTTTCTCCTTAGCTGC |
| CTNNB1.11.1.CTNNB1.1_R | chr3:41277844-41278000 | TGCTAAAGGCTTTGGTTCCT |
| CTNNB1.11.2.CTNNB1.2_F | chr3:41277760-41277890 | GCCTCTTGCACTCTGAATTG |
| CTNNB1.11.2.CTNNB1.2_R | chr3:41277760-41277890 | TTGTCCTGAGCAAGTTCACA |
| CTNNB1.3.1.CTNNB1.1_F | chr3:41266483-41266644 | CAATGACTCGAGCTCAGAGG |
| CTNNB1.3.1.CTNNB1.1_R | chr3:41266483-41266644 | GATTGCACGTGTGGCAAG |
| CTNNB1.3.2.CTNNB1.2_F | chr3:41266609-41266742 | CACAGATGCTGAAACATGCA |
| CTNNB1.3.2.CTNNB1.2_R | chr3:41266609-41266742 | GAAACTACTCCCCTTGAGCA |
| CTNNB1.3.2.CTNNB1.3_F | chr3:41266423-41266558 | TGCTGAACTGTGGATAGTGA |
| CTNNB1.3.2.CTNNB1.3_R | chr3:41266423-41266558 | CAAACGCTGGACATTAGTGG |
| CTNNB1.7.1.CTNNB1.1_F | chr3:41274810-41274947 | TCTAGCTAATGACTAGGGCCT |
| CTNNB1.7.1.CTNNB1.1_R | chr3:41274810-41274947 | ATTCCCATGGCACCAGTTTA |
| CTNNB1.9.1.CTNNB1.1_F | chr3:41275640-41275798 | CTGTTTTTCAGGCTACTGTTG |
| CTNNB1.9.1.CTNNB1.1_R | chr3:41275640-41275798 | AGCCAGGTATCACTGTAAGA |
| CTNNB1.9.2.CTNNB1.2_F | chr3:41275495-41275647 | GTAGGAAGTATGGCTGCGAT |
| CTNNB1.9.2.CTNNB1.2_R | chr3:41275495-41275647 | GGACAAAGGGCAAGATTTCG |
| CTNNB1.12.1.CTNNB1.1_F | chr3:41278074-41278233 | GGGCCTTTTTCTCCTTGTCT |
| CTNNB1.12.1.CTNNB1.1_R | chr3:41278074-41278233 | GCCATACCTGCTCTAGGAAA |
| CTNNB1.2.1.CTNNB1.1_F | chr3:41266047-41266206 | AGTTGGACATGGCCATGG |
| CTNNB1.2.1.CTNNB1.1_R | chr3:41266047-41266206 | GTGAAGGACTGAGAAAATCCC |
| CTNNB1.2.2.CTNNB1.2_F | chr3:41266126-41266265 | ATCCATTCTGGTGCCACTAC |
| CTNNB1.2.2.CTNNB1.2_R | chr3:41266126-41266265 | GCATTCTGACTTTCAGTAAGGC |
| CTNNB1.2.2.CTNNB1.3_F | chr3:41265901-41266051 | TCTGCTTTTCTTGGCTGTCT |
| CTNNB1.2.2.CTNNB1.3_R | chr3:41265901-41266051 | CTAACAGCCGCTTTTCTGTC |
| CTNNB1.14.1.CTNNB1.1_F | chr3:41280625-41280785 | TGACACCCTGACTCTTCTAG |
| CTNNB1.14.1.CTNNB1.1_R | chr3:41280625-41280785 | GATTGCTGTCACCTGGAGG |
| CTNNB1.14.2.CTNNB1.2_F | chr3:41280747-41280906 | ACTATCCAGTTGATGGGCTG |
| CTNNB1.14.2.CTNNB1.2_R | chr3:41280747-41280906 | ACCCTACCAACCAAGTCTTT |
| CTNNB1.8.1.CTNNB1.1_F | chr3:41275040-41275199 | GAAGGGATGGAAGGTCTCCT |
| CTNNB1.8.1.CTNNB1.1_R | chr3:41275040-41275199 | TCAGTGATGTCTTCCCTGTC |
| CTNNB1.8.2.CTNNB1.2_F | chr3:41274975-41275133 | GTGCCATGGGAATAGAGTCA |
| CTNNB1.8.2.CTNNB1.2_R | chr3:41274975-41275133 | TGGCAGACCATCATCTTGTT |
| CTNNB1.8.2.CTNNB1.3_F | chr3:41275240-41275397 | CCTGCCATCTGTGCTCTTC |
| CTNNB1.8.2.CTNNB1.3_R | chr3:41275240-41275397 | CAGCTAGAGATGCTTCATTTTC |
| CTNNB1.8.3.CTNNB1.4_F | chr3:41275176-41275319 | TGGGTGGTATAGAGGCTCTT |
| CTNNB1.8.3.CTNNB1.4_R | chr3:41275176-41275319 | GATGGTGGGTGTAAGAGCTT |
| CTNNB1.1.1.CTNNB1.1_F | chr3:41265557-41265709 | GTTTTGAAAATCCAGCGTGG |
| CTNNB1.1.1.CTNNB1.1_R | chr3:41265557-41265709 | TTCACCAAGCCATTAGGAGG |
| CTNNB1.4.1.CTNNB1.1_F | chr3:41266913-41267071 | CATGCGTTCTCCTCAGATGG |
| CTNNB1.4.1.CTNNB1.1_R | chr3:41266913-41267071 | GCTTCAAGCATTCTGACATGT |
| CTNNB1.4.2.CTNNB1.2_F | chr3:41266764-41266916 | GCTCAAGGGGAGTAGTTTCA |
| CTNNB1.4.2.CTNNB1.2_R | chr3:41266764-41266916 | TGCATGGTACGTACAATAGCA |
| CTNNB1.10.4.CTNNB1.1_F | chr3:41277190-41277319 | TACAGAGGAGAATGCCCTGT |
| CTNNB1.10.4.CTNNB1.1_R | chr3:41277190-41277319 | ACATACCTGCACAAACAATGG |
| CTNNB1.10.5.CTNNB1.2_F | chr3:41277258-41277415 | AGTTGAAGGTTGTACCGGAG |
| CTNNB1.10.5.CTNNB1.2_R | chr3:41277258-41277415 | TGGTAACCCTATTATGGTCCC |
| CTNNB1.13.1.CTNNB1.1_F | chr3:41279495-41279638 | TCTATTCTTCCTTGCTTTGTGC |
| CTNNB1.13.1.CTNNB1.1_R | chr3:41279495-41279638 | TGGTTTCCCAGATGAGCAAA |
| CTNNB1.5.1.CTNNB1.1_F | chr3:41267171-41267330 | TTCACCAGTGGATTCTGTGT |
| CTNNB1.5.1.CTNNB1.1_R | chr3:41267171-41267330 | TGCTTTCTTGGTTGCCATAA |
| CTNNB1.5.2.CTNNB1.2_F | chr3:41267276-41267426 | GAAAATGGTTGCCTTGCTCA |
| CTNNB1.5.2.CTNNB1.2_R | chr3:41267276-41267426 | TGGTGCCAAAAAGGTTAGGA |
| CTNNB1.5.2.CTNNB1.3_F | chr3:41267045-41267177 | TAAGTCTGGAGGCATTCCTG |
| CTNNB1.5.2.CTNNB1.3_R | chr3:41267045-41267177 | TGTGGAGAGTTGTAATGGCA |
| ABL2.6.1.ABL2.1_F | chr1:179086469-179086629 | AGTCCCTTTCAACCTTACCC |
| ABL2.6.1.ABL2.1_R | chr1:179086469-179086629 | TCTTGCAGCTCGTAACTGC |

| | | |
|---|---|---|
| ABL2.6.2.ABL2.2_F | chr1:179086530-179086688 | CTCTGGTGCTGTCCACTTAA |
| ABL2.6.2.ABL2.2_R | chr1:179086530-179086688 | TCATGACCATTTTCCTCACCA |
| ABL2.6.2.ABL2.3_F | chr1:179086376-179086509 | TGGCAGGTAAAGGTAGAGGA |
| ABL2.6.2.ABL2.3_R | chr1:179086376-179086509 | TTAAGTGGACAGCACCAGAG |
| ABL2.11.1.ABL2.1_F | chr1:179100465-179100620 | ACCTTTAGTGATGCTGAGTGT |
| ABL2.11.1.ABL2.1_R | chr1:179100465-179100620 | TCCCTTCCCTTCTTATGTCTT |
| ABL2.11.2.ABL2.2_F | chr1:179100445-179100592 | GGATGCTACATAGAAGCCTCA |
| ABL2.11.2.ABL2.2_R | chr1:179100445-179100592 | TTTGCATCGTCCCTATGGTT |
| ABL2.3.1.ABL2.1_F | chr1:179079837-179079982 | GCATGCCAACGTGTGAATAT |
| ABL2.3.1.ABL2.1_R | chr1:179079837-179079982 | CGTATACCCTGTGATCCACC |
| ABL2.7.1.ABL2.1_F | chr1:179087780-179087939 | AGAAGAAATCTGAGTGGCCA |
| ABL2.7.1.ABL2.1_R | chr1:179087780-179087939 | AGTGGTTTGGAGTGTTTGAA |
| ABL2.7.2.ABL2.2_F | chr1:179087689-179087821 | CTGCATGTCAAGCAAGATGG |
| ABL2.7.2.ABL2.2_R | chr1:179087689-179087821 | TTTGCTGGATTACCTCCGAG |
| ABL2.9.1.ABL2.1_F | chr1:179090730-179090889 | TGTAATTCTCAGCAACCCAC |
| ABL2.9.1.ABL2.1_R | chr1:179090730-179090889 | TACCCAGCACCCAAGTGTAA |
| ABL2.9.2.ABL2.2_F | chr1:179090860-179091002 | CCATTTCCCATTTGTCGTGG |
| ABL2.9.2.ABL2.2_R | chr1:179090860-179091002 | GCTTTTCTCCTTTTCTTACCAG |
| ABL2.12.1.ABL2.1_F | chr1:179102420-179102577 | ACTGCCAATGCCTTAGTTCA |
| ABL2.12.1.ABL2.1_R | chr1:179102420-179102577 | TGAATGCCATGAAAAGGAGAC |
| ABL2.2.1.ABL2.1_F | chr1:179079416-179079576 | ACCTGCTAAGGGACCCATA |
| ABL2.2.1.ABL2.1_R | chr1:179079416-179079576 | TCACAGAGGTAGCTGAGGAG |
| ABL2.2.2.ABL2.2_F | chr1:179079506-179079660 | CCTGTTTCTTCAGTGTCCGA |
| ABL2.2.2.ABL2.2_R | chr1:179079506-179079660 | TCTTTCTCAGTTGTGTGCCT |
| ABL2.14.1.ABL2.1_F | chr1:179198403-179198564 | GGTGAAGATATTGAAGCCGG |
| ABL2.14.1.ABL2.1_R | chr1:179198403-179198564 | AGGAATGTGACCAGGGGT |
| ABL2.14.12.ABL2.2_F | chr1:179198376-179198517 | GCTCCCAGACACACTCAC |
| ABL2.14.12.ABL2.2_R | chr1:179198376-179198517 | GATGGGGCAGCAGGTG |
| ABL2.8.1.ABL2.1_F | chr1:179089298-179089436 | GGCAAGCATTTTTGGAGGAA |
| ABL2.8.1.ABL2.1_R | chr1:179089298-179089436 | GGTTTGTCCCTCTCGTTTTC |
| ABL2.4.1.ABL2.1_F | chr1:179081412-179081568 | CACCACCACTACCCAGAATT |
| ABL2.4.1.ABL2.1_R | chr1:179081412-179081568 | CCACTGGATATTGAATTTCTGC |
| ABL2.1.1.ABL2.1_F | chr1:179076851-179077012 | TTCCCACCAGGCTAACAGT |
| ABL2.1.1.ABL2.1_R | chr1:179076851-179077012 | TGGACTGCATCCCTCAAAC |
| ABL2.1.2.ABL2.2_F | chr1:179077010-179077172 | CTCGGAAGGCAAATTTGTTG |
| ABL2.1.2.ABL2.2_R | chr1:179077010-179077172 | AACCAAACAGGCCGCTG |
| ABL2.1.3.ABL2.3_F | chr1:179077182-179077342 | GAGATTTTCTCAGCGGCCT |
| ABL2.1.3.ABL2.3_R | chr1:179077182-179077342 | TCCACATCAGAAACACAGGA |
| ABL2.1.4.ABL2.4_F | chr1:179077343-179077502 | AGCTGCCTTCTTTCCTCCT |
| ABL2.1.4.ABL2.4_R | chr1:179077343-179077502 | TCTTATCTGAGCATCAGGTCA |
| ABL2.1.4.ABL2.5_F | chr1:179077049-179077197 | CCACATAGCCTGAGCAGTAG |
| ABL2.1.4.ABL2.5_R | chr1:179077049-179077197 | CAGCAGGTACTAAAGTGGCT |
| ABL2.1.5.ABL2.6_F | chr1:179077553-179077712 | ATTCCCCTGAGAGTCTGTG |
| ABL2.1.5.ABL2.6_R | chr1:179077553-179077712 | GTAAAGAGAAGAATGGTGGGG |
| ABL2.1.6.ABL2.7_F | chr1:179077713-179077875 | AGCCATCCCAAGTCGTG |
| ABL2.1.6.ABL2.7_R | chr1:179077713-179077875 | AATCAGAGGAAAGTGCTGCT |
| ABL2.1.6.ABL2.8_F | chr1:179077477-179077608 | CACTTTGGTTTTACCCGTCG |
| ABL2.1.6.ABL2.8_R | chr1:179077477-179077608 | CCACTCACAACCACAAAGTG |
| ABL2.1.7.ABL2.9_F | chr1:179077886-179078046 | TGCTTGGAGCAGCACTT |
| ABL2.1.7.ABL2.9_R | chr1:179077886-179078046 | TCCAAGGTCAAACTCTACATCT |
| ABL2.1.8.ABL2.10_F | chr1:179078047-179078206 | AAGCCCTGAGGACATGGA |
| ABL2.1.8.ABL2.10_R | chr1:179078047-179078206 | AACCTCTGTAATGACGACGG |
| ABL2.1.8.ABL2.11_F | chr1:179077854-179078005 | TCCTCTGGGCAATAACTTGG |
| ABL2.1.8.ABL2.11_R | chr1:179077854-179078005 | GAGCAGGATAGGATGGCAAT |
| ABL2.1.9.ABL2.12_F | chr1:179078227-179078386 | CCGTCGTCATTACAGAGGTT |
| ABL2.1.9.ABL2.12_R | chr1:179078227-179078386 | AAACGCAGCAGCTCCTT |
| ABL2.1.10.ABL2.13_F | chr1:179078387-179078549 | GCTGATTCTCCATTTCTCGG |
| ABL2.1.10.ABL2.13_R | chr1:179078387-179078549 | AGGTGCACAGGCCTCTA |
| ABL2.1.10.ABL2.14_F | chr1:179078165-179078307 | TAAAGAAGCCTGTGATGCCA |
| ABL2.1.10.ABL2.14_R | chr1:179078165-179078307 | TTGCTTCTCTACAGCATGCT |
| ABL2.1.11.ABL2.15_F | chr1:179078492-179078637 | AGCATGTCTCTTTGGCATCT |
| ABL2.1.11.ABL2.15_R | chr1:179078492-179078637 | CTCATAATGCAGCCTCGGTA |
| ABL2.10.1.ABL2.1_F | chr1:179095512-179095671 | TGGTGACCAGGGAGTCTTAC |
| ABL2.10.1.ABL2.1_R | chr1:179095512-179095671 | AAACACTCCTGGTACCATGG |
| ABL2.10.2.ABL2.2_F | chr1:179095661-179095810 | AGATACTCAGCTGCACTGC |
| ABL2.10.2.ABL2.2_R | chr1:179095661-179095810 | TCCTGTGATGACCTTATGGC |
| ABL2.13.1.ABL2.1_F | chr1:179112043-179112185 | ATGTGGTTATCAAGCCTCCC |

| | | |
|---|---|---|
| ABL2.13.1.ABL2.1_R | chr1:179112043-179112185 | TAACTTTTGTGCTTCTGGGC |
| ABL2.5.1.ABL2.1_F | chr1:179084030-179084186 | CTCACATGCTCTCATAAGTTCA |
| ABL2.5.1.ABL2.1_R | chr1:179084030-179084186 | CGGAGAAGAAACACTAAGTGC |
| ABL2.5.2.ABL2.2_F | chr1:179083917-179084049 | TGTCATGAGGCTGGATTCAA |
| ABL2.5.2.ABL2.2_R | chr1:179083917-179084049 | ATCGAATGGAACAGCCTGAG |
| BRAF.11.3.BRAF.1_F | chr7:140494105-140494258 | AGTTCTAGCAATGCTGGATACT |
| BRAF.11.3.BRAF.1_R | chr7:140494105-140494258 | CATTTTTCCAGGCCCCAAAT |
| BRAF.11.11.BRAF.2_F | chr7:140494231-140494381 | AAGGGCTGTGGAATTGGAAT |
| BRAF.11.11.BRAF.2_R | chr7:140494231-140494381 | GTCATTACTGCTAGGTTTTTAATGC |
| BRAF.7.5.BRAF.1_F | chr7:140477734-140477881 | TAGTTGCTACCACTGGGAAC |
| BRAF.7.5.BRAF.1_R | chr7:140477734-140477881 | ACACTTGGAGTAACAATTGCC |
| BRAF.17.13.BRAF.1_F | chr7:140549836-140550087 | TCCCACCTCCTAAAATAATCAAGA |
| BRAF.17.13.BRAF.1_R | chr7:140549836-140550087 | ACACTGGCAGTTACTGTGATGT |
| BRAF.2.3.BRAF.1_F | chr7:140439570-140439722 | GTGCTCAGAAATCTGTCTATGA |
| BRAF.2.3.BRAF.1_R | chr7:140439570-140439722 | TTTTTATGGTGGGACGAGGA |
| BRAF.2.6.BRAF.2_F | chr7:140439671-140439803 | GCACTCTGCCATTAATCTCT |
| BRAF.2.6.BRAF.2_R | chr7:140439671-140439803 | TGTGTTTTCAATGAGTGTGAAG |
| BRAF.1.3.BRAF.1_F | chr7:140434391-140434550 | CTCCTGAACTCTCTCACTCA |
| BRAF.1.3.BRAF.1_R | chr7:140434391-140434550 | ATTCTCGCCTCTATTGAGCT |
| BRAF.1.4.BRAF.2_F | chr7:140434533-140434683 | ATGCACTGCGGTGAATTTTT |
| BRAF.1.4.BRAF.2_R | chr7:140434533-140434683 | TGTAAAGTGTGATGGGACTCT |
| BRAF.18.9.BRAF.1_F | chr7:140624386-140624552 | TCCGGAATGGCAGGGT |
| BRAF.18.9.BRAF.1_R | chr7:140624386-140624552 | TCTCCGCCTCCCTTCC |
| BRAF.18.10.BRAF.2_F | chr7:140624307-140624444 | GCCGCCTCTTTCCAAAATAA |
| BRAF.18.10.BRAF.2_R | chr7:140624307-140624444 | GGCCAGGCTCTGTTCAA |
| BRAF.13.11.BRAF.1_F | chr7:140501251-140501404 | ACACACATCAGTGGAACTTCT |
| BRAF.13.11.BRAF.1_R | chr7:140501251-140501404 | GTAGATACGTCAGTTTCTAGAAAGT |
| BRAF.13.12.BRAF.2_F | chr7:140501131-140501279 | TGGAAGAAAAACCCTCACAGA |
| BRAF.13.12.BRAF.2_R | chr7:140501131-140501279 | GTTTCCGCTGTCAAACATGT |
| BRAF.16.1.BRAF.1_F | chr7:140534429-140534588 | CACTGTCCTCTGTTTGTTGG |
| BRAF.16.1.BRAF.1_R | chr7:140534429-140534588 | TCTGGGGAACGGAACTGAT |
| BRAF.16.2.BRAF.2_F | chr7:140534535-140534694 | GAAGGTAGCACTGAAAGGCT |
| BRAF.16.2.BRAF.2_R | chr7:140534535-140534694 | GGACAAAGTCCGGATTGAAT |
| BRAF.16.2.BRAF.3_F | chr7:140534299-140534448 | TCCTGTATGACATGGATGCC |
| BRAF.16.2.BRAF.3_R | chr7:140534299-140534448 | CCCAAGTCACCACAAAAACC |
| BRAF.6.7.BRAF.1_F | chr7:140476678-140476830 | ACATCCTCAATGGTCTTCAAAA |
| BRAF.6.7.BRAF.1_R | chr7:140476678-140476830 | ATTCCACAAAGCCACAACTG |
| BRAF.6.8.BRAF.2_F | chr7:140476806-140476953 | ATGGTGATACAAGCTGGAGC |
| BRAF.6.8.BRAF.2_R | chr7:140476806-140476953 | ACCGACAGACTACTTTGGTT |
| BRAF.3.3.BRAF.1_F | chr7:140449054-140449209 | TGGAGCCTTGTATATAGACGG |
| BRAF.3.3.BRAF.1_R | chr7:140449054-140449209 | TGTTCTAACAGGCACCAGAA |
| BRAF.3.4.BRAF.2_F | chr7:140449136-140449289 | GGTAACTGTCCAGTCATCAAT |
| BRAF.3.4.BRAF.2_R | chr7:140449136-140449289 | TGGTAAAAGCATTGCTCTAGGA |
| BRAF.9.1.BRAF.1_F | chr7:140482840-140482983 | ACCATTCGATTCCTGTCTTCT |
| BRAF.9.1.BRAF.1_R | chr7:140482840-140482983 | CATACTTGCTCCTCCTTAATGT |
| BRAF.9.2.BRAF.2_F | chr7:140482784-140482933 | TGGCTGTGACTTCTAAGAAGA |
| BRAF.9.2.BRAF.2_R | chr7:140482784-140482933 | AACCACAGGTTTGTCTGCTA |
| BRAF.12.1.BRAF.1_F | chr7:140500158-140500313 | CCAAGAGCAGAAGTCAAACC |
| BRAF.12.1.BRAF.1_R | chr7:140500158-140500313 | ATGAAGCTTCTGGGTTTTGC |
| BRAF.14.13.BRAF.1_F | chr7:140507692-140507947 | ACTCATCCATATTTCACATTCCCT |
| BRAF.14.13.BRAF.1_R | chr7:140507692-140507947 | CACTGTTACTAGCCCCTCGA |
| BRAF.15.1.BRAF.1_F | chr7:140508689-140508821 | TTCACGTCACATACAAACCA |
| BRAF.15.1.BRAF.1_R | chr7:140508689-140508821 | TGTAATTTGCTCCCTTTACCTC |
| BRAF.8.1.BRAF.1_F | chr7:140481364-140481514 | CTTGTCACAATGTCACCACA |
| BRAF.8.1.BRAF.1_R | chr7:140481364-140481514 | TCTGTTTGGCTTGACTTGAC |
| BRAF.4.1.BRAF.1_F | chr7:140453081-140453228 | AGCCTCAATTCTTACCATCCA |
| BRAF.4.1.BRAF.1_R | chr7:140453081-140453228 | TGCTTGCTCTGATAGGAAAATG |
| BRAF.4.2.BRAF.2_F | chr7:140453034-140453165 | TAACTCAGCAGCATCTCAGG |
| BRAF.4.2.BRAF.2_R | chr7:140453034-140453165 | TCTTCATGAAGACCTCACAGT |
| BRAF.10.1.BRAF.1_F | chr7:140487301-140487429 | AGGAAATAAGCAGCAAAGCA |
| BRAF.10.1.BRAF.1_R | chr7:140487301-140487429 | CAGTGTTATCGCTACTCTCTGA |
| BRAF.5.1.BRAF.1_F | chr7:140453965-140454093 | CAGGATGTTTTCAAACTTCGC |
| BRAF.5.1.BRAF.1_R | chr7:140453965-140454093 | CACCCCCAAGTATGTTCTGT |
| EGFR.11.1.EGFR.1_F | chr7:55225328-55225466 | AGAAACTCCTACGTGGTGTG |
| EGFR.11.1.EGFR.1_R | chr7:55225328-55225466 | GGCAAATGTAATCTACCAGGC |
| EGFR.21.1.EGFR.1_F | chr7:55242382-55242516 | GTGGCACCATCTCACAATTG |
| EGFR.21.1.EGFR.1_R | chr7:55242382-55242516 | CACACAGCAAAGCAGAAACT |

| | | |
|---|---|---|
| EGFR.7.1.EGFR.1_F | chr7:55221703-55221855 | CGTGTCCTCTCTCCTCCATA |
| EGFR.7.1.EGFR.1_R | chr7:55221703-55221855 | AGTTAGAGGGCCCACAGAG |
| EGFR.26.1.EGFR.1_F | chr7:55267952-55268110 | GCCTTCTTTAAGCAATGCCA |
| EGFR.26.1.EGFR.1_R | chr7:55267952-55268110 | GGAAGCACAGACTGCAATTT |
| EGFR.17.1.EGFR.1_F | chr7:55238010-55238172 | CTTTCTCCAGGCCAGGAAAT |
| EGFR.17.1.EGFR.1_R | chr7:55238010-55238172 | ATGGAGGTGGCTGCATC |
| EGFR.17.2.EGFR.2_F | chr7:55238168-55238323 | TCAGAGTTTCAGCTGGGTTG |
| EGFR.17.2.EGFR.2_R | chr7:55238168-55238323 | AGTGGAAGGAAGGAAATGGG |
| EGFR.17.2.EGFR.3_F | chr7:55237975-55238104 | CCAGGCCTCTCACATATTGA |
| EGFR.17.2.EGFR.3_R | chr7:55237975-55238104 | CTAAGCATGACTCCTGAGCA |
| EGFR.2.1.EGFR.1_F | chr7:55209989-55210133 | TTTCTTCCAGTTTGCCAAGG |
| EGFR.2.1.EGFR.1_R | chr7:55209989-55210133 | GTGTAGGAAAATCAAAGTCACC |
| EGFR.2.2.EGFR.2_F | chr7:55209879-55210038 | TCTCTGTGTGGAGAGAGTGA |
| EGFR.2.2.EGFR.2_R | chr7:55209879-55210038 | ATTGAACATCCTCTGGAGGC |
| EGFR.22.1.EGFR.1_F | chr7:55249005-55249165 | AAGCCTACGTGATGGCCA |
| EGFR.22.1.EGFR.1_R | chr7:55249005-55249165 | CCTTCCCTGATTACCTTTGC |
| EGFR.22.2.EGFR.2_F | chr7:55249120-55249254 | GACTATGTCCGGGAACACAA |
| EGFR.22.2.EGFR.2_R | chr7:55249120-55249254 | ACACATATCCCCATGGCAAA |
| EGFR.22.2.EGFR.3_F | chr7:55248915-55249064 | CATTCATGCGTCTTCACCTG |
| EGFR.22.2.EGFR.3_R | chr7:55248915-55249064 | GCATGAGCTGCGTGATGA |
| EGFR.1.4.EGFR.1_F | chr7:55086948-55087164 | GTATTGATCGGGAGAGCCGG |
| EGFR.1.4.EGFR.1_R | chr7:55086948-55087164 | AGGAAACAGGAAAGGACGGG |
| EGFR.18.1.EGFR.1_F | chr7:55238748-55238906 | CCAACATCCAGACACATAGTG |
| EGFR.18.1.EGFR.1_R | chr7:55238748-55238906 | ACAGAGCTGTGAACACTTAC |
| EGFR.30.1.EGFR.1_F | chr7:55272999-55273160 | GCTGGCTCTGTGCAGAAT |
| EGFR.30.1.EGFR.1_R | chr7:55272999-55273160 | CCAGGCTAATTTGGTGGCT |
| EGFR.30.2.EGFR.2_F | chr7:55272855-55273006 | GCTCCCTGTCATAAGTCTCC |
| EGFR.30.2.EGFR.2_R | chr7:55272855-55273006 | GGTTCAGAGGCTGATTGTGA |
| EGFR.30.2.EGFR.3_F | chr7:55273171-55273310 | AGAAAGGCAGCCACCAAA |
| EGFR.30.2.EGFR.3_R | chr7:55273171-55273310 | GCTCATACTATCCTCCGTGG |
| EGFR.30.3.EGFR.4_F | chr7:55273102-55273243 | CCCCGAGTATCTCAACACTG |
| EGFR.30.3.EGFR.4_R | chr7:55273102-55273243 | CTGCATTTTCAGCTGTGGAG |
| EGFR.23.1.EGFR.1_F | chr7:55259410-55259567 | AGCAGGGTCTTCTCTGTTTC |
| EGFR.23.1.EGFR.1_R | chr7:55259410-55259567 | ACCTAAAGCCACCTCCTTAC |
| EGFR.13.1.EGFR.1_F | chr7:55229187-55229345 | CTGTGACCCACTCTGTCTC |
| EGFR.13.1.EGFR.1_R | chr7:55229187-55229345 | TTTTTGATCAACGCAAGGGG |
| EGFR.16.1.EGFR.1_F | chr7:55236086-55236244 | AGGTCCAGAAAGTGATTGGG |
| EGFR.16.1.EGFR.1_R | chr7:55236086-55236244 | TGCAGTTTTAGTGGGAGACT |
| EGFR.29.1.EGFR.1_F | chr7:55270210-55270373 | CCCTCATTTCCTCCTGCAG |
| EGFR.29.1.EGFR.1_R | chr7:55270210-55270373 | ACTGGAGACTGGCTGCT |
| EGFR.29.2.EGFR.2_F | chr7:55270310-55270459 | CATAGACGACACCTTCCTCC |
| EGFR.29.2.EGFR.2_R | chr7:55270310-55270459 | TTTATCTGCTCCTTACGCCC |
| EGFR.27.1.EGFR.1_F | chr7:55268891-55269051 | GCACCAGGGGGATGAAA |
| EGFR.27.1.EGFR.1_R | chr7:55268891-55269051 | AGAGAGAGAGACAGAGATTTCA |
| EGFR.27.2.EGFR.2_F | chr7:55268790-55268930 | AAATTGGCAAACACACAGGC |
| EGFR.27.2.EGFR.2_R | chr7:55268790-55268930 | CTTCTTCATCCATCAGGGCA |
| EGFR.6.1.EGFR.1_F | chr7:55220200-55220358 | CCTGGGAAATGATCCTACCC |
| EGFR.6.1.EGFR.1_R | chr7:55220200-55220358 | TGCTGGAGGGGCATCTTA |
| EGFR.25.1.EGFR.1_F | chr7:55266396-55266557 | CCCACTGCCTTCTTTTCTTG |
| EGFR.25.1.EGFR.1_R | chr7:55266396-55266557 | AGACCCACCAGTCACTCA |
| EGFR.28.1.EGFR.1_F | chr7:55269421-55269579 | TGGGCAACTTCTCTGTTTCT |
| EGFR.28.1.EGFR.1_R | chr7:55269421-55269579 | ACCCACACAGGAAGAAAACT |
| EGFR.3.1.EGFR.1_F | chr7:55211038-55211198 | TTATGTCCTCATTGCCCTCA |
| EGFR.3.1.EGFR.1_R | chr7:55211038-55211198 | TTATGAACCCCCAGCCTTG |
| EGFR.3.2.EGFR.2_F | chr7:55210906-55211053 | CGTGTGCATTAGGGTTCAAC |
| EGFR.3.2.EGFR.2_R | chr7:55210906-55211053 | TCTGCAGGTTTTCCAAAGGA |
| EGFR.9.1.EGFR.1_F | chr7:55224211-55224356 | TGTGAACGGAATACACGTCT |
| EGFR.9.1.EGFR.1_R | chr7:55224211-55224356 | CAAGCAACTGAACCTGTGAC |
| EGFR.12.1.EGFR.1_F | chr7:55227872-55228031 | TCGTCAGCCTGAACATAACA |
| EGFR.12.1.EGFR.1_R | chr7:55227872-55228031 | AACAGAAAGCGGTGACTTAC |
| EGFR.12.2.EGFR.2_F | chr7:55227723-55227877 | CACAGCATGACCTACCATCA |
| EGFR.12.2.EGFR.2_R | chr7:55227723-55227877 | CCTTGAGGGAGCGTAATCC |
| EGFR.15.1.EGFR.1_F | chr7:55232972-55233130 | TTTTTCTCCACCTTGGTGCA |
| EGFR.15.1.EGFR.1_R | chr7:55232972-55233130 | TCTCCTTCACTTTCCACTCAC |
| EGFR.20.1.EGFR.1_F | chr7:55241601-55241736 | TGACCCTTGTCTCTGTGTTC |
| EGFR.20.1.EGFR.1_R | chr7:55241601-55241736 | TGTGCCAGGGACCTTAC |
| EGFR.14.1.EGFR.1_F | chr7:55231378-55231531 | GAGGTGATTTGTGTTCCTGC |

| | | |
|---|---|---|
| EGFR.14.1.EGFR.1_R | chr7:55231378-55231531 | TGAAATGGACGTGGATAGCA |
| EGFR.8.1.EGFR.1_F | chr7:55223523-55223668 | TTTCATGCTCTCTTCCCCAG |
| EGFR.8.1.EGFR.1_R | chr7:55223523-55223668 | TCAGCAGCCGAGAACAAG |
| EGFR.4.1.EGFR.1_F | chr7:55214292-55214447 | CTCACCGCAGTTCCATTCT |
| EGFR.4.1.EGFR.1_R | chr7:55214292-55214447 | CTGGAGGCAGAGATAGTGTG |
| EGFR.24.1.EGFR.1_F | chr7:55260420-55260557 | ACTTTTTCCAACAGAGGGAAAC |
| EGFR.24.1.EGFR.1_R | chr7:55260420-55260557 | TTGGCCTCAGTACAAACTCA |
| EGFR.10.1.EGFR.1_F | chr7:55224452-55224586 | TCACCCTGTTGTTTGTTTCAG |
| EGFR.10.1.EGFR.1_R | chr7:55224452-55224586 | AGGGAACAGGAAATATGTCGA |
| EGFR.19.1.EGFR.1_F | chr7:55240695-55240853 | GCCTAAGATCCCGTCCATC |
| EGFR.19.1.EGFR.1_R | chr7:55240695-55240853 | TTGTTCCTGTCGGGGTG |
| EGFR.19.2.EGFR.2_F | chr7:55240594-55240747 | ACAAGATGTCAGTGCACTGA |
| EGFR.19.2.EGFR.2_R | chr7:55240594-55240747 | TTCGCATGAAGAGGCCG |
| EGFR.5.1.EGFR.1_F | chr7:55218943-55219092 | GAAAGGGCGTCATCAGTTTC |
| EGFR.5.1.EGFR.1_R | chr7:55218943-55219092 | CAAGTGAAGGAAGAGAGGGG |
| AKT1.6.3.AKT1.1_F | chr14:105239742-105239901 | AAAGGTGGCCTCAGGTCA |
| AKT1.6.3.AKT1.1_R | chr14:105239742-105239901 | CAGCTGTTCTTCCACCTGT |
| AKT1.6.4.AKT1.2_F | chr14:105239834-105239985 | TTCTTCTCCGAGTGCAGGTA |
| AKT1.6.4.AKT1.2_R | chr14:105239834-105239985 | GAGACCCGGTCTGAGAAAC |
| AKT1.11.5.AKT1.1_F | chr14:105242953-105243117 | TGTCCCTCCTAAGCGCT |
| AKT1.11.5.AKT1.1_R | chr14:105242953-105243117 | GCAGACAGAGGCTCTGC |
| AKT1.3.1.AKT1.1_F | chr14:105238666-105238799 | GAGTGTGGATATGTGGGGAG |
| AKT1.3.1.AKT1.1_R | chr14:105238666-105238799 | GACACTGTGGCCTTGTTTC |
| AKT1.7.1.AKT1.1_F | chr14:105240168-105240321 | TCTGGTGCCATGGAGAGTA |
| AKT1.7.1.AKT1.1_R | chr14:105240168-105240321 | TTCCAGCTCCCCTTCCTT |
| AKT1.9.3.AKT1.1_F | chr14:105241362-105241516 | TAAAGCAGGGCTGGGTGA |
| AKT1.9.3.AKT1.1_R | chr14:105241362-105241516 | CGAGTTTGAGTACCTGAAGC |
| AKT1.9.4.AKT1.2_F | chr14:105241495-105241655 | CCTTCACCAGGATCACCTTG |
| AKT1.9.4.AKT1.2_R | chr14:105241495-105241655 | CACCAGGTGTGAAGTGGAC |
| AKT1.2.1.AKT1.1_F | chr14:105237046-105237197 | CCTCTCTGAGTGTGGAGAGA |
| AKT1.2.1.AKT1.1_R | chr14:105237046-105237197 | CTCATGACTGTCCCGTCTG |
| AKT1.12.1.AKT1.1_F | chr14:105246435-105246582 | GATACTTACGCGCCACAGA |
| AKT1.12.1.AKT1.1_R | chr14:105246435-105246582 | GTCTGACGGGTAGAGTGTG |
| AKT1.12.2.AKT1.2_F | chr14:105246407-105246543 | CCCAAATCTGAATCCCGAGA |
| AKT1.12.2.AKT1.2_R | chr14:105246407-105246543 | ACGTCTGTAGGGGAGTACAT |
| AKT1.8.1.AKT1.1_F | chr14:105241206-105241343 | CCAAGAAGACAGGACATCGT |
| AKT1.8.1.AKT1.1_R | chr14:105241206-105241343 | TCACCCAGCCCTGCTTTA |
| AKT1.4.1.AKT1.1_F | chr14:105239235-105239399 | CTGCTTGGGGTCCTTCTT |
| AKT1.4.1.AKT1.1_R | chr14:105239235-105239399 | ATGACTACGGCCGTGCA |
| AKT1.4.2.AKT1.2_F | chr14:105239380-105239509 | TCATCTCGTACATGACCACG |
| AKT1.4.2.AKT1.2_R | chr14:105239380-105239509 | AATGCTGTGTCCTCTCTGTG |
| AKT1.4.2.AKT1.3_F | chr14:105239188-105239318 | ATGAGGGGATGGAGGTGTAG |
| AKT1.4.2.AKT1.3_R | chr14:105239188-105239318 | ACAACCAGGACCATGAGAAG |
| AKT1.1.1.AKT1.1_F | chr14:105236629-105236760 | ATACAGATCATGGCACGAGG |
| AKT1.1.1.AKT1.1_R | chr14:105236629-105236760 | TCTGAGCTGTCTACACCCA |
| AKT1.10.1.AKT1.1_F | chr14:105242029-105242165 | CAGGGACACCTCCATCTCT |
| AKT1.10.1.AKT1.1_R | chr14:105242029-105242165 | GGAGCTCCTGATCTGGTACA |
| AKT1.10.2.AKT1.2_F | chr14:105241904-105242063 | GAGTGCTGAGTGTCTCCTG |
| AKT1.10.2.AKT1.2_R | chr14:105241904-105242063 | AGGAGGAGGAGATGGACTTC |
| AKT1.13.1.AKT1.1_F | chr14:105258935-105259068 | TGGCAGCGGGTACTAAC |
| AKT1.13.1.AKT1.1_R | chr14:105258935-105259068 | TCACATTCAGCTTCCTTTGC |
| AKT1.5.1.AKT1.1_F | chr14:105239571-105239723 | CAGCAACGCGTATGCAC |
| AKT1.5.1.AKT1.1_R | chr14:105239571-105239723 | TGACCTGAGGCCACCTTT |
| MSH2.11.5.MSH2.1_F | chr2:47698066-47698223 | ACACATTGCTTCTAGTACACAT |
| MSH2.11.5.MSH2.1_R | chr2:47698066-47698223 | GCCAGGTGACATTCAGAACA |
| MSH2.7.1.MSH2.1_F | chr2:47656971-47657131 | TCGATTCCCAGATCTTAACCG |
| MSH2.7.1.MSH2.1_R | chr2:47656971-47657131 | AGGACAGCACATTGCCAA |
| MSH2.7.2.MSH2.2_F | chr2:47656825-47656981 | TGAGACTTACGTGCTTAGTTGA |
| MSH2.7.2.MSH2.2_R | chr2:47656825-47656981 | TGCTGCTTGTCTTTGAAACT |
| MSH2.2.3.MSH2.1_F | chr2:47635498-47635655 | CAGCTAATACAGTGCTTGAACA |
| MSH2.2.3.MSH2.1_R | chr2:47635498-47635655 | TCATTCTCCTTGGATGCCTT |
| MSH2.2.10.MSH2.2_F | chr2:47635633-47635784 | GGTTCGTCAGTATAGAGTTGAAG |
| MSH2.2.10.MSH2.2_R | chr2:47635633-47635784 | CTCAAACCATTCTACTATCACAATC |
| MSH2.17.1.MSH2.1_F | chr2:47739417-47739566 | CCCGTGGAACTCCTACATTT |
| MSH2.17.1.MSH2.1_R | chr2:47739417-47739566 | AACAGACACCAGGTCATGAG |
| MSH2.1.1.MSH2.1_F | chr2:47630331-47630491 | GAGGTGAGGAGGTTTCGAC |
| MSH2.1.1.MSH2.1_R | chr2:47630331-47630491 | TCTTGAACACCTCCCGG |

| | | |
|---|---|---|
| MSH2.1.2.MSH2.2_F | chr2:47630461-47630598 | GACCGGGGCGACTTCTATA |
| MSH2.1.2.MSH2.2_R | chr2:47630461-47630598 | ACTCTCTGAGGCGGGAAA |
| MSH2.18.5.MSH2.1_F | chr2:47888122-47888263 | TTTTGAGACAAGGTGTCATTCT |
| MSH2.18.5.MSH2.1_R | chr2:47888122-47888263 | TCATTTGACAATTTCCTCCCA |
| MSH2.18.6.MSH2.2_F | chr2:47887997-47888134 | GTTAGGCCCCTGGAAATCTT |
| MSH2.18.6.MSH2.2_R | chr2:47887997-47888134 | AGATCGTGGCATTGCACTC |
| MSH2.13.1.MSH2.1_F | chr2:47703555-47703714 | ATTCGACAAACTGGGGTGAT |
| MSH2.13.1.MSH2.1_R | chr2:47703555-47703714 | CAAGGGACTAGGAGATGCAC |
| MSH2.13.2.MSH2.2_F | chr2:47703412-47703570 | GTCAGTGTAAACCTACGCGA |
| MSH2.13.2.MSH2.2_R | chr2:47703412-47703570 | CATGGCACAAAACACCCAAT |
| MSH2.16.1.MSH2.1_F | chr2:47709957-47710092 | GGAGTTCCTGTCCAAGGTG |
| MSH2.16.1.MSH2.1_R | chr2:47709957-47710092 | ACCTTCATTCCATTACTGGGA |
| MSH2.16.10.MSH2.2_F | chr2:47709871-47710012 | TGTCACTGTCTAACATGACTTT |
| MSH2.16.10.MSH2.2_R | chr2:47709871-47710012 | CTTTGCTATTACTTCAGCTTTTAGC |
| MSH2.6.1.MSH2.1_F | chr2:47643454-47643606 | AGGGTTCTGTTGAAGATACCA |
| MSH2.6.1.MSH2.1_R | chr2:47643454-47643606 | ATCATGTGGGTAACTGCAGG |
| MSH2.6.2.MSH2.2_F | chr2:47643378-47643521 | TCCTCTGTTTTTCATGGCGT |
| MSH2.6.2.MSH2.2_R | chr2:47643378-47643521 | AGAGGCTGCTTAATCCACTG |
| MSH2.3.1.MSH2.1_F | chr2:47637312-47637473 | GCTTCCATTGGTGTTGTGG |
| MSH2.3.1.MSH2.1_R | chr2:47637312-47637473 | TCTCCAGCAGTCTCTCCTC |
| MSH2.3.2.MSH2.2_F | chr2:47637406-47637540 | GGACTGTGTGAATTCCCTGA |
| MSH2.3.2.MSH2.2_R | chr2:47637406-47637540 | CCTTTCCTAGGCCTGGAATC |
| MSH2.3.11.MSH2.3_F | chr2:47637164-47637316 | GGTTCATAGAGTTTGGATTTTTCC |
| MSH2.3.11.MSH2.3_R | chr2:47637164-47637316 | CCATCAACTGCGGACATTTT |
| MSH2.9.1.MSH2.1_F | chr2:47690170-47690301 | GTCACTTTGTTCTGTTTGCAG |
| MSH2.9.1.MSH2.1_R | chr2:47690170-47690301 | ATTCCAACCTCCAATGACCC |
| MSH2.12.1.MSH2.1_F | chr2:47702254-47702412 | GTGTCAAATGGAGCACCTG |
| MSH2.12.1.MSH2.1_R | chr2:47702254-47702412 | AGCCCAAAAACCAGGTTTTT |
| MSH2.12.9.MSH2.2_F | chr2:47702105-47702255 | GGGGGATTAAATGTATTTTTACGGC |
| MSH2.12.9.MSH2.2_R | chr2:47702105-47702255 | ATGGCTGGTCGTACATATGG |
| MSH2.15.3.MSH2.1_F | chr2:47707832-47707990 | ACGCTTCCCCAAATTTCTTA |
| MSH2.15.3.MSH2.1_R | chr2:47707832-47707990 | CCTCTCTTTCCAGATAGCACT |
| MSH2.15.4.MSH2.2_F | chr2:47707930-47708061 | AACAGAAAGCCCTGGAACTT |
| MSH2.15.4.MSH2.2_R | chr2:47707930-47708061 | ACCTTCATCTTAGTGTCCTGT |
| MSH2.14.5.MSH2.1_F | chr2:47705535-47705684 | ACAAAGATTGGTGCTTTTTGC |
| MSH2.14.5.MSH2.1_R | chr2:47705535-47705684 | GTTTCCCATTACCAAGTTCTGA |
| MSH2.14.6.MSH2.2_F | chr2:47705419-47705565 | TATGTGCTTCAGGTCTGCAA |
| MSH2.14.6.MSH2.2_R | chr2:47705419-47705565 | TGGTATCTGATTGGCCAAGG |
| MSH2.14.7.MSH2.3_F | chr2:47705329-47705469 | TTGTGGCATATCCTTCCCAA |
| MSH2.14.7.MSH2.3_R | chr2:47705329-47705469 | TGCTAACCCAAATCCATCGT |
| MSH2.8.12.MSH2.1_F | chr2:47672697-47672839 | ACTACTTTCTTTTAGGAAAACACCA |
| MSH2.8.12.MSH2.1_R | chr2:47672697-47672839 | TCTTAAAGTGGCCTTTGCTT |
| MSH2.8.13.MSH2.2_F | chr2:47672576-47672708 | ACTTTGGAGACCTGCTGTAC |
| MSH2.8.13.MSH2.2_R | chr2:47672576-47672708 | GAGGAGTCACAAAAACTGCC |
| MSH2.4.14.MSH2.1_F | chr2:47639583-47639812 | TCAAAGAGGAGGAATTCTGATCACA |
| MSH2.4.14.MSH2.1_R | chr2:47639583-47639812 | TCATTGATACACAGTTTAGGTTTTGA |
| MSH2.4.15.MSH2.2_F | chr2:47639437-47639593 | ATCAGTGTCTTGCACATTGT |
| MSH2.4.15.MSH2.2_R | chr2:47639437-47639593 | GTCTTTTGTGGAAAAGTCAGCT |
| MSH2.19.1.MSH2.1_F | chr2:47905291-47905440 | TGTCAGTTTGAAGGAGGCAA |
| MSH2.19.1.MSH2.1_R | chr2:47905291-47905440 | TTGTGAGCTTGGCTATGTGA |
| MSH2.10.5.MSH2.1_F | chr2:47693846-47693994 | GATTCCAGTGCACAGTTTGG |
| MSH2.10.5.MSH2.1_R | chr2:47693846-47693994 | ACATTCACATCATGTTAGAGCA |
| MSH2.5.8.MSH2.1_F | chr2:47641383-47641537 | CTGGATCCAGTGGTATAGAAATCT |
| MSH2.5.8.MSH2.1_R | chr2:47641383-47641537 | CCTGAAAAAGGTTAAGGGCTC |
| MSH2.5.10.MSH2.2_F | chr2:47641487-47641631 | TCCAACTTTGGACAGTTTGAAC |
| MSH2.5.10.MSH2.2_R | chr2:47641487-47641631 | GCTCCTTTATAAGCTTCTTCAGT |
| JAK2.11.1.JAK2.1_F | chr9:5072508-5072660 | GAAGAATGAAAGCCTTGGCC |
| JAK2.11.1.JAK2.1_R | chr9:5072508-5072660 | GAGAGCACATCTTTAAACAGCA |
| JAK2.11.2.JAK2.2_F | chr9:5072453-5072605 | TGTTCCTACTTCGTTCTCCA |
| JAK2.11.2.JAK2.2_R | chr9:5072453-5072605 | CCTCTGAATAGTTTCTGTGTGC |
| JAK2.21.3.JAK2.1_F | chr9:5122961-5123118 | GCAGGTAAAATCAAGAGTCCAC |
| JAK2.21.3.JAK2.1_R | chr9:5122961-5123118 | ATAAAAAGCACACTGACCGC |
| JAK2.21.4.JAK2.2_F | chr9:5123074-5123222 | TTTGGAGCTTTGGAGTGGTT |
| JAK2.21.4.JAK2.2_R | chr9:5123074-5123222 | AAAGCTCTGACTTCACCACT |
| JAK2.7.3.JAK2.1_F | chr9:5064857-5065012 | GAGTTGACTTTCTAAAAGGTGC |
| JAK2.7.3.JAK2.1_R | chr9:5064857-5065012 | GCCATGACAGTTGCTTTGTA |
| JAK2.7.5.JAK2.2_F | chr9:5065000-5065159 | GAAGTAGCACCTCCAGCC |

| | | |
|---|---|---|
| JAK2.7.5.JAK2.2_R | chr9:5065000-5065159 | CTGATTGCAAATAGCCTTTTGT |
| JAK2.17.7.JAK2.1_F | chr9:5081692-5081844 | AGTCCAGAGAATGTTATTTGCT |
| JAK2.17.7.JAK2.1_R | chr9:5081692-5081844 | TACCTTGCCAAGTTGCTGTA |
| JAK2.17.8.JAK2.2_F | chr9:5081825-5081980 | CGGGATCCTACACAGTTTGA |
| JAK2.17.8.JAK2.2_R | chr9:5081825-5081980 | TCTCCAACATCTGACAACCA |
| JAK2.2.5.JAK2.1_F | chr9:5029781-5029936 | TTCCTTTCTCTGCTTCTTTTCT |
| JAK2.2.5.JAK2.1_R | chr9:5029781-5029936 | CTCCCATTTTGCCTTTTAGCA |
| JAK2.22.5.JAK2.1_F | chr9:5126335-5126464 | TGAAAGTGGGTTTGTTTTAGGA |
| JAK2.22.5.JAK2.1_R | chr9:5126335-5126464 | AAATGCATGACTACCCTGGA |
| JAK2.22.6.JAK2.2_F | chr9:5126271-5126417 | TTCCCATTGACTGGAGGAAA |
| JAK2.22.6.JAK2.2_R | chr9:5126271-5126417 | GCATCCATCTGGTCTTGGTA |
| JAK2.1.1.JAK2.1_F | chr9:5021998-5022157 | AAGACTCTGCATGGGAATGG |
| JAK2.1.1.JAK2.1_R | chr9:5021998-5022157 | CAACATACTCCCCAGATGGA |
| JAK2.1.2.JAK2.2_F | chr9:5022155-5022311 | AATCTGAGGCAGATTATCTGAC |
| JAK2.1.2.JAK2.2_R | chr9:5022155-5022311 | GCCAGCCATACAAGCATTAA |
| JAK2.1.2.JAK2.3_F | chr9:5021861-5022020 | GAGGCTTGTCTCCAACTTCT |
| JAK2.1.2.JAK2.3_R | chr9:5021861-5022020 | GAAGAGGTGGATGTTCCCTC |
| JAK2.18.1.JAK2.1_F | chr9:5089711-5089866 | GAGATGTGCCGGTATGACC |
| JAK2.18.1.JAK2.1_R | chr9:5089711-5089866 | ATAGGTTTCAATGGGCAGCT |
| JAK2.18.2.JAK2.2_F | chr9:5089587-5089729 | ACAGTCTGCTAATTCCAGCT |
| JAK2.18.2.JAK2.2_R | chr9:5089587-5089729 | TTTTTACAGCGACCACCTCC |
| JAK2.13.16.JAK2.1_F | chr9:5077471-5077680 | TGCAGATATTCTGGTTCAGGAGT |
| JAK2.13.16.JAK2.1_R | chr9:5077471-5077680 | ACACAGACTATTTTACATGAATTGGCA |
| JAK2.13.17.JAK2.2_F | chr9:5077380-5077532 | ACCTATAATGGTCACATGTAAGTAT |
| JAK2.13.17.JAK2.2_R | chr9:5077380-5077532 | GTTTAGCAACTTCAAGTTTCCA |
| JAK2.16.3.JAK2.1_F | chr9:5080496-5080643 | AGAAGGTTGGTGTGGCATTA |
| JAK2.16.3.JAK2.1_R | chr9:5080496-5080643 | AGATCTCGTATGATGGCTCT |
| JAK2.16.4.JAK2.2_F | chr9:5080639-5080782 | TGAACCAGATTTCAGGCCTT |
| JAK2.16.4.JAK2.2_R | chr9:5080639-5080782 | TTAAGAGGGCCCAAATGACA |
| JAK2.23.1.JAK2.1_F | chr9:5126654-5126797 | AAGATGGCCCTTAGTGTTCA |
| JAK2.23.1.JAK2.1_R | chr9:5126654-5126797 | TGGTCTCAGAATGAAGGTCA |
| JAK2.6.7.JAK2.1_F | chr9:5055641-5055768 | GGCTCTGTAAATTCTACCCGT |
| JAK2.6.7.JAK2.1_R | chr9:5055641-5055768 | ACCAGATTTTTACCATCTTGCT |
| JAK2.6.11.JAK2.2_F | chr9:5055754-5055907 | GGGTTCAAATGAAAGCCGAG |
| JAK2.6.11.JAK2.2_R | chr9:5055754-5055907 | AGTTTACTGAACTGATGTTTAGAGT |
| JAK2.3.16.JAK2.1_F | chr9:5044382-5044633 | TGAACTATTTGGAAGCTGACCA |
| JAK2.3.16.JAK2.1_R | chr9:5044382-5044633 | ACTTCTCTGCACCTGCTGTG |
| JAK2.9.1.JAK2.1_F | chr9:5069092-5069246 | AATGAAGAGTACAACCTCAGTG |
| JAK2.9.1.JAK2.1_R | chr9:5069092-5069246 | CACGCCACATAAACAATCCA |
| JAK2.9.2.JAK2.2_F | chr9:5068997-5069139 | CCATTGTGACTATCCCTCCC |
| JAK2.9.2.JAK2.2_R | chr9:5068997-5069139 | TGAGCGAACAGTTTCCATCT |
| JAK2.12.1.JAK2.1_F | chr9:5073671-5073811 | TGGACAACAGTCAAACAACA |
| JAK2.12.1.JAK2.1_R | chr9:5073671-5073811 | ACAGATGCTCTGAGAAAGGC |
| JAK2.14.1.JAK2.1_F | chr9:5078335-5078492 | ACACCCTTATTCATGGGAATGT |
| JAK2.14.1.JAK2.1_R | chr9:5078335-5078492 | AACATGCCCTTTACACCACT |
| JAK2.14.2.JAK2.2_F | chr9:5078271-5078425 | ATGCTCCAGTACTTGTGGAC |
| JAK2.14.2.JAK2.2_R | chr9:5078271-5078425 | CTGTCCTTTGGCAAAACTGT |
| JAK2.15.3.JAK2.1_F | chr9:5080221-5080361 | AACCCTACTCTGTTCGTATCA |
| JAK2.15.3.JAK2.1_R | chr9:5080221-5080361 | ACTCTTTGAGAATCCAGAGCA |
| JAK2.15.4.JAK2.2_F | chr9:5080339-5080475 | CCACTTTGTGGGAAATCTGC |
| JAK2.15.4.JAK2.2_R | chr9:5080339-5080475 | TAATGCCACACCAACCTTCT |
| JAK2.20.5.JAK2.1_F | chr9:5090787-5090944 | ATATCCACAGGGATCTGGCA |
| JAK2.20.5.JAK2.1_R | chr9:5090787-5090944 | AAAGTTTGAAGTCTGTGCTCT |
| JAK2.20.10.JAK2.2_F | chr9:5090685-5090837 | AGGGAATATATAGGGTTAAGACCA |
| JAK2.20.10.JAK2.2_R | chr9:5090685-5090837 | GGCAAGACTTTGGTTAACCC |
| JAK2.8.10.JAK2.1_F | chr9:5066641-5066789 | CACTTGGTCATAATATTATGGTGCT |
| JAK2.8.10.JAK2.1_R | chr9:5066641-5066789 | CCACTAATAAGTGTGACATACTAAC |
| JAK2.4.11.JAK2.1_F | chr9:5050693-5050836 | TTTTTGGTTTTAGTGGCGGC |
| JAK2.4.11.JAK2.1_R | chr9:5050693-5050836 | TGTGTAAGGATTTGCAAAAGAAA |
| JAK2.4.12.JAK2.2_F | chr9:5050582-5050723 | AGCCTGGCCAATTTGTATCT |
| JAK2.4.12.JAK2.2_R | chr9:5050582-5050723 | TCCTGTGTTTCATGAGTCACA |
| JAK2.10.16.JAK2.1_F | chr9:5069889-5070112 | TGGAGCAATTCATACTTTCAGTGT |
| JAK2.10.16.JAK2.1_R | chr9:5069889-5070112 | TCCAATGTCACATGAATGTAAATCA |
| JAK2.19.1.JAK2.1_F | chr9:5090456-5090594 | ATGTTAAAAGGTCGGCGTAA |
| JAK2.19.1.JAK2.1_R | chr9:5090456-5090594 | ACACGGTTGCTTCATCTACA |
| JAK2.19.9.JAK2.2_F | chr9:5090433-5090581 | GGCAGAGTAAAACATTATTTCCACC |
| JAK2.19.9.JAK2.2_R | chr9:5090433-5090581 | GCTTCATCTACAGCAAATAATCAGG |

| | | |
|---|---|---|
| JAK2.5.13.JAK2.1_F | chr9:5054550-5054685 | TGTTTTGTTTTTCTGTATGTGCT |
| JAK2.5.13.JAK2.1_R | chr9:5054550-5054685 | ACTTAAGTTTCAAGTTTCTGGCA |
| JAK2.5.14.JAK2.2_F | chr9:5054681-5054835 | AGCAATTCAGCCAATGCAAA |
| JAK2.5.14.JAK2.2_R | chr9:5054681-5054835 | GTTTCCCTCTTGACCACTGA |
| JAK2.5.15.JAK2.3_F | chr9:5054811-5054967 | GTGAGGAGATTTTTGCAACCA |
| JAK2.5.15.JAK2.3_R | chr9:5054811-5054967 | TGGGAGAAGTGCAATACCAT |
| PIK3R1.11.6.PIK3R1.1_F | chr5:67589187-67589340 | TCGAGATGGGAAATATGGCT |
| PIK3R1.11.6.PIK3R1.1_R | chr5:67589187-67589340 | AAGGATCTTGTCTAAACATCGT |
| PIK3R1.11.7.PIK3R1.2_F | chr5:67589041-67589200 | CACTAAGGTAAGCCAGGGAA |
| PIK3R1.11.7.PIK3R1.2_R | chr5:67589041-67589200 | TCAACCACAGAACTGAAGGT |
| PIK3R1.7.1.PIK3R1.1_F | chr5:67584507-67584642 | ACTTGGTGGAAGAACAGCTT |
| PIK3R1.7.1.PIK3R1.1_R | chr5:67584507-67584642 | ACACACACGTACTCCAAGAC |
| PIK3R1.2.1.PIK3R1.1_F | chr5:67569163-67569310 | TGTACGTCCTTCATTGTGGT |
| PIK3R1.2.1.PIK3R1.1_R | chr5:67569163-67569310 | CCCTCTAGCAGTCTGGTTAC |
| PIK3R1.17.1.PIK3R1.1_F | chr5:67593247-67593409 | CTCTCCTCTCTAGGGTGGAC |
| PIK3R1.17.1.PIK3R1.1_R | chr5:67593247-67593409 | TCGCCTCTGCTGTGCAT |
| PIK3R1.17.2.PIK3R1.2_F | chr5:67593363-67593509 | CATTACCAACACACCTCCCT |
| PIK3R1.17.2.PIK3R1.2_R | chr5:67593363-67593509 | TCTGCAGCTCAATTCACAGA |
| PIK3R1.17.2.PIK3R1.3_F | chr5:67593194-67593342 | TGCCCTGAAGAGTTGTGAAT |
| PIK3R1.17.2.PIK3R1.3_R | chr5:67593194-67593342 | AGGGAGGTGTGTTGGTAATG |
| PIK3R1.1.1.PIK3R1.1_F | chr5:67522522-67522683 | CATGAGTGCTGAGGGGTAC |
| PIK3R1.1.1.PIK3R1.1_R | chr5:67522522-67522683 | CCCTTTCCCCTGTGGTTTC |
| PIK3R1.1.2.PIK3R1.2_F | chr5:67522496-67522626 | ACTGTTGCATGGTAGCAGAT |
| PIK3R1.1.2.PIK3R1.2_R | chr5:67522496-67522626 | GCTTCCTGTCCATCACTGAA |
| PIK3R1.1.2.PIK3R1.3_F | chr5:67522702-67522852 | ATGAAACCACAGGGGAAAGG |
| PIK3R1.1.2.PIK3R1.3_R | chr5:67522702-67522852 | AGGGAGTCATTAAGCAACCA |
| PIK3R1.1.3.PIK3R1.4_F | chr5:67522643-67522794 | TGGATTCAGTGATGGACAGG |
| PIK3R1.1.3.PIK3R1.4_R | chr5:67522643-67522794 | GTTTTCGAAGAACCTGGTGC |
| PIK3R1.13.1.PIK3R1.1_F | chr5:67590382-67590511 | GCAGGAAATCCAAATGAAAAGG |
| PIK3R1.13.1.PIK3R1.1_R | chr5:67590382-67590511 | GCACAAGAACAAGGGAAACA |
| PIK3R1.13.2.PIK3R1.2_F | chr5:67590308-67590440 | ATGTCCTGGTAGTGTCTTGC |
| PIK3R1.13.2.PIK3R1.2_R | chr5:67590308-67590440 | ATTCTTTGCTGTACCGCTCT |
| PIK3R1.16.1.PIK3R1.1_F | chr5:67592069-67592227 | AGACATGGAATGTTGGAAGC |
| PIK3R1.16.1.PIK3R1.1_R | chr5:67592069-67592227 | CCCCCAAAACTCAATGAATGA |
| PIK3R1.16.2.PIK3R1.2_F | chr5:67591940-67592080 | GTATGCCTAGGGAAGACAGC |
| PIK3R1.16.2.PIK3R1.2_R | chr5:67591940-67592080 | CGCAACAGGTTTTCAGCTTT |
| PIK3R1.6.9.PIK3R1.1_F | chr5:67576745-67576893 | GGGAAAAGGTTTCTAATAAACTCTC |
| PIK3R1.6.9.PIK3R1.1_R | chr5:67576745-67576893 | TCAAAGGAGGTTTATAGCCATCA |
| PIK3R1.3.1.PIK3R1.1_F | chr5:67569731-67569877 | GTTCTCCAGAGAGCTGTGTT |
| PIK3R1.3.1.PIK3R1.1_R | chr5:67569731-67569877 | AACAGAAAGCCAAGACCTGT |
| PIK3R1.9.1.PIK3R1.1_F | chr5:67588073-67588231 | GGGATTGCGAACAACTTTTTC |
| PIK3R1.9.1.PIK3R1.1_R | chr5:67588073-67588231 | AGCTGGTGCTTTTCTTTCTT |
| PIK3R1.12.10.PIK3R1.1_F | chr5:67589491-67589740 | TGTCAGCTATTTTGTTAAACAATTGTT |
| PIK3R1.12.10.PIK3R1.1_R | chr5:67589491-67589740 | CCTCATTCGTAAAAACTCAACCT |
| PIK3R1.14.4.PIK3R1.1_F | chr5:67590976-67591132 | TACGTTTCTTTTGCCTGCAG |
| PIK3R1.14.4.PIK3R1.1_R | chr5:67590976-67591132 | ACATCAAGTATTGGTCTCTCGT |
| PIK3R1.14.5.PIK3R1.2_F | chr5:67591129-67591268 | CCAGACCTTATCCAGCTGAG |
| PIK3R1.14.5.PIK3R1.2_R | chr5:67591129-67591268 | CGTTCAACTTCTTTTGCCGA |
| PIK3R1.15.8.PIK3R1.1_F | chr5:67591210-67591334 | TGGTGATTGCTACAATTCAGG |
| PIK3R1.15.8.PIK3R1.1_R | chr5:67591210-67591334 | GAGAAAAATCTTCTGCTATCACCA |
| PIK3R1.8.1.PIK3R1.1_F | chr5:67586543-67586684 | ATAACTGAGCTCAGCCAAGG |
| PIK3R1.8.1.PIK3R1.1_R | chr5:67586543-67586684 | GCCACGAAATTAACTAACTCTC |
| PIK3R1.4.1.PIK3R1.1_F | chr5:67575443-67575597 | TTCCTAGATACACCCTCCGT |
| PIK3R1.4.1.PIK3R1.1_R | chr5:67575443-67575597 | GCACCTCTACATCTCCATGT |
| PIK3R1.4.2.PIK3R1.2_F | chr5:67575380-67575524 | GTTCAGAAAATTAGCCCAACTG |
| PIK3R1.4.2.PIK3R1.2_R | chr5:67575380-67575524 | ATTTCACTGTAAACGGCTGC |
| PIK3R1.10.1.PIK3R1.1_F | chr5:67588900-67589029 | TCTGTGGTCACTAAACCTTAAG |
| PIK3R1.10.1.PIK3R1.1_R | chr5:67588900-67589029 | TCAGCTATATTCCCTGGCTT |
| PIK3R1.5.1.PIK3R1.1_F | chr5:67576386-67576545 | AGCTCCGAAGAATATATTCAGC |
| PIK3R1.5.1.PIK3R1.1_R | chr5:67576386-67576545 | ACTTACCTGGCTGCTGAG |
| PIK3R1.5.2.PIK3R1.2_F | chr5:67576489-67576642 | CTCTCTCAAACCTCCAGCAA |
| PIK3R1.5.2.PIK3R1.2_R | chr5:67576489-67576642 | GCACATGCATCTCTGGAGTA |
| PIK3R1.5.2.PIK3R1.3_F | chr5:67576236-67576395 | TACTGTGTGCTTCTCCCAAC |
| PIK3R1.5.2.PIK3R1.3_R | chr5:67576236-67576395 | TGCTAGGCGACCTAATAAGC |
| NOTCH1.33.1.NOTCH1.1_F | chr9:139438406-139438565 | AATGGCCTAGTGTTCTGTCC |
| NOTCH1.33.1.NOTCH1.1_R | chr9:139438406-139438565 | GACTGACCTCTCTTCTCCTG |
| NOTCH1.32.1.NOTCH1.1_F | chr9:139418278-139418440 | TTGTCCAGGGGTGTCAG |

| | | |
|---|---|---|
| NOTCH1.32.1.NOTCH1.1_R | chr9:139418278-139418440 | CTAACTGCCCTGGCACATC |
| NOTCH1.32.10.NOTCH1.2_F | chr9:139418162-139418305 | CTCAGGCTGTGGGTCCT |
| NOTCH1.32.10.NOTCH1.2_R | chr9:139418162-139418305 | CAGACTATGCCTGCAGCTG |
| NOTCH1.21.1.NOTCH1.1_F | chr9:139407835-139408000 | CACTGAGAAACGCGCAG |
| NOTCH1.21.1.NOTCH1.1_R | chr9:139407835-139408000 | TCGACCTGCAGTGTGGT |
| NOTCH1.7.10.NOTCH1.1_F | chr9:139396730-139396893 | CGTCCGCTCACTTGAGG |
| NOTCH1.7.10.NOTCH1.1_R | chr9:139396730-139396893 | GCAGCTGCACTTCATGTAC |
| NOTCH1.7.11.NOTCH1.2_F | chr9:139396883-139397044 | GAACAGAAGCACAAAGGCG |
| NOTCH1.7.11.NOTCH1.2_R | chr9:139396883-139397044 | ATGGAGGAGAGTGGGTGAG |
| NOTCH1.7.11.NOTCH1.3_F | chr9:139396600-139396761 | TAGGAAGCCCCCAGAGA |
| NOTCH1.7.11.NOTCH1.3_R | chr9:139396600-139396761 | TGAGGCCAGCAAGAAGAAG |
| NOTCH1.26.13.NOTCH1.1_F | chr9:139411686-139411843 | TCAGGAGGCCAGGGTG |
| NOTCH1.26.13.NOTCH1.1_R | chr9:139411686-139411843 | CTCACTGTTGCCCCACC |
| NOTCH1.2.1.NOTCH1.1_F | chr9:139393304-139393448 | TTTGGCCCTCACTTCTCTG |
| NOTCH1.2.1.NOTCH1.1_R | chr9:139393304-139393448 | CATCTGCTTCTTTCACGCAG |
| NOTCH1.17.1.NOTCH1.1_F | chr9:139404201-139404364 | CACCTCTCTGTGCAGTCAG |
| NOTCH1.17.1.NOTCH1.1_R | chr9:139404201-139404364 | GGCATCAACACGGCCTT |
| NOTCH1.17.2.NOTCH1.2_F | chr9:139404339-139404499 | GTCCTCCTCACAGAAAGTGC |
| NOTCH1.17.2.NOTCH1.2_R | chr9:139404339-139404499 | AACGAAAGGCTGGGCTG |
| NOTCH1.17.2.NOTCH1.3_F | chr9:139404047-139404205 | ACAGGTCGGTACAATGAACA |
| NOTCH1.17.2.NOTCH1.3_R | chr9:139404047-139404205 | GGGATCCACTGTGAGAACAA |
| NOTCH1.1.1.NOTCH1.1_F | chr9:139390541-139390703 | TTTACTTGAAGGCCTCCGG |
| NOTCH1.1.1.NOTCH1.1_R | chr9:139390541-139390703 | TCCTCGCCTGTGGACAA |
| NOTCH1.1.2.NOTCH1.2_F | chr9:139390447-139390595 | TAAAAAGGCTCCTCTGGTCG |
| NOTCH1.1.2.NOTCH1.2_R | chr9:139390447-139390595 | ATTCCAACGTCTCCGACTG |
| NOTCH1.1.2.NOTCH1.3_F | chr9:139390984-139391148 | CAGGCTTTGCTGCTGCT |
| NOTCH1.1.2.NOTCH1.3_R | chr9:139390984-139391148 | TGCAGCATGGCATGGTAG |
| NOTCH1.1.3.NOTCH1.4_F | chr9:139390829-139390983 | CTCTCCTGGGGCAGAATAGT |
| NOTCH1.1.3.NOTCH1.4_R | chr9:139390829-139390983 | GCAGCCAGCAAACATCC |
| NOTCH1.1.3.NOTCH1.5_F | chr9:139391317-139391480 | ACAGTGAAATTCAGGGCCC |
| NOTCH1.1.3.NOTCH1.5_R | chr9:139391317-139391480 | AACCACCTGCCTGGGAT |
| NOTCH1.1.4.NOTCH1.6_F | chr9:139390718-139390879 | GTGTTGTCCACAGGCGA |
| NOTCH1.1.4.NOTCH1.6_R | chr9:139390718-139390879 | GAGCTTCCTGAGTGGAGAG |
| NOTCH1.1.4.NOTCH1.7_F | chr9:139391498-139391663 | ATCCCAGGCAGGTGGTT |
| NOTCH1.1.4.NOTCH1.7_R | chr9:139391498-139391663 | AAGGAGGCCAAGGACCT |
| NOTCH1.1.5.NOTCH1.8_F | chr9:139390677-139390812 | TGAGGAAGGGGTGCTCA |
| NOTCH1.1.5.NOTCH1.8_R | chr9:139390677-139390812 | CACACTATTCTGCCCCAGG |
| NOTCH1.1.5.NOTCH1.9_F | chr9:139391664-139391828 | TTCTTCCTCCGTGCCTTG |
| NOTCH1.1.5.NOTCH1.9_R | chr9:139391664-139391828 | TACAACCTGGTGCGCAG |
| NOTCH1.1.5.NOTCH1.10_F | chr9:139391396-139391554 | AGGTGGGCCAGTCTCAAA |
| NOTCH1.1.5.NOTCH1.10_R | chr9:139391396-139391554 | CCATGGCTACCTGTCAGAC |
| NOTCH1.1.6.NOTCH1.11_F | chr9:139391144-139391297 | AGCAAGGCTACTGTGCAG |
| NOTCH1.1.6.NOTCH1.11_R | chr9:139391144-139391297 | GGGCCCTGAATTTCACTGT |
| NOTCH1.1.7.NOTCH1.12_F | chr9:139391234-139391377 | AGAGGGTTGTATTGGTTCGG |
| NOTCH1.1.7.NOTCH1.12_R | chr9:139391234-139391377 | TTTGAGACTGGCCCACCT |
| NOTCH1.1.10.NOTCH1.13_F | chr9:139391898-139392058 | GCTCCTGTGCGATGTCG |
| NOTCH1.1.10.NOTCH1.13_R | chr9:139391898-139392058 | AACCTTGGGGAGGGTCC |
| NOTCH1.1.11.NOTCH1.14_F | chr9:139391759-139391906 | TTGAGGCTGCCCAGGTA |
| NOTCH1.1.11.NOTCH1.14_R | chr9:139391759-139391906 | ATCACGGATCATATGGACCG |
| NOTCH1.18.6.NOTCH1.1_F | chr9:139405082-139405229 | TGAGAGCTTCCTGGAGGAG |
| NOTCH1.18.6.NOTCH1.1_R | chr9:139405082-139405229 | GTGAGGTCGACATCAACGA |
| NOTCH1.18.8.NOTCH1.2_F | chr9:139405200-139405354 | TGGGTGTTCTGGCAGGAT |
| NOTCH1.18.8.NOTCH1.2_R | chr9:139405200-139405354 | GTCACGGAAGCCTACTCCT |
| NOTCH1.30.1.NOTCH1.1_F | chr9:139413904-139414042 | TATACGCGCCTGTCCACT |
| NOTCH1.30.1.NOTCH1.1_R | chr9:139413904-139414042 | ACTGGACCTCAGGGAAGAG |
| NOTCH1.30.2.NOTCH1.2_F | chr9:139413837-139413968 | ATGCCTGTGAGTGCAGTTTA |
| NOTCH1.30.2.NOTCH1.2_R | chr9:139413837-139413968 | ATCGACGATTGTCCAGGAAA |
| NOTCH1.16.10.NOTCH1.1_F | chr9:139403342-139403505 | CAGTTGGGGCCAGTGT |
| NOTCH1.16.10.NOTCH1.1_R | chr9:139403342-139403505 | CTCCTGCTTCAACGGTGG |
| NOTCH1.16.11.NOTCH1.2_F | chr9:139403440-139403601 | ACTCATTGACATCGTGCTGG |
| NOTCH1.16.11.NOTCH1.2_R | chr9:139403440-139403601 | TTGAGCAGAAGGGGAGGT |
| NOTCH1.16.12.NOTCH1.3_F | chr9:139403261-139403418 | GTGACCGTTCCCACCTC |
| NOTCH1.16.12.NOTCH1.3_R | chr9:139403261-139403418 | CAGCACGATGTCAATGAGTG |
| NOTCH1.25.13.NOTCH1.1_F | chr9:139410404-139410568 | TGTCCCGTCCCCAGTC |
| NOTCH1.25.13.NOTCH1.1_R | chr9:139410404-139410568 | CTGTTGCACATGACTCCCT |
| NOTCH1.27.1.NOTCH1.1_F | chr9:139412274-139412435 | TTCTGGCACGGGTTCGA |
| NOTCH1.27.1.NOTCH1.1_R | chr9:139412274-139412435 | TGCAGTGAGGTGTTGCAG |

| | | |
|---|---|---|
| NOTCH1.27.2.NOTCH1.2_F | chr9:139412137-139412277 | GAAGCAACCCACAGATGTTC |
| NOTCH1.27.2.NOTCH1.2_R | chr9:139412137-139412277 | AGATCGACGTCAACGAGTG |
| NOTCH1.28.6.NOTCH1.1_F | chr9:139412541-139412696 | GACTTCTCATCGGTTCTGGG |
| NOTCH1.28.6.NOTCH1.1_R | chr9:139412541-139412696 | ATCAGCAACCCCTGTAACG |
| NOTCH1.28.8.NOTCH1.2_F | chr9:139412678-139412812 | CTTGCCATTGACAGGGTTG |
| NOTCH1.28.8.NOTCH1.2_R | chr9:139412678-139412812 | TGTCAGTGGGGTTGGAC |
| NOTCH1.20.10.NOTCH1.1_F | chr9:139407480-139407620 | CCACCCCTCACCTGTGTA |
| NOTCH1.20.10.NOTCH1.1_R | chr9:139407480-139407620 | CAAGGTGCCTGGGAGTG |
| NOTCH1.20.11.NOTCH1.2_F | chr9:139407348-139407508 | CTTTACCCTCCAGTCACGG |
| NOTCH1.20.11.NOTCH1.2_R | chr9:139407348-139407508 | CACGTGTATTGACGACGTTG |
| NOTCH1.14.1.NOTCH1.1_F | chr9:139402443-139402599 | AGGTAGTCCGTGCAGGT |
| NOTCH1.14.1.NOTCH1.1_R | chr9:139402443-139402599 | ACCTGTCTACCACCCCATC |
| NOTCH1.14.2.NOTCH1.2_F | chr9:139402321-139402481 | GGGCCTATCAGGTTCAGTTT |
| NOTCH1.14.2.NOTCH1.2_R | chr9:139402321-139402481 | CAGCTACTGTGAGGACCTG |
| NOTCH1.24.1.NOTCH1.1_F | chr9:139409964-139410126 | CTTCAGGCAGAAGCAGAGG |
| NOTCH1.24.1.NOTCH1.1_R | chr9:139409964-139410126 | TGGACATCGATGAGTGCG |
| NOTCH1.24.2.NOTCH1.2_F | chr9:139410039-139410191 | ACTCGTTGATGTTGGTCTCG |
| NOTCH1.24.2.NOTCH1.2_R | chr9:139410039-139410191 | GCCAGTCCTAAGTCTTCCTG |
| NOTCH1.24.2.NOTCH1.3_F | chr9:139409853-139410011 | TTGATCTCGCAGTTGGGTC |
| NOTCH1.24.2.NOTCH1.3_R | chr9:139409853-139410011 | AACATCAACGAGTGCTCCAG |
| NOTCH1.10.1.NOTCH1.1_F | chr9:139399772-139399935 | AGCCTTACTTGCACTGGC |
| NOTCH1.10.1.NOTCH1.1_R | chr9:139399772-139399935 | CAGTGCAACAACCACGC |
| NOTCH1.10.2.NOTCH1.2_F | chr9:139399732-139399880 | CACATCCAAGTTCAGGTCCT |
| NOTCH1.10.2.NOTCH1.2_R | chr9:139399732-139399880 | TCAATGACCCCTGGAAGAAC |
| NOTCH1.10.2.NOTCH1.3_F | chr9:139399956-139400115 | GTGGTTGTTGCACTGCAG |
| NOTCH1.10.2.NOTCH1.3_R | chr9:139399956-139400115 | ACATCCGAGAGCCCCTT |
| NOTCH1.10.3.NOTCH1.4_F | chr9:139399911-139400052 | GGGTCATTGAAGTTGAGGGA |
| NOTCH1.10.3.NOTCH1.4_R | chr9:139399911-139400052 | CACATCCTGGACTACAGCTT |
| NOTCH1.10.3.NOTCH1.5_F | chr9:139400126-139400291 | ACGGTAGAAGGGGCTCT |
| NOTCH1.10.3.NOTCH1.5_R | chr9:139400126-139400291 | ATGACGCTCGTACCTGC |
| NOTCH1.10.4.NOTCH1.6_F | chr9:139400276-139400425 | ATGTGCCGCCGTTGA |
| NOTCH1.10.4.NOTCH1.6_R | chr9:139400276-139400425 | CCTCCCAGGTTAGAGGAGAG |
| NOTCH1.10.4.NOTCH1.7_F | chr9:139399972-139400127 | AGGCTGCAGACCTTGTTG |
| NOTCH1.10.4.NOTCH1.7_R | chr9:139399972-139400127 | ACCTGTGAGCCCACATC |
| NOTCH1.31.1.NOTCH1.1_F | chr9:139417507-139417640 | CTTCTGGCCACACTCGTT |
| NOTCH1.31.1.NOTCH1.1_R | chr9:139417507-139417640 | CTTGTCCCCTTGTCTCCAG |
| NOTCH1.31.8.NOTCH1.2_F | chr9:139417332-139417495 | ACACTCGTGGGTGACGT |
| NOTCH1.31.8.NOTCH1.2_R | chr9:139417332-139417495 | AGGATGTCAACGAGTGTGG |
| NOTCH1.31.9.NOTCH1.3_F | chr9:139417465-139417629 | ACCTCGTTGTGGCAGGT |
| NOTCH1.31.9.NOTCH1.3_R | chr9:139417465-139417629 | TCTCCAGGGAAATCGTGC |
| NOTCH1.31.13.NOTCH1.4_F | chr9:139417190-139417356 | CATCCCGCCTTCCCAAC |
| NOTCH1.31.13.NOTCH1.4_R | chr9:139417190-139417356 | CTCGCCCTGCCAGAAC |
| NOTCH1.11.10.NOTCH1.1_F | chr9:139400934-139401097 | ACGTGGACCTCTCCAGG |
| NOTCH1.11.10.NOTCH1.1_R | chr9:139400934-139401097 | CTCACTGCCCTGCTCTTAC |
| NOTCH1.22.6.NOTCH1.1_F | chr9:139408949-139409114 | TGTGTCCCCATGATCGG |
| NOTCH1.22.6.NOTCH1.1_R | chr9:139408949-139409114 | GATGAGTGTGCGGGCAA |
| NOTCH1.22.7.NOTCH1.2_F | chr9:139409083-139409214 | CAGGTGAAGCCATTGATGC |
| NOTCH1.22.7.NOTCH1.2_R | chr9:139409083-139409214 | AACAGACAGGGAAATCGAGG |
| NOTCH1.13.1.NOTCH1.1_F | chr9:139401735-139401889 | ATGGATGGCCAACACCAG |
| NOTCH1.13.1.NOTCH1.1_R | chr9:139401735-139401889 | TTCTGTCGCCTGCACAG |
| NOTCH1.23.1.NOTCH1.1_F | chr9:139409712-139409865 | CTTGACCTCTGAGCACAGTG |
| NOTCH1.23.1.NOTCH1.1_R | chr9:139409712-139409865 | AGGACTGACCGACACGT |
| NOTCH1.29.1.NOTCH1.1_F | chr9:139413071-139413235 | ATGGGGACACTCGCAGTA |
| NOTCH1.29.1.NOTCH1.1_R | chr9:139413071-139413235 | ACGAGTGCCAGCTGATG |
| NOTCH1.29.2.NOTCH1.2_F | chr9:139413188-139413322 | TTGACACACACGCAGTTGTA |
| NOTCH1.29.2.NOTCH1.2_R | chr9:139413188-139413322 | TCTTAGGGGACAGGGAGCT |
| NOTCH1.29.2.NOTCH1.3_F | chr9:139412994-139413129 | TGAGGTCACACAGCTCAGG |
| NOTCH1.29.2.NOTCH1.3_R | chr9:139412994-139413129 | CTGCAGCGAGAACATTGATG |
| NOTCH1.6.1.NOTCH1.1_F | chr9:139396388-139396548 | ACATAGAGGGAGTGAGCAGA |
| NOTCH1.6.1.NOTCH1.1_R | chr9:139396388-139396548 | CTCACTTCTCGACCCCTCA |
| NOTCH1.3.1.NOTCH1.1_F | chr9:139393577-139393739 | GCTCACCCAGGTCATCTAC |
| NOTCH1.3.1.NOTCH1.1_R | chr9:139393577-139393739 | TGTACGTCTGCCCAGCA |
| NOTCH1.3.2.NOTCH1.2_F | chr9:139393463-139393593 | CTTGCCTGCGTGAAAGAAG |
| NOTCH1.3.2.NOTCH1.2_R | chr9:139393463-139393593 | GGAGGACCTCATCAACTCAC |
| NOTCH1.9.6.NOTCH1.1_F | chr9:139399102-139399247 | GAGAGTTGCGGGGATTGAC |
| NOTCH1.9.6.NOTCH1.1_R | chr9:139399102-139399247 | CGCAAGCACCCCATCAA |
| NOTCH1.9.10.NOTCH1.2_F | chr9:139399348-139399514 | ACGTTGGTGTGCAGCA |

| | | |
|---|---|---|
| NOTCH1.9.10.NOTCH1.2_R | chr9:139399348-139399514 | GACCACTTCAGCGACGG |
| NOTCH1.9.12.NOTCH1.3_F | chr9:139399508-139399663 | CTGTTGCAGCCCTGGTC |
| NOTCH1.9.12.NOTCH1.3_R | chr9:139399508-139399663 | CAGTTCTAAGGCTCTGCTCA |
| NOTCH1.9.12.NOTCH1.4_F | chr9:139399204-139399363 | AGCGAGGCCTTCACCT |
| NOTCH1.9.12.NOTCH1.4_R | chr9:139399204-139399363 | CAGCTCCTTCCACTTCCTG |
| NOTCH1.12.1.NOTCH1.1_F | chr9:139401267-139401431 | TGGACAGGCACTCGTTGA |
| NOTCH1.12.1.NOTCH1.1_R | chr9:139401267-139401431 | ACTGACGAAACCTGGCC |
| NOTCH1.12.10.NOTCH1.2_F | chr9:139401117-139401274 | GTAAGAGCAGGGCAGTGAG |
| NOTCH1.12.10.NOTCH1.2_R | chr9:139401117-139401274 | TGGGTGAGCGCTGTGA |
| NOTCH1.15.1.NOTCH1.1_F | chr9:139402684-139402842 | TGGGCACAGCAGGTTAC |
| NOTCH1.15.1.NOTCH1.1_R | chr9:139402684-139402842 | CAGCTGACCCCAATCTGTC |
| NOTCH1.8.1.NOTCH1.1_F | chr9:139397491-139397650 | GCGGAGTGCCATTCAGAAAA |
| NOTCH1.8.1.NOTCH1.1_R | chr9:139397491-139397650 | AGCCTCAACATCCCCTACAA |
| NOTCH1.8.2.NOTCH1.2_F | chr9:139397636-139397789 | GATGGGGCCACACTTACTC |
| NOTCH1.8.2.NOTCH1.2_R | chr9:139397636-139397789 | CAGCCCCTCTCTGATTGTC |
| NOTCH1.8.2.NOTCH1.3_F | chr9:139397379-139397537 | CCAACAAAGAAAATTCAGGAGG |
| NOTCH1.8.2.NOTCH1.3_R | chr9:139397379-139397537 | TTTGTGCCAGTTGCTACCC |
| NOTCH1.8.3.NOTCH1.4_F | chr9:139397219-139397378 | CAACTTCAAATCGCTGCACT |
| NOTCH1.8.3.NOTCH1.4_R | chr9:139397219-139397378 | GCTTCTCTCCCCACCCTTT |
| NOTCH1.4.1.NOTCH1.1_F | chr9:139395024-139395185 | GGAAGACACCTTGTGCGT |
| NOTCH1.4.1.NOTCH1.1_R | chr9:139395024-139395185 | GACTTCATCTACCAGGGCG |
| NOTCH1.4.2.NOTCH1.2_F | chr9:139395176-139395311 | TGCGGTCTGTCTGGTTG |
| NOTCH1.4.2.NOTCH1.2_R | chr9:139395176-139395311 | AGACTGAGCACCCGTCT |
| NOTCH1.4.2.NOTCH1.3_F | chr9:139394895-139395056 | ACCAGTTGTCCCGGACT |
| NOTCH1.4.2.NOTCH1.3_R | chr9:139394895-139395056 | CAACATCCAGGACAACATGG |
| NOTCH1.34.14.NOTCH1.1_F | chr9:139440140-139440342 | CCGCCAAAGTTTCCAAAGGG |
| NOTCH1.34.14.NOTCH1.1_R | chr9:139440140-139440342 | GGAAGAGAGGGCGGGACC |
| NOTCH1.19.1.NOTCH1.1_F | chr9:139405573-139405724 | AGACATCCTGACCTCCCATC |
| NOTCH1.19.1.NOTCH1.1_R | chr9:139405573-139405724 | TGTGTCTCCTTCCCGCA |
| NOTCH1.5.1.NOTCH1.1_F | chr9:139396230-139396367 | ACATTGACGTCCATGCAGT |
| NOTCH1.5.1.NOTCH1.1_R | chr9:139396230-139396367 | TCTGCTCACTCCCTCTATGT |
| NOTCH1.5.2.NOTCH1.2_F | chr9:139396103-139396264 | GCTCTCACCCCCAATTCTAA |
| NOTCH1.5.2.NOTCH1.2_R | chr9:139396103-139396264 | CTGCGCATGTCTGCCAT |
| FGFR1.11.1.FGFR1.1_F | chr8:38279174-38279335 | GGATTCAGCCCTCAAAGCT |
| FGFR1.11.1.FGFR1.1_R | chr8:38279174-38279335 | GGACTCTCCCATCACTCTG |
| FGFR1.11.2.FGFR1.2_F | chr8:38279326-38279469 | AGTGTGTACCTTCCAGAACG |
| FGFR1.11.2.FGFR1.2_R | chr8:38279326-38279469 | TGTCCATTTTGCTTCCGTTG |
| FGFR1.11.2.FGFR1.3_F | chr8:38279158-38279305 | TCTTCTCTGAGCTGAGAGGA |
| FGFR1.11.2.FGFR1.3_R | chr8:38279158-38279305 | CGTTCTGGAAGGTACACACT |
| FGFR1.7.1.FGFR1.1_F | chr8:38274789-38274946 | CAGCTCAGATCTTCTCCCC |
| FGFR1.7.1.FGFR1.1_R | chr8:38274789-38274946 | CCCAAGTAAATGAGTCTCAACG |
| FGFR1.2.4.FGFR1.1_F | chr8:38271423-38271566 | AGCTCTGGCTCTGGCA |
| FGFR1.2.4.FGFR1.1_R | chr8:38271423-38271566 | TATCCATCTCCAGGAGACGT |
| FGFR1.17.1.FGFR1.1_F | chr8:38297747-38297905 | TAAAAGGAGCACAGAACAGC |
| FGFR1.17.1.FGFR1.1_R | chr8:38297747-38297905 | ATTTTTGGGGCTCTCTCCAC |
| FGFR1.1.1.FGFR1.1_F | chr8:38271161-38271323 | AGTCAGCGGCGTTTGAG |
| FGFR1.1.1.FGFR1.1_R | chr8:38271161-38271323 | TTCCTCTCGCCCATCACA |
| FGFR1.1.2.FGFR1.2_F | chr8:38271114-38271273 | GGTGAGGGTTACAGCTGAC |
| FGFR1.1.2.FGFR1.2_R | chr8:38271114-38271273 | CAGTACTCCCCCAGCTTTC |
| FGFR1.18.1.FGFR1.1_F | chr8:38314924-38315068 | TGCAGAGTGTGGCTGTG |
| FGFR1.18.1.FGFR1.1_R | chr8:38314924-38315068 | CCCTCCTAACTTTTGGGGTT |
| FGFR1.18.2.FGFR1.2_F | chr8:38314812-38314970 | ACCTGCAACCATATCACCTC |
| FGFR1.18.2.FGFR1.2_R | chr8:38314812-38314970 | CACCAACCTCTAACTGCAGA |
| FGFR1.16.1.FGFR1.1_F | chr8:38287199-38287360 | CAGGGCTTGGCTACCAA |
| FGFR1.16.1.FGFR1.1_R | chr8:38287199-38287360 | GATGTGCAGAGCATCAACTG |
| FGFR1.16.2.FGFR1.2_F | chr8:38287344-38287505 | GTTGCTTTCCGCCAGCT |
| FGFR1.16.2.FGFR1.2_R | chr8:38287344-38287505 | TCTCCCTGTCTTCCTCTCTC |
| FGFR1.13.1.FGFR1.1_F | chr8:38283613-38283767 | GGCTCTTCCCACTAAACTCA |
| FGFR1.13.1.FGFR1.1_R | chr8:38283613-38283767 | TATCCCTTTGGCATTTCCCC |
| FGFR1.6.1.FGFR1.1_F | chr8:38273388-38273547 | GAGCCTTCAGGTTCCACAC |
| FGFR1.6.1.FGFR1.1_R | chr8:38273388-38273547 | GTCATCGTGGAGTATGCCTC |
| FGFR1.6.2.FGFR1.2_F | chr8:38273501-38273630 | GGGGTTGTAGCAGTATTCCA |
| FGFR1.6.2.FGFR1.2_R | chr8:38273501-38273630 | GGGTTTCTTTGAGGTGAAGC |
| FGFR1.3.1.FGFR1.1_F | chr8:38271670-38271828 | GACATCTCCTCGGGCTTAC |
| FGFR1.3.1.FGFR1.1_R | chr8:38271670-38271828 | GGAGAAGCTCTAACACCCTG |
| FGFR1.9.1.FGFR1.1_F | chr8:38275731-38275892 | TGCCTTCAAAAAGTTGGGAG |
| FGFR1.9.1.FGFR1.1_R | chr8:38275731-38275892 | ACCCCACTCTTCCTTGCA |

| | | |
|---|---|---|
| FGFR1.12.1.FGFR1.1_F | chr8:38281837-38282002 | CTGTTCCCAGCTCACCT |
| FGFR1.12.1.FGFR1.1_R | chr8:38281837-38282002 | TGGCACCAGTCTTCGTG |
| FGFR1.12.2.FGFR1.2_F | chr8:38282000-38282162 | GTCTCCTTCCCAGTAGACTG |
| FGFR1.12.2.FGFR1.2_R | chr8:38282000-38282162 | CCCGCCAACAAAACAGT |
| FGFR1.12.2.FGFR1.3_F | chr8:38281745-38281885 | GTCCAAATGCCTTCCTTGTG |
| FGFR1.12.2.FGFR1.3_R | chr8:38281745-38281885 | CACACACACTCCATCTCAGT |
| FGFR1.12.3.FGFR1.4_F | chr8:38282105-38282253 | ATGTGCTTTAGCCACTGGAT |
| FGFR1.12.3.FGFR1.4_R | chr8:38282105-38282253 | CTCTTTAGCCATGGCAAGGT |
| FGFR1.14.1.FGFR1.1_F | chr8:38285435-38285598 | CTTTCGCATGCACACACAC |
| FGFR1.14.1.FGFR1.1_R | chr8:38285435-38285598 | GCAGCCGTAGCTCCATA |
| FGFR1.14.2.FGFR1.2_F | chr8:38285462-38285618 | GTAGCCTCCAATTCTGTGGT |
| FGFR1.14.2.FGFR1.2_R | chr8:38285462-38285618 | CTGTCTCTCTTGGCTTTCCC |
| FGFR1.15.1.FGFR1.1_F | chr8:38285847-38286000 | GACCCAAAGGGCAGTAAGAT |
| FGFR1.15.1.FGFR1.1_R | chr8:38285847-38286000 | TGCACTAGCCTTGGTGAAAT |
| FGFR1.8.1.FGFR1.1_F | chr8:38275358-38275516 | TGCTTGGAATGGGACAAGAT |
| FGFR1.8.1.FGFR1.1_R | chr8:38275358-38275516 | TATGCCACTCTCTGTTTCCC |
| FGFR1.4.1.FGFR1.1_F | chr8:38271986-38272148 | TTTCTCTCTGGGGCAGAAAG |
| FGFR1.4.1.FGFR1.1_R | chr8:38271986-38272148 | CCCCTGTGCTGCTTTCA |
| FGFR1.10.1.FGFR1.1_F | chr8:38277081-38277240 | CGCAGAGGGATGCTCTT |
| FGFR1.10.1.FGFR1.1_R | chr8:38277081-38277240 | ACTCAGCCCTGGAAGAGAG |
| FGFR1.10.2.FGFR1.2_F | chr8:38277204-38277345 | GCCCCTGTGCAATAGATGAT |
| FGFR1.10.2.FGFR1.2_R | chr8:38277204-38277345 | CTCCCTAAAAGTTCCCAGGG |
| FGFR1.10.2.FGFR1.3_F | chr8:38277048-38277183 | GTGCAAATCCCCCATCTACT |
| FGFR1.10.2.FGFR1.3_R | chr8:38277048-38277183 | ATCATCTATTGCACAGGGGC |
| FGFR1.19.1.FGFR1.1_F | chr8:38318531-38318669 | CTAGGCTCCTGGGATTCATG |
| FGFR1.19.1.FGFR1.1_R | chr8:38318531-38318669 | CTCCAGTGAGAAGACAGCAG |
| FGFR1.5.1.FGFR1.1_F | chr8:38272272-38272421 | TCTCAGATGAAACCACCAGC |
| FGFR1.5.1.FGFR1.1_R | chr8:38272272-38272421 | CTCTCCCTTCCTCCTTCCTC |
| CYP2C9.8.1.CYP2C9.1_F | chr10:96745800-96745946 | TTGTTTCTAGGGCACAACCA |
| CYP2C9.8.1.CYP2C9.1_R | chr10:96745800-96745946 | ACCCTGAAACACAAATGGAA |
| CYP2C9.8.2.CYP2C9.2_F | chr10:96745675-96745808 | GGAAATGGTACTGCCCTTCT |
| CYP2C9.8.2.CYP2C9.2_R | chr10:96745675-96745808 | ATGTAGCACAGAAGTCAGGG |
| CYP2C9.6.1.CYP2C9.1_F | chr10:96731881-96732040 | GAAAAGCACAACCAACCATC |
| CYP2C9.6.1.CYP2C9.1_R | chr10:96731881-96732040 | GCAACACCTTCCCAAAATCT |
| CYP2C9.6.4.CYP2C9.2_F | chr10:96731770-96731914 | ACTTTCCAGTTTCTATGTTGGT |
| CYP2C9.6.4.CYP2C9.2_R | chr10:96731770-96731914 | CCAGCTCCAAACAAGTCAAC |
| CYP2C9.1.1.CYP2C9.1_F | chr10:96698450-96698607 | AGAAGGCTTCAATGGATTCTC |
| CYP2C9.1.1.CYP2C9.1_R | chr10:96698450-96698607 | CCACTGAAGGAGCATACTTAC |
| CYP2C9.1.2.CYP2C9.2_F | chr10:96698380-96698537 | ACACACCGAATTAGCATGGA |
| CYP2C9.1.2.CYP2C9.2_R | chr10:96698380-96698537 | CAATCACTGGGAGAGGAGTG |
| CYP2C9.4.1.CYP2C9.1_F | chr10:96707551-96707699 | TTTAGCCTCACCCTGTGATC |
| CYP2C9.4.1.CYP2C9.1_R | chr10:96707551-96707699 | GGAAGCAAAAATCTTGGCCT |
| CYP2C9.4.2.CYP2C9.2_F | chr10:96707421-96707576 | ATGCATGCCGAACTCTTTTT |
| CYP2C9.4.2.CYP2C9.2_R | chr10:96707421-96707576 | ATGGAGCAGATCACATTGCA |
| CYP2C9.3.1.CYP2C9.1_F | chr10:96701963-96702106 | CCTGTTAGGAATTGTTTTCAGC |
| CYP2C9.3.1.CYP2C9.1_R | chr10:96701963-96702106 | GGTCAGTGATATGGAGTAGGG |
| CYP2C9.3.2.CYP2C9.2_F | chr10:96701836-96701969 | GATGGGGAGGATGGAAAACA |
| CYP2C9.3.2.CYP2C9.2_R | chr10:96701836-96701969 | CCGGATCTCCTTCCATTTCT |
| CYP2C9.7.1.CYP2C9.1_F | chr10:96740980-96741139 | GAACGTGTGATTGGCAGAAA |
| CYP2C9.7.1.CYP2C9.1_R | chr10:96740980-96741139 | ATGGAGTTGCAGTGTAGGAG |
| CYP2C9.7.2.CYP2C9.2_F | chr10:96740890-96741033 | ACCCCTGAATTGCTACAACA |
| CYP2C9.7.2.CYP2C9.2_R | chr10:96740890-96741033 | GTATCTCTGGACCTCGTGC |
| CYP2C9.9.1.CYP2C9.1_F | chr10:96748623-96748783 | GGAAAACGGATTTGTGTGGG |
| CYP2C9.9.1.CYP2C9.1_R | chr10:96748623-96748783 | AGGCCATCTGCTCTTCTTC |
| CYP2C9.9.2.CYP2C9.2_F | chr10:96748775-96748923 | CTTCTACCAGCTGTGCTTCA |
| CYP2C9.9.2.CYP2C9.2_R | chr10:96748775-96748923 | TCCGTAATGGAGGTCGAATG |
| CYP2C9.9.2.CYP2C9.3_F | chr10:96748571-96748704 | ACTGTCACAGTTACGCATGA |
| CYP2C9.9.2.CYP2C9.3_R | chr10:96748571-96748704 | GGAGTGGTGTCAAGGTTCTT |
| CYP2C9.2.1.CYP2C9.1_F | chr10:96701625-96701783 | CTACTTTCCTAGCTCTCAAAGG |
| CYP2C9.2.1.CYP2C9.1_R | chr10:96701625-96701783 | CTGAAACAGGCACATGCAC |
| CYP2C9.2.2.CYP2C9.2_F | chr10:96701572-96701720 | TCAGGCTTAGCAAATGGACA |
| CYP2C9.2.2.CYP2C9.2_R | chr10:96701572-96701720 | GCCTCTTCCAGAAAACTCCT |
| CYP2C9.5.1.CYP2C9.1_F | chr10:96708905-96709058 | CCTATCATTGATTACTTCCCGG |
| CYP2C9.5.1.CYP2C9.1_R | chr10:96708905-96709058 | ACAAGCAGTCACATAACTAAGC |
| CYP2C9.5.3.CYP2C9.2_F | chr10:96708815-96708969 | CCAGAGCTTGGTATATGGTATG |
| CYP2C9.5.3.CYP2C9.2_R | chr10:96708815-96708969 | TGTCCATTGATTCTTGGTGTTC |
| MET.11.1.MET.1_F | chr7:116399461-116399620 | CAGTGGTGGGAGCACAATA |

| | | |
|---|---|---|
| MET.11.1.MET.1_R | chr7:116399461-116399620 | AAAGGCGAAACTGAGATGTC |
| MET.11.2.MET.2_F | chr7:116399583-116399715 | GGTTCTACTCAGAGCTCTGC |
| MET.11.2.MET.2_R | chr7:116399583-116399715 | TTTCCACATGGTTAGCCCAA |
| MET.11.2.MET.3_F | chr7:116399345-116399487 | TTGATGTTGACTGTGCCTCT |
| MET.11.2.MET.3_R | chr7:116399345-116399487 | ACCATTCTCGGGACACTAAC |
| MET.21.1.MET.1_F | chr7:116435694-116435851 | TCTCACCTCATCTGTCCTGT |
| MET.21.1.MET.1_R | chr7:116435694-116435851 | ACGTAAGAGGTACAGAAAGACT |
| MET.7.1.MET.1_F | chr7:116395418-116395576 | TGTCCTTGTAGGTTTTCCCA |
| MET.7.1.MET.1_R | chr7:116395418-116395576 | CCCCAGCAAAGCATTTTAAGA |
| MET.7.10.MET.2_F | chr7:116395266-116395417 | TGTGACCCTTTTCCCTTTAGT |
| MET.7.10.MET.2_R | chr7:116395266-116395417 | CCTTCAAGGGGTGCACTATT |
| MET.17.1.MET.1_F | chr7:116417443-116417594 | CCAAGTTCTTTCTTTTGCACAG |
| MET.17.1.MET.1_R | chr7:116417443-116417594 | GGGGAAAGTGTAAATCAACGT |
| MET.2.1.MET.1_F | chr7:116339125-116339284 | CCTTGAACCTGTTTTGGCAG |
| MET.2.1.MET.1_R | chr7:116339125-116339284 | TGACATTCTGGATGGGTGTT |
| MET.2.2.MET.2_F | chr7:116339395-116339555 | AGAAGGTTGCTGAGTACAAGA |
| MET.2.2.MET.2_R | chr7:116339395-116339555 | ACATGTCGCTGGCAGGT |
| MET.2.3.MET.3_F | chr7:116339280-116339409 | ATCAGCTTCCCAACTTCACC |
| MET.2.3.MET.3_R | chr7:116339280-116339409 | TGGGAAACAATCTGGGTGTT |
| MET.2.3.MET.4_F | chr7:116339596-116339755 | TCCCCACAATCATACTGCTG |
| MET.2.3.MET.4_R | chr7:116339596-116339755 | AATGCAATGGATGATCTGGG |
| MET.2.4.MET.5_F | chr7:116339542-116339701 | TCAACTCATTAGCTGTGGCA |
| MET.2.4.MET.5_R | chr7:116339542-116339701 | AGTTGATGAACCGGTCCTTT |
| MET.2.4.MET.6_F | chr7:116339846-116340005 | ACGGACCAGTCCTACATTGA |
| MET.2.4.MET.6_R | chr7:116339846-116340005 | AGAGGCATTTCCATGTAGGA |
| MET.2.5.MET.7_F | chr7:116340005-116340165 | GTTCCATAAACTCTGGATTGCA |
| MET.2.5.MET.7_R | chr7:116340005-116340165 | TTGTGCGAACACCCCGA |
| MET.2.5.MET.8_F | chr7:116339725-116339883 | GGACCGGTTCATCAACTTCT |
| MET.2.5.MET.8_R | chr7:116339725-116339883 | GCTTTCAAAGGCATGGACAT |
| MET.2.6.MET.9_F | chr7:116340179-116340340 | TCTTTTCGGGGTGTTCGC |
| MET.2.6.MET.9_R | chr7:116340179-116340340 | GTGGGGAACTGATGTGACTT |
| MET.2.7.MET.10_F | chr7:116340034-116340189 | GGAAATGCCTCTGGAGTGTA |
| MET.2.7.MET.10_R | chr7:116340034-116340189 | CATTGGTTCGGCAGAATCTG |
| MET.22.1.MET.1_F | chr7:116435970-116436131 | ATGCTAAAATGCTGGCACC |
| MET.22.1.MET.1_R | chr7:116435970-116436131 | CGTGTGTCCACCTCATCAT |
| MET.22.2.MET.2_F | chr7:116436072-116436203 | TGTCCATGTGAACGCTACTT |
| MET.22.2.MET.2_R | chr7:116436072-116436203 | ACCATTGGACAAAGTGTGGA |
| MET.22.2.MET.3_F | chr7:116435904-116436051 | TACAGAAATGCCTGCCTTCA |
| MET.22.2.MET.3_R | chr7:116435904-116436051 | AAGTAGCGTTCACATGGACA |
| MET.1.1.MET.1_F | chr7:116335808-116335965 | ACTTCCTTCTTCACAGGGTG |
| MET.1.1.MET.1_R | chr7:116335808-116335965 | CACTGGGGGTACAGGTTATG |
| MET.18.1.MET.1_F | chr7:116418858-116419014 | CTGACATAGGAGAAGTTTCCCA |
| MET.18.1.MET.1_R | chr7:116418858-116419014 | AGTAAGCTTGGCAGTCAACT |
| MET.18.2.MET.2_F | chr7:116418725-116418862 | GTCAAACCCTCAGGACAAGA |
| MET.18.2.MET.2_R | chr7:116418725-116418862 | CATGATGATTCCCTCGGTCA |
| MET.13.1.MET.1_F | chr7:116409799-116409954 | TTCTGAAGCCGTTTTATGCA |
| MET.13.1.MET.1_R | chr7:116409799-116409954 | GAATGCAGGCTGAGTTGATG |
| MET.13.2.MET.2_F | chr7:116409666-116409815 | TTCCTGCAGAACTGTGAAGT |
| MET.13.2.MET.2_R | chr7:116409666-116409815 | AGCTCGCTGTTCAATTTCAG |
| MET.16.1.MET.1_F | chr7:116415061-116415218 | CCATTACTGCAAAATACTGTCC |
| MET.16.1.MET.1_R | chr7:116415061-116415218 | AATCTGCAAAGGCCAAAGAT |
| MET.16.8.MET.2_F | chr7:116414896-116415053 | TCAGTCCCCATTAAATGAGGT |
| MET.16.8.MET.2_R | chr7:116414896-116415053 | CTGAGGTCAATGTGGACAGT |
| MET.16.9.MET.3_F | chr7:116414970-116415101 | CATCTCAGAACGGTTCATGC |
| MET.16.9.MET.3_R | chr7:116414970-116415101 | CCAATCACTACATGCTGCAC |
| MET.6.1.MET.1_F | chr7:116381056-116381215 | CGGGACATGGACTCAACAG |
| MET.6.1.MET.1_R | chr7:116381056-116381215 | AGCTGATGACTCACAGCTAA |
| MET.6.2.MET.2_F | chr7:116380904-116381058 | TTTCCACCCCTTCTCTTCAC |
| MET.6.2.MET.2_R | chr7:116380904-116381058 | TTGTAGATTGCAGGCAGACA |
| MET.3.1.MET.1_F | chr7:116364766-116364903 | GCAAGGGAAGCTTTTGGAAG |
| MET.3.1.MET.1_R | chr7:116364766-116364903 | AGGCACTGAGTGTTCACATT |
| MET.9.1.MET.1_F | chr7:116397740-116397899 | AATACGGTCCTATGGCTGGT |
| MET.9.1.MET.1_R | chr7:116397740-116397899 | CCACACACACACACAAAACA |
| MET.9.2.MET.2_F | chr7:116397604-116397759 | TTCTCCTATGTGGTAAGGAAGA |
| MET.9.2.MET.2_R | chr7:116397604-116397759 | CCCACTGTTTAGGTAATTTCCA |
| MET.12.8.MET.1_F | chr7:116403070-116403203 | TGGATGTTGCCAAGCTGTAT |
| MET.12.8.MET.1_R | chr7:116403070-116403203 | TGGAAAGGATCCCATCTAACA |

| | | |
|---|---|---|
| MET.12.11.MET.2_F | chr7:116403233-116403388 | TGGGATCCTTTCCAAATACTTT |
| MET.12.11.MET.2_R | chr7:116403233-116403388 | GGCCAAGTACAACAATTGTATT |
| MET.12.12.MET.3_F | chr7:116403120-116403279 | CTAGGCATGTCAACATCGCT |
| MET.12.12.MET.3_R | chr7:116403120-116403279 | TGCCCATTGAGATCATCACT |
| MET.14.8.MET.1_F | chr7:116411534-116411678 | CATGGCTAAATGCTGACTTTTC |
| MET.14.8.MET.1_R | chr7:116411534-116411678 | GCTTTCTCTTTTTCAGCCACA |
| MET.14.9.MET.2_F | chr7:116411633-116411778 | TCACAGGATTGATTGCTGGT |
| MET.14.9.MET.2_R | chr7:116411633-116411778 | AGACAGCACACAAGAATCGA |
| MET.20.1.MET.1_F | chr7:116423378-116423532 | AGGCTGGATGAAAAATTCACAG |
| MET.20.1.MET.1_R | chr7:116423378-116423532 | AGGAGAAACTCAGAGATAACCA |
| MET.20.2.MET.2_F | chr7:116423245-116423383 | AGGCCAGATGAAATACTTCCT |
| MET.20.2.MET.2_R | chr7:116423245-116423383 | GGCAAGACCAAAATCAGCAA |
| MET.15.1.MET.1_F | chr7:116411922-116412078 | AGATCTGGGCAGTGAATTAGT |
| MET.15.1.MET.1_R | chr7:116411922-116412078 | ACAACCCACTGAGGTATATGT |
| MET.15.2.MET.2_F | chr7:116411799-116411953 | TCGATTCTTGTGTGCTGTCT |
| MET.15.2.MET.2_R | chr7:116411799-116411953 | GGCACTTACAAGCCTATCCA |
| MET.8.1.MET.1_F | chr7:116397511-116397667 | GATTGAAATGCACAGTTGGTC |
| MET.8.1.MET.1_R | chr7:116397511-116397667 | GGAGGGGAGATAAAAACAAAAC |
| MET.8.2.MET.2_F | chr7:116397408-116397544 | GCAGTCAGCTCACCATTTAG |
| MET.8.2.MET.2_R | chr7:116397408-116397544 | TCCCGTGGCCATTTGAAATA |
| MET.4.1.MET.1_F | chr7:116371772-116371932 | GCGCCGTGATGAATATCG |
| MET.4.1.MET.1_R | chr7:116371772-116371932 | CCAGATAGAACAGACACAGCT |
| MET.4.2.MET.2_F | chr7:116371643-116371796 | CCATTATCCTCCAGGCTCTG |
| MET.4.2.MET.2_R | chr7:116371643-116371796 | CCCATGAATAAGTCAACGCG |
| MET.19.1.MET.1_F | chr7:116422042-116422187 | TTCTAACTCTCTTTGACTGCAG |
| MET.19.1.MET.1_R | chr7:116422042-116422187 | ACAGATTCCTCCTTGTCACT |
| MET.10.1.MET.1_F | chr7:116398526-116398677 | TGTTCAGTGTGTCAAACAGT |
| MET.10.1.MET.1_R | chr7:116398526-116398677 | ACCCATCAGAAGCTTCTACT |
| MET.10.2.MET.2_F | chr7:116398402-116398531 | AGAAAAGGCTTCCACTCAGG |
| MET.10.2.MET.2_R | chr7:116398402-116398531 | TGGGCTGGGGTATAACATTC |
| MET.5.1.MET.1_F | chr7:116380014-116380168 | TGCTGTTTAGGTTGTGGTTT |
| MET.5.1.MET.1_R | chr7:116380014-116380168 | GCATTTGGGAAAAACCACGT |
| MET.5.2.MET.2_F | chr7:116379921-116380059 | ACAAGCCCTGCTAATCTGTT |
| MET.5.2.MET.2_R | chr7:116379921-116380059 | CTTCTGGAGACACTGGATGG |
| CHEK2.6.1.CHEK2.1_F | chr22:29092881-29093039 | AGCCACATACAGAATGCCAA |
| CHEK2.6.1.CHEK2.1_R | chr22:29092881-29093039 | TGTGAGATGTGTGTGTTGGT |
| CHEK2.11.8.CHEK2.1_F | chr22:29115337-29115477 | TGAGAAACCACCAATCACAAA |
| CHEK2.11.8.CHEK2.1_R | chr22:29115337-29115477 | ACCCATTTCTACTCTTTTCTTCC |
| CHEK2.3.9.CHEK2.1_F | chr22:29089989-29090214 | CAGCAGGGCTTCCCATGTAT |
| CHEK2.3.9.CHEK2.1_R | chr22:29089989-29090214 | TGTCTGCTGACTCCGTGATG |
| CHEK2.7.1.CHEK2.1_F | chr22:29095808-29095964 | GAAAGGCAGCTGTCAAAAGA |
| CHEK2.7.1.CHEK2.1_R | chr22:29095808-29095964 | TTCTGTCCAAGTGCGTTTTC |
| CHEK2.9.9.CHEK2.1_F | chr22:29105909-29106125 | CCACCACACCTGGCCAATAT |
| CHEK2.9.9.CHEK2.1_R | chr22:29105909-29106125 | CTCAGGCAGCCTTGAGTCAA |
| CHEK2.12.6.CHEK2.1_F | chr22:29120929-29121083 | CCTCCTATGAGAGAGTGGAAA |
| CHEK2.12.6.CHEK2.1_R | chr22:29120929-29121083 | GGGTCCTAAAAACTCTTACATTGC |
| CHEK2.12.7.CHEK2.2_F | chr22:29121076-29121224 | TCCATTGCCACTGTGATCTT |
| CHEK2.12.7.CHEK2.2_R | chr22:29121076-29121224 | TTCGGATTTTCAGGGTAGGT |
| CHEK2.2.1.CHEK2.1_F | chr22:29085095-29085254 | GCTCCTTAAGCCCAGACTAC |
| CHEK2.2.1.CHEK2.1_R | chr22:29085095-29085254 | TGCTAGCCCTGTCATTCTAG |
| CHEK2.14.1.CHEK2.1_F | chr22:29126402-29126554 | GGAAAGACCCACAGCTAACA |
| CHEK2.14.1.CHEK2.1_R | chr22:29126402-29126554 | CGTGGCTGGCCTAACTTTTT |
| CHEK2.15.1.CHEK2.1_F | chr22:29130411-29130570 | CCAAGATTGGCAAATCCATCC |
| CHEK2.15.1.CHEK2.1_R | chr22:29130411-29130570 | TCCTCTACCAGCACGATGC |
| CHEK2.15.2.CHEK2.2_F | chr22:29130571-29130727 | GAGGACTGGCTGGAGTTTG |
| CHEK2.15.2.CHEK2.2_R | chr22:29130571-29130727 | TCACCTTTGTTGTTGGACAC |
| CHEK2.15.2.CHEK2.3_F | chr22:29130324-29130466 | TCCAATCAGAACCTTCCACC |
| CHEK2.15.2.CHEK2.3_R | chr22:29130324-29130466 | CTGAGGACCAAGAACCTGAG |
| CHEK2.8.1.CHEK2.1_F | chr22:29099425-29099584 | TGAGAAAGGCAAGCCTACAT |
| CHEK2.8.1.CHEK2.1_R | chr22:29099425-29099584 | CCTCTTGGGAGTTTCTCACT |
| CHEK2.1.5.CHEK2.1_F | chr22:29083881-29084039 | TCTTTCGTGTTCAAACCACG |
| CHEK2.1.5.CHEK2.1_R | chr22:29083881-29084039 | ACTGGAAGCATATTGAGGAAC |
| CHEK2.4.3.CHEK2.1_F | chr22:29091076-29091220 | CACCACAGCACATACACATT |
| CHEK2.4.3.CHEK2.1_R | chr22:29091076-29091220 | ACCAATATTAAGCCTTAGTGGG |
| CHEK2.4.4.CHEK2.2_F | chr22:29091220-29091379 | ATGCTCAGAGAAAGGTGGAT |
| CHEK2.4.4.CHEK2.2_R | chr22:29091220-29091379 | GTGCTGGGATTACAAGCCTA |
| CHEK2.10.1.CHEK2.1_F | chr22:29107891-29108048 | GGTGATCAGCCTTTTATTGGT |

| | | |
|---|---|---|
| CHEK2.10.1.CHEK2.1_R | chr22:29107891-29108048 | CAGGAGTGGTAGGTCTCATAA |
| CHEK2.13.1.CHEK2.1_F | chr22:29121218-29121372 | AGCTCTCCTAGATACATGGGT |
| CHEK2.13.1.CHEK2.1_R | chr22:29121218-29121372 | ATTCAACAGCCCTCTGATGC |
| CHEK2.5.1.CHEK2.1_F | chr22:29091738-29091879 | TAAACTCCAGCAGTCCACAG |
| CHEK2.5.1.CHEK2.1_R | chr22:29091738-29091879 | TGGCAAGTTCAACATTATTCCC |
| CHEK2.5.2.CHEK2.2_F | chr22:29091612-29091751 | CAAGATCAAGCCACTGCATG |
| CHEK2.5.2.CHEK2.2_R | chr22:29091612-29091751 | CTTGTTTCTGTTGGGACTGC |
| VKORC1.1.1.VKORC1.1_F | chr16:31102451-31102614 | AGCCTGGCTTGGGTTGA |
| VKORC1.1.1.VKORC1.1_R | chr16:31102451-31102614 | ATGCTGCTGAGCTCCCT |
| VKORC1.1.2.VKORC1.2_F | chr16:31102582-31102732 | AGAGCACGAAGAACAGGATC |
| VKORC1.1.2.VKORC1.2_R | chr16:31102582-31102732 | CTGGCATCTAGGTAGTGCAG |
| VKORC1.4.1.VKORC1.1_F | chr16:31105888-31106047 | TGCACACCTGGAGGAGA |
| VKORC1.4.1.VKORC1.1_R | chr16:31105888-31106047 | GGCGGAACCTGGAGATAATG |
| VKORC1.4.2.VKORC1.2_F | chr16:31106014-31106176 | CGTCAGGCAAAGAGCGA |
| VKORC1.4.2.VKORC1.2_R | chr16:31106014-31106176 | TGGAACAGCCATTTGGGTC |
| VKORC1.4.2.VKORC1.3_F | chr16:31105836-31105974 | TAATCATCTGGCATCCTGGC |
| VKORC1.4.2.VKORC1.3_R | chr16:31105836-31105974 | CTTAGTGCTCTCGCTCTACG |
| VKORC1.3.1.VKORC1.1_F | chr16:31104641-31104770 | GGAGCCACTCACCTAACAAT |
| VKORC1.3.1.VKORC1.1_R | chr16:31104641-31104770 | GGGTTGACAGTCCTAACCTG |
| VKORC1.3.2.VKORC1.2_F | chr16:31104488-31104647 | CCTGTTAGTTACCTCCCCAC |
| VKORC1.3.2.VKORC1.2_R | chr16:31104488-31104647 | CGGTTGCATCTTCTACACAC |
| VKORC1.2.1.VKORC1.1_F | chr16:31104082-31104212 | TTTTCCACAAAACTGGGGTG |
| VKORC1.2.1.VKORC1.1_R | chr16:31104082-31104212 | CACCACTATGCCCAGCTAAT |
| MLH1.11.1.MLH1.1_F | chr3:37061819-37061961 | ACAGTTTAGAAATCAGTCCCCA |
| MLH1.11.1.MLH1.1_R | chr3:37061819-37061961 | AAGTAGCTGGATGAGAAGCG |
| MLH1.11.2.MLH1.2_F | chr3:37061702-37061839 | AGGTTTTGACCACTGTGTCA |
| MLH1.11.2.MLH1.2_R | chr3:37061702-37061839 | GAACTTCATGCTTTGTGGGG |
| MLH1.7.1.MLH1.1_F | chr3:37053266-37053417 | GGGCTCTGACATCTAGTGTG |
| MLH1.7.1.MLH1.1_R | chr3:37053266-37053417 | ACCTTATCTCCACCAGCAAA |
| MLH1.17.1.MLH1.1_F | chr3:37090006-37090163 | TTGTCCTTTTTCCTGCAAGC |
| MLH1.17.1.MLH1.1_R | chr3:37090006-37090163 | CCAGATCAAAGGGTGGTCAT |
| MLH1.2.1.MLH1.1_F | chr3:37038082-37038238 | ACATTAGAGTAGTTGCAGACTG |
| MLH1.2.1.MLH1.1_R | chr3:37038082-37038238 | AGGTCCTGACTCTTCCATGA |
| MLH1.1.1.MLH1.1_F | chr3:37035026-37035182 | TCTAGACGTTTCCTTGGCTC |
| MLH1.1.1.MLH1.1_R | chr3:37035026-37035182 | CCGTTAAGTCGTAGCCCTTA |
| MLH1.18.1.MLH1.1_F | chr3:37090404-37090537 | CTTTGGACCAGGTGAATTGG |
| MLH1.18.1.MLH1.1_R | chr3:37090404-37090537 | ATGAGGTCCTGTCCTAGTCC |
| MLH1.18.2.MLH1.2_F | chr3:37090270-37090426 | AGCAATATTCAGCAGTCCCA |
| MLH1.18.2.MLH1.2_R | chr3:37090270-37090426 | CGCATTCTTTACTGAGGCTT |
| MLH1.13.1.MLH1.1_F | chr3:37070291-37070450 | GCAGAAAGAGACATCGGGAA |
| MLH1.13.1.MLH1.1_R | chr3:37070291-37070450 | CAGTTGAGGCCCTATGCAT |
| MLH1.13.2.MLH1.2_F | chr3:37070191-37070322 | CAGCACAGAGAAGTTGCTTG |
| MLH1.13.2.MLH1.2_R | chr3:37070191-37070322 | CTGCAGTCATTTCCTTTCGG |
| MLH1.16.1.MLH1.1_F | chr3:37089010-37089169 | TCATGTTCTTGCTTCTTCCTAG |
| MLH1.16.1.MLH1.1_R | chr3:37089010-37089169 | ATGGCTGTCACACCTCAT |
| MLH1.16.2.MLH1.2_F | chr3:37089153-37089312 | GCTGAGATGCTTGCAGACTA |
| MLH1.16.2.MLH1.2_R | chr3:37089153-37089312 | GGTTTCATCATGTTGGCCAG |
| MLH1.6.1.MLH1.1_F | chr3:37050250-37050404 | CTTTTGCCAGGACATCTTGG |
| MLH1.6.1.MLH1.1_R | chr3:37050250-37050404 | AGACCCACTCCCAGATTTTG |
| MLH1.3.6.MLH1.1_F | chr3:37042415-37042565 | GAGATTTGGAAAAATGAGTAACATG |
| MLH1.3.6.MLH1.1_R | chr3:37042415-37042565 | CATCACAGGAGGATATTTTACACA |
| MLH1.9.6.MLH1.1_F | chr3:37055918-37056054 | TCAAAAGCTTCAGAATCTCTTTTCT |
| MLH1.9.6.MLH1.1_R | chr3:37055918-37056054 | CCTGTGAGTGGATTTCCCAT |
| MLH1.12.1.MLH1.1_F | chr3:37067148-37067311 | CTTTGCTACCAGGACTTGC |
| MLH1.12.1.MLH1.1_R | chr3:37067148-37067311 | TGTGACAATGGCCTGGG |
| MLH1.12.2.MLH1.2_F | chr3:37067311-37067471 | CAAACCCCTGTCCAGTCAG |
| MLH1.12.2.MLH1.2_R | chr3:37067311-37067471 | TGCTGGAAGTAGGTCCTCTC |
| MLH1.12.2.MLH1.3_F | chr3:37067038-37067176 | TAGTACTGCTCCATTTGGGG |
| MLH1.12.2.MLH1.3_R | chr3:37067038-37067176 | AGACGAGGTCAGACTTGTTG |
| MLH1.12.3.MLH1.4_F | chr3:37067424-37067580 | GAAGTGGCTGCCAAAAATCA |
| MLH1.12.3.MLH1.4_R | chr3:37067424-37067580 | AGGCAGAGAGAAGATGCAAG |
| MLH1.14.1.MLH1.1_F | chr3:37081632-37081790 | AATGAAGTGGGGTTGGTAGG |
| MLH1.14.1.MLH1.1_R | chr3:37081632-37081790 | TTGTTCACACACTCAGCTGA |
| MLH1.20.1.MLH1.1_F | chr3:37107060-37107191 | AAACCGATGTGTATAGGCCC |
| MLH1.20.1.MLH1.1_R | chr3:37107060-37107191 | GCATAAGGGCTGAGACTCAA |
| MLH1.15.4.MLH1.1_F | chr3:37083712-37083862 | GTCCCAACTGGTTGTATCTCA |
| MLH1.15.4.MLH1.1_R | chr3:37083712-37083862 | GGAGAGCTACTATTTTCAGAAACG |

| | | |
|---|---|---|
| MLH1.8.5.MLH1.1_F | chr3:37053492-37053643 | TCCTTGTGTCTTCTGCTGTT |
| MLH1.8.5.MLH1.1_R | chr3:37053492-37053643 | GGTTCCAAAATAATGTGATGGAATG |
| MLH1.4.1.MLH1.1_F | chr3:37045865-37046014 | ACCCAGCAGTGAGTTTTTCT |
| MLH1.4.1.MLH1.1_R | chr3:37045865-37046014 | ACTCTGAGACCTAGGCAAAA |
| MLH1.10.1.MLH1.1_F | chr3:37058971-37059106 | CCCCTCAGGACAGTTTTGAA |
| MLH1.10.1.MLH1.1_R | chr3:37058971-37059106 | TGTGAGTCTTGGTTGAGGAG |
| MLH1.19.1.MLH1.1_F | chr3:37091997-37092156 | TGAAGTGCCTGGCTCCAT |
| MLH1.19.1.MLH1.1_R | chr3:37091997-37092156 | AACACATCCCACAGTGCAT |
| MLH1.19.2.MLH1.2_F | chr3:37092105-37092241 | GGAAATATCCTGCAGCTTGC |
| MLH1.19.2.MLH1.2_R | chr3:37092105-37092241 | AGTCTTAAGTGCTACCAACACT |
| MLH1.19.2.MLH1.3_F | chr3:37091937-37092084 | GACACCAGTGTATGTTGGGA |
| MLH1.19.2.MLH1.3_R | chr3:37091937-37092084 | GCAAGCTGCAGGATATTTCC |
| MLH1.5.1.MLH1.1_F | chr3:37048431-37048568 | TTTTCTCTTTTCCCCTTGGG |
| MLH1.5.1.MLH1.1_R | chr3:37048431-37048568 | GCTTCAACAATTTACTCTCCCA |
| GATA3.1.6.GATA3.1_F | chr10:8097629-8097795 | CGAGGCCATGGAGGTGA |
| GATA3.1.6.GATA3.1_R | chr10:8097629-8097795 | CGTAGTAGGGCGGGAC |
| GATA3.1.7.GATA3.2_F | chr10:8097786-8097941 | GCTTTTTAACATCGACGGTCA |
| GATA3.1.7.GATA3.2_R | chr10:8097786-8097941 | CTTTCGCTTGACCTCTCCC |
| GATA3.1.8.GATA3.3_F | chr10:8097510-8097670 | CCGAAAGCAAATCATTCAACG |
| GATA3.1.8.GATA3.3_R | chr10:8097510-8097670 | TGCTGCCCGTTGAGCA |
| GATA3.4.1.GATA3.1_F | chr10:8111433-8111569 | TCTCCTCTCTCCCCACTCT |
| GATA3.4.1.GATA3.1_R | chr10:8111433-8111569 | CTGTTCTTGCTGATCCCAGT |
| GATA3.3.1.GATA3.1_F | chr10:8105950-8106102 | GTGGCTTATCTGTGCTTTTGT |
| GATA3.3.1.GATA3.1_R | chr10:8105950-8106102 | CATCCCTTCCCGAGAACTTA |
| GATA3.2.1.GATA3.1_F | chr10:8100307-8100467 | CCTCTGCTTCATGGATCCC |
| GATA3.2.1.GATA3.1_R | chr10:8100307-8100467 | AAGGTGAAGAGGTGCGG |
| GATA3.2.2.GATA3.2_F | chr10:8100243-8100372 | ACTCACCCTCCTTCTCTCTC |
| GATA3.2.2.GATA3.2_R | chr10:8100243-8100372 | GATGGACGTCTTGGAGAAGG |
| GATA3.2.3.GATA3.3_F | chr10:8100596-8100760 | AAGTACCAGGTGCCCCT |
| GATA3.2.3.GATA3.3_R | chr10:8100596-8100760 | ACTTGCATCCGAAGCCG |
| GATA3.2.4.GATA3.4_F | chr10:8100715-8100847 | CTACGTGCCCGAGTACA |
| GATA3.2.4.GATA3.4_R | chr10:8100715-8100847 | CCCTGGATTGAGGAAAAGGG |
| GATA3.2.5.GATA3.5_F | chr10:8100451-8100604 | CCTCGTCCTCCTCCTTGT |
| GATA3.2.5.GATA3.5_R | chr10:8100451-8100604 | GGACGACTCCAGCTTCATG |
| GATA3.5.1.GATA3.1_F | chr10:8115722-8115881 | AGATTAACAGACCCCTGACTAT |
| GATA3.5.1.GATA3.1_R | chr10:8115722-8115881 | ATGTGGCTGGAGTGGCT |
| GATA3.5.2.GATA3.2_F | chr10:8115865-8116025 | AGACACATGTCCTCCCTGA |
| GATA3.5.2.GATA3.2_R | chr10:8115865-8116025 | AAGTCGAAAGGGACTGCAG |
| GATA3.5.2.GATA3.3_F | chr10:8115593-8115722 | GCCAGCTGAAATGGAAACAG |
| GATA3.5.2.GATA3.3_R | chr10:8115593-8115722 | GTCTGGATGCCTTCCTTCTT |
| PIK3CA.11.9.PIK3CA.1_F | chr3:178937326-178937481 | CATTCACAACTATCTTTCCCCT |
| PIK3CA.11.9.PIK3CA.1_R | chr3:178937326-178937481 | ACTGAGAAAGTTTGTCATCTGT |
| PIK3CA.11.10.PIK3CA.2_F | chr3:178937476-178937603 | CTGTTCGGTGCTTGGAAAAA |
| PIK3CA.11.10.PIK3CA.2_R | chr3:178937476-178937603 | GCATAAAACTAGTTAGTGCAGTAGG |
| PIK3CA.7.1.PIK3CA.1_F | chr3:178928002-178928161 | TCCATTGGCATGGGGAAATA |
| PIK3CA.7.1.PIK3CA.1_R | chr3:178928002-178928161 | AGAGAAGGTTTGACTGCCAT |
| PIK3CA.7.2.PIK3CA.2_F | chr3:178927920-178928053 | CCTTTTGGGGAAGAAAAGTGT |
| PIK3CA.7.2.PIK3CA.2_R | chr3:178927920-178928053 | CATGAGGTACTGGCCAAAGA |
| PIK3CA.17.1.PIK3CA.1_F | chr3:178947130-178947289 | GGTGGTGCGAAATTCTCAC |
| PIK3CA.17.1.PIK3CA.1_R | chr3:178947130-178947289 | TCAGAGGAATACACAAACACC |
| PIK3CA.17.2.PIK3CA.2_F | chr3:178947060-178947194 | AACGTGTTACTCCTCTTTCAG |
| PIK3CA.17.2.PIK3CA.2_R | chr3:178947060-178947194 | TGTCTTTGAGCCACTGATGT |
| PIK3CA.2.1.PIK3CA.1_F | chr3:178917577-178917724 | CTGAACGTTTGTAAAGAAGCTG |
| PIK3CA.2.1.PIK3CA.1_R | chr3:178917577-178917724 | ACACAGGTAGAAGACTGCAC |
| PIK3CA.2.8.PIK3CA.2_F | chr3:178917441-178917585 | ACATGTTCATGCTGTGTATGT |
| PIK3CA.2.8.PIK3CA.2_R | chr3:178917441-178917585 | ATGAGGTGAATTGAGGTCCC |
| PIK3CA.1.1.PIK3CA.1_F | chr3:178916674-178916833 | ACTTGATGCCCCCAAGAATC |
| PIK3CA.1.1.PIK3CA.1_R | chr3:178916674-178916833 | TTCCCTTTCTGCTTCTTGAG |
| PIK3CA.1.2.PIK3CA.2_F | chr3:178916555-178916711 | TAGGTTTCTGCTTTGGGACA |
| PIK3CA.1.2.PIK3CA.2_R | chr3:178916555-178916711 | CCTCACGGAGGCATTCTAAA |
| PIK3CA.1.2.PIK3CA.3_F | chr3:178916853-178917006 | ACTCAAGAAGCAGAAAGGGA |
| PIK3CA.1.2.PIK3CA.3_R | chr3:178916853-178917006 | GAAAGGGACAACAGTTAAGCT |
| PIK3CA.1.3.PIK3CA.4_F | chr3:178916789-178916924 | AGCAAGAAAATACCCCCTCC |
| PIK3CA.1.3.PIK3CA.4_R | chr3:178916789-178916924 | TTTCTTCACGGTTGCCTACT |
| PIK3CA.18.4.PIK3CA.1_F | chr3:178947753-178947899 | TCAAGTTGGCCTGAATCACT |
| PIK3CA.18.4.PIK3CA.1_R | chr3:178947753-178947899 | GAAAACCATTACTTGTCCATCG |
| PIK3CA.18.5.PIK3CA.2_F | chr3:178947878-178948029 | GGGAATTGGAGATCGTCACA |

| | | |
|---|---|---|
| PIK3CA.18.5.PIK3CA.2_R | chr3:178947878-178948029 | CTTGTGATCCAAAAAGTGTCCA |
| PIK3CA.16.1.PIK3CA.1_F | chr3:178943710-178943843 | CGTGATCCCCAAATTTGCAT |
| PIK3CA.16.1.PIK3CA.1_R | chr3:178943710-178943843 | ACTTTCAACATACAGGTTGCC |
| PIK3CA.13.1.PIK3CA.1_F | chr3:178938813-178938970 | ACAGTTAGCCAGAGGTTTGG |
| PIK3CA.13.1.PIK3CA.1_R | chr3:178938813-178938970 | CCACTGCAGTGAAAAGAGTC |
| PIK3CA.13.14.PIK3CA.2_F | chr3:178938620-178938866 | CCTGAAACTCATGGTGGTTTTGT |
| PIK3CA.13.14.PIK3CA.2_R | chr3:178938620-178938866 | CCATTGCCTCGACTTGCCTA |
| PIK3CA.6.13.PIK3CA.1_F | chr3:178927355-178927498 | CTTTGATGAAGACTTTTCTTGATGT |
| PIK3CA.6.13.PIK3CA.1_R | chr3:178927355-178927498 | AGGTAAACATAATTGTTCCTTCTGA |
| PIK3CA.3.1.PIK3CA.1_F | chr3:178919222-178919374 | ACTCGAAGTATGTTGCTATCCT |
| PIK3CA.3.1.PIK3CA.1_R | chr3:178919222-178919374 | ACAGATACTCATCCTCAATGTG |
| PIK3CA.3.4.PIK3CA.2_F | chr3:178919060-178919209 | AGAGAGATGGTGATTGCATCT |
| PIK3CA.3.4.PIK3CA.2_R | chr3:178919060-178919209 | AGTTGTTCAGAGGATAGCAACA |
| PIK3CA.3.5.PIK3CA.3_F | chr3:178919167-178919309 | TCAACCATGACTGTGTACCA |
| PIK3CA.3.5.PIK3CA.3_R | chr3:178919167-178919309 | CACCTTATACTGACTCAGAGGA |
| PIK3CA.9.6.PIK3CA.1_F | chr3:178935963-178936112 | TCCAGAGGGGAAAAATATGACA |
| PIK3CA.9.6.PIK3CA.1_R | chr3:178935963-178936112 | TAGCACTTACCTGTGACTCC |
| PIK3CA.9.7.PIK3CA.2_F | chr3:178936050-178936176 | AGGGAAAATGACAAAGAACAGC |
| PIK3CA.9.7.PIK3CA.2_R | chr3:178936050-178936176 | CTGAGATCAGCCAAATTCAGT |
| PIK3CA.12.14.PIK3CA.1_F | chr3:178937712-178937936 | GCCATGCAGAAACTGACCCT |
| PIK3CA.12.14.PIK3CA.1_R | chr3:178937712-178937936 | GGAAAACTCTTCCAGCCAAACA |
| PIK3CA.15.11.PIK3CA.1_F | chr3:178942503-178942627 | TTAAAGGCTTGAAGAGTGTCG |
| PIK3CA.15.11.PIK3CA.1_R | chr3:178942503-178942627 | GCTAAATTCATGCATCATAAGCTC |
| PIK3CA.15.12.PIK3CA.2_F | chr3:178942473-178942601 | AGCAAAGGTACCTAGTAAAGTTT |
| PIK3CA.15.12.PIK3CA.2_R | chr3:178942473-178942601 | ACTCTTCCTTACCATCCCCA |
| PIK3CA.20.1.PIK3CA.1_F | chr3:178951922-178952081 | CAAGGCTTATCTAGCTATTCGA |
| PIK3CA.20.1.PIK3CA.1_R | chr3:178951922-178952081 | TCCAGCCACCATGATGTG |
| PIK3CA.20.2.PIK3CA.2_F | chr3:178952061-178952217 | GCAAGAGGCTTTGGAGTATT |
| PIK3CA.20.2.PIK3CA.2_R | chr3:178952061-178952217 | CTATGCAATCGGTCTTTGCC |
| PIK3CA.20.2.PIK3CA.3_F | chr3:178951796-178951954 | AGCTTTGTCTACGAAAGCCT |
| PIK3CA.20.2.PIK3CA.3_R | chr3:178951796-178951954 | ATTCCAGAGCCAAGCATCAT |
| PIK3CA.14.10.PIK3CA.1_F | chr3:178941854-178941978 | GTCAGTTTCTTACTGTGACTATCC |
| PIK3CA.14.10.PIK3CA.1_R | chr3:178941854-178941978 | TCAATGAAACCCCCAAGAAAG |
| PIK3CA.8.4.PIK3CA.1_F | chr3:178928184-178928344 | GGCAGTCAAACCTTCTCTCT |
| PIK3CA.8.4.PIK3CA.1_R | chr3:178928184-178928344 | TTTGCCTTACCAGTCCTGC |
| PIK3CA.8.5.PIK3CA.2_F | chr3:178928303-178928459 | CAGTGATTGAAGAGCATGCC |
| PIK3CA.8.5.PIK3CA.2_R | chr3:178928303-178928459 | GCAATATTGGTCCTAGAGTTCA |
| PIK3CA.4.4.PIK3CA.1_F | chr3:178921331-178921481 | CGCCCCCTTAATCTCTTACA |
| PIK3CA.4.4.PIK3CA.1_R | chr3:178921331-178921481 | CCCAAAGGGATTTTGTAGATGT |
| PIK3CA.4.8.PIK3CA.2_F | chr3:178921420-178921577 | TTCTCAACTGCCAATGGACT |
| PIK3CA.4.8.PIK3CA.2_R | chr3:178921420-178921577 | AAGCATCAGCATTTGACTTTAC |
| PIK3CA.19.1.PIK3CA.1_F | chr3:178948052-178948196 | TGGACACTTTTTGGATCACAAG |
| PIK3CA.19.1.PIK3CA.1_R | chr3:178948052-178948196 | TCTAATAGAGCAGCCAGAACTC |
| PIK3CA.19.2.PIK3CA.2_F | chr3:178947910-178948067 | TGGTGAAAGACGATGGACAA |
| PIK3CA.19.2.PIK3CA.2_R | chr3:178947910-178948067 | TGGCACACGTTCTCGTTTAT |
| PIK3CA.10.11.PIK3CA.1_F | chr3:178936950-178937094 | ACCTTTTGAACAGCATGCAA |
| PIK3CA.10.11.PIK3CA.1_R | chr3:178936950-178937094 | GTGACATACCAATTTGTACAACAG |
| PIK3CA.5.12.PIK3CA.1_F | chr3:178922248-178922402 | CGAGTGTGTGCATATGTGTA |
| PIK3CA.5.12.PIK3CA.1_R | chr3:178922248-178922402 | TGCTAAACACTAATATAACCTTTGG |
| TPMT.6.3.TPMT.1_F | chr6:18143792-18143929 | CTGAGAAAAACTTTTGTGGGGA |
| TPMT.6.3.TPMT.1_R | chr6:18143792-18143929 | GGGGACACAGTGTAGTTGG |
| TPMT.6.4.TPMT.2_F | chr6:18143928-18144078 | TCCCAAGTTCACTGATTTCCA |
| TPMT.6.4.TPMT.2_R | chr6:18143928-18144078 | CTGCATGTTCTTTGAAACCCT |
| TPMT.3.3.TPMT.1_F | chr6:18133939-18134094 | TGCAGTATGCTTCCTATGAGA |
| TPMT.3.3.TPMT.1_R | chr6:18133939-18134094 | GCAGATACAATGTTTTCCCTCC |
| TPMT.3.4.TPMT.2_F | chr6:18134040-18134197 | ACCCAACAACTTTACCTGGA |
| TPMT.3.4.TPMT.2_R | chr6:18134040-18134197 | AGCGAAAGTAACTTCTGGCT |
| TPMT.7.5.TPMT.1_F | chr6:18148021-18148172 | CACCCAAAGAATTCATCATTAAGGC |
| TPMT.7.5.TPMT.1_R | chr6:18148021-18148172 | TCACCTACCATACAATTGTCCT |
| TPMT.2.1.TPMT.1_F | chr6:18132292-18132441 | TGCTGGAAATACAGGCATGA |
| TPMT.2.1.TPMT.1_R | chr6:18132292-18132441 | AGTACACCTACGTCATTGGA |
| TPMT.8.1.TPMT.1_F | chr6:18149237-18149387 | ACTGATGTCCTTGTTCCTGA |
| TPMT.8.1.TPMT.1_R | chr6:18149237-18149387 | CGTAGGCACGGAAGACATAT |
| TPMT.8.2.TPMT.2_F | chr6:18149078-18149237 | TACATCATGCCACAGATGCA |
| TPMT.8.2.TPMT.2_R | chr6:18149078-18149237 | GAACGGCAAGACTGCTTTTC |
| TPMT.1.6.TPMT.1_F | chr6:18130877-18131010 | CCTCAAAAACATGTCAGTGTGA |
| TPMT.1.6.TPMT.1_R | chr6:18130877-18131010 | CATGTTACTCTTTCTTGTTTCAGG |

| | | |
|---|---|---|
| TPMT.1.7.TPMT.2_F | chr6:18130996-18131153 | TCAACCTTCTCAAGACAACGT |
| TPMT.1.7.TPMT.2_R | chr6:18130996-18131153 | GGTCATGAACTCCTGACCTC |
| TPMT.4.8.TPMT.1_F | chr6:18139137-18139315 | AGCTGATTTTCTAGAACCCAGAA |
| TPMT.4.8.TPMT.1_R | chr6:18139137-18139315 | GGGACGCTGCTCATCTTCTT |
| TPMT.5.1.TPMT.1_F | chr6:18139865-18140019 | GCCTGGCAAGCATTCAAATT |
| TPMT.5.1.TPMT.1_R | chr6:18139865-18140019 | GAACTTTTGCTTTGCTTGGC |
| BRCA2.11.42.BRCA2.1_F | chr13:32918572-32918813 | TCAAACATTAGGTCACTATTTGTTGT |
| BRCA2.11.42.BRCA2.1_R | chr13:32918572-32918813 | ACCATACCTATAGAGGGAGAACAGA |
| BRCA2.21.37.BRCA2.1_F | chr13:32953454-32953602 | ATTCCAATATCTTAAATGGTCACAG |
| BRCA2.21.37.BRCA2.1_R | chr13:32953454-32953602 | CAATACGCAACTTCCACACG |
| BRCA2.21.38.BRCA2.2_F | chr13:32953601-32953755 | AGGTTTATCAAGGGATGTCACA |
| BRCA2.21.38.BRCA2.2_R | chr13:32953601-32953755 | GTAGTGGATTTTGCTTCTCTGA |
| BRCA2.7.21.BRCA2.1_F | chr13:32903520-32903664 | TGTGCTTTTTGATGTCTGACA |
| BRCA2.7.21.BRCA2.1_R | chr13:32903520-32903664 | AGGCATTCCAAAATTGTTAGCA |
| BRCA2.26.1.BRCA2.1_F | chr13:32972357-32972518 | CCTTTATCACTTTGTATGGCCA |
| BRCA2.26.1.BRCA2.1_R | chr13:32972357-32972518 | TGTGCAGCCGGAGAAA |
| BRCA2.26.2.BRCA2.2_F | chr13:32972201-32972357 | GGAGGGAGACTGTGTGTAAT |
| BRCA2.26.2.BRCA2.2_R | chr13:32972201-32972357 | GTGTGGAAACAGACTTCCTT |
| BRCA2.26.2.BRCA2.3_F | chr13:32972538-32972699 | TCTCCGGCTGCACAGAA |
| BRCA2.26.2.BRCA2.3_R | chr13:32972538-32972699 | GTTGAACCAGACAAAAGAGCT |
| BRCA2.26.3.BRCA2.4_F | chr13:32972402-32972540 | CTCAGCCCAGATGACTTCAA |
| BRCA2.26.3.BRCA2.4_R | chr13:32972402-32972540 | AACTCCTTGGTGGCTGAAAT |
| BRCA2.26.3.BRCA2.5_F | chr13:32972769-32972907 | GAATCCACTAGGACTGCTCC |
| BRCA2.26.3.BRCA2.5_R | chr13:32972769-32972907 | ATTGTCGCCTTTGCAAATGC |
| BRCA2.26.4.BRCA2.6_F | chr13:32972686-32972844 | TAGCTGACGAAGAACTTGCA |
| BRCA2.26.4.BRCA2.6_R | chr13:32972686-32972844 | TCACATTCTTCCGTACTGGC |
| BRCA2.17.1.BRCA2.1_F | chr13:32937355-32937515 | AGAAGCAGAAGATCGGCTAT |
| BRCA2.17.1.BRCA2.1_R | chr13:32937355-32937515 | AACTGGGCCTTAACAGCAT |
| BRCA2.17.2.BRCA2.2_F | chr13:32937281-32937410 | ACAGTGGAATTCTAGAGTCACA |
| BRCA2.17.2.BRCA2.2_R | chr13:32937281-32937410 | GCGCTCAATGAAATTATGTCAG |
| BRCA2.17.2.BRCA2.3_F | chr13:32937525-32937682 | AGATGGGTGGTATGCTGTTA |
| BRCA2.17.2.BRCA2.3_R | chr13:32937525-32937682 | GACTGATTTTTACCAAGAGTGC |
| BRCA2.17.3.BRCA2.4_F | chr13:32937393-32937528 | GGATGACACAGCTGCAAAAA |
| BRCA2.17.3.BRCA2.4_R | chr13:32937393-32937528 | AGAGGGGAGGATCTAACTGG |
| BRCA2.2.1.BRCA2.1_F | chr13:32893354-32893482 | AAACTCCACAAAGGAAACCA |
| BRCA2.2.1.BRCA2.1_R | chr13:32893354-32893482 | AGTTTGTAGTTCTCCCCAGTC |
| BRCA2.2.2.BRCA2.2_F | chr13:32893199-32893354 | CTGGTTAAAACTAAGGTGGGAT |
| BRCA2.2.2.BRCA2.2_R | chr13:32893199-32893354 | GTTGAAGCCAGCTGATTATAAG |
| BRCA2.22.14.BRCA2.1_F | chr13:32953882-32954038 | GCATCTTTCTCATCTTTCTCCA |
| BRCA2.22.14.BRCA2.1_R | chr13:32953882-32954038 | GGTTTGTACCGGTAGTTGTTG |
| BRCA2.22.15.BRCA2.2_F | chr13:32954024-32954162 | ACATACAGTTAGCAGCGACA |
| BRCA2.22.15.BRCA2.2_R | chr13:32954024-32954162 | CCGTGGCTGGTAAATCTGAA |
| BRCA2.1.16.BRCA2.1_F | chr13:32890590-32890741 | CAAGCATTGGAGGAATATCGT |
| BRCA2.1.16.BRCA2.1_R | chr13:32890590-32890741 | TCTAAGCAACACTGTGACGT |
| BRCA2.18.42.BRCA2.1_F | chr13:32944554-32944766 | TGTCCAGATTTCTGCTAACAGT |
| BRCA2.18.42.BRCA2.1_R | chr13:32944554-32944766 | GGCAAGAGACCGAAACTCCA |
| BRCA2.18.43.BRCA2.2_F | chr13:32944470-32944597 | GGCAGTTCTAGAAGAATGAAAACTC |
| BRCA2.18.43.BRCA2.2_R | chr13:32944470-32944597 | GCAGAGGAAAAGGTCTAGGG |
| BRCA2.23.29.BRCA2.1_F | chr13:32954113-32954252 | TGCTTGTTAGTTTATGGAATCTCC |
| BRCA2.23.29.BRCA2.1_R | chr13:32954113-32954252 | TTCACAACAGAAACGACAAATC |
| BRCA2.23.30.BRCA2.2_F | chr13:32954247-32954406 | ATCTTGTTCTGAGGTGGACC |
| BRCA2.23.30.BRCA2.2_R | chr13:32954247-32954406 | TCAGAGGTTCAAAGAGGCTT |
| BRCA2.16.23.BRCA2.1_F | chr13:32936698-32936852 | AGGTGTGGATCCAAAGCTTA |
| BRCA2.16.23.BRCA2.1_R | chr13:32936698-32936852 | CCATACTGCCGTATATGATTACG |
| BRCA2.16.26.BRCA2.2_F | chr13:32936626-32936750 | TTGAATTCAGTATCATCCTATGTGG |
| BRCA2.16.26.BRCA2.2_R | chr13:32936626-32936750 | GGAAAGGCACATTCCATAGC |
| BRCA2.13.1.BRCA2.1_F | chr13:32929027-32929187 | AGGAACGTCAAGAGATACAGA |
| BRCA2.13.1.BRCA2.1_R | chr13:32929027-32929187 | GACTTTGGTTGGTCTGCC |
| BRCA2.13.2.BRCA2.2_F | chr13:32928881-32929039 | TGGCAACCATGGTGAATACA |
| BRCA2.13.2.BRCA2.2_R | chr13:32928881-32929039 | GAAATTCTTGACCAGGTGCG |
| BRCA2.13.16.BRCA2.3_F | chr13:32929208-32929367 | GGCAGACCAACCAAAGTCTT |
| BRCA2.13.16.BRCA2.3_R | chr13:32929208-32929367 | ACTGCTTGATTGGAGTTGTT |
| BRCA2.13.17.BRCA2.4_F | chr13:32929135-32929272 | AGCAGTTTCAGGACATCCAT |
| BRCA2.13.17.BRCA2.4_R | chr13:32929135-32929272 | GCTTTTGTCTGTTTTCCTCCA |
| BRCA2.13.22.BRCA2.5_F | chr13:32929295-32929439 | GGAGGAAAACAGACAAAAGCA |
| BRCA2.13.22.BRCA2.5_R | chr13:32929295-32929439 | AGGCAAAAATTCATCACACAAA |
| BRCA2.25.1.BRCA2.1_F | chr13:32971063-32971215 | GACATACTTTGCAATGAAGCAG |

| | | |
|---|---|---|
| BRCA2.25.1.BRCA2.1_R | chr13:32971063-32971215 | ACGATGGCCTCCATATATACT |
| BRCA2.25.2.BRCA2.2_F | chr13:32970957-32971107 | GGTCCAAACTTTTCATTTCTGC |
| BRCA2.25.2.BRCA2.2_R | chr13:32970957-32971107 | ACAGTCTTTAGTTGGGGTGG |
| BRCA2.6.1.BRCA2.1_F | chr13:32900640-32900773 | TTTTCTTTCCTCCCAGGGTC |
| BRCA2.6.1.BRCA2.1_R | chr13:32900640-32900773 | ACCTCATCTGCTCTTTCTTGT |
| BRCA2.6.2.BRCA2.2_F | chr13:32900534-32900673 | AGCATTCTGCCTCATACAGG |
| BRCA2.6.2.BRCA2.2_R | chr13:32900534-32900673 | TATCAGGATCCACCTCAGCT |
| BRCA2.3.42.BRCA2.1_F | chr13:32899166-32899418 | AAACACTTCCAAAGAATGCAAATTT |
| BRCA2.3.42.BRCA2.1_R | chr13:32899166-32899418 | GATCTTCTACCAGGCTCTTAGC |
| BRCA2.9.1.BRCA2.1_F | chr13:32906629-32906788 | ATTTTCCATGAAGCAAACGC |
| BRCA2.9.1.BRCA2.1_R | chr13:32906629-32906788 | AGTTGAGACCATTCACAGGC |
| BRCA2.9.2.BRCA2.2_F | chr13:32907089-32907248 | AGCAGGCAATATCTGGAACT |
| BRCA2.9.2.BRCA2.2_R | chr13:32907089-32907248 | GTCCTCCTTCTGTGAGCAAA |
| BRCA2.9.3.BRCA2.3_F | chr13:32906959-32907095 | TCAGAGAATTCTTTGCCACG |
| BRCA2.9.3.BRCA2.3_R | chr13:32906959-32907095 | ACCCTGAAATGAAGAAGCCA |
| BRCA2.9.4.BRCA2.4_F | chr13:32906835-32906985 | TTCAGGTCTAAATGGAGCCC |
| BRCA2.9.4.BRCA2.4_R | chr13:32906835-32906985 | CACTGTTTCCTCATTTAATGGC |
| BRCA2.9.5.BRCA2.5_F | chr13:32906785-32906939 | TCCAAGGAAGTTGTACCGTC |
| BRCA2.9.5.BRCA2.5_R | chr13:32906785-32906939 | ACGTGGCAAAGAATTCTCTG |
| BRCA2.9.18.BRCA2.6_F | chr13:32907269-32907429 | TGCTCACAGAAGGAGGAC |
| BRCA2.9.18.BRCA2.6_R | chr13:32907269-32907429 | TCTGATTTTTGGTCTTTCGGT |
| BRCA2.9.19.BRCA2.7_F | chr13:32907197-32907330 | TCAGGTCATATGACTGATCCA |
| BRCA2.9.19.BRCA2.7_R | chr13:32907197-32907330 | AACCTGCATTCTTCAAAGCT |
| BRCA2.9.23.BRCA2.8_F | chr13:32906529-32906670 | GAAACAGTTGTAGATACCTCTGAAG |
| BRCA2.9.23.BRCA2.8_R | chr13:32906529-32906670 | GGTTCCACTTCAGATACAAATGAG |
| BRCA2.9.26.BRCA2.9_F | chr13:32906429-32906580 | AGGATTTGGAAAAACATCAGGG |
| BRCA2.9.26.BRCA2.9_R | chr13:32906429-32906580 | TCCTAGTCTTGCTAGTTCTTACT |
| BRCA2.9.27.BRCA2.10_F | chr13:32906283-32906439 | GCCAAGTACTCAGAATAACCC |
| BRCA2.9.27.BRCA2.10_R | chr13:32906283-32906439 | GTGGTCTTTGCAGCTATTTACT |
| BRCA2.9.42.BRCA2.11_F | chr13:32907327-32907569 | AGCCACCACCACACAGAATT |
| BRCA2.9.42.BRCA2.11_R | chr13:32907327-32907569 | AACACAGAAGGAATCGTCATCT |
| BRCA2.12.42.BRCA2.1_F | chr13:32920859-32921066 | TGAGCATCTGTTACATTCACTGA |
| BRCA2.12.42.BRCA2.1_R | chr13:32920859-32921066 | AAAAACGAGACTTTTCTCATACTGT |
| BRCA2.20.29.BRCA2.1_F | chr13:32950760-32950908 | GCTTGGTTCTTTAGTTTTAGTTGC |
| BRCA2.20.29.BRCA2.1_R | chr13:32950760-32950908 | CTCAAGGTAAGCTGGGTCTG |
| BRCA2.20.30.BRCA2.2_F | chr13:32950850-32950988 | CCATCACGTGCACTAACAAG |
| BRCA2.20.30.BRCA2.2_R | chr13:32950850-32950988 | TGTGATGGCCAGAGAGTCTA |
| BRCA2.15.1.BRCA2.1_F | chr13:32931952-32932107 | GCAGAGTCTTTTCAGTTTCACA |
| BRCA2.15.1.BRCA2.1_R | chr13:32931952-32932107 | GAGAAGAAAGAGGGATGAGGG |
| BRCA2.15.42.BRCA2.2_F | chr13:32931805-32931999 | TTTGGTAAATTCAGTTTTGGTTTGTT |
| BRCA2.15.42.BRCA2.2_R | chr13:32931805-32931999 | CCATCCACCATCAGCCAACT |
| BRCA2.14.1.BRCA2.1_F | chr13:32930610-32930765 | GCCAGAGATATACAGGATATGC |
| BRCA2.14.1.BRCA2.1_R | chr13:32930610-32930765 | CTCTGTCATAAAAGCCATCAGT |
| BRCA2.14.2.BRCA2.2_F | chr13:32930525-32930678 | CCAGGGGTTGTGCTTTTTAA |
| BRCA2.14.2.BRCA2.2_R | chr13:32930525-32930678 | CAGAGAGATTCGAGGCAGAG |
| BRCA2.8.14.BRCA2.1_F | chr13:32905011-32905157 | GGGGGACTACTACTATATGTGC |
| BRCA2.8.14.BRCA2.1_R | chr13:32905011-32905157 | AGAGGACTTACCATGACTTGC |
| BRCA2.8.15.BRCA2.2_F | chr13:32905136-32905292 | GCTTCTGTGACAGACAGTGA |
| BRCA2.8.15.BRCA2.2_R | chr13:32905136-32905292 | TGAGGCAGGATCGCTTGA |
| BRCA2.4.18.BRCA2.1_F | chr13:32900229-32900374 | ACCTAAGGGATTTGCTTTGTT |
| BRCA2.4.18.BRCA2.1_R | chr13:32900229-32900374 | AAACTCCCACATACCACTGG |
| BRCA2.24.1.BRCA2.1_F | chr13:32968846-32969005 | ACTTGCCCCTTTCGTCTAT |
| BRCA2.24.1.BRCA2.1_R | chr13:32968846-32969005 | CCTCTTTTGGACTAGCAGAAA |
| BRCA2.24.2.BRCA2.2_F | chr13:32968935-32969086 | AGCCTCATATGTTAATTGCTGC |
| BRCA2.24.2.BRCA2.2_R | chr13:32968935-32969086 | ACCAAAATGTGTGGTGATGC |
| BRCA2.24.18.BRCA2.3_F | chr13:32968701-32968853 | GCTTTCGCCAAATTCAGCTA |
| BRCA2.24.18.BRCA2.3_R | chr13:32968701-32968853 | TGCCAGTAAATTGTAACATTCG |
| BRCA2.19.44.BRCA2.1_F | chr13:32945038-32945217 | CTGTGCCTGGCCTGATACAA |
| BRCA2.19.44.BRCA2.1_R | chr13:32945038-32945217 | ATTTTACCTTCATGTTCTTCAAATTCC |
| BRCA2.19.45.BRCA2.2_F | chr13:32945187-32945403 | TGTGGAGGCCCAACAAAAGA |
| BRCA2.19.45.BRCA2.2_R | chr13:32945187-32945403 | CCTGATATTTCTGTCCCTTGTTGC |
| BRCA2.10.1.BRCA2.1_F | chr13:32910710-32910872 | TGGCTGCAGCATGTCAC |
| BRCA2.10.1.BRCA2.1_R | chr13:32910710-32910872 | ACCTTTGAGCTTGTCTGACA |
| BRCA2.10.2.BRCA2.2_F | chr13:32911303-32911463 | TGGAGACACAGGTGATAAACA |
| BRCA2.10.2.BRCA2.2_R | chr13:32911303-32911463 | AGAGTCCTGCCCATTTGT |
| BRCA2.10.3.BRCA2.3_F | chr13:32911824-32911983 | CCTAGCCAAAAGGCAGAAA |
| BRCA2.10.3.BRCA2.3_R | chr13:32911824-32911983 | GGGGCATTCATTATGACATGA |

| | | |
|---|---|---|
| BRCA2.10.4.BRCA2.4_F | chr13:32912014-32912175 | GAATGCCCCATCGATTGG |
| BRCA2.10.4.BRCA2.4_R | chr13:32912014-32912175 | GCAGAGCTTCAGTAGAAACA |
| BRCA2.10.5.BRCA2.5_F | chr13:32911938-32912068 | TGAAGTGCCTGAAAACCAGA |
| BRCA2.10.5.BRCA2.5_R | chr13:32911938-32912068 | TCATTTTTCAACAGGCCAGC |
| BRCA2.10.5.BRCA2.6_F | chr13:32912616-32912775 | TTATCTGGCCAGTTTATGAAGG |
| BRCA2.10.5.BRCA2.6_R | chr13:32912616-32912775 | TTCCCACTTGCAGTCTGA |
| BRCA2.10.6.BRCA2.7_F | chr13:32913036-32913195 | CAACCCGAACGTGATGAAA |
| BRCA2.10.6.BRCA2.7_R | chr13:32913036-32913195 | TCTTTACAGGCCTCTCTGTAC |
| BRCA2.10.7.BRCA2.8_F | chr13:32913266-32913428 | CAGCTGCCCCAAAGTGT |
| BRCA2.10.7.BRCA2.8_R | chr13:32913266-32913428 | TGCAGGACTTTTTGCTGTTT |
| BRCA2.10.8.BRCA2.9_F | chr13:32913189-32913321 | TAGCCATCAATGGGCAAAGA |
| BRCA2.10.8.BRCA2.9_R | chr13:32913189-32913321 | TTAAGAGCTTAGGTGGCACC |
| BRCA2.10.8.BRCA2.10_F | chr13:32914399-32914558 | AGGGAAGCTTCATAAGTCAGT |
| BRCA2.10.8.BRCA2.10_R | chr13:32914399-32914558 | CTTCTCTTGTGAGCTGGTC |
| BRCA2.10.9.BRCA2.11_F | chr13:32915218-32915345 | TGGAAGATGATGAACTGACAGA |
| BRCA2.10.9.BRCA2.11_R | chr13:32915218-32915345 | TGGCAACACGAAAGGTAAAA |
| BRCA2.10.9.BRCA2.12_F | chr13:32914276-32914426 | TGCTGACATTCAGAGTGAAGA |
| BRCA2.10.9.BRCA2.12_R | chr13:32914276-32914426 | GATTTTCCACTTGCTGTGCT |
| BRCA2.10.10.BRCA2.13_F | chr13:32915062-32915219 | CAGGCTTCACCTAAAAACGT |
| BRCA2.10.10.BRCA2.13_R | chr13:32915062-32915219 | GGCATGACTTGGCAGTTTAG |
| BRCA2.10.10.BRCA2.14_F | chr13:32914167-32914298 | GCCAAACGAAAATTATGGCAG |
| BRCA2.10.10.BRCA2.14_R | chr13:32914167-32914298 | GAAACTTTCTCCAATCCAGACA |
| BRCA2.10.11.BRCA2.15_F | chr13:32914018-32914172 | AATTTTGAGGTAGGGCCACC |
| BRCA2.10.11.BRCA2.15_R | chr13:32914018-32914172 | TGAATCATCCAATGCCTCGT |
| BRCA2.10.12.BRCA2.16_F | chr13:32913899-32914029 | TGCAAATGCATACCCACAA |
| BRCA2.10.12.BRCA2.16_R | chr13:32913899-32914029 | CAAACGATTTTACCACTGGCT |
| BRCA2.10.13.BRCA2.17_F | chr13:32913754-32913907 | ACAGCTATTCCTACCATTCTGA |
| BRCA2.10.13.BRCA2.17_R | chr13:32913754-32913907 | GTTCCTCAACGCAAATATCTTC |
| BRCA2.10.14.BRCA2.18_F | chr13:32910857-32911012 | CCTAGTCATGATTTCTAGAGGC |
| BRCA2.10.14.BRCA2.18_R | chr13:32910857-32911012 | TCTTGAAGGTGATGCTACTCT |
| BRCA2.10.14.BRCA2.19_F | chr13:32914531-32914671 | ACCAAGCAAGTCTTTTCCAA |
| BRCA2.10.14.BRCA2.19_R | chr13:32914531-32914671 | AAACTTGCTTTCCACTTGCT |
| BRCA2.10.18.BRCA2.20_F | chr13:32911691-32911847 | AGCAAGCCTCAGTCAATTAAT |
| BRCA2.10.18.BRCA2.20_R | chr13:32911691-32911847 | TCAAACTGACTTCCTGATTCTT |
| BRCA2.10.18.BRCA2.21_F | chr13:32912516-32912658 | AGAATTTGATGGCAGTGATTCA |
| BRCA2.10.18.BRCA2.21_R | chr13:32912516-32912658 | GAGCTTTCGCAACTTCCAAA |
| BRCA2.10.19.BRCA2.22_F | chr13:32911558-32911697 | AGGAAATCAAGCTCTCTGAACA |
| BRCA2.10.19.BRCA2.22_R | chr13:32911558-32911697 | ACACTACTCTGTAAATGTGCAG |
| BRCA2.10.19.BRCA2.23_F | chr13:32912412-32912542 | TGACTACTGGCACTTTTGTTG |
| BRCA2.10.19.BRCA2.23_R | chr13:32912412-32912542 | AGCAAGTCCGTTTCATCTTT |
| BRCA2.10.20.BRCA2.24_F | chr13:32914667-32914822 | TTCATCTGCTTTCTCTGGATTT |
| BRCA2.10.20.BRCA2.24_R | chr13:32914667-32914822 | CAGTGCTCTGGGTTTCTCTT |
| BRCA2.10.23.BRCA2.25_F | chr13:32911187-32911338 | GTCTTCCAAGTAGCTAATGAAAGG |
| BRCA2.10.23.BRCA2.25_R | chr13:32911187-32911338 | TGTTCTCCTCTGCAAGAACA |
| BRCA2.10.24.BRCA2.26_F | chr13:32911058-32911210 | GGTACAATTCAACCAAAACACA |
| BRCA2.10.24.BRCA2.26_R | chr13:32911058-32911210 | AGTCTGTTTCATGAAGTTCCTT |
| BRCA2.10.25.BRCA2.27_F | chr13:32910954-32911111 | CCCATGGAAAAGAATCAAGATG |
| BRCA2.10.25.BRCA2.27_R | chr13:32910954-32911111 | TCAGAGTCTGGATTGACAGT |
| BRCA2.10.26.BRCA2.28_F | chr13:32914927-32915081 | TGAAGGTGGTTCTTCAGAAAA |
| BRCA2.10.26.BRCA2.28_R | chr13:32914927-32915081 | CAGGAACATCAGAAAAAGTTTCAG |
| BRCA2.10.28.BRCA2.29_F | chr13:32914821-32914968 | GTATCAAAAATACTTCCTCGTGTTG |
| BRCA2.10.28.BRCA2.29_R | chr13:32914821-32914968 | ACCAACTGTTGTTTGTCTTGT |
| BRCA2.10.31.BRCA2.30_F | chr13:32910565-32910709 | CTCAGGATCTTGATTATAAAGAAGC |
| BRCA2.10.31.BRCA2.30_R | chr13:32910565-32910709 | TCCACTTTTGAATGTTGTACTGG |
| BRCA2.10.32.BRCA2.31_F | chr13:32910441-32910598 | GTGAAAAGAAGCTGTTCACAGA |
| BRCA2.10.32.BRCA2.31_R | chr13:32910441-32910598 | AGAGAATCAGCTTCTGGGGT |
| BRCA2.10.33.BRCA2.32_F | chr13:32910327-32910473 | TGTGCCCAAACACTACCTTT |
| BRCA2.10.33.BRCA2.32_R | chr13:32910327-32910473 | TCAGAATTGTCCCAAAAGAGC |
| BRCA2.10.34.BRCA2.33_F | chr13:32911426-32911566 | AAAATCGGACATCTCCTTGAA |
| BRCA2.10.34.BRCA2.33_R | chr13:32911426-32911566 | TCTTTGAAGAACATTTTGCTCTTC |
| BRCA2.10.34.BRCA2.34_F | chr13:32912299-32912449 | CTTCAAGTAAATGTCATGATTCTGT |
| BRCA2.10.34.BRCA2.34_R | chr13:32912299-32912449 | GGCAGCAGTATATTTGTTATCTTC |
| BRCA2.10.34.BRCA2.35_F | chr13:32912922-32913060 | GACATAAGAAAGAACAAAATGGACA |
| BRCA2.10.34.BRCA2.35_R | chr13:32912922-32913060 | TCCCGCTAGCTGTATGAAAA |
| BRCA2.10.35.BRCA2.36_F | chr13:32912811-32912966 | GGGAAAAAATATTAGTGTCGCCA |
| BRCA2.10.35.BRCA2.36_R | chr13:32912811-32912966 | ACCAACTGGGACACTTTCTT |
| BRCA2.10.36.BRCA2.37_F | chr13:32912696-32912842 | CTCAAGAAGCATGTCATGGT |

| | | |
|---|---|---|
| BRCA2.10.36.BRCA2.37_R | chr13:32912696-32912842 | GCAATTCTTCTGGTTTCTGATC |
| BRCA2.10.37.BRCA2.38_F | chr13:32913382-32913533 | GACAAACTGAAAATCTCAAAACATC |
| BRCA2.10.37.BRCA2.38_R | chr13:32913382-32913533 | TTCAAGTAATGAAGTCTGACTCA |
| BRCA2.10.39.BRCA2.39_F | chr13:32913516-32913671 | GCCTTAGCTTTTTACACAAGTT |
| BRCA2.10.39.BRCA2.39_R | chr13:32913516-32913671 | TCGGAGAGATGATTTTTGTCAT |
| BRCA2.10.40.BRCA2.40_F | chr13:32912146-32912284 | AATGAAGTGGGGTTTAGGGG |
| BRCA2.10.40.BRCA2.40_R | chr13:32912146-32912284 | CATTGAAACAACAGAATCATGACA |
| BRCA2.10.41.BRCA2.41_F | chr13:32912256-32912395 | TGAGGAAACTTCTGCAGAGG |
| BRCA2.10.41.BRCA2.41_R | chr13:32912256-32912395 | TCTTCAACAAAAGTGCCAGT |
| BRCA2.10.42.BRCA2.42_F | chr13:32913548-32913792 | AAACTTCTGTGAGTCAGACTTCA |
| BRCA2.10.42.BRCA2.42_R | chr13:32913548-32913792 | ACTGGCTCAATACCAGAATCAAGT |
| BRCA2.5.34.BRCA2.1_F | chr13:32900308-32900455 | GTCAGGTATGATTAAAAACAATGCT |
| BRCA2.5.34.BRCA2.1_R | chr13:32900308-32900455 | ACGCTATTGTCAAATTCTCAATT |
| ALK.11.1.ALK.1_F | chr2:29448287-29448446 | GACACTATTCAGTCCTGCCT |
| ALK.11.1.ALK.1_R | chr2:29448287-29448446 | TGGTTGTCAATACCCCAGTG |
| ALK.21.1.ALK.1_F | chr2:29519754-29519913 | GGGAGATGCATAGAGCCTAC |
| ALK.21.1.ALK.1_R | chr2:29519754-29519913 | CCTCACAGCTCCGAATGT |
| ALK.21.2.ALK.2_F | chr2:29519908-29520062 | CCTCAAGACTCCACGAATGA |
| ALK.21.2.ALK.2_R | chr2:29519908-29520062 | AAAGCCTCTTGGTGAGACAG |
| ALK.7.1.ALK.1_F | chr2:29443560-29443706 | AACAACTGCAGCAAAGACTG |
| ALK.7.1.ALK.1_R | chr2:29443560-29443706 | GGTTACATCCCTCTCTGCTC |
| ALK.26.1.ALK.1_F | chr2:29754781-29754940 | TTGGACATGGAGAAGTACTTAC |
| ALK.26.1.ALK.1_R | chr2:29754781-29754940 | CAGCTGACTCCAAGCACA |
| ALK.26.2.ALK.2_F | chr2:29754865-29755016 | GGGCAATGTACCTTCCAGAG |
| ALK.26.2.ALK.2_R | chr2:29754865-29755016 | CTGCTGGTCTTACCTGAACA |
| ALK.2.1.ALK.1_F | chr2:29419630-29419780 | TGTTGACCAAAGGGAGAAAATG |
| ALK.2.1.ALK.1_R | chr2:29419630-29419780 | CCTGGGGCTGGAAGAAAATA |
| ALK.17.1.ALK.1_F | chr2:29462545-29462698 | CCTGCTCTGGGCACTTA |
| ALK.17.1.ALK.1_R | chr2:29462545-29462698 | AACGTTCTTTGTCCCATTGC |
| ALK.22.1.ALK.1_F | chr2:29541134-29541290 | GAAGTGACAAGAGGTGGGAG |
| ALK.22.1.ALK.1_R | chr2:29541134-29541290 | CTGATAATGGCGTGCTTGTG |
| ALK.1.1.ALK.1_F | chr2:29416088-29416250 | AGTGAGTGTGCGACCGA |
| ALK.1.1.ALK.1_R | chr2:29416088-29416250 | GCTGACTGCCAATATGAAGG |
| ALK.1.2.ALK.2_F | chr2:29416251-29416411 | AGCCTGAACAGAGGTACCT |
| ALK.1.2.ALK.2_R | chr2:29416251-29416411 | GGCTCCTGGTTTACAGAGAA |
| ALK.1.3.ALK.3_F | chr2:29416420-29416581 | TTTTTGGTGGGTTTCTCTGT |
| ALK.1.3.ALK.3_R | chr2:29416420-29416581 | GCTGCAAAGAAACCCACA |
| ALK.1.4.ALK.4_F | chr2:29416390-29416519 | TGTGGCTCCTTCTTTGCTAT |
| ALK.1.4.ALK.4_R | chr2:29416390-29416519 | GAAGGGGGACACGTGAATAT |
| ALK.1.4.ALK.5_F | chr2:29416591-29416751 | CTCTGCAGCTGTGGGTTT |
| ALK.1.4.ALK.5_R | chr2:29416591-29416751 | ACCGCTTTGCCGATAGAAT |
| ALK.1.5.ALK.6_F | chr2:29416735-29416877 | ACAGGCACTTTCTCTTCCTC |
| ALK.1.5.ALK.6_R | chr2:29416735-29416877 | CTGGTTTCCTCATCTGTAGAAT |
| ALK.1.5.ALK.7_F | chr2:29416539-29416668 | CATATTCACGTGTCCCCCTT |
| ALK.1.5.ALK.7_R | chr2:29416539-29416668 | CTCCTCTCCTGGTCTCTCAA |
| ALK.18.1.ALK.1_F | chr2:29473970-29474131 | CCCCTCTTCCATACGCA |
| ALK.18.1.ALK.1_R | chr2:29473970-29474131 | CTCCTCTTGTGCCACAGTT |
| ALK.18.2.ALK.2_F | chr2:29474065-29474202 | CAGGTTGGAGTTCTGGTAGG |
| ALK.18.2.ALK.2_R | chr2:29474065-29474202 | GAGCAGATATCTCAGAGGCC |
| ALK.13.1.ALK.1_F | chr2:29450411-29450541 | GGAGGACTGACCTAAGCAAG |
| ALK.13.1.ALK.1_R | chr2:29450411-29450541 | CTCTGCTCTGGTTTGCTACT |
| ALK.23.1.ALK.1_F | chr2:29543616-29543765 | AAGGCAGGAGAGCAGTAGTA |
| ALK.23.1.ALK.1_R | chr2:29543616-29543765 | TCCTGAAAATGATGGTGCCT |
| ALK.16.1.ALK.1_F | chr2:29456415-29456574 | AGGAACCTGGTGGAAATCTG |
| ALK.16.1.ALK.1_R | chr2:29456415-29456574 | CCACAAACCTAACGTGCTTT |
| ALK.29.1.ALK.1_F | chr2:30143118-30143283 | ACCAACTGCTTGGCACG |
| ALK.29.1.ALK.1_R | chr2:30143118-30143283 | TCCTCCTCGGAGCTGAA |
| ALK.29.2.ALK.2_F | chr2:30143374-30143532 | AGTCAACTGCCAGACTCTTC |
| ALK.29.2.ALK.2_R | chr2:30143374-30143532 | CAGTGTAAACGGCCTCCTC |
| ALK.29.2.ALK.3_F | chr2:30142983-30143141 | ATCCTCAGTCGCCCTTC |
| ALK.29.2.ALK.3_R | chr2:30142983-30143141 | TGTCCAGGGTGCTGAAG |
| ALK.29.3.ALK.4_F | chr2:30142839-30142991 | AAACACTAAATCCCGGCACA |
| ALK.29.3.ALK.4_R | chr2:30142839-30142991 | AGCTGTTCAGTTGGTGGATT |
| ALK.29.6.ALK.5_F | chr2:30143235-30143384 | CCCAGCAACCTGAGCAG |
| ALK.29.6.ALK.5_R | chr2:30143235-30143384 | GAGCCACTCAGCTACTCG |
| ALK.27.1.ALK.1_F | chr2:29917721-29917880 | AGCCTCTCCCTTACCTCTG |
| ALK.27.1.ALK.1_R | chr2:29917721-29917880 | GACCCTTGGCTATTATTGCAG |

| | | |
|---|---|---|
| ALK.27.2.ALK.2_F | chr2:29917591-29917721 | AAGCCATGGAAAGTCACAGA |
| ALK.27.2.ALK.2_R | chr2:29917591-29917721 | AGAGCGTTCTAAGGAGATGC |
| ALK.25.1.ALK.1_F | chr2:29606575-29606725 | ACAATAGGCTACCAAGGAGC |
| ALK.25.1.ALK.1_R | chr2:29606575-29606725 | TCTGTTTTCCAATGCAACCAG |
| ALK.6.1.ALK.1_F | chr2:29436838-29436976 | ATGACAGGAAGAGCACAGTC |
| ALK.6.1.ALK.1_R | chr2:29436838-29436976 | CTCTCACTGACAAGCTCCTC |
| ALK.28.1.ALK.1_F | chr2:29940457-29940597 | TACTTACCATATCGGCTGCG |
| ALK.28.1.ALK.1_R | chr2:29940457-29940597 | TAAAAGACACACCCCAAGCA |
| ALK.28.2.ALK.2_F | chr2:29940317-29940460 | GAGACAGAGGGCTCAGAAAG |
| ALK.28.2.ALK.2_R | chr2:29940317-29940460 | GACTCCTTCCCTTTCCTGTC |
| ALK.3.1.ALK.1_F | chr2:29420395-29420551 | AGTAACTAGCAGAAGTGTTCCT |
| ALK.3.1.ALK.1_R | chr2:29420395-29420551 | CATCTCCATGTCCTCTGTCC |
| ALK.9.1.ALK.1_F | chr2:29445380-29445514 | CTCAAGAGTGAGCCACTTCT |
| ALK.9.1.ALK.1_R | chr2:29445380-29445514 | CAGCTGCCTCATTATTGTGG |
| ALK.12.1.ALK.1_F | chr2:29449798-29449941 | CCCCCTTACCAATGCAGGA |
| ALK.12.1.ALK.1_R | chr2:29449798-29449941 | CCCTCCCTCCTTGTCACTA |
| ALK.12.2.ALK.2_F | chr2:29449683-29449827 | TAGGGGAGGGACAGAAAGTT |
| ALK.12.2.ALK.2_R | chr2:29449683-29449827 | AGGTCATCTGCTTCTGTGAC |
| ALK.20.1.ALK.1_F | chr2:29498268-29498401 | GGGGTCAGAGTGAACTCAC |
| ALK.20.1.ALK.1_R | chr2:29498268-29498401 | AATCCTAAGACAAGACCCCC |
| ALK.15.1.ALK.1_F | chr2:29455173-29455333 | CTTGCAGCGCTTTACCTG |
| ALK.15.1.ALK.1_R | chr2:29455173-29455333 | AGCTCGGTTTCTCAATGTCT |
| ALK.15.2.ALK.2_F | chr2:29455047-29455188 | TCCCTGGATATCGGTACCAA |
| ALK.15.2.ALK.2_R | chr2:29455047-29455188 | CCTCGGTTCTAGGGCTAAAC |
| ALK.14.1.ALK.1_F | chr2:29451772-29451932 | ATATATCCTCCGCCTCCTCC |
| ALK.14.1.ALK.1_R | chr2:29451772-29451932 | TCCCTATCTCTTCCCGCAG |
| ALK.14.2.ALK.2_F | chr2:29451740-29451890 | AGTGACACCTTGAACACGAA |
| ALK.14.2.ALK.2_R | chr2:29451740-29451890 | TAACACTTCCTTGCTCTGGG |
| ALK.8.1.ALK.1_F | chr2:29445209-29445363 | GGTGTCTCTCTGTGGCTTTA |
| ALK.8.1.ALK.1_R | chr2:29445209-29445363 | GGTAAGAAGTGGCTCACTCT |
| ALK.4.1.ALK.1_F | chr2:29430029-29430163 | GATACCTGGAGGATGATGGC |
| ALK.4.1.ALK.1_R | chr2:29430029-29430163 | TGCCATCTCCAGATCCTAGT |
| ALK.24.1.ALK.1_F | chr2:29551215-29551355 | GCAGCCCCATCTACTCA |
| ALK.24.1.ALK.1_R | chr2:29551215-29551355 | CATGGTTGTGTGTCTGTGTG |
| ALK.19.1.ALK.1_F | chr2:29497961-29498096 | CACATTTGTGAGCTGAACCC |
| ALK.19.1.ALK.1_R | chr2:29497961-29498096 | CCGTGGGGTCTTCTCTATTG |
| ALK.10.1.ALK.1_F | chr2:29446227-29446389 | CCGAATGAGGGTGATGTTTT |
| ALK.10.1.ALK.1_R | chr2:29446227-29446389 | ACCCACCTGCAGTGTAC |
| ALK.10.2.ALK.2_F | chr2:29446293-29446447 | AAAGCAGTAGTTGGGGTTGT |
| ALK.10.2.ALK.2_R | chr2:29446293-29446447 | GTAGGAAGTGGCCTGTGTAG |
| ALK.10.2.ALK.3_F | chr2:29446080-29446235 | GTCAAGGCTTGTCCTCCTAG |
| ALK.10.2.ALK.3_R | chr2:29446080-29446235 | CATCAGTGACCTGAAGGAGG |
| ALK.5.1.ALK.1_F | chr2:29432651-29432793 | GGTGAGGCAGTCTTTACTCA |
| ALK.5.1.ALK.1_R | chr2:29432651-29432793 | CTAGTGATGGCCGTTGTACA |
| TP53.6.1.TP53.1_F | chr17:7576571-7576709 | TCAGGAAGTAACACCATCGT |
| TP53.6.1.TP53.1_R | chr17:7576571-7576709 | AAAGACAATGGCTCCTGGTT |
| TP53.11.1.TP53.1_F | chr17:7578380-7578540 | GCTGCTCACCATCGCTAT |
| TP53.11.1.TP53.1_R | chr17:7578380-7578540 | TACAGTACTCCCCTGCCCT |
| TP53.11.2.TP53.2_F | chr17:7578444-7578578 | ATGTGCTGTGACTGCTTGTA |
| TP53.11.2.TP53.2_R | chr17:7578444-7578578 | CTTGTGCCCTGACTTTCAAC |
| TP53.11.2.TP53.3_F | chr17:7578282-7578423 | CGGATAAGATGCTGAGGAGG |
| TP53.11.2.TP53.3_R | chr17:7578282-7578423 | TACAAGCAGTCACAGCACAT |
| TP53.3.1.TP53.1_F | chr17:7572854-7573011 | CAAGCAAGGGTTCAAAGACC |
| TP53.3.1.TP53.1_R | chr17:7572854-7573011 | CTCCCTGCTTCTGTCTCCTA |
| TP53.7.1.TP53.1_F | chr17:7576797-7576928 | CAGTCAAGAAGAAAACGGCA |
| TP53.7.1.TP53.1_R | chr17:7576797-7576928 | CCTTTCCTTGCCTCTTTCCT |
| TP53.9.1.TP53.1_F | chr17:7577451-7577609 | GGTCAGAGGCAAGCAGA |
| TP53.9.1.TP53.1_R | chr17:7577451-7577609 | TGGGCCTGTGTTATCTCCTA |
| TP53.12.1.TP53.1_F | chr17:7579322-7579484 | CAACTGACCGTGCAAGTC |
| TP53.12.1.TP53.1_R | chr17:7579322-7579484 | GAAGCTCCCAGAATGCCAG |
| TP53.12.2.TP53.2_F | chr17:7579463-7579596 | TAGGAGCTGCTGGTGCA |
| TP53.12.2.TP53.2_R | chr17:7579463-7579596 | TGACTGCTCTTTTCACCCAT |
| TP53.12.2.TP53.3_F | chr17:7579234-7579386 | GGGTGAAGAGGAATCCCAAA |
| TP53.12.2.TP53.3_R | chr17:7579234-7579386 | CATCTTCTGTCCCTTCCCAG |
| TP53.14.1.TP53.1_F | chr17:7579838-7579967 | CCTTCCAATGGATCCACTCA |
| TP53.14.1.TP53.1_R | chr17:7579838-7579967 | CAGGGTTGGAAGTGTCTCAT |
| TP53.15.1.TP53.1_F | chr17:7580598-7580738 | AGTCATTCTCAGCCTGCAAT |

| | | |
|---|---|---|
| TP53.15.1.TP53.1_R | chr17:7580598-7580738 | GTTTGTAATGCAGGGCTGAG |
| TP53.8.1.TP53.1_F | chr17:7577019-7577174 | CTTCTTGTCCTGCTTGCTTAC |
| TP53.8.1.TP53.1_R | chr17:7577019-7577174 | CCTTACTGCCTCTTGCTTCT |
| TP53.1.1.TP53.1_F | chr17:7565193-7565338 | GGTTAAGAGATCCTCCTGCC |
| TP53.1.1.TP53.1_R | chr17:7565193-7565338 | GACTCCGTCTCTCTCTCTCT |
| TP53.4.1.TP53.1_F | chr17:7573892-7574043 | TTTCCAACCTAGGAAGGCAG |
| TP53.4.1.TP53.1_R | chr17:7573892-7574043 | CTTACTTCTCCCCCTCCTCT |
| TP53.10.1.TP53.1_F | chr17:7578135-7578292 | AGGGCCACTGACAACCA |
| TP53.10.1.TP53.1_R | chr17:7578135-7578292 | TGATTCCTCACTGATTGCTCT |
| TP53.13.1.TP53.1_F | chr17:7579617-7579753 | ATGGGTGAAAAGAGCAGTCA |
| TP53.13.1.TP53.1_R | chr17:7579617-7579753 | CCTGTGGGAAGCGAAAATTC |
| TP53.5.1.TP53.1_F | chr17:7576483-7576641 | GCAGGCTAGGCTAAGCTATG |
| TP53.5.1.TP53.1_R | chr17:7576483-7576641 | AAAGGACCAGACCAGCTTTC |
| DPYD.11.1.DPYD.1_F | chr1:97981312-97981471 | GAGAAAGTTTTGGTGAGGGC |
| DPYD.11.1.DPYD.1_R | chr1:97981312-97981471 | TATGGAGCTTCCGTTTCTGC |
| DPYD.11.2.DPYD.2_F | chr1:97981469-97981628 | AAAAGAGGGGTAGTTCAGGC |
| DPYD.11.2.DPYD.2_R | chr1:97981469-97981628 | CTCACTGACTTTTTGCCAGT |
| DPYD.11.9.DPYD.3_F | chr1:97981173-97981311 | TGTGTAATGATAGGTCTTGTCAA |
| DPYD.11.9.DPYD.3_R | chr1:97981173-97981311 | TTGAAGCTGGATGGGGTTTT |
| DPYD.21.10.DPYD.1_F | chr1:98205887-98206103 | ACAAGCTGTATTCTGTACCCACA |
| DPYD.21.10.DPYD.1_R | chr1:98205887-98206103 | ACTAGACACGGACTCTGAATGA |
| DPYD.7.1.DPYD.1_F | chr1:97771750-97771907 | TACCTTCCTTTGCAGCTCTT |
| DPYD.7.1.DPYD.1_R | chr1:97771750-97771907 | TGCTGTTGTTCCAGTACACA |
| DPYD.7.2.DPYD.2_F | chr1:97771606-97771764 | TTGTGGGATCAAGTGCTCAA |
| DPYD.7.2.DPYD.2_R | chr1:97771606-97771764 | CTGACCCCAAATGTCACTGA |
| DPYD.2.1.DPYD.1_F | chr1:97547879-97548035 | AGCATTCTAATTCCAGCAGGA |
| DPYD.2.1.DPYD.1_R | chr1:97547879-97548035 | GCTTGCTAAGTAATTCAGTGGC |
| DPYD.17.1.DPYD.1_F | chr1:98157235-98157365 | AGTGTAGAGCTTACTCCTTTCT |
| DPYD.17.1.DPYD.1_R | chr1:98157235-98157365 | TGATGCCTGTTTTTCTTTCTCC |
| DPYD.22.7.DPYD.1_F | chr1:98293661-98293790 | TGACTGTTGCTATGGTGACC |
| DPYD.22.7.DPYD.1_R | chr1:98293661-98293790 | CTGCATAGATACAATTTTTCTCAGG |
| DPYD.1.1.DPYD.1_F | chr1:97544570-97544727 | AGATAAGGGTACGCCTCTCT |
| DPYD.1.1.DPYD.1_R | chr1:97544570-97544727 | ACCTTTGTGGTCAGTGACAT |
| DPYD.1.2.DPYD.2_F | chr1:97544463-97544608 | GGAAAGAGCTGAACACAAGG |
| DPYD.1.2.DPYD.2_R | chr1:97544463-97544608 | TGTTTGCCCTATTGTCGACT |
| DPYD.18.1.DPYD.1_F | chr1:98164962-98165106 | AGTGATGTCAGAGTACCCCA |
| DPYD.18.1.DPYD.1_R | chr1:98164962-98165106 | CCATGACAATTGATTTCCCCG |
| DPYD.18.2.DPYD.2_F | chr1:98164822-98164964 | ATCTGTGAGCCTGAAGTTCC |
| DPYD.18.2.DPYD.2_R | chr1:98164822-98164964 | TTGTGCTTCCTTTTTGGCTC |
| DPYD.16.10.DPYD.1_F | chr1:98144579-98144805 | CACGAAAATGTCTGAAGGCAGT |
| DPYD.16.10.DPYD.1_R | chr1:98144579-98144805 | TGGCTGACTTTTCATTCTTTTTGA |
| DPYD.23.3.DPYD.1_F | chr1:98348778-98348920 | TGCCTTACAATGTGTGGAGT |
| DPYD.23.3.DPYD.1_R | chr1:98348778-98348920 | TGCTGTCTTTAGAGTATCCTGG |
| DPYD.23.4.DPYD.2_F | chr1:98348913-98349050 | GCATGAGTTTGTGTTCGAGG |
| DPYD.23.4.DPYD.2_R | chr1:98348913-98349050 | AGATTGTACCACTGCACTCC |
| DPYD.13.1.DPYD.1_F | chr1:98039315-98039477 | TCAGCTCCCAGCACTGTA |
| DPYD.13.1.DPYD.1_R | chr1:98039315-98039477 | TTTCTGCCATTCCTGTCCC |
| DPYD.13.6.DPYD.2_F | chr1:98039382-98039536 | GGCTTTCAGATGGACCATCT |
| DPYD.13.6.DPYD.2_R | chr1:98039382-98039536 | TCAAGTTTCTTCTCTGTTCTGT |
| DPYD.6.1.DPYD.1_F | chr1:97770804-97770960 | AAGCACAATGCAAGAAGTGG |
| DPYD.6.1.DPYD.1_R | chr1:97770804-97770960 | TCGTGTCTTATAAGAGCTGCA |
| DPYD.3.1.DPYD.1_F | chr1:97564064-97564210 | ACCTTGATGGTAGGAATAGGC |
| DPYD.3.1.DPYD.1_R | chr1:97564064-97564210 | TCTGAGCTAACATGCTTCCT |
| DPYD.3.5.DPYD.2_F | chr1:97563912-97564066 | GCAGTTTTCACCATGGACAG |
| DPYD.3.5.DPYD.2_R | chr1:97563912-97564066 | AGAGAAACTGTTTTATCCCCAA |
| DPYD.9.8.DPYD.1_F | chr1:97847895-97848044 | CTCATGGCAGCTCTTTATTTAAAAC |
| DPYD.9.8.DPYD.1_R | chr1:97847895-97848044 | GTACAACTGGATAACTACTTGAACT |
| DPYD.12.1.DPYD.1_F | chr1:98015166-98015324 | TGCTTTCCATCATTCACCG |
| DPYD.12.1.DPYD.1_R | chr1:98015166-98015324 | GTCTGTTGACTAGTGATTGTCT |
| DPYD.12.2.DPYD.2_F | chr1:98015112-98015242 | GTGGAAATGATGGCAAATGC |
| DPYD.12.2.DPYD.2_R | chr1:98015112-98015242 | GGTCTCCCAGAAGTAGATCC |
| DPYD.15.10.DPYD.1_F | chr1:98060526-98060748 | GGTTGGGTGTGAGAGCTGAA |
| DPYD.15.10.DPYD.1_R | chr1:98060526-98060748 | AAGCCCCTCCTCCTGCTAAT |
| DPYD.20.3.DPYD.1_F | chr1:98187036-98187178 | TTGTGCATGGTGATGGTAGT |
| DPYD.20.3.DPYD.1_R | chr1:98187036-98187178 | TTCTGACAACCCACTTGGTC |
| DPYD.20.5.DPYD.2_F | chr1:98187148-98187277 | TGCATCCACCTACACAAAGA |
| DPYD.20.5.DPYD.2_R | chr1:98187148-98187277 | TGTTTGTCGTAATTTGGCTGT |

| | | |
|---|---|---|
| DPYD.14.1.DPYD.1_F | chr1:98058794-98058944 | CTCCTCAGGGACAGCTCTTA |
| DPYD.14.1.DPYD.1_R | chr1:98058794-98058944 | GGGGTTTTCCTTTCATCATTCA |
| DPYD.14.2.DPYD.2_F | chr1:98058658-98058810 | GAAACAATTATGTGTAGCCCTG |
| DPYD.14.2.DPYD.2_R | chr1:98058658-98058810 | CGTGTGTTCATCGTCTTCAG |
| DPYD.8.1.DPYD.1_F | chr1:97839109-97839250 | ACTTAGTGGCAGCTGTCAAG |
| DPYD.8.1.DPYD.1_R | chr1:97839109-97839250 | CAACGGTGAAAGCCTATTGG |
| DPYD.4.1.DPYD.1_F | chr1:97658653-97658809 | TGAGTTCAGCTATACGTGGA |
| DPYD.4.1.DPYD.1_R | chr1:97658653-97658809 | TGGTTGACTGCTCTGTCTTT |
| DPYD.4.2.DPYD.2_F | chr1:97658537-97658688 | GAGGCACTGTGTTTCCTTTG |
| DPYD.4.2.DPYD.2_R | chr1:97658537-97658688 | CAAGACTGGGATGGACAGAG |
| DPYD.24.1.DPYD.1_F | chr1:98386339-98386481 | AAACTTTCCCGCGTCTCTC |
| DPYD.24.1.DPYD.1_R | chr1:98386339-98386481 | ACTCGAGACTGTAGGCACT |
| DPYD.19.1.DPYD.1_F | chr1:98186399-98186539 | ACCTGCTTGTTGACTCCAAA |
| DPYD.19.1.DPYD.1_R | chr1:98186399-98186539 | TGGAAGCAGGTCTTTTATTTGG |
| DPYD.10.3.DPYD.1_F | chr1:97915588-97915745 | TCACCAACTTATGCCAATTCTC |
| DPYD.10.3.DPYD.1_R | chr1:97915588-97915745 | TGTTTCCCCCAGAATCATCC |
| DPYD.10.4.DPYD.2_F | chr1:97915717-97915869 | AGAAAGGAGCTTTGTCCAGG |
| DPYD.10.4.DPYD.2_R | chr1:97915717-97915869 | AAATGTGAGAAGGGACCTCA |
| DPYD.5.3.DPYD.1_F | chr1:97700405-97700542 | GGGACAGACAAACACAATGAC |
| DPYD.5.3.DPYD.1_R | chr1:97700405-97700542 | TGATTTTGTGTAGGGACAGCA |
| DPYD.5.4.DPYD.2_F | chr1:97700478-97700625 | AGTCAATTCCACCAGTAGCC |
| DPYD.5.4.DPYD.2_R | chr1:97700478-97700625 | TGACGCTGTCATCACATCAT |
| PMS2.6.9.PMS2.1_F | chr7:6029420-6029576 | TTAGAAGCTGTTTGTACACTGT |
| PMS2.6.9.PMS2.1_R | chr7:6029420-6029576 | TTTGCCTTAGAATGCGTTGA |
| PMS2.6.10.PMS2.2_F | chr7:6029524-6029652 | GAGGTCTTTAAAACTGCCAACA |
| PMS2.6.10.PMS2.2_R | chr7:6029524-6029652 | AGTGAGACGCTGTCTGAAAA |
| PMS2.11.1.PMS2.1_F | chr7:6042150-6042287 | GAAAATAACTGCTGCACGCT |
| PMS2.11.1.PMS2.1_R | chr7:6042150-6042287 | GTCTCTCACTGAACACACCA |
| PMS2.11.2.PMS2.2_F | chr7:6042052-6042211 | TGCTCATGTGCATTAACCAA |
| PMS2.11.2.PMS2.2_R | chr7:6042052-6042211 | GGTTGGAACTCGACTGATGT |
| PMS2.3.8.PMS2.1_F | chr7:6018214-6018357 | ACCCAGCCGCTATAGTTCTA |
| PMS2.3.8.PMS2.1_R | chr7:6018214-6018357 | AGCTAAATGTTTGACTTCAGGA |
| PMS2.7.1.PMS2.1_F | chr7:6031601-6031739 | ACTGAAATGCCAATGGAACT |
| PMS2.7.1.PMS2.1_R | chr7:6031601-6031739 | AATGTTGTTAGAATGCGGGG |
| PMS2.9.1.PMS2.1_F | chr7:6036951-6037095 | AGTTCTCTTGCCAGCAATCT |
| PMS2.9.1.PMS2.1_R | chr7:6036951-6037095 | ACAGATTAAGTCCACTCTGTCT |
| PMS2.12.11.PMS2.1_F | chr7:6043303-6043440 | CTAAGGGGTCAAGTGAGTGG |
| PMS2.12.11.PMS2.1_R | chr7:6043303-6043440 | GCACTTGAGATATCTTAGTCCTTAC |
| PMS2.2.1.PMS2.1_F | chr7:6017258-6017415 | GAGGCAAACATCTGCTTGAC |
| PMS2.2.1.PMS2.1_R | chr7:6017258-6017415 | ACGTGTTTGTCAAGTCATGG |
| PMS2.2.2.PMS2.2_F | chr7:6017214-6017366 | TGACAGCCAGGCTTTCTTTA |
| PMS2.2.2.PMS2.2_R | chr7:6017214-6017366 | CCAGTCACTGAAAGGGCTAA |
| PMS2.14.12.PMS2.1_F | chr7:6045516-6045652 | TGTGGCTTAAAACTCTCCCA |
| PMS2.14.12.PMS2.1_R | chr7:6045516-6045652 | ACTGATTTCTCTAGTACAGAACCT |
| PMS2.15.1.PMS2.1_F | chr7:6048583-6048716 | AATGCCGTGGGTCTCAAAG |
| PMS2.15.1.PMS2.1_R | chr7:6048583-6048716 | CCAATGGGAGTTCAGGAGG |
| PMS2.8.13.PMS2.1_F | chr7:6035143-6035282 | AGTCAAAGGCATAAAGAACAAACT |
| PMS2.8.13.PMS2.1_R | chr7:6035143-6035282 | CTCTGGAATCCTGCCCTTAC |
| PMS2.4.6.PMS2.1_F | chr7:6022451-6022605 | TGGGAATGAACACTAAACACAC |
| PMS2.4.6.PMS2.1_R | chr7:6022451-6022605 | GCAGTAAAACGATGTTTGCAG |
| PMS2.4.7.PMS2.2_F | chr7:6022594-6022736 | TCCCAGGTTAAACTGACCAA |
| PMS2.4.7.PMS2.2_R | chr7:6022594-6022736 | AAAAGAAAGCGGGATGGCTA |
| PMS2.1.1.PMS2.1_F | chr7:6013037-6013174 | ACAGTGACTACGGTCAGTTC |
| PMS2.1.1.PMS2.1_R | chr7:6013037-6013174 | ACCATTGTGTCTCACACTCA |
| PMS2.1.2.PMS2.2_F | chr7:6012879-6013038 | GCAATGCTCCATCTGGTTTG |
| PMS2.1.2.PMS2.2_R | chr7:6012879-6013038 | CCAACCTGGGTGTCATTTCT |
| PMS2.10.6.PMS2.1_F | chr7:6038720-6038878 | AGAGCAATAAGAGGCGTTGA |
| PMS2.10.6.PMS2.1_R | chr7:6038720-6038878 | CCAAAATGGTCCAGGTCTTAC |
| PMS2.10.7.PMS2.2_F | chr7:6038845-6038988 | CAAGCTGATTGGTGCAACTT |
| PMS2.10.7.PMS2.2_R | chr7:6038845-6038988 | TGCGATGATGTGAGAACCTT |
| PMS2.13.1.PMS2.1_F | chr7:6043568-6043721 | TTCTGAGACATGTGACCCAA |
| PMS2.13.1.PMS2.1_R | chr7:6043568-6043721 | TGATAGCATGGGTCCGTTTT |
| PMS2.5.1.PMS2.1_F | chr7:6026500-6026659 | ACTTTGCTGTGCTTCATGATG |
| PMS2.5.1.PMS2.1_R | chr7:6026500-6026659 | CCCCAAACACAAAGCGTTT |
| PMS2.5.2.PMS2.2_F | chr7:6026679-6026839 | AAAACGCTTTGTGTTTGGGG |
| PMS2.5.2.PMS2.2_R | chr7:6026679-6026839 | CACTGCAGCAGCGAGTA |
| PMS2.5.3.PMS2.3_F | chr7:6026551-6026682 | ATAGAAAAGTCCAGGGGCAC |

| | | |
|---|---|---|
| PMS2.5.3.PMS2.3_R | chr7:6026551-6026682 | TGCCTCAGCCAACTAATCTC |
| PMS2.5.3.PMS2.4_F | chr7:6026860-6027021 | TCGCTGCTGCAGTGACT |
| PMS2.5.3.PMS2.4_R | chr7:6026860-6027021 | GGTATGCTGTCTTCTAGCACT |
| PMS2.5.4.PMS2.5_F | chr7:6026714-6026871 | GCTGAGGCAAAACTCGAAAT |
| PMS2.5.4.PMS2.5_R | chr7:6026714-6026871 | ATTCTGAGGGGTTCAGCATC |
| PMS2.5.4.PMS2.6_F | chr7:6027021-6027181 | TTGTCAGAGATGGCACCTG |
| PMS2.5.4.PMS2.6_R | chr7:6027021-6027181 | AAGCAGGATCAATCCCCTTC |
| PMS2.5.5.PMS2.7_F | chr7:6027115-6027268 | GGCTTGTTCTCTGTTGTGTG |
| PMS2.5.5.PMS2.7_R | chr7:6027115-6027268 | TAGTCCCTGACCCTCTTCTC |
| PTEN.6.1.PTEN.1_F | chr10:89711879-89712021 | TTTTTCTGTCCACCAGGGAG |
| PTEN.6.1.PTEN.1_R | chr10:89711879-89712021 | GGAAGGATGAGAATTTCAAGCA |
| PTEN.6.2.PTEN.2_F | chr10:89711800-89711952 | AATGGCTACGACCCAGTTAC |
| PTEN.6.2.PTEN.2_R | chr10:89711800-89711952 | TTGTGAAACAACAGTGCCAC |
| PTEN.3.1.PTEN.1_F | chr10:89685244-89685379 | GCTCATTTTTGTTAATGGTGGC |
| PTEN.3.1.PTEN.1_R | chr10:89685244-89685379 | ACCTCACTCTAACAAGCAGA |
| PTEN.7.3.PTEN.1_F | chr10:89717579-89717716 | ACAGTTAAAGGCATTTCCTGTG |
| PTEN.7.3.PTEN.1_R | chr10:89717579-89717716 | CTTTGATATCACCACACACAGG |
| PTEN.7.4.PTEN.2_F | chr10:89717717-89717877 | TGAGTTCCCTCAGCCGTTA |
| PTEN.7.4.PTEN.2_R | chr10:89717717-89717877 | GCAAAACACCTGCAGATCTA |
| PTEN.9.1.PTEN.1_F | chr10:89725084-89725240 | AACAGTAGAGGAGCCGTCAA |
| PTEN.9.1.PTEN.1_R | chr10:89725084-89725240 | CATGGTGTTTTATCCCTCTTGA |
| PTEN.9.5.PTEN.2_F | chr10:89724926-89725083 | AGATCATGTTTGTTACAGTGCT |
| PTEN.9.5.PTEN.2_R | chr10:89724926-89725083 | TGAACTGCTAGCCTCTGGAT |
| PTEN.2.6.PTEN.1_F | chr10:89653754-89654004 | AGTTTGATTGCTGCATATTTCAGA |
| PTEN.2.6.PTEN.1_R | chr10:89653754-89654004 | GTCCATTAGGTACGGTAAGCCA |
| PTEN.8.5.PTEN.1_F | chr10:89720757-89720884 | TGTGATCAAGAAATCGATAGCA |
| PTEN.8.5.PTEN.1_R | chr10:89720757-89720884 | CAACCCCCACAAAATGTTTAAT |
| PTEN.8.6.PTEN.2_F | chr10:89720570-89720758 | TGCAAATGTTTAACATAGGTGACAGA |
| PTEN.8.6.PTEN.2_R | chr10:89720570-89720758 | TCTGCACGCTCTATACTGCA |
| PTEN.1.1.PTEN.1_F | chr10:89624218-89624369 | CCTTTTTCTTCAGCCACAGG |
| PTEN.1.1.PTEN.1_R | chr10:89624218-89624369 | CTACGGACATTTTCGCATCC |
| PTEN.4.5.PTEN.1_F | chr10:89690736-89690884 | GATTCAGGCAATGTTTGTTAGT |
| PTEN.4.5.PTEN.1_R | chr10:89690736-89690884 | AGTAAGATACAGTCTATCGGGT |
| PTEN.5.1.PTEN.1_F | chr10:89692820-89692979 | CCACCACAGCTAGAACTTATC |
| PTEN.5.1.PTEN.1_R | chr10:89692820-89692979 | CTGGTCCTTACTTCCCCAT |
| PTEN.5.2.PTEN.2_F | chr10:89692906-89693035 | TGTAAAGCTGGAAAGGGACG |
| PTEN.5.2.PTEN.2_R | chr10:89692906-89693035 | TCTCAGATCCAGGAAGAGGA |
| PTEN.5.5.PTEN.3_F | chr10:89692693-89692829 | CCTGTTAAGTTTGTATGCAACA |
| PTEN.5.5.PTEN.3_R | chr10:89692693-89692829 | GCCATTGGTCAAGATCTTCAC |
| PDGFRB.11.1.PDGFRB.1_F | chr5:149504236-149504394 | GAGAGGCTAAGTGTGTGGG |
| PDGFRB.11.1.PDGFRB.1_R | chr5:149504236-149504394 | ATGGATCATCTCTGCACCAG |
| PDGFRB.21.1.PDGFRB.1_F | chr5:149515128-149515287 | GCCCTTACCTGGCACAAA |
| PDGFRB.21.1.PDGFRB.1_R | chr5:149515128-149515287 | GTGGTGTGGGAACGGAT |
| PDGFRB.21.2.PDGFRB.2_F | chr5:149515281-149515443 | ATTTCCTGTGGGGGCTC |
| PDGFRB.21.2.PDGFRB.2_R | chr5:149515281-149515443 | TCTGATACCTCCTCTCCCAA |
| PDGFRB.21.2.PDGFRB.3_F | chr5:149515031-149515187 | TAGGATGGCTGCATTTCACA |
| PDGFRB.21.2.PDGFRB.3_R | chr5:149515031-149515187 | GGCTAGACACGGGAGAATAC |
| PDGFRB.7.1.PDGFRB.1_F | chr5:149500766-149500898 | CCACCAACACCACACGTA |
| PDGFRB.7.1.PDGFRB.1_R | chr5:149500766-149500898 | AAGATAGCAGGTGACCCTCT |
| PDGFRB.2.1.PDGFRB.1_F | chr5:149497260-149497422 | AGGCAGGGGGATGATATAGT |
| PDGFRB.2.1.PDGFRB.1_R | chr5:149497260-149497422 | TTTGGTTCTCCCTGCCC |
| PDGFRB.2.2.PDGFRB.2_F | chr5:149497157-149497287 | ACACACTGTGCACAATTTCC |
| PDGFRB.2.2.PDGFRB.2_R | chr5:149497157-149497287 | CCAGCTCCGTCCTCTATACT |
| PDGFRB.17.1.PDGFRB.1_F | chr5:149512392-149512546 | GTGCGGTTGTCTTTGAACC |
| PDGFRB.17.1.PDGFRB.1_R | chr5:149512392-149512546 | CTTTGGGATTCAGTTGGTGC |
| PDGFRB.17.2.PDGFRB.2_F | chr5:149512302-149512445 | CAAGAATGGGATGGGAGAGC |
| PDGFRB.17.2.PDGFRB.2_R | chr5:149512302-149512445 | ACAATTTGCTGAGCTGCATC |
| PDGFRB.22.1.PDGFRB.1_F | chr5:149516460-149516610 | AACTAAAGCTGAAGGGCAGG |
| PDGFRB.22.1.PDGFRB.1_R | chr5:149516460-149516610 | GTTTAACTCTGCAGGACACC |
| PDGFRB.1.1.PDGFRB.1_F | chr5:149495351-149495509 | GGAAGCTATCCTCTGCTTCC |
| PDGFRB.1.1.PDGFRB.1_R | chr5:149495351-149495509 | CTCACTGCCTCTTTCCTCTAG |
| PDGFRB.1.2.PDGFRB.2_F | chr5:149495228-149495371 | GAAGGGGACAGCTGATAAGG |
| PDGFRB.1.2.PDGFRB.2_R | chr5:149495228-149495371 | TGGAACAGTTGCCGGATTC |
| PDGFRB.18.1.PDGFRB.1_F | chr5:149513156-149513319 | AGGCACACACCAACCAC |
| PDGFRB.18.1.PDGFRB.1_R | chr5:149513156-149513319 | AACCTGTTGCTGCAGAGTG |
| PDGFRB.18.2.PDGFRB.2_F | chr5:149513304-149513440 | CCAAGAGGAAGTCAGTCACC |
| PDGFRB.18.2.PDGFRB.2_R | chr5:149513304-149513440 | CATACCCCCGCAAAGAAGTA |

| | | |
|---|---|---|
| PDGFRB.18.2.PDGFRB.3_F | chr5:149513130-149513283 | TGAAGGAATTGGGGATTGGG |
| PDGFRB.18.2.PDGFRB.3_R | chr5:149513130-149513283 | GGTGACTGACTTCCTCTTGG |
| PDGFRB.13.1.PDGFRB.1_F | chr5:149506032-149506180 | GGCAGAGGGGTGGAATTTAT |
| PDGFRB.13.1.PDGFRB.1_R | chr5:149506032-149506180 | CTGCTCTCATCTCTGCCTC |
| PDGFRB.16.1.PDGFRB.1_F | chr5:149511523-149511685 | AAGGAGCAGGGACATGG |
| PDGFRB.16.1.PDGFRB.1_R | chr5:149511523-149511685 | ACTGTAAGAGTCCTTCCGAC |
| PDGFRB.6.1.PDGFRB.1_F | chr5:149500430-149500595 | ACTGTGACTGCCCTGCA |
| PDGFRB.6.1.PDGFRB.1_R | chr5:149500430-149500595 | TTGTCCCCAAGGCCTCA |
| PDGFRB.3.1.PDGFRB.1_F | chr5:149498261-149498418 | CCTTGTACTCGGTGTCTGAC |
| PDGFRB.3.1.PDGFRB.1_R | chr5:149498261-149498418 | CTGACCCTTCTCCCTATCCC |
| PDGFRB.9.1.PDGFRB.1_F | chr5:149502645-149502788 | GGCAGAGCATTGCTGTAGA |
| PDGFRB.9.1.PDGFRB.1_R | chr5:149502645-149502788 | TGAGGCCTCCATAGGGACT |
| PDGFRB.9.2.PDGFRB.2_F | chr5:149502577-149502733 | GCTCCTCAGGTATCCCAAAG |
| PDGFRB.9.2.PDGFRB.2_R | chr5:149502577-149502733 | ATCATCACTGAGTACTGCCG |
| PDGFRB.12.1.PDGFRB.1_F | chr5:149505004-149505145 | ACATGAGGCCTCTCAGGAC |
| PDGFRB.12.1.PDGFRB.1_R | chr5:149505004-149505145 | CTAATTCCTGGGGTTGGTCC |
| PDGFRB.14.1.PDGFRB.1_F | chr5:149509329-149509490 | GGACTCACAGTGTGGCA |
| PDGFRB.14.1.PDGFRB.1_R | chr5:149509329-149509490 | GCTGCTGGGGAACAGTT |
| PDGFRB.14.2.PDGFRB.2_F | chr5:149509467-149509623 | CTCCCAGTACGTCACGTTAG |
| PDGFRB.14.2.PDGFRB.2_R | chr5:149509467-149509623 | CAGTGGGACATAGGAACTGG |
| PDGFRB.14.2.PDGFRB.3_F | chr5:149509267-149509420 | GGGCACATTACCAATTAGGC |
| PDGFRB.14.2.PDGFRB.3_R | chr5:149509267-149509420 | CAGGAGTTTGAGGTGGTGAG |
| PDGFRB.15.1.PDGFRB.1_F | chr5:149510070-149510228 | TTTTTCTAGCCAGCTGGGG |
| PDGFRB.15.1.PDGFRB.1_R | chr5:149510070-149510228 | AACTCAGTGCTCTCTCCATG |
| PDGFRB.20.1.PDGFRB.1_F | chr5:149514279-149514438 | TCTGTGGAGGGTTATACTTGC |
| PDGFRB.20.1.PDGFRB.1_R | chr5:149514279-149514438 | AAGAAAGGGGACGTTGCAC |
| PDGFRB.20.2.PDGFRB.2_F | chr5:149514428-149514585 | AAGCCACGTTGGTGATCATA |
| PDGFRB.20.2.PDGFRB.2_R | chr5:149514428-149514585 | TTTATTCACGGCTCCACTCC |
| PDGFRB.8.1.PDGFRB.1_F | chr5:149501443-149501603 | ATGTCACTATGTCCACCCAC |
| PDGFRB.8.1.PDGFRB.1_R | chr5:149501443-149501603 | GATGCTCCTTTACCCCCAG |
| PDGFRB.4.1.PDGFRB.1_F | chr5:149499030-149499187 | ATGCATGAGACTCCACTCAC |
| PDGFRB.4.1.PDGFRB.1_R | chr5:149499030-149499187 | CCTTGTCCTGTAGAAGAGGG |
| PDGFRB.19.1.PDGFRB.1_F | chr5:149513443-149513581 | TGCCTGGCCCCACATTA |
| PDGFRB.19.1.PDGFRB.1_R | chr5:149513443-149513581 | CCCAGACCTAAGCCAATCTC |
| PDGFRB.10.1.PDGFRB.1_F | chr5:149503781-149503924 | CTTGGTGGTGGGCACTTT |
| PDGFRB.10.1.PDGFRB.1_R | chr5:149503781-149503924 | GGCCCCCATTTTCCTCA |
| PDGFRB.5.1.PDGFRB.1_F | chr5:149499532-149499686 | CATGAGTGGTCGAGGTAGAC |
| PDGFRB.5.1.PDGFRB.1_R | chr5:149499532-149499686 | TGTCCTGACCTCCCTACAG |
| MSH6.6.1.MSH6.1_F | chr2:48032046-48032181 | GACCTTTTCCTCCCTCATTCA |
| MSH6.6.1.MSH6.1_R | chr2:48032046-48032181 | ACTGAATGAGAACTTAAGTGGG |
| MSH6.3.1.MSH6.1_F | chr2:48023053-48023210 | AGGTTCAAAATCAAAGGAAGCC |
| MSH6.3.1.MSH6.1_R | chr2:48023053-48023210 | TAACAACTGAATGCTTGCCG |
| MSH6.3.2.MSH6.2_F | chr2:48023015-48023158 | ACCCGGCCCTTATTGTTTAT |
| MSH6.3.2.MSH6.2_R | chr2:48023015-48023158 | CTCTGAGGGCTCATCACAAA |
| MSH6.7.19.MSH6.1_F | chr2:48032629-48032849 | GCCCGGCCAATAATTGCATA |
| MSH6.7.19.MSH6.1_R | chr2:48032629-48032849 | AGTCTTCAAATGAGAAGTTTAATGTCT |
| MSH6.9.1.MSH6.1_F | chr2:48033681-48033840 | GGGAGCTTGTCCTAAAAGC |
| MSH6.9.1.MSH6.1_R | chr2:48033681-48033840 | CCTTCCCCTTTTACTGTTTCT |
| MSH6.9.2.MSH6.2_F | chr2:48033572-48033701 | TTGCTAGCACATGTATCGCT |
| MSH6.9.2.MSH6.2_R | chr2:48033572-48033701 | TCCTCTGGGAGATTAGCAAG |
| MSH6.2.1.MSH6.1_F | chr2:48018106-48018267 | TTGGTTTGGGCCAAGATG |
| MSH6.2.1.MSH6.1_R | chr2:48018106-48018267 | CACACATGGCAGTAGTGACT |
| MSH6.2.2.MSH6.2_F | chr2:48017991-48018120 | TGCCAGAAGACTTGGAATTG |
| MSH6.2.2.MSH6.2_R | chr2:48017991-48018120 | TTGTAAACCAGACAAGGCCA |
| MSH6.8.1.MSH6.1_F | chr2:48033392-48033551 | GCAATAGCAAATGCAGTTGT |
| MSH6.8.1.MSH6.1_R | chr2:48033392-48033551 | AGCGATACATGTGCTAGCAA |
| MSH6.8.16.MSH6.2_F | chr2:48033268-48033400 | CCGATGTTGCTTTTCTGTCC |
| MSH6.8.16.MSH6.2_R | chr2:48033268-48033400 | ACGACATTTTATAGTCTCAGCA |
| MSH6.4.1.MSH6.1_F | chr2:48025760-48025919 | CTGGCAGGTAGGCACAA |
| MSH6.4.1.MSH6.1_R | chr2:48025760-48025919 | CTTCCTCCTTAGTGTCTGGC |
| MSH6.4.2.MSH6.2_F | chr2:48025918-48026079 | GTGGCTCTGATGTGGAATTT |
| MSH6.4.2.MSH6.2_R | chr2:48025918-48026079 | TTGTTTGGTGGCTGAGGG |
| MSH6.4.2.MSH6.3_F | chr2:48025748-48025881 | ATACTCTTTCCTTGCCTGGC |
| MSH6.4.2.MSH6.3_R | chr2:48025748-48025881 | CCACCAATGTCACTCTCAGA |
| MSH6.4.3.MSH6.4_F | chr2:48026088-48026249 | AAGGAAACGCCCTCAGC |
| MSH6.4.3.MSH6.4_R | chr2:48026088-48026249 | CTGTGCTCATCTCTTCTCTTT |
| MSH6.4.4.MSH6.5_F | chr2:48026052-48026190 | GTGACTGGAAATGGCTCTCT |

| | | |
|---|---|---|
| MSH6.4.4.MSH6.5_R | chr2:48026052-48026190 | GTAGGGCGACTACTGTCATC |
| MSH6.4.4.MSH6.6_F | chr2:48026286-48026450 | AGGAGGAGGCCTGATCA |
| MSH6.4.4.MSH6.6_R | chr2:48026286-48026450 | AGCCCCAGTTCACTGACT |
| MSH6.4.5.MSH6.7_F | chr2:48026211-48026351 | GATGACAGTAGTCGCCCTAC |
| MSH6.4.5.MSH6.7_R | chr2:48026211-48026351 | TAATCTGCCACCACTTCCTC |
| MSH6.4.5.MSH6.8_F | chr2:48026470-48026630 | TCAGTGAACTGGGGCTG |
| MSH6.4.5.MSH6.8_R | chr2:48026470-48026630 | CCTCACCACTCTATCATACTTG |
| MSH6.4.6.MSH6.9_F | chr2:48026441-48026592 | TGAGCTGTACCACATGGATG |
| MSH6.4.6.MSH6.9_R | chr2:48026441-48026592 | CTACATCGTGCCTCCATCAT |
| MSH6.4.6.MSH6.10_F | chr2:48026711-48026870 | TACAGTGTGCTGGAAGGTG |
| MSH6.4.6.MSH6.10_R | chr2:48026711-48026870 | GGATAGTGTGCCACTAGAGTC |
| MSH6.4.7.MSH6.11_F | chr2:48026613-48026765 | ATGATGGAGGCACGATGTAG |
| MSH6.4.7.MSH6.11_R | chr2:48026613-48026765 | CACGAGTATGGCCAGAAGAA |
| MSH6.4.7.MSH6.12_F | chr2:48026900-48027060 | GCACACTATCCCCCAGTAC |
| MSH6.4.7.MSH6.12_R | chr2:48026900-48027060 | ACCCCAATGCCATCACTTAG |
| MSH6.4.8.MSH6.13_F | chr2:48026863-48026997 | CAGATGATCGCCATTGTTCG |
| MSH6.4.8.MSH6.13_R | chr2:48026863-48026997 | TTGGATGCATCCCAAAACTG |
| MSH6.4.8.MSH6.14_F | chr2:48027090-48027250 | GCATTGGGGTGATGTTACC |
| MSH6.4.8.MSH6.14_R | chr2:48027090-48027250 | TGTGTCAGAATCCAAGGGAA |
| MSH6.4.9.MSH6.15_F | chr2:48027018-48027168 | CAGTTTTGGGATGCATCCAA |
| MSH6.4.9.MSH6.15_R | chr2:48027018-48027168 | AAGACACAACCACCTAGAGC |
| MSH6.4.9.MSH6.16_F | chr2:48027271-48027430 | TTCCCTTGGATTCTGACACA |
| MSH6.4.9.MSH6.16_R | chr2:48027271-48027430 | GCTTTAGGAGCCGCTTAC |
| MSH6.4.10.MSH6.17_F | chr2:48027183-48027312 | CTCTCTGCTCTAGGTGGTTG |
| MSH6.4.10.MSH6.17_R | chr2:48027183-48027312 | GCATCTAGCACCATTCGTTG |
| MSH6.4.10.MSH6.18_F | chr2:48027470-48027633 | GCTTTGTGCCCCACTCT |
| MSH6.4.10.MSH6.18_R | chr2:48027470-48027633 | TTATAGCCCTGCTGTCTGG |
| MSH6.4.11.MSH6.19_F | chr2:48027420-48027563 | GAGAGGGTTGATACTTGCCA |
| MSH6.4.11.MSH6.19_R | chr2:48027420-48027563 | GCCTCTCAAGATCTGGAAGC |
| MSH6.4.11.MSH6.20_F | chr2:48027654-48027813 | CCAGACAGCAGGGCTATAAT |
| MSH6.4.11.MSH6.20_R | chr2:48027654-48027813 | TCAGGAAAACGACCTTCAGG |
| MSH6.4.12.MSH6.21_F | chr2:48027536-48027694 | CATGGTTGTGCCTGACAAAA |
| MSH6.4.12.MSH6.21_R | chr2:48027536-48027694 | TCCTTCCAGAGCAGAAAGAA |
| MSH6.4.12.MSH6.22_F | chr2:48027834-48027993 | CCTGAAGGTCGTTTTCCTGA |
| MSH6.4.12.MSH6.22_R | chr2:48027834-48027993 | GCCAATTCTGTTGCGCTG |
| MSH6.4.13.MSH6.23_F | chr2:48027766-48027910 | TGGAAGAAGTTGCTGATGGT |
| MSH6.4.13.MSH6.23_R | chr2:48027766-48027910 | ATCAGAGTCAAAGCCTGCTT |
| MSH6.4.13.MSH6.24_F | chr2:48028014-48028173 | GCGCAACAGAATTGGCTG |
| MSH6.4.13.MSH6.24_R | chr2:48028014-48028173 | TCCGTTCTTCAGCATTTATGAG |
| MSH6.4.14.MSH6.25_F | chr2:48027921-48028052 | ATTACTCCCAAAGCAGGCTT |
| MSH6.4.14.MSH6.25_R | chr2:48027921-48028052 | TCTCAGGAATTTCCAGCTGG |
| MSH6.4.14.MSH6.26_F | chr2:48028199-48028329 | AAATGCTGAAGAACGGAGGG |
| MSH6.4.14.MSH6.26_R | chr2:48028199-48028329 | CTGGCAAACAGCACTACTTA |
| MSH6.4.15.MSH6.27_F | chr2:48028073-48028218 | CCAGCTGGAAATTCCTGAGA |
| MSH6.4.15.MSH6.27_R | chr2:48028073-48028218 | TTATAGAACAGTCGCCGCAT |
| MSH6.1.1.MSH6.1_F | chr2:48010412-48010572 | TGTACAGCTTCTTCCCCAAG |
| MSH6.1.1.MSH6.1_R | chr2:48010412-48010572 | GTTGAGGTTCTTCGCCTTG |
| MSH6.1.16.MSH6.2_F | chr2:48010250-48010413 | TGTTGATTGGCCACTGGG |
| MSH6.1.16.MSH6.2_R | chr2:48010250-48010413 | ATCACTCAGCGCCGGA |
| MSH6.1.17.MSH6.3_F | chr2:48010509-48010654 | TCTCCTTCCCCAGGCG |
| MSH6.1.17.MSH6.3_R | chr2:48010509-48010654 | CTATGCCCCCGCCTTC |
| MSH6.10.1.MSH6.1_F | chr2:48033875-48034024 | GGGAAGGGATGATGCACTAT |
| MSH6.10.1.MSH6.1_R | chr2:48033875-48034024 | ACCACCTTTGTCAGAAGTCA |
| MSH6.5.1.MSH6.1_F | chr2:48030579-48030738 | ATGTTTTACTGTGCCTGGCT |
| MSH6.5.1.MSH6.1_R | chr2:48030579-48030738 | ATTTTCCTGCTCCTCTTCCT |
| MSH6.5.2.MSH6.2_F | chr2:48030526-48030669 | GCTGATAAAACCCCCAAACG |
| MSH6.5.2.MSH6.2_R | chr2:48030526-48030669 | CTTCGTAATGCAAGGATGGC |
| MSH6.5.18.MSH6.3_F | chr2:48030739-48030887 | CCTAATGACATTCTAATAGGCTGTG |
| MSH6.5.18.MSH6.3_R | chr2:48030739-48030887 | CTGTGTTTGGAAAATGATCACC |
| JAK3.11.1.JAK3.1_F | chr19:17946732-17946891 | AGGCAGAGGAGCACTCA |
| JAK3.11.1.JAK3.1_R | chr19:17946732-17946891 | ACACAAGTGCAGTTCAGTCT |
| JAK3.21.1.JAK3.1_F | chr19:17953846-17953994 | GGCTCTCACCTGACAGTCT |
| JAK3.21.1.JAK3.1_R | chr19:17953846-17953994 | TCAGCTGATGCTCCCTGT |
| JAK3.21.2.JAK3.2_F | chr19:17953750-17953911 | GAAAGCTTGCAGGAGAACTC |
| JAK3.21.2.JAK3.2_R | chr19:17953750-17953911 | AGCAGGGTGAGTGTCTCA |
| JAK3.7.1.JAK3.1_F | chr19:17943604-17943750 | TGCCCACCTTACCTTGC |
| JAK3.7.1.JAK3.1_R | chr19:17943604-17943750 | TGCTTACCACTGCCCATG |

| | | |
|---|---|---|
| JAK3.7.2.JAK3.2_F | chr19:17943478-17943623 | GCACCTGTATTGTCGCCTA |
| JAK3.7.2.JAK3.2_R | chr19:17943478-17943623 | ATCTTCGAGGAGAGACACCT |
| JAK3.17.1.JAK3.1_F | chr19:17951001-17951156 | ATTCAAACGTCACCTCCTCC |
| JAK3.17.1.JAK3.1_R | chr19:17951001-17951156 | CATTCCCTCTCACCTTCCC |
| JAK3.2.1.JAK3.1_F | chr19:17940839-17940998 | CCTTTCTTCTCAGTACAGAGAC |
| JAK3.2.1.JAK3.1_R | chr19:17940839-17940998 | GATGATGGGATGTGAGCG |
| JAK3.2.2.JAK3.2_F | chr19:17940902-17941034 | GACTGGGAAACTGAGGCTAG |
| JAK3.2.2.JAK3.2_R | chr19:17940902-17941034 | ACATGCGCTCCTCCTTG |
| JAK3.22.1.JAK3.1_F | chr19:17954184-17954315 | TCAAACCCCAGGCAGAAC |
| JAK3.22.1.JAK3.1_R | chr19:17954184-17954315 | CACTTTCATCCCAGGGTTCT |
| JAK3.1.1.JAK3.1_F | chr19:17937562-17937725 | GGGCAGGAGCTATGAAAAG |
| JAK3.1.1.JAK3.1_R | chr19:17937562-17937725 | ATTATCTGTCCCCCGCC |
| JAK3.1.2.JAK3.2_F | chr19:17937459-17937593 | TCCTGAAGTAGAGGACCCTC |
| JAK3.1.2.JAK3.2_R | chr19:17937459-17937593 | GTGAGACTCATGCCTTCACT |
| JAK3.18.1.JAK3.1_F | chr19:17952207-17952369 | AGGTCCTTACGTGATGGGG |
| JAK3.18.1.JAK3.1_R | chr19:17952207-17952369 | TTTCACTGGGCTCTGACG |
| JAK3.18.2.JAK3.2_F | chr19:17952095-17952250 | GCAGGTGTGGTTTGAAAACT |
| JAK3.18.2.JAK3.2_R | chr19:17952095-17952250 | ACTTCTTCTGCAAGGAGGTG |
| JAK3.23.1.JAK3.1_F | chr19:17954568-17954726 | AATTGTACAATCCCTGGGGG |
| JAK3.23.1.JAK3.1_R | chr19:17954568-17954726 | GACTCAGGAGGGAGCTGAT |
| JAK3.13.1.JAK3.1_F | chr19:17948729-17948872 | GACTCCAAAAGTCTGGTCCA |
| JAK3.13.1.JAK3.1_R | chr19:17948729-17948872 | CTGTCCCCTCACCATTCAG |
| JAK3.16.1.JAK3.1_F | chr19:17950292-17950455 | AAGGGGCTCACCTTTGG |
| JAK3.16.1.JAK3.1_R | chr19:17950292-17950455 | AACCCCCTTGGTCCTGA |
| JAK3.16.2.JAK3.2_F | chr19:17950422-17950561 | CCAACCAGAAGGAAGGTTCC |
| JAK3.16.2.JAK3.2_R | chr19:17950422-17950561 | CCAGACTCCGTCTCCAAAAA |
| JAK3.16.2.JAK3.3_F | chr19:17950268-17950401 | CAGATCAGCCACTCATTCCA |
| JAK3.16.2.JAK3.3_R | chr19:17950268-17950401 | GGAACCTTCCTTCTGGTTGG |
| JAK3.6.1.JAK3.1_F | chr19:17943348-17943508 | GGCCATAGCTGACACCA |
| JAK3.6.1.JAK3.1_R | chr19:17943348-17943508 | TATCCCCCAGGGCAACTTT |
| JAK3.6.2.JAK3.2_F | chr19:17943478-17943623 | GCACCTGTATTGTCGCCTA |
| JAK3.6.2.JAK3.2_R | chr19:17943478-17943623 | ATCTTCGAGGAGAGACACCT |
| JAK3.6.2.JAK3.3_F | chr19:17943243-17943387 | TAATCACGTTCCCAGCCTAC |
| JAK3.6.2.JAK3.3_R | chr19:17943243-17943387 | CTTTCAGCGGGAGATTCAGA |
| JAK3.3.1.JAK3.1_F | chr19:17941288-17941436 | GTGGAGGGAGAAGAAGGCT |
| JAK3.3.1.JAK3.1_R | chr19:17941288-17941436 | CATCTCAAGACCTTGTCCCC |
| JAK3.9.1.JAK3.1_F | chr19:17945666-17945822 | TGAGGGGCTCTGACCTTAG |
| JAK3.9.1.JAK3.1_R | chr19:17945666-17945822 | GGATCCCTGATCCCACTTTC |
| JAK3.9.2.JAK3.2_F | chr19:17945559-17945699 | GAGAGGAGCAGTCGGTAATC |
| JAK3.9.2.JAK3.2_R | chr19:17945559-17945699 | CGGTCTGGGAAGTGTTTAGT |
| JAK3.12.1.JAK3.1_F | chr19:17947883-17948031 | CTCAGAACAGAGGTGGGAAG |
| JAK3.12.1.JAK3.1_R | chr19:17947883-17948031 | CATCTTCTCTCCCTTCCCAC |
| JAK3.20.3.JAK3.1_F | chr19:17953235-17953400 | CACGTGGAAGGTCTCGG |
| JAK3.20.3.JAK3.1_R | chr19:17953235-17953400 | ACAAGGCCTGCCTACCC |
| JAK3.20.4.JAK3.2_F | chr19:17953383-17953536 | AAGCTCAGGCCCTGGAT |
| JAK3.20.4.JAK3.2_R | chr19:17953383-17953536 | AATGAGAGTCTGTGTGTGCC |
| JAK3.20.5.JAK3.3_F | chr19:17953135-17953267 | GCCCTCACCTCCTGTTCT |
| JAK3.20.5.JAK3.3_R | chr19:17953135-17953267 | GCTCATGGCCAAGTACATCA |
| JAK3.20.6.JAK3.4_F | chr19:17953065-17953216 | ACATCCCCTACCACTCTCC |
| JAK3.20.6.JAK3.4_R | chr19:17953065-17953216 | CGAGACCTTCCACGTGG |
| JAK3.14.1.JAK3.1_F | chr19:17949072-17949204 | CATTTCCCAGGGCCTCTTAC |
| JAK3.14.1.JAK3.1_R | chr19:17949072-17949204 | TTTCTCCCTCATCCTCTCCC |
| JAK3.15.1.JAK3.1_F | chr19:17949969-17950107 | CGGTTCTACCAGGGATTCTG |
| JAK3.15.1.JAK3.1_R | chr19:17949969-17950107 | AACCTTTAGGTCTGGCTTGA |
| JAK3.8.1.JAK3.1_F | chr19:17945394-17945538 | GGCACCTGAAGAGATGAGG |
| JAK3.8.1.JAK3.1_R | chr19:17945394-17945538 | GATTACCGACTGCTCCTCTC |
| JAK3.8.2.JAK3.2_F | chr19:17945346-17945505 | CTGGGCCCAATATGACATCA |
| JAK3.8.2.JAK3.2_R | chr19:17945346-17945505 | CCAATTTTATGAGGACCGGC |
| JAK3.4.1.JAK3.1_F | chr19:17942084-17942232 | CGGACCACGTAGTAGTCTTT |
| JAK3.4.1.JAK3.1_R | chr19:17942084-17942232 | TATCCTGGGATCCTCTCTGC |
| JAK3.4.2.JAK3.2_F | chr19:17941927-17942086 | AAGCTGCAGCGGAGGAA |
| JAK3.4.2.JAK3.2_R | chr19:17941927-17942086 | CTAAGCTGCTGCCGCTT |
| JAK3.24.1.JAK3.1_F | chr19:17955043-17955202 | CCACTAGGGATGCACTCAC |
| JAK3.24.1.JAK3.1_R | chr19:17955043-17955202 | CACCTCCAAGTGAAGAGACG |
| JAK3.24.2.JAK3.2_F | chr19:17955199-17955351 | CATGAACGCTGAGGGATCA |
| JAK3.24.2.JAK3.2_R | chr19:17955199-17955351 | CCTCCCAAAATGCTGTGATT |
| JAK3.10.1.JAK3.1_F | chr19:17945892-17946032 | CTCCACCTCCAGGAACTTAC |

| | | |
|---|---|---|
| JAK3.10.1.JAK3.1_R | chr19:17945892-17946032 | GCATTTTCCTGTGTCTGGC |
| JAK3.19.1.JAK3.1_F | chr19:17952457-17952618 | TCCTGCACCCACTAAAATCT |
| JAK3.19.1.JAK3.1_R | chr19:17952457-17952618 | GTTCATGGGCGTGGTTTG |
| JAK3.19.2.JAK3.2_F | chr19:17952390-17952537 | GTCAGAGCCCAGTGAAACC |
| JAK3.19.2.JAK3.2_R | chr19:17952390-17952537 | CTGCGACTTTCCAGAAATCG |
| JAK3.5.1.JAK3.1_F | chr19:17942463-17942610 | CAGCTGAATCCCCACAAGT |
| JAK3.5.1.JAK3.1_R | chr19:17942463-17942610 | GTGACAGATCCTGCCTTCTC |
| TOP1.11.1.TOP1.1_F | chr20:39726838-39726995 | CGTTGTTTTTCTTTCATCTCCC |
| TOP1.11.1.TOP1.1_R | chr20:39726838-39726995 | TCCAGAATTCTCCAGAGGTT |
| TOP1.21.1.TOP1.1_F | chr20:39751806-39751943 | CCATTGCTGAGTCACCCTAA |
| TOP1.21.1.TOP1.1_R | chr20:39751806-39751943 | CACAGAACTCTGCCTCTTGA |
| TOP1.7.1.TOP1.1_F | chr20:39709736-39709889 | GGAGGCATCCAAGAGTTTGT |
| TOP1.7.1.TOP1.1_R | chr20:39709736-39709889 | CCCCAAAGGACCTAAGTCTC |
| TOP1.17.1.TOP1.1_F | chr20:39744916-39745071 | TCTGTTGCTTTGTCTCCTCC |
| TOP1.17.1.TOP1.1_R | chr20:39744916-39745071 | CAAGGTCACCGGATACCATT |
| TOP1.2.1.TOP1.1_F | chr20:39658081-39658244 | TTTTTCCAGATCGAAGCGG |
| TOP1.2.1.TOP1.1_R | chr20:39658081-39658244 | AAAGGGGGTCAAGAGGC |
| TOP1.2.2.TOP1.2_F | chr20:39658245-39658404 | ATTGTTCCCATCGGGCC |
| TOP1.2.2.TOP1.2_R | chr20:39658245-39658404 | AAAAGAAAATGGCGCAGAGA |
| TOP1.2.2.TOP1.3_F | chr20:39657932-39658086 | AAGTTACAGTTCGAGGCAGG |
| TOP1.2.2.TOP1.3_R | chr20:39657932-39658086 | GGCACACTCACCATTCAATC |
| TOP1.1.5.TOP1.1_F | chr20:39657668-39657832 | TCTGCGTCTCCCCCAC |
| TOP1.1.5.TOP1.1_R | chr20:39657668-39657832 | CTCCGGCCTGTCAAAGC |
| TOP1.18.1.TOP1.1_F | chr20:39746797-39746950 | ACCCTCTTGCTACCATGTTC |
| TOP1.18.1.TOP1.1_R | chr20:39746797-39746950 | GCAGACACAGTTCCCTTCAT |
| TOP1.16.1.TOP1.1_F | chr20:39743950-39744092 | GCAAGTTTCTTTAAGTTTGGGG |
| TOP1.16.1.TOP1.1_R | chr20:39743950-39744092 | CTCCCTTCAACATTGTGTGC |
| TOP1.13.1.TOP1.1_F | chr20:39729841-39729999 | CACTATCCTCGTGCTCTGTT |
| TOP1.13.1.TOP1.1_R | chr20:39729841-39729999 | AGTGCAGCTCTCAACTATCC |
| TOP1.6.6.TOP1.1_F | chr20:39708710-39708846 | CTAGCACTCTGACCAGCAAT |
| TOP1.6.6.TOP1.1_R | chr20:39708710-39708846 | AACTCCTCCTTTTTGTTTTCAAA |
| TOP1.3.1.TOP1.1_F | chr20:39689978-39690132 | TGACCAGCTTGTCAAGGTAG |
| TOP1.3.1.TOP1.1_R | chr20:39689978-39690132 | ACTTGCTTGATTCCACCCTT |
| TOP1.9.1.TOP1.1_F | chr20:39721104-39721255 | AAATGAGGCTGTGCTTTGTC |
| TOP1.9.1.TOP1.1_R | chr20:39721104-39721255 | GCAGATTTCTTTTCCTGGCC |
| TOP1.12.3.TOP1.1_F | chr20:39728696-39728853 | ACTTTCTTGCTGTCTTCCAG |
| TOP1.12.3.TOP1.1_R | chr20:39728696-39728853 | GTTGATGATTATATCCTCGGGC |
| TOP1.12.4.TOP1.2_F | chr20:39728852-39728996 | GCATGCTGAAGAGACGAATC |
| TOP1.12.4.TOP1.2_R | chr20:39728852-39728996 | CGCATGAAATACTGTCCTGC |
| TOP1.15.1.TOP1.1_F | chr20:39742620-39742779 | TGGGCAGCTTGCTCTGA |
| TOP1.15.1.TOP1.1_R | chr20:39742620-39742779 | CTTACTCGTTTCTCAACAGGG |
| TOP1.15.2.TOP1.2_F | chr20:39742753-39742899 | GAGTTTGACTTCCTCGGGAA |
| TOP1.15.2.TOP1.2_R | chr20:39742753-39742899 | ATGTTGCCTGTCTCAAGTCA |
| TOP1.15.2.TOP1.3_F | chr20:39742478-39742633 | GCTTGCAGTGAGCCAAAATT |
| TOP1.15.2.TOP1.3_R | chr20:39742478-39742633 | GCTGTTTCTCCTTCCTCCTT |
| TOP1.14.1.TOP1.1_F | chr20:39741418-39741575 | ACATTTGGGTTTGGGTCTTT |
| TOP1.14.1.TOP1.1_R | chr20:39741418-39741575 | TAGACAATGCCGATGGGAAG |
| TOP1.20.1.TOP1.1_F | chr20:39750636-39750795 | TGGTTCTTGAGGACTTTGCT |
| TOP1.20.1.TOP1.1_R | chr20:39750636-39750795 | AAGGAGGGCTCAACACTTAC |
| TOP1.8.1.TOP1.1_F | chr20:39713058-39713208 | AAAACTGCCTCCAGAAGTGT |
| TOP1.8.1.TOP1.1_R | chr20:39713058-39713208 | ACCCAGTGCTGAGATGTTAC |
| TOP1.4.1.TOP1.1_F | chr20:39704792-39704947 | CTACTCTGGGCTAACGCTTT |
| TOP1.4.1.TOP1.1_R | chr20:39704792-39704947 | CGTTCCTTCCTCCCCTACTA |
| TOP1.10.1.TOP1.1_F | chr20:39725848-39725981 | ACCTCACTTTTTGGAACCAC |
| TOP1.10.1.TOP1.1_R | chr20:39725848-39725981 | TTAACACATGGGCTACAGTAC |
| TOP1.19.1.TOP1.1_F | chr20:39750296-39750453 | TCTCAGGTGGAGCCATTTTT |
| TOP1.19.1.TOP1.1_R | chr20:39750296-39750453 | CTTTTCTCTACCAGCCCCAA |
| TOP1.5.1.TOP1.1_F | chr20:39706132-39706277 | GCCAGCTTGTGATCATGATG |
| TOP1.5.1.TOP1.1_R | chr20:39706132-39706277 | CGCAGCAGCAAAGTCTTAC |
| JAK1.11.1.JAK1.1_F | chr1:65311182-65311337 | GGTGGGACCATTATGGACAT |
| JAK1.11.1.JAK1.1_R | chr1:65311182-65311337 | CAGGTTCCAGACATGGCTAT |
| JAK1.21.1.JAK1.1_F | chr1:65339053-65339210 | CTGGGCCCAAACTTCCTAC |
| JAK1.21.1.JAK1.1_R | chr1:65339053-65339210 | GGCATTCATTTTCCTGCCTT |
| JAK1.7.1.JAK1.1_F | chr1:65306927-65307083 | GCAGCACGGGTGTAACTTA |
| JAK1.7.1.JAK1.1_R | chr1:65306927-65307083 | CTTGGGGAGAAACAGGAGC |
| JAK1.2.5.JAK1.1_F | chr1:65301084-65301242 | GTGGCCCATAGATACCTCAT |
| JAK1.2.5.JAK1.1_R | chr1:65301084-65301242 | TTCACCTTAATCTGTTTGGCA |

| | | |
|---|---|---|
| JAK1.2.6.JAK1.2_F | chr1:65301018-65301160 | CTCTACCCATTGCTGGACAA |
| JAK1.2.6.JAK1.2_R | chr1:65301018-65301160 | AAAATGATAGGCCCAACCCA |
| JAK1.17.1.JAK1.1_F | chr1:65325792-65325946 | AGGTTCTAGGACCAACCAGA |
| JAK1.17.1.JAK1.1_R | chr1:65325792-65325946 | GGGAATTTCTTCTCTCTCAACA |
| JAK1.17.2.JAK1.2_F | chr1:65325733-65325880 | AATGATGACATGCCCCATCA |
| JAK1.17.2.JAK1.2_R | chr1:65325733-65325880 | CTGGTAGATGGCTACTTCCG |
| JAK1.22.1.JAK1.1_F | chr1:65344705-65344851 | TACCAGGTCAGTACTTCCCA |
| JAK1.22.1.JAK1.1_R | chr1:65344705-65344851 | CCGCCTTGATTTTCTGATGT |
| JAK1.1.7.JAK1.1_F | chr1:65300210-65300360 | TTGTTGCAGGAGAAGGACTT |
| JAK1.1.7.JAK1.1_R | chr1:65300210-65300360 | CCCAGATCTAACATTTTTACTGTTG |
| JAK1.18.1.JAK1.1_F | chr1:65330547-65330702 | TGAGTGATTTCAGGGAAGTAAG |
| JAK1.18.1.JAK1.1_R | chr1:65330547-65330702 | GCACTGACCTTTTACCAATAGA |
| JAK1.18.2.JAK1.2_F | chr1:65330426-65330557 | GGAACTCAGCAATTCTCCCT |
| JAK1.18.2.JAK1.2_R | chr1:65330426-65330557 | AACAAGATCCGGGAAGAGTG |
| JAK1.16.1.JAK1.1_F | chr1:65323311-65323465 | GACACCTCATGGCTGTATGG |
| JAK1.16.1.JAK1.1_R | chr1:65323311-65323465 | CCTTTGCCTACTTCTCCCTC |
| JAK1.13.1.JAK1.1_F | chr1:65313209-65313358 | GGAGGAGTTCAGAGGAAGAC |
| JAK1.13.1.JAK1.1_R | chr1:65313209-65313358 | CCTTCTGCTTCTTCCCTCAG |
| JAK1.23.1.JAK1.1_F | chr1:65348959-65349119 | GCAGCTGCCAGTACTCA |
| JAK1.23.1.JAK1.1_R | chr1:65348959-65349119 | CTGCAATGCCATGGCTTTC |
| JAK1.23.2.JAK1.2_F | chr1:65349065-65349212 | GAAGATCACTTCCACCCCTG |
| JAK1.23.2.JAK1.2_R | chr1:65349065-65349212 | AGAACACTCCCTTTTGCCAT |
| JAK1.6.1.JAK1.1_F | chr1:65305285-65305445 | CCCCAGCCTTGCATAACATA |
| JAK1.6.1.JAK1.1_R | chr1:65305285-65305445 | GGTTGAGCTCTGCAGGTAT |
| JAK1.6.2.JAK1.2_F | chr1:65305394-65305536 | GTGGTTACCTCCACTCTCAG |
| JAK1.6.2.JAK1.2_R | chr1:65305394-65305536 | TGAGGAGCAAGACTTCTGTG |
| JAK1.3.1.JAK1.1_F | chr1:65301764-65301920 | TGGGTACTGGATTCAAATGAAG |
| JAK1.3.1.JAK1.1_R | chr1:65301764-65301920 | GCCTCCCAGGAAATGAAGTT |
| JAK1.9.1.JAK1.1_F | chr1:65309746-65309905 | CGGGAAACACCTGCTCA |
| JAK1.9.1.JAK1.1_R | chr1:65309746-65309905 | TCCATCAGTGTGTCTGTTGT |
| JAK1.12.1.JAK1.1_F | chr1:65312274-65312426 | GAAGAGCCTCCACCATCTC |
| JAK1.12.1.JAK1.1_R | chr1:65312274-65312426 | ACATTCCTATGTCCTGCTCG |
| JAK1.14.1.JAK1.1_F | chr1:65316437-65316598 | ATCCCCAAGCTGCTCCAT |
| JAK1.14.1.JAK1.1_R | chr1:65316437-65316598 | CAGACTCCCCCAATCTCTTC |
| JAK1.20.1.JAK1.1_F | chr1:65334994-65335158 | GCCAGTGGGAAGCTTTAC |
| JAK1.20.1.JAK1.1_R | chr1:65334994-65335158 | TCTCCCCCTTGCTCCTA |
| JAK1.15.1.JAK1.1_F | chr1:65321191-65321351 | TGGCACAGGGAGACGAA |
| JAK1.15.1.JAK1.1_R | chr1:65321191-65321351 | TGCCCAGAAGCAGTTCAAG |
| JAK1.15.2.JAK1.2_F | chr1:65321277-65321420 | TTCTTGAGGTGGCTCATGAG |
| JAK1.15.2.JAK1.2_R | chr1:65321277-65321420 | TTACATACCCATGCCTGACC |
| JAK1.8.1.JAK1.1_F | chr1:65307127-65307285 | GTTAAAGCAGCACATGGCAG |
| JAK1.8.1.JAK1.1_R | chr1:65307127-65307285 | ATGTGCCCCTCTTGTTTACA |
| JAK1.4.4.JAK1.1_F | chr1:65303565-65303722 | CCAGAAAGGGCCTATTAAAACA |
| JAK1.4.4.JAK1.1_R | chr1:65303565-65303722 | GCAGCAAGAAATGTCCTTGT |
| JAK1.4.5.JAK1.2_F | chr1:65303654-65303799 | GGTCATCCTTGACGGTGTAA |
| JAK1.4.5.JAK1.2_R | chr1:65303654-65303799 | GCAATGTGCTTGACTTTTACT |
| JAK1.24.1.JAK1.1_F | chr1:65351873-65352007 | CAATAGCCCTACAGCCTCAA |
| JAK1.24.1.JAK1.1_R | chr1:65351873-65352007 | TAGCGCTTCTCTGAAGTAGC |
| JAK1.19.1.JAK1.1_F | chr1:65332549-65332709 | ACTGCCAACAGCCACTTAC |
| JAK1.19.1.JAK1.1_R | chr1:65332549-65332709 | CTTGGCTACCTTGGAAACTT |
| JAK1.19.2.JAK1.2_F | chr1:65332754-65332911 | ACCTTCAGGTCATGCGTG |
| JAK1.19.2.JAK1.2_R | chr1:65332754-65332911 | CCTGTACATGAAAAGGAGATGT |
| JAK1.19.3.JAK1.3_F | chr1:65332622-65332760 | AGTAGAGAACGTTTCCACCG |
| JAK1.19.3.JAK1.3_R | chr1:65332622-65332760 | CAAGACCATTTGTGACAGCA |
| JAK1.10.1.JAK1.1_F | chr1:65310436-65310589 | GGAAGAGAGGGGAGACACA |
| JAK1.10.1.JAK1.1_R | chr1:65310436-65310589 | GAGCAGCTTGGCTAAACTTG |
| JAK1.5.1.JAK1.1_F | chr1:65304125-65304282 | GTGCCTGATGTCTTAATCTGT |
| JAK1.5.1.JAK1.1_R | chr1:65304125-65304282 | CCTCAGACTGATGGATGTTTT |
| ATM.33.15.ATM.1_F | chr11:108170421-108170573 | GATCTCTTACCTATGACTCTACTGA |
| ATM.33.15.ATM.1_R | chr11:108170421-108170573 | GTTATTCAGGTAGGTCAGCATT |
| ATM.33.17.ATM.2_F | chr11:108170534-108170688 | TGCATCTTATACCAAGGCCC |
| ATM.33.17.ATM.2_R | chr11:108170534-108170688 | GGACAAAGCTTTAGTTACTGAGA |
| ATM.32.8.ATM.1_F | chr11:108167981-108168133 | ACAAAAGTGTTGTCTTCATGCT |
| ATM.32.8.ATM.1_R | chr11:108167981-108168133 | CCGCAGTTGACTGAAATGTC |
| ATM.21.1.ATM.1_F | chr11:108143447-108143604 | AACCACAGTTCTTTTCCCGT |
| ATM.21.1.ATM.1_R | chr11:108143447-108143604 | CCATCTGCAGCATTCCAAAT |
| ATM.7.8.ATM.1_F | chr11:108117701-108117856 | TTTGGATTACAGGTGCTTATGA |

| | | |
|---|---|---|
| ATM.7.8.ATM.1_R | chr11:108117701-108117856 | TGTGACATGACCTACTTACTGT |
| ATM.7.9.ATM.2_F | chr11:108117648-108117791 | GGGAGCTAGCAGTGTAAACA |
| ATM.7.9.ATM.2_R | chr11:108117648-108117791 | TGACGGCAATATTACGAAATCC |
| ATM.26.8.ATM.1_F | chr11:108158337-108158493 | GCCCTTGCAGATTGATCACT |
| ATM.26.8.ATM.1_R | chr11:108158337-108158493 | AGGTGTCAACCAATAAACTTCT |
| ATM.26.9.ATM.2_F | chr11:108158278-108158423 | GCCTTTTGAGCTGTCTTGAC |
| ATM.26.9.ATM.2_R | chr11:108158278-108158423 | CCCTGAAAAGTCACAGAGGT |
| ATM.2.24.ATM.1_F | chr11:108098429-108098668 | AGAGCTACAGAACGAAAGGTAGT |
| ATM.2.24.ATM.1_R | chr11:108098429-108098668 | ACACACAAAAGTAATATCACAACAGA |
| ATM.17.1.ATM.1_F | chr11:108139157-108139316 | GGTGCCATTAATCCTTTAGCT |
| ATM.17.1.ATM.1_R | chr11:108139157-108139316 | ATATGCAGGTGGAGGGATT |
| ATM.17.2.ATM.2_F | chr11:108139316-108139457 | CTAGCACGCTAGAACCTACC |
| ATM.17.2.ATM.2_R | chr11:108139316-108139457 | TTCCAACTAGTCTCAGGTTCA |
| ATM.17.2.ATM.3_F | chr11:108139110-108139250 | GCTGTTGTGCCCTTCTCTTA |
| ATM.17.2.ATM.3_R | chr11:108139110-108139250 | CCGAATATCAGCTGCCCTAA |
| ATM.1.1.ATM.1_F | chr11:108098352-108098508 | GTGTGTTCTGAAATTGTGAACC |
| ATM.1.1.ATM.1_R | chr11:108098352-108098508 | CAGGCGCTTAAATTTCTCAAC |
| ATM.18.20.ATM.1_F | chr11:108141767-108141917 | GTAAATGATTTGTGGATAAACCTGA |
| ATM.18.20.ATM.1_R | chr11:108141767-108141917 | GCTTAACAGAACACATCAGTTATT |
| ATM.30.22.ATM.1_F | chr11:108163991-108164144 | ACAGTAAGTTTTGTTGGCTTACT |
| ATM.30.22.ATM.1_R | chr11:108163991-108164144 | TTTGCTGAGTAATACGCAAATC |
| ATM.30.23.ATM.2_F | chr11:108164135-108164258 | TAGATCCTTTTCCTGACCATGT |
| ATM.30.23.ATM.2_R | chr11:108164135-108164258 | TGCTAGAGCATTACAGATTTTTGA |
| ATM.16.3.ATM.1_F | chr11:108137871-108138023 | CTCCTGCAAGAAGCCATCTT |
| ATM.16.3.ATM.1_R | chr11:108137871-108138023 | TCGTTTGCATCACTAACACT |
| ATM.16.4.ATM.2_F | chr11:108137989-108138118 | AATGGAGGTGGAGGATCAGT |
| ATM.16.4.ATM.2_R | chr11:108137989-108138118 | GAGGCCTCTTATACTGCCAA |
| ATM.44.10.ATM.1_F | chr11:108191995-108192130 | TTTCCCTGAAAACCTCTTCTTT |
| ATM.44.10.ATM.1_R | chr11:108191995-108192130 | TACCTTGAGAAAAGCTCCCC |
| ATM.44.11.ATM.2_F | chr11:108192130-108192265 | ATTGGAGAGCTGGAAAGCAT |
| ATM.44.11.ATM.2_R | chr11:108192130-108192265 | CACCCACTTCAGCCTTCTAA |
| ATM.55.1.ATM.1_F | chr11:108206582-108206735 | TATTCTGAAGGGCCGTGATG |
| ATM.55.1.ATM.1_R | chr11:108206582-108206735 | CACTTCACCCAACCAAATGG |
| ATM.55.2.ATM.2_F | chr11:108206496-108206634 | AGATTGGTTTGAGTGCCCTT |
| ATM.55.2.ATM.2_R | chr11:108206496-108206634 | GTTTCCGTGTTTCTCTGCAG |
| ATM.25.17.ATM.1_F | chr11:108155079-108155230 | ACAGACTGCTTTCCAAAGATTC |
| ATM.25.17.ATM.1_R | chr11:108155079-108155230 | GGTTATATCTCATATCATTCAGGGA |
| ATM.25.24.ATM.2_F | chr11:108154868-108155109 | AAAGTATGATACTTTAATGCTGATGGT |
| ATM.25.24.ATM.2_R | chr11:108154868-108155109 | TCCCACTGTCTCTGGTACCC |
| ATM.27.3.ATM.1_F | chr11:108159655-108159812 | TGGAAGTTCACTGGTCTATGA |
| ATM.27.3.ATM.1_R | chr11:108159655-108159812 | ACAGGGCTTTTGGAAAGAAT |
| ATM.27.24.ATM.2_F | chr11:108159723-108159969 | GGGATTTGGATCCTGCTCCT |
| ATM.27.24.ATM.2_R | chr11:108159723-108159969 | TGCTGACAAGTAAAATACCATTTAAAC |
| ATM.28.19.ATM.1_F | chr11:108160302-108160452 | GTAGCCGAGTATCTAATTAAACAAG |
| ATM.28.19.ATM.1_R | chr11:108160302-108160452 | AGCTCCTCCTAAGCCACTTT |
| ATM.28.20.ATM.2_F | chr11:108160444-108160565 | CACCTGTTTGTTAGTTTATTACTGA |
| ATM.28.20.ATM.2_R | chr11:108160444-108160565 | AAAAACAGGAAGAACAGGATAGA |
| ATM.40.20.ATM.1_F | chr11:108186540-108186693 | TCCTTCTTCAATTTTTGTTGTTTCC |
| ATM.40.20.ATM.1_R | chr11:108186540-108186693 | AGCTTTGGGTTTTACACACAC |
| ATM.57.1.ATM.1_F | chr11:108216526-108216665 | GTCTTCATGGATGTTTGCCA |
| ATM.57.1.ATM.1_R | chr11:108216526-108216665 | ACCTGCCAAACAACAAAGTG |
| ATM.57.2.ATM.2_F | chr11:108216380-108216535 | CCTGTTCATCTTTATTGCCCC |
| ATM.57.2.ATM.2_R | chr11:108216380-108216535 | GCAGAAGTAACGGAAAACTGG |
| ATM.61.1.ATM.1_F | chr11:108235814-108235959 | TGTTCTCTCTGTTTAGGTCCT |
| ATM.61.1.ATM.1_R | chr11:108235814-108235959 | ACTGACAACAGGACCTTCTT |
| ATM.61.2.ATM.2_F | chr11:108235694-108235823 | TGTGCATGATGTTTGTTCCC |
| ATM.61.2.ATM.2_R | chr11:108235694-108235823 | ATGGTCCAGTCAAAGAGTGG |
| ATM.20.20.ATM.1_F | chr11:108143213-108143362 | TCAGTGAGTTTTCTGAGTGCT |
| ATM.20.20.ATM.1_R | chr11:108143213-108143362 | GACTGCTTTAAAATTTTCAATGGAG |
| ATM.14.14.ATM.1_F | chr11:108128194-108128319 | AACTGGAATTTGCATTTTTCCT |
| ATM.14.14.ATM.1_R | chr11:108128194-108128319 | AATCTTACCTTGGTACAGTTGC |
| ATM.14.24.ATM.2_F | chr11:108128316-108128444 | TGCAGCTATGTACACGTTGC |
| ATM.14.24.ATM.2_R | chr11:108128316-108128444 | TGAGGCAGGAGAATTGCTGG |
| ATM.59.16.ATM.1_F | chr11:108224472-108224607 | AGCTGTCAAACCTCCTAACT |
| ATM.59.16.ATM.1_R | chr11:108224472-108224607 | CCCTCCTTTACTTCATATCACTTAC |
| ATM.49.1.ATM.1_F | chr11:108200981-108201140 | AGAGCTGGAGTTGGATGAA |
| ATM.49.1.ATM.1_R | chr11:108200981-108201140 | GAGTAACACTTGCCTTCATCA |

| | | |
|---|---|---|
| ATM.49.2.ATM.2_F | chr11:108201106-108201238 | TTCCGACTTTGTTCCCTCTG |
| ATM.49.2.ATM.2_R | chr11:108201106-108201238 | CTGGACCAAGTGCTAGGAAT |
| ATM.49.2.ATM.3_F | chr11:108200847-108200990 | TTTTCCCTGGGATAAAAACCC |
| ATM.49.2.ATM.3_R | chr11:108200847-108200990 | ACGATCCTCTTTCAGTGCAC |
| ATM.24.17.ATM.1_F | chr11:108153504-108153656 | GAAGACTTTATGGCATCTCATTTAG |
| ATM.24.17.ATM.1_R | chr11:108153504-108153656 | AGGGGACTTGCTAAGTATTGT |
| ATM.24.18.ATM.2_F | chr11:108153362-108153508 | TGTTGTTTCTAGGTCCTACTCT |
| ATM.24.18.ATM.2_R | chr11:108153362-108153508 | GATTTAGCCATTCCAAAACCAG |
| ATM.10.3.ATM.1_F | chr11:108122549-108122704 | GCTTGCTTTTCACAATTGTCC |
| ATM.10.3.ATM.1_R | chr11:108122549-108122704 | TTCTAAGTCACCCTCTAACTGA |
| ATM.10.8.ATM.2_F | chr11:108122630-108122762 | CCAGGAACGGTAAAAATGGG |
| ATM.10.8.ATM.2_R | chr11:108122630-108122762 | GCAGCATGCTAATGAACTTAAA |
| ATM.31.3.ATM.1_F | chr11:108165625-108165776 | CACAGGCTTAACCAATACGTG |
| ATM.31.3.ATM.1_R | chr11:108165625-108165776 | AAATTAGCACCCTGAGAAGC |
| ATM.31.4.ATM.2_F | chr11:108165709-108165853 | TGCACTTCCATTGACAAGAC |
| ATM.31.4.ATM.2_R | chr11:108165709-108165853 | GTGACAATGAAACCAAGAGCA |
| ATM.35.3.ATM.1_F | chr11:108173543-108173696 | GGAAAGGTACAATGATTTCCAC |
| ATM.35.3.ATM.1_R | chr11:108173543-108173696 | TGCCTCCACTGTCCAAAAA |
| ATM.35.4.ATM.2_F | chr11:108173696-108173850 | GGATAAAGACACTGACTTGTGC |
| ATM.35.4.ATM.2_R | chr11:108173696-108173850 | TACAACAGTTTGAGTGGGGG |
| ATM.11.24.ATM.1_F | chr11:108123525-108123758 | GGCTTTTGGTCTTCTAAGTGAAGC |
| ATM.11.24.ATM.1_R | chr11:108123525-108123758 | TCCCTGGCTAACAGTTGCAA |
| ATM.53.24.ATM.1_F | chr11:108204588-108204840 | TGCTGTTCCTCAGTTTGTCACT |
| ATM.53.24.ATM.1_R | chr11:108204588-108204840 | AGGGCTTGGGCAAAGGAAAT |
| ATM.48.1.ATM.1_F | chr11:108199768-108199927 | GCAGTAGAAGTTGCTGGAAA |
| ATM.48.1.ATM.1_R | chr11:108199768-108199927 | TGTTCCCTAAGGAGACCTAC |
| ATM.48.2.ATM.2_F | chr11:108199913-108200067 | ACAAGCAAGCTCTCCTGAAA |
| ATM.48.2.ATM.2_R | chr11:108199913-108200067 | TAAGCCGACCTTTAGAGCTC |
| ATM.48.2.ATM.3_F | chr11:108199618-108199773 | GGGTTGGACAAGTTTGCAAT |
| ATM.48.2.ATM.3_R | chr11:108199618-108199773 | AGCTCATCACTACTTTCTCCA |
| ATM.22.13.ATM.1_F | chr11:108150213-108150357 | TGTTTGTTTGCTTGCTTGTT |
| ATM.22.13.ATM.1_R | chr11:108150213-108150357 | AGCAAGCATATGATAACAGCA |
| ATM.42.1.ATM.1_F | chr11:108188117-108188276 | TAGGCCTTGCAGAATTTGGG |
| ATM.42.1.ATM.1_R | chr11:108188117-108188276 | AGAATGAGGAGAGAGGCAAA |
| ATM.42.2.ATM.2_F | chr11:108187972-108188116 | CACCACACCCAGCTGATATT |
| ATM.42.2.ATM.2_R | chr11:108187972-108188116 | ACGGAAAGAATATGGCAGAGT |
| ATM.46.1.ATM.1_F | chr11:108196843-108196968 | AATTCAGTTAGCTGTGGAGTCT |
| ATM.46.1.ATM.1_R | chr11:108196843-108196968 | ACAGTAAAACACTAATCCAGCC |
| ATM.46.2.ATM.2_F | chr11:108196709-108196860 | TCAATGAATGGTAGTTGCTGC |
| ATM.46.2.ATM.2_R | chr11:108196709-108196860 | TGCCCAGAATACTTGTGCTT |
| ATM.13.24.ATM.1_F | chr11:108126842-108127089 | CACCTTTAACTCAGTTAACTGAACT |
| ATM.13.24.ATM.1_R | chr11:108126842-108127089 | ACATTTCATTCAAATTTATCCGAAACT |
| ATM.23.20.ATM.1_F | chr11:108151762-108151916 | CCCTGAAACTTTGGATGAAATTT |
| ATM.23.20.ATM.1_R | chr11:108151762-108151916 | GTGACCTAAGGAAGCTTCTAAT |
| ATM.23.21.ATM.2_F | chr11:108151660-108151812 | TCTGGAGTTCAGTTGGGATT |
| ATM.23.21.ATM.2_R | chr11:108151660-108151812 | CTGTTTTTCGCAGATAGGGC |
| ATM.29.6.ATM.1_F | chr11:108163324-108163481 | CAGAACTTACTGGTTGTTGTTG |
| ATM.29.6.ATM.1_R | chr11:108163324-108163481 | TGCTCATACACAAGGGGTAT |
| ATM.29.8.ATM.2_F | chr11:108163397-108163527 | AGCTTCTCCCTTTGTTGTGA |
| ATM.29.8.ATM.2_R | chr11:108163397-108163527 | ACCATTTTGAAGATGAGTCAGA |
| ATM.6.3.ATM.1_F | chr11:108115494-108115647 | CCCCCTGTTATACCCAGTTG |
| ATM.6.3.ATM.1_R | chr11:108115494-108115647 | CATTAAGCCTATGTTGAGTCCA |
| ATM.6.17.ATM.2_F | chr11:108115641-108115784 | TGAAATTCTTCCCACTTTGCT |
| ATM.6.17.ATM.2_R | chr11:108115641-108115784 | GACTTCTATGTTTGAATGAAGAAGC |
| ATM.50.24.ATM.1_F | chr11:108202085-108202287 | ACCACTTGTGCTAATAGAGGAGC |
| ATM.50.24.ATM.1_R | chr11:108202085-108202287 | TGTGGTTTGATTTTCAGGTTTACT |
| ATM.39.1.ATM.1_F | chr11:108183137-108183291 | GAGGTGTTCTTGTGACAAACA |
| ATM.39.1.ATM.1_R | chr11:108183137-108183291 | ACATGGCATCTGTACAGTGT |
| ATM.3.10.ATM.1_F | chr11:108099888-108100039 | TCAACGAGTTTCTGAAATTGC |
| ATM.3.10.ATM.1_R | chr11:108099888-108100039 | ACATCACTTACTTCTGTTTGCA |
| ATM.3.12.ATM.2_F | chr11:108099960-108100087 | ATGTCTGAGAATAGCAAAACCA |
| ATM.3.12.ATM.2_R | chr11:108099960-108100087 | AACTCACGCGACAGTAATCT |
| ATM.36.3.ATM.1_F | chr11:108175388-108175540 | CTCAAACTATTGGGTGGATTTG |
| ATM.36.3.ATM.1_R | chr11:108175388-108175540 | GTTGTGGATCGGCTCGTTT |
| ATM.36.4.ATM.2_F | chr11:108175509-108175643 | GCTTTCTACACATGTTCAGGG |
| ATM.36.4.ATM.2_R | chr11:108175509-108175643 | AGTGCTTTTAGTGGGATTCCA |
| ATM.51.17.ATM.1_F | chr11:108202636-108202777 | AGAATTTCAATGGATCACCCC |

| | | |
|---|---|---|
| ATM.51.17.ATM.1_R | chr11:108202636-108202777 | CACACTTCTAAAAGGTACGTATGT |
| ATM.51.18.ATM.2_F | chr11:108202510-108202656 | GCTCTACCCACTGCAGTATC |
| ATM.51.18.ATM.2_R | chr11:108202510-108202656 | TTTGCATTTGCTAAGGCCAG |
| ATM.9.1.ATM.1_F | chr11:108121538-108121698 | ACCCCAACAGCGACATG |
| ATM.9.1.ATM.1_R | chr11:108121538-108121698 | CTCCAAGTAAGCCAAAGTTTTC |
| ATM.9.2.ATM.2_F | chr11:108121689-108121844 | TCGTGGTATAAGTTCTGAGCA |
| ATM.9.2.ATM.2_R | chr11:108121689-108121844 | ATGTGAGTGTGTGTGTGTCT |
| ATM.9.20.ATM.3_F | chr11:108121393-108121537 | CCTTTTAGTTTGTTAATGTGATGGA |
| ATM.9.20.ATM.3_R | chr11:108121393-108121537 | CACATATGGTGTACGTTCCC |
| ATM.58.17.ATM.1_F | chr11:108217990-108218144 | GGAATCAGTGATTTCAGATTGTTTG |
| ATM.58.17.ATM.1_R | chr11:108217990-108218144 | GGTAGGCAAACAACATTCCA |
| ATM.12.1.ATM.1_F | chr11:108124551-108124710 | ACTTTCTTGAAGTGAACACCAC |
| ATM.12.1.ATM.1_R | chr11:108124551-108124710 | CCAGAAGACAGCGATCCA |
| ATM.12.2.ATM.2_F | chr11:108124683-108124825 | ACCAGTCCAGTATTGGCTTC |
| ATM.12.2.ATM.2_R | chr11:108124683-108124825 | CTGCCCCTATTTCTCCTTCC |
| ATM.12.2.ATM.3_F | chr11:108124510-108124662 | GGCAAAGCATTAGGTACTTGG |
| ATM.12.2.ATM.3_R | chr11:108124510-108124662 | GAAGCCAATACTGGACTGGT |
| ATM.41.1.ATM.1_F | chr11:108186715-108186850 | GTGTGTGTAAAACCCAAAGCT |
| ATM.41.1.ATM.1_R | chr11:108186715-108186850 | TGGCTTTACCAAATCTGGGA |
| ATM.15.1.ATM.1_F | chr11:108129657-108129809 | GATAGAGAAAACACTGTCTGCC |
| ATM.15.1.ATM.1_R | chr11:108129657-108129809 | TGATAGCAAAACAGGAAGCA |
| ATM.47.1.ATM.1_F | chr11:108198334-108198487 | CAGTTGGGTACAGTCATGGT |
| ATM.47.1.ATM.1_R | chr11:108198334-108198487 | AGGGTTGCTCCAAAAATCTT |
| ATM.8.6.ATM.1_F | chr11:108119621-108119760 | GCGAAACTCTGGCTCAAAAA |
| ATM.8.6.ATM.1_R | chr11:108119621-108119760 | ATTACTTCCCAGCCTAGTTCT |
| ATM.8.7.ATM.2_F | chr11:108119756-108119900 | GTGTCCCTTGCAAAAGGAAG |
| ATM.8.7.ATM.2_R | chr11:108119756-108119900 | TGGTTGAGATGAAAGGATTCCA |
| ATM.38.1.ATM.1_F | chr11:108180930-108181081 | ATGATGCTTTCTGGCTGGAT |
| ATM.38.1.ATM.1_R | chr11:108180930-108181081 | ACCCTTATTGAGACAATGCCA |
| ATM.38.2.ATM.2_F | chr11:108180807-108180940 | TCTGTTAAGCAGTCACTACCA |
| ATM.38.2.ATM.2_R | chr11:108180807-108180940 | GAGCTACCTTGGCAACTTCT |
| ATM.52.1.ATM.1_F | chr11:108203505-108203658 | ATAGGATCGAACAGAGGCTG |
| ATM.52.1.ATM.1_R | chr11:108203505-108203658 | ACAGAGAGTAACACAGCAAGA |
| ATM.52.8.ATM.2_F | chr11:108203398-108203548 | TCCATTGTCTAGATTTGTGCA |
| ATM.52.8.ATM.2_R | chr11:108203398-108203548 | AGTGCCTCAACACTTCTGAC |
| ATM.4.8.ATM.1_F | chr11:108106409-108106565 | TGAAATAGGAGCACCTAGGC |
| ATM.4.8.ATM.1_R | chr11:108106409-108106565 | CACAAACAACAACCTTCAAAAC |
| ATM.4.9.ATM.2_F | chr11:108106348-108106476 | AGTTGCCATTCCAAGTGTCT |
| ATM.4.9.ATM.2_R | chr11:108106348-108106476 | GTATGTTGCTACAATCAGCTCC |
| ATM.60.5.ATM.1_F | chr11:108225503-108225661 | CGTTGACAACATTGGTGTGT |
| ATM.60.5.ATM.1_R | chr11:108225503-108225661 | TCACATCAGTGACTTCCTGA |
| ATM.34.1.ATM.1_F | chr11:108172402-108172561 | GTTCGATCAGCAGCTGTTAC |
| ATM.34.1.ATM.1_R | chr11:108172402-108172561 | CCCACAGCAAACAGAACTGT |
| ATM.34.2.ATM.2_F | chr11:108172327-108172467 | CAGTGGAGGTTAACATTCATCA |
| ATM.34.2.ATM.2_R | chr11:108172327-108172467 | GATAGGCCAGCATTGGATCT |
| ATM.56.1.ATM.1_F | chr11:108213957-108214100 | CTGTAACTCCAGGTGGTTCC |
| ATM.56.1.ATM.1_R | chr11:108213957-108214100 | CCTTTAATTTTGGGTGTCACTC |
| ATM.56.2.ATM.2_F | chr11:108213822-108213975 | CCTCCAAGGAGCTTTGTCTT |
| ATM.56.2.ATM.2_R | chr11:108213822-108213975 | CCTGTGCACCATTCAAGAAC |
| ATM.37.1.ATM.1_F | chr11:108178584-108178720 | AGGAAGAAGGTGTGTAAGCA |
| ATM.37.1.ATM.1_R | chr11:108178584-108178720 | AACGTAAGAAGCAACACTCA |
| ATM.45.1.ATM.1_F | chr11:108196047-108196206 | TGGCATTCAGATCAGTCACA |
| ATM.45.1.ATM.1_R | chr11:108196047-108196206 | GTGTTTGGTGAGAATGTCCT |
| ATM.45.2.ATM.2_F | chr11:108196177-108196328 | TGGAGATCCTGATGGAAAAGG |
| ATM.45.2.ATM.2_R | chr11:108196177-108196328 | TCCCTCAGGCTTTCTGTTTT |
| ATM.45.2.ATM.3_F | chr11:108196033-108196162 | TGAACTCTATGTCGTGGCAT |
| ATM.45.2.ATM.3_R | chr11:108196033-108196162 | GTCCATTTCCTTTTCCATCAGG |
| ATM.43.24.ATM.1_F | chr11:108190616-108190825 | AAATTTTGTCCTTTGGTGAAGCT |
| ATM.43.24.ATM.1_R | chr11:108190616-108190825 | AGTTTTCAGAAAAGAAGCCATGACA |
| ATM.19.1.ATM.1_F | chr11:108141988-108142136 | TTTACCACAGCAATGTGTGT |
| ATM.19.1.ATM.1_R | chr11:108141988-108142136 | GGACCACAAAATAGACTGTACC |
| ATM.19.2.ATM.2_F | chr11:108141841-108141994 | GAAGAGTACCCCTTGCCAAT |
| ATM.19.2.ATM.2_R | chr11:108141841-108141994 | AACATCTTGGTCACGACGAT |
| ATM.62.1.ATM.1_F | chr11:108236091-108236246 | CAACAAAGTAGCTGAACGTGT |
| ATM.62.1.ATM.1_R | chr11:108236091-108236246 | GGCTGAATGAAAGGGTAATTCA |
| ATM.62.2.ATM.2_F | chr11:108235955-108236113 | CGAAATCTCAGGTGAGCAGT |
| ATM.62.2.ATM.2_R | chr11:108235955-108236113 | GCCTTCTTCCACTCCTTTCA |

| | | |
|---|---|---|
| ATM.5.1.ATM.1_F | chr11:108114731-108114887 | CTGAAACCTTCACAAGATGTTC |
| ATM.5.1.ATM.1_R | chr11:108114731-108114887 | ACATGGTCTTGCAAGATCAA |
| ATM.5.17.ATM.2_F | chr11:108114601-108114741 | CCTTTTTCTGTATGGGATTATGGA |
| ATM.5.17.ATM.2_R | chr11:108114601-108114741 | ACAGCATGAATTATTCTAGCCAC |
| ATM.54.1.ATM.1_F | chr11:108205716-108205860 | GTGGACCACACAGGAGAATA |
| ATM.54.1.ATM.1_R | chr11:108205716-108205860 | TCATCACTAAAACTCTAAGGGC |
| ATM.54.2.ATM.2_F | chr11:108205597-108205750 | CCACTATCACATCGTCATTTGT |
| ATM.54.2.ATM.2_R | chr11:108205597-108205750 | ACCTCCTGCTAAGCGAAATT |
| ERBB3.11.1.ERBB3.1_F | chr12:56486713-56486867 | AAGTTATAGGGGAGGAGCCA |
| ERBB3.11.1.ERBB3.1_R | chr12:56486713-56486867 | GCCATTTCCTCCATACCCTT |
| ERBB3.21.1.ERBB3.1_F | chr12:56491599-56491756 | CTACCTTGAGGAACATGGTATG |
| ERBB3.21.1.ERBB3.1_R | chr12:56491599-56491756 | TATCCCCATGCTTCACTCCA |
| ERBB3.21.2.ERBB3.2_F | chr12:56491493-56491648 | CACTGCTGAGAGGTACCTTC |
| ERBB3.21.2.ERBB3.2_R | chr12:56491493-56491648 | CAAAATCTGCCACCTGAACC |
| ERBB3.7.1.ERBB3.1_F | chr12:56481795-56481947 | ACATAAATCTGATGAGCCTCCT |
| ERBB3.7.1.ERBB3.1_R | chr12:56481795-56481947 | TCCTTCCCCTCAGACACTTA |
| ERBB3.26.1.ERBB3.1_F | chr12:56493901-56494046 | TTAACTACTCAAAGGCCCCC |
| ERBB3.26.1.ERBB3.1_R | chr12:56493901-56494046 | TGATCCTAAAACCCAGCCTC |
| ERBB3.2.1.ERBB3.1_F | chr12:56477544-56477701 | GCTTTGAACAGTGTGTCCTG |
| ERBB3.2.1.ERBB3.1_R | chr12:56477544-56477701 | GAGCAGGTTGAGGAAGGAG |
| ERBB3.2.2.ERBB3.2_F | chr12:56477531-56477671 | CACTCTTCCCTCTGCTTTGA |
| ERBB3.2.2.ERBB3.2_R | chr12:56477531-56477671 | CTAACCTGCAGGAAGGAGAG |
| ERBB3.17.1.ERBB3.1_F | chr12:56489468-56489626 | AGCAAAACCCATCTGACAATG |
| ERBB3.17.1.ERBB3.1_R | chr12:56489468-56489626 | ACTTCCAGGACATGCAAAAA |
| ERBB3.17.2.ERBB3.2_F | chr12:56489375-56489518 | CCAGGTCAGCATCATACCTT |
| ERBB3.17.2.ERBB3.2_R | chr12:56489375-56489518 | CCACGCCAGTAGAGAAAAGT |
| ERBB3.22.1.ERBB3.1_F | chr12:56492238-56492372 | TGGGACTTGGAAATCCTAAGA |
| ERBB3.22.1.ERBB3.1_R | chr12:56492238-56492372 | GTGATGGTAGAGAGGGCATC |
| ERBB3.1.1.ERBB3.1_F | chr12:56474031-56474167 | CTCTTGCCTCGATGTCCTAG |
| ERBB3.1.1.ERBB3.1_R | chr12:56474031-56474167 | TGCTCTCGCGCCACTTA |
| ERBB3.18.1.ERBB3.1_F | chr12:56490275-56490422 | TCCTGAGTAACTCCTTCCCA |
| ERBB3.18.1.ERBB3.1_R | chr12:56490275-56490422 | TTCCCCACCTCTCCAGAATT |
| ERBB3.16.1.ERBB3.1_F | chr12:56488960-56489101 | ATGCCACGGTAAGTTCTGAA |
| ERBB3.16.1.ERBB3.1_R | chr12:56488960-56489101 | TTTCCAGAATCTCCAACCCC |
| ERBB3.13.1.ERBB3.1_F | chr12:56487556-56487719 | TTCCTTTCCAGTGGCAGAG |
| ERBB3.13.1.ERBB3.1_R | chr12:56487556-56487719 | AGTTCCATTGCAGGGCC |
| ERBB3.13.2.ERBB3.2_F | chr12:56487653-56487783 | TTATAGCCGAGGAGGTGTCT |
| ERBB3.13.2.ERBB3.2_R | chr12:56487653-56487783 | CTACCTCAATCCCACCAAGG |
| ERBB3.13.2.ERBB3.3_F | chr12:56487482-56487632 | TGGAAGCAGTAACGAGGAAG |
| ERBB3.13.2.ERBB3.3_R | chr12:56487482-56487632 | AGACACCTCCTCGGCTATAA |
| ERBB3.23.1.ERBB3.1_F | chr12:56492531-56492693 | GCCAGTGACTAGTCCATGTC |
| ERBB3.23.1.ERBB3.1_R | chr12:56492531-56492693 | TGGGCTCAGCAGGTAAC |
| ERBB3.23.2.ERBB3.2_F | chr12:56492393-56492536 | GATGCCCTCTCTACCATCAC |
| ERBB3.23.2.ERBB3.2_R | chr12:56492393-56492536 | CCAAACTGTCACACCTGTTG |
| ERBB3.27.1.ERBB3.1_F | chr12:56494865-56495024 | GAGTCTGCAGTTTCTGGGAG |
| ERBB3.27.1.ERBB3.1_R | chr12:56494865-56495024 | GAATGGTAGGCGCTATCTCC |
| ERBB3.27.2.ERBB3.2_F | chr12:56494768-56494927 | AGGCAGTGAACAACCCAATA |
| ERBB3.27.2.ERBB3.2_R | chr12:56494768-56494927 | TTACATGCCCCTCTGATGAC |
| ERBB3.27.2.ERBB3.3_F | chr12:56495045-56495178 | GGAGATAGCGCCTACCATTC |
| ERBB3.27.2.ERBB3.3_R | chr12:56495045-56495178 | CGGGGAAAGAATTCGAGGAA |
| ERBB3.27.3.ERBB3.4_F | chr12:56494950-56495108 | CATCAGAGGGGCATGTAACA |
| ERBB3.27.3.ERBB3.4_R | chr12:56494950-56495108 | GGCATGACATAACCGTTGAC |
| ERBB3.25.1.ERBB3.1_F | chr12:56493642-56493804 | AGAGAGTGGGCCTGGAAT |
| ERBB3.25.1.ERBB3.1_R | chr12:56493642-56493804 | TGTCTTACCCCACGTGG |
| ERBB3.25.2.ERBB3.2_F | chr12:56493740-56493880 | CCTAGACTTGGAAGCAGAGG |
| ERBB3.25.2.ERBB3.2_R | chr12:56493740-56493880 | GGGGGCCTTTGAGTAGTTAA |
| ERBB3.25.2.ERBB3.3_F | chr12:56493554-56493691 | GTAGGGAGTAGGAGGTGCTA |
| ERBB3.25.2.ERBB3.3_R | chr12:56493554-56493691 | TAGTTCTGGCTCCAGCTCTA |
| ERBB3.6.1.ERBB3.1_F | chr12:56481576-56481716 | CCATGCTTTCTCTCCTTCCA |
| ERBB3.6.1.ERBB3.1_R | chr12:56481576-56481716 | TCCCAATTTCAGAACCCAGG |
| ERBB3.28.1.ERBB3.1_F | chr12:56495351-56495514 | AGGCACCCTTTCTTCAGTG |
| ERBB3.28.1.ERBB3.1_R | chr12:56495351-56495514 | AACTCTGTGTGCTGCCC |
| ERBB3.28.2.ERBB3.2_F | chr12:56495199-56495355 | TTCCTCGAATTCTTTCCCCG |
| ERBB3.28.2.ERBB3.2_R | chr12:56495199-56495355 | CAGTACCCAGGACAGAACTG |
| ERBB3.28.2.ERBB3.3_F | chr12:56495555-56495715 | ACTCCACCCTGTACCCATC |
| ERBB3.28.2.ERBB3.3_R | chr12:56495555-56495715 | GGCATAATGGACATGGGGG |
| ERBB3.28.3.ERBB3.4_F | chr12:56495702-56495858 | AGATGAGAGCTTTTCAGGGG |

| | | |
|---|---|---|
| ERBB3.28.3.ERBB3.4_R | chr12:56495702-56495858 | TGCCATTAAATGCTCCCTGA |
| ERBB3.28.3.ERBB3.5_F | chr12:56495499-56495657 | GTACATGGATGTGGGGTCAG |
| ERBB3.28.3.ERBB3.5_R | chr12:56495499-56495657 | ATACCCTTGCTCAGATGCTG |
| ERBB3.3.1.ERBB3.1_F | chr12:56478789-56478949 | TCTGTCACAGTGGATTCGAG |
| ERBB3.3.1.ERBB3.1_R | chr12:56478789-56478949 | ACCGGTGAGCTGAGTCAA |
| ERBB3.3.2.ERBB3.2_F | chr12:56478937-56479096 | AACTCCAGCCACGCTCT |
| ERBB3.3.2.ERBB3.2_R | chr12:56478937-56479096 | CTTAGGGAGCAATGGACCT |
| ERBB3.3.2.ERBB3.3_F | chr12:56478725-56478874 | CCTGAAGGAGGAGAGGAGAT |
| ERBB3.3.2.ERBB3.3_R | chr12:56478725-56478874 | ATGGCAAACTTCCCATCGTA |
| ERBB3.9.1.ERBB3.1_F | chr12:56482532-56482691 | TGAACCTCTCTTACATTTGCAG |
| ERBB3.9.1.ERBB3.1_R | chr12:56482532-56482691 | ATGAACTGTGAGGGGTGC |
| ERBB3.9.2.ERBB3.2_F | chr12:56482674-56482824 | TTAGAGATCCTGCCTTCCCT |
| ERBB3.9.2.ERBB3.2_R | chr12:56482674-56482824 | CAAAGATGTTCCTGAGGGGA |
| ERBB3.12.1.ERBB3.1_F | chr12:56487149-56487310 | CGGGGCTTCTCATTGTTGA |
| ERBB3.12.1.ERBB3.1_R | chr12:56487149-56487310 | TCTGCGCGGCCGATTAT |
| ERBB3.12.2.ERBB3.2_F | chr12:56487260-56487396 | CTGCTACCACCACTCTTTGA |
| ERBB3.12.2.ERBB3.2_R | chr12:56487260-56487396 | CCACGTCTAGGGCAGAATAG |
| ERBB3.12.2.ERBB3.3_F | chr12:56487034-56487163 | TTCCCTCTCAGTGGATCTGA |
| ERBB3.12.2.ERBB3.3_R | chr12:56487034-56487163 | GAAGCCCAGAGATGTGACAT |
| ERBB3.14.1.ERBB3.1_F | chr12:56487846-56487981 | GAGCATGAAGGTCAGGACTT |
| ERBB3.14.1.ERBB3.1_R | chr12:56487846-56487981 | CTTGGAGATCCTGGTGCTAC |
| ERBB3.15.1.ERBB3.1_F | chr12:56488196-56488354 | TTCCACTCAGGGCTCTGATA |
| ERBB3.15.1.ERBB3.1_R | chr12:56488196-56488354 | CCTGACCCCCTCTCCTTATT |
| ERBB3.15.2.ERBB3.2_F | chr12:56488140-56488296 | CTTTGCTGGGAGGTATGGAA |
| ERBB3.15.2.ERBB3.2_R | chr12:56488140-56488296 | GGCCGACATTCATTCTGAAC |
| ERBB3.20.1.ERBB3.1_F | chr12:56490846-56491009 | GCATATGCTGGCCATTGG |
| ERBB3.20.1.ERBB3.1_R | chr12:56490846-56491009 | AGGCTTCTCTCACCTTGG |
| ERBB3.20.2.ERBB3.2_F | chr12:56490873-56491020 | GACCATGCCCACATTGTAAG |
| ERBB3.20.2.ERBB3.2_R | chr12:56490873-56491020 | ACAGAATTCCTCCAGGCTTC |
| ERBB3.20.2.ERBB3.3_F | chr12:56490786-56490930 | GTGCACATGCTGAGTGTATG |
| ERBB3.20.2.ERBB3.3_R | chr12:56490786-56490930 | ACATGATCCAGCAGAGAACC |
| ERBB3.8.1.ERBB3.1_F | chr12:56482327-56482469 | GTCCTCCTCCTCTTCCCTAG |
| ERBB3.8.1.ERBB3.1_R | chr12:56482327-56482469 | CTCAGAGGAAAGGCTGTCTC |
| ERBB3.4.1.ERBB3.1_F | chr12:56480307-56480452 | AACCCTGTCACTTCTTTCCC |
| ERBB3.4.1.ERBB3.1_R | chr12:56480307-56480452 | ACTGGGGAGCAATCTTGATC |
| ERBB3.24.1.ERBB3.1_F | chr12:56493397-56493533 | CCGGCCATGGAATGTATTCT |
| ERBB3.24.1.ERBB3.1_R | chr12:56493397-56493533 | TAGCACCTCCTACTCCCTAC |
| ERBB3.19.1.ERBB3.1_F | chr12:56490503-56490638 | TGACCTAGGGAGAATGACCT |
| ERBB3.19.1.ERBB3.1_R | chr12:56490503-56490638 | AGCGGCATACAGAATTTCCT |
| ERBB3.10.1.ERBB3.1_F | chr12:56486490-56486634 | GTGCTCATTGCCATTGAGTT |
| ERBB3.10.1.ERBB3.1_R | chr12:56486490-56486634 | GCCAGTGGTTCACCTATTCT |
| ERBB3.5.1.ERBB3.1_F | chr12:56481331-56481466 | AGCCTTTCTTAGCCCTGATG |
| ERBB3.5.1.ERBB3.1_R | chr12:56481331-56481466 | TGGGGAGGAAGGGATATGAA |
| RET.11.1.RET.1_F | chr10:43609988-43610151 | GCTGTCCTCTTCTCCTTCA |
| RET.11.1.RET.1_R | chr10:43609988-43610151 | TCCACGGAGACCTGGTT |
| RET.11.2.RET.2_F | chr10:43610151-43610311 | TCGCTGGACTCCATGGA |
| RET.11.2.RET.2_R | chr10:43610151-43610311 | CTATGGAAATGGGGGCAGAA |
| RET.11.2.RET.3_F | chr10:43609864-43610017 | CCATGAGGCAGAGCATACG |
| RET.11.2.RET.3_R | chr10:43609864-43610017 | AAACTTGTGGTAGCAGTGGA |
| RET.7.1.RET.1_F | chr10:43606674-43606835 | GATCGGGAAAGTCTGTGTGG |
| RET.7.1.RET.1_R | chr10:43606674-43606835 | TGGCCACCACCATGTAGT |
| RET.7.2.RET.2_F | chr10:43606809-43606970 | TGAATGACACCAAGGCCC |
| RET.7.2.RET.2_R | chr10:43606809-43606970 | TTTTCTCAAAGGGCAGGAGG |
| RET.7.2.RET.3_F | chr10:43606579-43606708 | CTGTTCCAGGACTTAGGCTG |
| RET.7.2.RET.3_R | chr10:43606579-43606708 | CCAGAGGAATGCAGCTTGTA |
| RET.17.1.RET.1_F | chr10:43619119-43619278 | GGTCCTTTCACTCTCTGCAG |
| RET.17.1.RET.1_R | chr10:43619119-43619278 | TTCCCAAGTGAGGCTGG |
| RET.2.1.RET.1_F | chr10:43595907-43596066 | CACTTCCCTACTTCCCACAG |
| RET.2.1.RET.1_R | chr10:43595907-43596066 | TTGTTCTCATGCAGCCGT |
| RET.2.2.RET.2_F | chr10:43596057-43596218 | CCAGCATCTCTACGGCA |
| RET.2.2.RET.2_R | chr10:43596057-43596218 | GTGATAAGGGCGGCTTGAG |
| RET.1.5.RET.1_F | chr10:43572688-43572850 | AGTCCCTCCAGCCGTG |
| RET.1.5.RET.1_R | chr10:43572688-43572850 | AACAGAAAGGCGCTTCTGAA |
| RET.18.1.RET.1_F | chr10:43620313-43620443 | TGCTTGGATCATATTGGCCT |
| RET.18.1.RET.1_R | chr10:43620313-43620443 | TTCAGCTTGTGGGAATTGGA |
| RET.13.1.RET.1_F | chr10:43613821-43613979 | GCTCTGTGCTGCATTTCAG |
| RET.13.1.RET.1_R | chr10:43613821-43613979 | GAGAACAGGGCTGTATGGAG |

| | | |
|---|---|---|
| RET.16.1.RET.1_F | chr10:43617356-43617488 | CCCCTCCTTCCTAGAGAGTT |
| RET.16.1.RET.1_R | chr10:43617356-43617488 | TGTTTCTGTAACCTCCACCC |
| RET.6.1.RET.1_F | chr10:43604508-43604668 | CAACCGGAACCTCTCCATC |
| RET.6.1.RET.1_R | chr10:43604508-43604668 | GGCTCACCTGGGCAAAT |
| RET.6.2.RET.2_F | chr10:43604557-43604710 | GCTGGTCAATGACTCAGACT |
| RET.6.2.RET.2_R | chr10:43604557-43604710 | ATGTCCCTTGGCCTTTCAAT |
| RET.6.2.RET.3_F | chr10:43604438-43604576 | TGCTACACATGAGGAAGCAG |
| RET.6.2.RET.3_R | chr10:43604438-43604576 | TTGAAGTGGAGCAAGAGGAC |
| RET.3.1.RET.1_F | chr10:43597790-43597950 | GACCTGACTTCTCTCTGCAG |
| RET.3.1.RET.1_R | chr10:43597790-43597950 | GGCCTTGTCTCTGGGAA |
| RET.3.2.RET.2_F | chr10:43597920-43598078 | TTCAACACCTCCTTTCCAGC |
| RET.3.2.RET.2_R | chr10:43597920-43598078 | CACAAGGTCGGCACTCA |
| RET.9.1.RET.1_F | chr10:43608286-43608423 | ATGGGTGACAGCCTGCT |
| RET.9.1.RET.1_R | chr10:43608286-43608423 | CTGTTCCCATGCCCTGATTA |
| RET.12.3.RET.1_F | chr10:43612029-43612169 | CCCTTCCCTCATTTCCAACA |
| RET.12.3.RET.1_R | chr10:43612029-43612169 | GGCAGGTACCTTTCAGCAT |
| RET.12.4.RET.2_F | chr10:43612121-43612272 | GGAAAAGTGGTCAAGGCAAC |
| RET.12.4.RET.2_R | chr10:43612121-43612272 | CTCTAGAACAGCATTGGGGG |
| RET.14.1.RET.1_F | chr10:43615016-43615179 | TGGAGTACGCCAAATACGG |
| RET.14.1.RET.1_R | chr10:43615016-43615179 | CACCTTCATCTCGGCCA |
| RET.14.2.RET.2_F | chr10:43615160-43615296 | ATCTCATTTGCCTGGCAGAT |
| RET.14.2.RET.2_R | chr10:43615160-43615296 | CATGGTGGGCTAGAGTGTG |
| RET.14.2.RET.3_F | chr10:43614884-43615046 | CCCCTTACTCATTGGGTGG |
| RET.14.2.RET.3_R | chr10:43614884-43615046 | TAGCCAGGCCCCACTTT |
| RET.20.1.RET.1_F | chr10:43623574-43623732 | TTTTAGGCATGTCAGACCCG |
| RET.20.1.RET.1_R | chr10:43623574-43623732 | CCCCTTGTGAGTCCATTACC |
| RET.20.2.RET.2_F | chr10:43623465-43623605 | CACAGAAACCACGAGTTTGG |
| RET.20.2.RET.2_R | chr10:43623465-43623605 | TGTTAGTGCCATCAGCTCTC |
| RET.15.1.RET.1_F | chr10:43615529-43615687 | TCGTGCTATTTTTCCTCACAG |
| RET.15.1.RET.1_R | chr10:43615529-43615687 | CATGGTGCACCTGGGAT |
| RET.8.1.RET.1_F | chr10:43607537-43607673 | CTGTGACCCTGCTTGTCTG |
| RET.8.1.RET.1_R | chr10:43607537-43607673 | GGCGTTTCCAGGGCTTA |
| RET.4.1.RET.1_F | chr10:43600496-43600656 | GAGAAGTACGAGCTGGTGG |
| RET.4.1.RET.1_R | chr10:43600496-43600656 | ACTGGGTAGACCACAGCA |
| RET.4.2.RET.2_F | chr10:43600399-43600558 | TCTGCTTGGTGCGCA |
| RET.4.2.RET.2_R | chr10:43600399-43600558 | AGTCGTCCTCGTCGTACA |
| RET.10.1.RET.1_F | chr10:43609019-43609167 | AGGGGGCAGCATTGTTG |
| RET.10.1.RET.1_R | chr10:43609019-43609167 | GATGTGCTGTTGAGACCTCT |
| RET.10.2.RET.2_F | chr10:43608947-43609081 | GGACACTGCCCTGGAAATAT |
| RET.10.2.RET.2_R | chr10:43608947-43609081 | GAAGCACTTCTCCTCCTCAG |
| RET.19.1.RET.1_F | chr10:43622033-43622192 | TGTCTTCCAGGACTACTTGG |
| RET.19.1.RET.1_R | chr10:43622033-43622192 | GACAGCGGTGCTAGAATCTA |
| RET.19.2.RET.2_F | chr10:43622067-43622225 | ACTCCATCTGACTCCCTGAT |
| RET.19.2.RET.2_R | chr10:43622067-43622225 | AAGCATCACAGAGAGGAAGG |
| RET.19.2.RET.3_F | chr10:43621962-43622097 | CCTGAGGATGGCTTGTTGTA |
| RET.19.2.RET.3_R | chr10:43621962-43622097 | TTATTACAGTCCACCAGCGG |
| RET.5.1.RET.1_F | chr10:43601891-43602053 | ATCAGGGGAGCTGGTGA |
| RET.5.1.RET.1_R | chr10:43601891-43602053 | GAGCACCTCATTTCCTGGG |
| RET.5.2.RET.2_F | chr10:43601729-43601892 | CATCTGGTCCACCTATGGG |
| RET.5.2.RET.2_R | chr10:43601729-43601892 | AGCGTGCTTGTGTACCG |
| FGFR2.11.1.FGFR2.1_F | chr10:123263324-123263481 | ACTTATCTCTTGGAAACTCCCA |
| FGFR2.11.1.FGFR2.1_R | chr10:123263324-123263481 | CAGATGGAGCCAGGATTACA |
| FGFR2.11.2.FGFR2.2_F | chr10:123263299-123263446 | GGACATGGCCAAGAGAAGTA |
| FGFR2.11.2.FGFR2.2_R | chr10:123263299-123263446 | TTTGTATCCAGGTTTCGGCT |
| FGFR2.7.1.FGFR2.1_F | chr10:123247512-123247671 | GCCATCGACTTACATTGGTG |
| FGFR2.7.1.FGFR2.1_R | chr10:123247512-123247671 | TTTCCTTTTTGTTCTGGCGG |
| FGFR2.7.2.FGFR2.2_F | chr10:123247466-123247602 | CTGGAACATTCTGAGCCTCA |
| FGFR2.7.2.FGFR2.2_R | chr10:123247466-123247602 | TCATCGAGATTTAGCAGCCA |
| FGFR2.2.1.FGFR2.1_F | chr10:123239377-123239535 | GCAGACACAGTCATTCATGT |
| FGFR2.2.1.FGFR2.1_R | chr10:123239377-123239535 | GTCTCTCTTCCCTTCTTTCAG |
| FGFR2.2.2.FGFR2.2_F | chr10:123239293-123239426 | ACAAGTGGAGACAACAAGCT |
| FGFR2.2.2.FGFR2.2_R | chr10:123239293-123239426 | TTTTTCTCCAGACCCCATGC |
| FGFR2.17.1.FGFR2.1_F | chr10:123310834-123310993 | AATGCGATGCTCCTGCTT |
| FGFR2.17.1.FGFR2.1_R | chr10:123310834-123310993 | GCTGTCCATCAGTATACATTCT |
| FGFR2.17.2.FGFR2.2_F | chr10:123310739-123310895 | GCGAGACTCCATCGCAAAAA |
| FGFR2.17.2.FGFR2.2_R | chr10:123310739-123310895 | GGCCAACACTGTCAAGTTTC |
| FGFR2.1.3.FGFR2.1_F | chr10:123239033-123239185 | TCTGTAAGTGTGTGCTGACA |

| | | |
|---|---|---|
| FGFR2.1.3.FGFR2.1_R | chr10:123239033-123239185 | TGAAAACTTGTAATCTTCCCCA |
| FGFR2.18.1.FGFR2.1_F | chr10:123324016-123324161 | GAGCCGGGCAGTTACTTAC |
| FGFR2.18.1.FGFR2.1_R | chr10:123324016-123324161 | TGCTATGGAGAAGGTCTCAG |
| FGFR2.13.1.FGFR2.1_F | chr10:123276828-123276985 | ACCCAGAGAGAAAGAACAGTA |
| FGFR2.13.1.FGFR2.1_R | chr10:123276828-123276985 | CCTTTTCTTTTGCTTCCCTTG |
| FGFR2.13.2.FGFR2.2_F | chr10:123276682-123276837 | GTGTGTGAATTTCCAAGGCA |
| FGFR2.13.2.FGFR2.2_R | chr10:123276682-123276837 | TCTGCATGGTTGACAGTTCT |
| FGFR2.16.1.FGFR2.1_F | chr10:123298052-123298212 | ACGTTCATGCTTTCAAACGA |
| FGFR2.16.1.FGFR2.1_R | chr10:123298052-123298212 | GGTACGAAACCAGCACTG |
| FGFR2.16.2.FGFR2.2_F | chr10:123298075-123298233 | AAGCAAGAATGGGCTGGTAT |
| FGFR2.16.2.FGFR2.2_R | chr10:123298075-123298233 | CCGCCTTTGCTTTGATCTTT |
| FGFR2.6.1.FGFR2.1_F | chr10:123246836-123246991 | TATTTTTGCAGCTCAAGCCC |
| FGFR2.6.1.FGFR2.1_R | chr10:123246836-123246991 | TAGCCCTATTGAGCCTGCTA |
| FGFR2.3.1.FGFR2.1_F | chr10:123241664-123241821 | CCTGACCAACTTTTCCCAGT |
| FGFR2.3.1.FGFR2.1_R | chr10:123241664-123241821 | ACAGCTGACCTTCAGAAGTT |
| FGFR2.9.1.FGFR2.1_F | chr10:123257996-123258154 | ATTTGGGCGAATGCAGTTTT |
| FGFR2.9.1.FGFR2.1_R | chr10:123257996-123258154 | CTGCCCATGAGTTAGAGGAA |
| FGFR2.12.1.FGFR2.1_F | chr10:123274661-123274821 | CGCAGGGGGATACGTTT |
| FGFR2.12.1.FGFR2.1_R | chr10:123274661-123274821 | CAATCTAGCGCCTGGAAGAG |
| FGFR2.12.2.FGFR2.2_F | chr10:123274733-123274878 | TTCTTCATTCGGCACAGGAT |
| FGFR2.12.2.FGFR2.2_R | chr10:123274733-123274878 | GTCAGTCTGGTGTGCTAACT |
| FGFR2.12.2.FGFR2.3_F | chr10:123274556-123274712 | ACAGAAGTCGATGGCATCAA |
| FGFR2.12.2.FGFR2.3_R | chr10:123274556-123274712 | ATCCTGTGCCGAATGAAGAA |
| FGFR2.15.1.FGFR2.1_F | chr10:123279517-123279677 | GAACCTTGAGGTAGGGCAG |
| FGFR2.15.1.FGFR2.1_R | chr10:123279517-123279677 | CTCTCTCCACCAGAGCGAT |
| FGFR2.15.2.FGFR2.2_F | chr10:123279623-123279774 | CAAACTCTACGTCTCCTCCG |
| FGFR2.15.2.FGFR2.2_R | chr10:123279623-123279774 | CTTGAGGCTTTTCTGGCATG |
| FGFR2.15.2.FGFR2.3_F | chr10:123279418-123279577 | CCTCTCTCAACTCCAACAGG |
| FGFR2.15.2.FGFR2.3_R | chr10:123279418-123279577 | AAGGTTTACAGTGATGCCCA |
| FGFR2.14.1.FGFR2.1_F | chr10:123278205-123278364 | AGCATTGTTACCTTGCTGTT |
| FGFR2.14.1.FGFR2.1_R | chr10:123278205-123278364 | TCCTGGTTGGCCGTTATATT |
| FGFR2.14.2.FGFR2.2_F | chr10:123278117-123278266 | ATCCTATAAGCTGCCTGCAG |
| FGFR2.14.2.FGFR2.2_R | chr10:123278117-123278266 | GCGGATGCTGGGGAATATAT |
| FGFR2.20.1.FGFR2.1_F | chr10:123353233-123353388 | AAATGACTTACCTTCTGGCTC |
| FGFR2.20.1.FGFR2.1_R | chr10:123353233-123353388 | CCGTAGAGGAAGTGTGCAG |
| FGFR2.20.2.FGFR2.2_F | chr10:123353181-123353325 | TTAACAATCTGCCCCCAGAC |
| FGFR2.20.2.FGFR2.2_R | chr10:123353181-123353325 | TTGGTACCGTAACCATGGTC |
| FGFR2.8.1.FGFR2.1_F | chr10:123256066-123256225 | CTTTTTGGGAAGCCAAGTACT |
| FGFR2.8.1.FGFR2.1_R | chr10:123256066-123256225 | CTCAACAGGGCCTCTCTAT |
| FGFR2.8.2.FGFR2.2_F | chr10:123256207-123256348 | TTCTCGGAGGTTGCCTTTAG |
| FGFR2.8.2.FGFR2.2_R | chr10:123256207-123256348 | CCAGCCGCTGTATGTTAAAC |
| FGFR2.8.2.FGFR2.3_F | chr10:123256037-123256186 | CCCAGCCAAGTAGAATGTGA |
| FGFR2.8.2.FGFR2.3_R | chr10:123256037-123256186 | CTAAAGGCAACCTCCGAGAA |
| FGFR2.4.1.FGFR2.1_F | chr10:123243190-123243347 | AAGACAACCAAGGACAAGGG |
| FGFR2.4.1.FGFR2.1_R | chr10:123243190-123243347 | CACGTACCCAGTGCATATGA |
| FGFR2.10.1.FGFR2.1_F | chr10:123260309-123260466 | GATAAGACTCTCCACCCAGC |
| FGFR2.10.1.FGFR2.1_R | chr10:123260309-123260466 | GTGCTAATCCCCTATTTACACA |
| FGFR2.19.1.FGFR2.1_F | chr10:123324962-123325123 | GCCAACTCACCTGTGACATT |
| FGFR2.19.1.FGFR2.1_R | chr10:123324962-123325123 | CTGTTGAAAGATGCCGCC |
| FGFR2.19.2.FGFR2.2_F | chr10:123325119-123325272 | CCCCATCCTTAGTCCAACTG |
| FGFR2.19.2.FGFR2.2_R | chr10:123325119-123325272 | TGACCTTTGTTGGACGTTCT |
| FGFR2.19.2.FGFR2.3_F | chr10:123324865-123325012 | ATGCCTGGCATCCAATTACA |
| FGFR2.19.2.FGFR2.3_R | chr10:123324865-123325012 | CTAGAGACTCCGGCCTCTAT |
| FGFR2.5.1.FGFR2.1_F | chr10:123244907-123245055 | CAGGAAAGACAACAGCCCTT |
| FGFR2.5.1.FGFR2.1_R | chr10:123244907-123245055 | TAATGCCGTGCTTTCTCCTT |
| APC.11.36.APC.1_F | chr5:112157564-112157710 | CATTGCTCTTCAAATAACAAAGCA |
| APC.11.36.APC.1_R | chr5:112157564-112157710 | TCCACCAGTAATTGTCTATGTCA |
| APC.7.1.APC.1_F | chr5:112128079-112128235 | GCTTTTAAGTGGTAGCCATAGT |
| APC.7.1.APC.1_R | chr5:112128079-112128235 | CCCACAAACAAGAAAGGCAA |
| APC.2.1.APC.1_F | chr5:112090616-112090772 | GCAGCTTCATATGATCAGTTGT |
| APC.2.1.APC.1_R | chr5:112090616-112090772 | ACCTCAAGTTTACAAGAGGGA |
| APC.2.2.APC.2_F | chr5:112090585-112090717 | TATAGGTCCAAGGGTAGCCA |
| APC.2.2.APC.2_R | chr5:112090585-112090717 | AGTCACAGTCTTGATACCTTCA |
| APC.1.1.APC.1_F | chr5:112043465-112043614 | CCTTTGCCCGCTTCTGT |
| APC.1.1.APC.1_R | chr5:112043465-112043614 | AAGCCGAGGAAGCTTTGC |
| APC.1.2.APC.2_F | chr5:112043384-112043544 | GGTGAGGAAGGTGAAGCAC |
| APC.1.2.APC.2_R | chr5:112043384-112043544 | CAGTGGCCAGAAGTACGAG |

| | | |
|---|---|---|
| APC.16.1.APC.1_F | chr5:112173359-112173519 | ACTTTGTGGAATCTCTCAGCA |
| APC.16.1.APC.1_R | chr5:112173359-112173519 | AAGCTTGAGCCAGGAGAC |
| APC.16.2.APC.2_F | chr5:112173219-112173371 | ACTGCATACACATTGTGACC |
| APC.16.2.APC.2_R | chr5:112173219-112173371 | GTCCCATAATGCTTCCTGGT |
| APC.16.2.APC.3_F | chr5:112173580-112173739 | CAAAAAGCCCTAGAAGCAGA |
| APC.16.2.APC.3_R | chr5:112173580-112173739 | GAAAGGACAGTCATGTTGCC |
| APC.16.3.APC.4_F | chr5:112173473-112173627 | GAAGTGCTGCAGCTTTAAGG |
| APC.16.3.APC.4_R | chr5:112173473-112173627 | GATGAGATGCCTTGGGACTT |
| APC.16.3.APC.5_F | chr5:112173760-112173919 | GGCAACATGACTGTCCTTTC |
| APC.16.3.APC.5_R | chr5:112173760-112173919 | TGGTGGAGATCTGCAAACC |
| APC.16.4.APC.6_F | chr5:112173648-112173782 | AAGTCCCAAGGCATCTCATC |
| APC.16.4.APC.6_R | chr5:112173648-112173782 | TGATGAAGAGGAGCTGGGTA |
| APC.16.4.APC.7_F | chr5:112174240-112174399 | GGTCAAATGAAACCCTCGAT |
| APC.16.4.APC.7_R | chr5:112174240-112174399 | GGACTTTGCCTTCCAGAGTT |
| APC.16.5.APC.8_F | chr5:112174410-112174569 | TGAGCAGTTGAACTCTGGAA |
| APC.16.5.APC.8_R | chr5:112174410-112174569 | AAACACATTCCTGCTGTCCA |
| APC.16.6.APC.9_F | chr5:112174299-112174437 | TTATGGTCAATACCCAGCCG |
| APC.16.6.APC.9_R | chr5:112174299-112174437 | TTATGTGTTTGGGTCTTGCC |
| APC.16.6.APC.10_F | chr5:112174640-112174799 | CAGAAACAAATCGAGTGGGT |
| APC.16.6.APC.10_R | chr5:112174640-112174799 | GGCTGATCCACATGACGTT |
| APC.16.7.APC.11_F | chr5:112174538-112174666 | TCCTGTTTATACTGAGAGCACT |
| APC.16.7.APC.11_R | chr5:112174538-112174666 | TCTTGACACAAAGACTGGCT |
| APC.16.7.APC.12_F | chr5:112174819-112174979 | AAACGTCATGTGGATCAGCC |
| APC.16.7.APC.12_R | chr5:112174819-112174979 | GCAGAACTTGGATGGAGCT |
| APC.16.8.APC.13_F | chr5:112174687-112174839 | AGCCAGTCTTTGTGTCAAGA |
| APC.16.8.APC.13_R | chr5:112174687-112174839 | TGATGAAGGAATATCTGTGGCA |
| APC.16.8.APC.14_F | chr5:112174999-112175159 | AGCTCCATCCAAGTTCTGC |
| APC.16.8.APC.14_R | chr5:112174999-112175159 | GCTTCCTGTGTCGTCTGAT |
| APC.16.9.APC.15_F | chr5:112174862-112174999 | TGCCACAGATATTCCTTCATCA |
| APC.16.9.APC.15_R | chr5:112174862-112174999 | GGCTGACCACTTCTACTCTG |
| APC.16.9.APC.16_F | chr5:112175260-112175420 | GAAGATCCTGTGAGCGAAGT |
| APC.16.9.APC.16_R | chr5:112175260-112175420 | ATGAGTGGGGTCTCCTGAA |
| APC.16.10.APC.17_F | chr5:112175149-112175277 | CTTTGTCATCAGCTGAAGATGA |
| APC.16.10.APC.17_R | chr5:112175149-112175277 | TGCTGGATTTGGTTCTAGGG |
| APC.16.10.APC.18_F | chr5:112175421-112175580 | AAAGTCCACCTGAACACTATG |
| APC.16.10.APC.18_R | chr5:112175421-112175580 | ACTTCTGCTTGGTGGCATG |
| APC.16.11.APC.19_F | chr5:112175631-112175791 | CCACCTCCTCAAACAGCTC |
| APC.16.11.APC.19_R | chr5:112175631-112175791 | CTCAGGCTGGATGAACAAGA |
| APC.16.12.APC.20_F | chr5:112175520-112175677 | CTCCGTTCAGAGTGAACCAT |
| APC.16.12.APC.20_R | chr5:112175520-112175677 | TGCTTAGGTCCACTCTCTCT |
| APC.16.12.APC.21_F | chr5:112175882-112176041 | ATGCCTCCAGTTCAGGAA |
| APC.16.12.APC.21_R | chr5:112175882-112176041 | GCTTTACGTGATGACTTTGTTG |
| APC.16.13.APC.22_F | chr5:112175749-112175897 | CAGGTTCTTCCAGATGCTGA |
| APC.16.13.APC.22_R | chr5:112175749-112175897 | AGGCTGCTCTGATTCTGTTT |
| APC.16.13.APC.23_F | chr5:112176052-112176211 | CTGCCATGCCAACAAAGTC |
| APC.16.13.APC.23_R | chr5:112176052-112176211 | TGTCCCTTCAACACAATACAC |
| APC.16.14.APC.24_F | chr5:112175918-112176073 | AAACAGAATCAGAGCAGCCT |
| APC.16.14.APC.24_R | chr5:112175918-112176073 | AATTTTGAAGCAGTCTGGGC |
| APC.16.14.APC.25_F | chr5:112176209-112176371 | CGGGGGATGATATGCCA |
| APC.16.14.APC.25_R | chr5:112176209-112176371 | AGCCTCATCTGTACTTCTGC |
| APC.16.15.APC.26_F | chr5:112176380-112176541 | TTCCTACAGAAGGCAGAAGT |
| APC.16.15.APC.26_R | chr5:112176380-112176541 | GACGCAGATGCTTGCTG |
| APC.16.16.APC.27_F | chr5:112176322-112176481 | GCTGGAGAAGGAGTTAGAGG |
| APC.16.16.APC.27_R | chr5:112176322-112176481 | CTTTTCCCTTTGGGCATAGC |
| APC.16.16.APC.28_F | chr5:112176922-112177081 | GATGTTGACCTTTCCAGGGA |
| APC.16.16.APC.28_R | chr5:112176922-112177081 | GATGACTGGGGAAAAGTGGA |
| APC.16.17.APC.29_F | chr5:112177111-112177271 | CCCCAGTCATCCAAAGACAT |
| APC.16.17.APC.29_R | chr5:112177111-112177271 | CTTGGTTCTCCCTGTGAGT |
| APC.16.17.APC.30_F | chr5:112176809-112176961 | AGAGTCAGAGGAAGTTTTGCT |
| APC.16.17.APC.30_R | chr5:112176809-112176961 | TGGCTGGTAACTTTAGCCTC |
| APC.16.18.APC.31_F | chr5:112176982-112177122 | GAGGCTAAAGTTACCAGCCA |
| APC.16.18.APC.31_R | chr5:112176982-112177122 | ACTTTTCATCAGTTGCTGCC |
| APC.16.18.APC.32_F | chr5:112177440-112177601 | GCTCCGCAATGCCAAAAA |
| APC.16.18.APC.32_R | chr5:112177440-112177601 | GGAATTTGCACCTTCCTGAA |
| APC.16.19.APC.33_F | chr5:112177622-112177781 | TTCAGGAAGGTGCAAATTCC |
| APC.16.19.APC.33_R | chr5:112177622-112177781 | TGGTTTTAGAATTCGTGGGC |
| APC.16.19.APC.34_F | chr5:112177291-112177444 | GACTCACAGGGAGAACCAAG |

| | | |
|---|---|---|
| APC.16.19.APC.34_R | chr5:112177291-112177444 | CCCTTGAGTCTTGAAGGCTT |
| APC.16.20.APC.35_F | chr5:112177582-112177711 | TGGTCTATCCCCTGATTCAGA |
| APC.16.20.APC.35_R | chr5:112177582-112177711 | GGTGATCCCAGAGAGATTCC |
| APC.16.20.APC.36_F | chr5:112177269-112177398 | TATCAAAGAGACTGAGCCCC |
| APC.16.20.APC.36_R | chr5:112177269-112177398 | TACATTCCTGCAACAGGTCA |
| APC.16.20.APC.37_F | chr5:112177801-112177961 | GGCCCACGAATTCTAAAACC |
| APC.16.20.APC.37_R | chr5:112177801-112177961 | ATTGTCCTGCCTCGAGAGA |
| APC.16.21.APC.38_F | chr5:112177653-112177800 | TTTACATCAAGCTGCTGCTG |
| APC.16.21.APC.38_R | chr5:112177653-112177800 | TCCAATGTACTTTTCTCCCCT |
| APC.16.21.APC.39_F | chr5:112177972-112178131 | ATGCCTTCAATCTCTCGAGG |
| APC.16.21.APC.39_R | chr5:112177972-112178131 | TGGCAACAGGGCTTAATTC |
| APC.16.22.APC.40_F | chr5:112177959-112178110 | CCTTCAAGCAAACATGCCTT |
| APC.16.22.APC.40_R | chr5:112177959-112178110 | GATTTCACAGATGGCTTGGC |
| APC.16.22.APC.41_F | chr5:112178161-112178322 | TGTTGCCAGGCAGACATC |
| APC.16.22.APC.41_R | chr5:112178161-112178322 | GGGATGATGTCCTTGGAAGT |
| APC.16.23.APC.42_F | chr5:112178089-112178226 | AGCCCTAGTGAAGGTCAAAC |
| APC.16.23.APC.42_R | chr5:112178089-112178226 | TACTTAATGGTTGCTGGGCA |
| APC.16.23.APC.43_F | chr5:112178613-112178772 | ACCTGTATTAGTACGCCAGT |
| APC.16.23.APC.43_R | chr5:112178613-112178772 | CCTGAACAGACGAATGTGTG |
| APC.16.24.APC.44_F | chr5:112178793-112178952 | CACACATTCGTCTGTTCAGG |
| APC.16.24.APC.44_R | chr5:112178793-112178952 | CTCGAGGAAGGGATGATGAA |
| APC.16.24.APC.45_F | chr5:112178457-112178612 | CAGGTTTATCCAAGAATGCCA |
| APC.16.24.APC.45_R | chr5:112178457-112178612 | GGAGCTTCTTTGATGAAAGTTG |
| APC.16.25.APC.46_F | chr5:112178715-112178855 | ATCTAGACCAGCTTCTCCCA |
| APC.16.25.APC.46_R | chr5:112178715-112178855 | AATATCATGGCGCTTTGCTG |
| APC.16.25.APC.47_F | chr5:112178303-112178458 | CCCTGGTAGAAATGGAATAAGT |
| APC.16.25.APC.47_R | chr5:112178303-112178458 | CAGACTCACTTCTTGGAATACT |
| APC.16.25.APC.48_F | chr5:112179243-112179402 | TTATCAAATGGCACCTGCTG |
| APC.16.25.APC.48_R | chr5:112179243-112179402 | CACTGCCATTACCCACATTT |
| APC.16.26.APC.49_F | chr5:112179423-112179582 | AAATGTGGGTAATGGCAGTG |
| APC.16.26.APC.49_R | chr5:112179423-112179582 | TGTGTTTGCTTGAGCTGCTA |
| APC.16.26.APC.50_F | chr5:112179122-112179251 | ACCAAGTATCCGCAAAAGGA |
| APC.16.26.APC.50_R | chr5:112179122-112179251 | TCTCACCCAAACATCCTCTG |
| APC.16.27.APC.51_F | chr5:112179583-112179742 | GAACGTACCCCATTCAGTTC |
| APC.16.27.APC.51_R | chr5:112179583-112179742 | TCTGTGCTGTCAGTTTTGGA |
| APC.16.27.APC.52_F | chr5:112179324-112179463 | TGGAAGATCTCCCACAGGTA |
| APC.16.27.APC.52_R | chr5:112179324-112179463 | GGGGCATCCACCTGAATAAA |
| APC.16.28.APC.53_F | chr5:112178952-112179111 | AAACGTGAGCACAGCAAAC |
| APC.16.28.APC.53_R | chr5:112178952-112179111 | TTCTCCATGTTCCTTTTGCG |
| APC.16.28.APC.54_F | chr5:112176537-112176690 | CGTGTGAAAAAGATAATGGACC |
| APC.16.28.APC.54_R | chr5:112176537-112176690 | TCTGAGAAAACTCTCTCAGCA |
| APC.16.28.APC.55_F | chr5:112173920-112174069 | CAGGAACTTCTTCAAAGCGA |
| APC.16.28.APC.55_R | chr5:112173920-112174069 | TTTGAATGTGTATGGGCAGC |
| APC.16.29.APC.56_F | chr5:112179037-112179181 | TGCTTCATCAGAATCCAGTGA |
| APC.16.29.APC.56_R | chr5:112179037-112179181 | CCATTTGTAGCACCTGAGGA |
| APC.16.30.APC.57_F | chr5:112174069-112174219 | GAAATGCACTTAGAAGAAGCTC |
| APC.16.30.APC.57_R | chr5:112174069-112174219 | ATCGAGGGTTTCATTTGACC |
| APC.16.31.APC.58_F | chr5:112174148-112174278 | GACATGTTCTATGCCTTATGCC |
| APC.16.31.APC.58_R | chr5:112174148-112174278 | CGGCTGGGTATTGACCATAA |
| APC.16.33.APC.59_F | chr5:112176662-112176815 | ACTGAATATAGGACACGTGTAAGA |
| APC.16.33.APC.59_R | chr5:112176662-112176815 | AGGCGTGTAATGATGAGGTG |
| APC.16.36.APC.60_F | chr5:112179681-112179823 | TAGGAAAAGCAGCGCAGATA |
| APC.16.36.APC.60_R | chr5:112179681-112179823 | TCTTAGTTTCATTCTTCCTCTCTT |
| APC.13.1.APC.1_F | chr5:112163592-112163742 | GCTTGGCTTCAAGTTGTCTT |
| APC.13.1.APC.1_R | chr5:112163592-112163742 | GAGACCCTGCCTCAAAGAAA |
| APC.6.37.APC.1_F | chr5:112116384-112116618 | TCATGCACCATGACTGACGT |
| APC.6.37.APC.1_R | chr5:112116384-112116618 | AGCTCTTCGCTGTTTTATCACT |
| APC.3.37.APC.1_F | chr5:112101922-112102180 | GGTGCGTGCTTTGAGAGTGA |
| APC.3.37.APC.1_R | chr5:112101922-112102180 | ACCCAAATCGAGAGAAGCTGT |
| APC.9.33.APC.1_F | chr5:112151141-112151293 | GGAGTACCTTAACATGATGTTATCT |
| APC.9.33.APC.1_R | chr5:112151141-112151293 | ACCAGGGTTTGTAATCTTCTGT |
| APC.12.28.APC.1_F | chr5:112162794-112162926 | TGTCTTTTTCCTCTTGCCCT |
| APC.12.28.APC.1_R | chr5:112162794-112162926 | ACCTTGTTGGCTACATCTCC |
| APC.12.29.APC.2_F | chr5:112162917-112163064 | GCTGGAATGGCTTTGACAAA |
| APC.12.29.APC.2_R | chr5:112162917-112163064 | CGAATGTGAAGCACAGGTTT |
| APC.15.34.APC.1_F | chr5:112170646-112170797 | GATGACCCATATTCTGTTTCTTACT |
| APC.15.34.APC.1_R | chr5:112170646-112170797 | ATCCCACCTCCACTTTCAAT |

| | | |
|---|---|---|
| APC.15.35.APC.2_F | chr5:112170774-112170906 | TTGGCACTCTTACTTACCGG |
| APC.15.35.APC.2_R | chr5:112170774-112170906 | GCTTACAATTAGGTCTTTTTGAGAG |
| APC.14.37.APC.1_F | chr5:112164563-112164722 | TGATTAATTTGCAGGTTATTGCGA |
| APC.14.37.APC.1_R | chr5:112164563-112164722 | TGGCTAAAAGAAGGCAGCAA |
| APC.14.38.APC.2_F | chr5:112164419-112164568 | GCCTCCCAAAGTGATAGGAT |
| APC.14.38.APC.2_R | chr5:112164419-112164568 | TCGCCAAGACAAATTCCTCA |
| APC.8.33.APC.1_F | chr5:112136976-112137123 | ACTGATGTTAACTCCATCTTAACAG |
| APC.8.33.APC.1_R | chr5:112136976-112137123 | CCATCTTGCTTCATACTTTTCTGA |
| APC.4.1.APC.1_F | chr5:112102956-112103109 | ATGTCCCTCCGTTCTTATGG |
| APC.4.1.APC.1_R | chr5:112102956-112103109 | TGGAGTACACAAGGCAATGT |
| APC.4.37.APC.2_F | chr5:112102730-112102986 | TACCCTGACCCAAGTGGACT |
| APC.4.37.APC.2_R | chr5:112102730-112102986 | GGAACAGGACTGCACTCTCC |
| APC.10.1.APC.1_F | chr5:112154742-112154902 | GCGAACTTTGCTAGCTATGT |
| APC.10.1.APC.1_R | chr5:112154742-112154902 | TGTCATCAGGCTGTGAGTG |
| APC.10.2.APC.2_F | chr5:112154903-112155054 | CAGCACTCCACAACATCATT |
| APC.10.2.APC.2_R | chr5:112154903-112155054 | ACATGCACTACGATGTACACT |
| APC.10.2.APC.3_F | chr5:112154663-112154796 | CTAAACTCATTTGGCCCACAG |
| APC.10.2.APC.3_R | chr5:112154663-112154796 | TGCCATGTAAAAGCTGGATG |
| APC.5.32.APC.1_F | chr5:112111281-112111436 | ATTGCTCTTCTGCAGTCTTT |
| APC.5.32.APC.1_R | chr5:112111281-112111436 | AAGTTGTACTGCCAAGTTACTT |
| ERBB2.11.1.ERBB2.1_F | chr17:37871458-37871617 | GAGTGAAGGCATTCATGGTG |
| ERBB2.11.1.ERBB2.1_R | chr17:37871458-37871617 | AACAGGATGCAGAGACAGAG |
| ERBB2.21.1.ERBB2.1_F | chr17:37880989-37881149 | TGTCCCCAGGAAGCATACG |
| ERBB2.21.1.ERBB2.1_R | chr17:37880989-37881149 | CATACCTTGGCAATCTGCAT |
| ERBB2.21.2.ERBB2.2_F | chr17:37881111-37881268 | TAGACCATGTCCGGGAAAAC |
| ERBB2.21.2.ERBB2.2_R | chr17:37881111-37881268 | CACCCATGTAGACCTTCTGG |
| ERBB2.21.2.ERBB2.3_F | chr17:37880915-37881059 | CTGTGGTTTGTGATGGTTGG |
| ERBB2.21.2.ERBB2.3_R | chr17:37880915-37881059 | TAGGGCATAAGCTGTGTCAC |
| ERBB2.7.1.ERBB2.1_F | chr17:37866294-37866454 | GATGCTGATGAGGGTCTGG |
| ERBB2.7.1.ERBB2.1_R | chr17:37866294-37866454 | CACAAAGCAGAGGCACATAC |
| ERBB2.26.1.ERBB2.1_F | chr17:37883075-37883237 | CCTTCCTCCACAGAATGAGG |
| ERBB2.26.1.ERBB2.1_R | chr17:37883075-37883237 | TGGTAGATGAGCTGCGG |
| ERBB2.26.2.ERBB2.2_F | chr17:37883204-37883350 | CTTCTTCTGTCCAGACCCTG |
| ERBB2.26.2.ERBB2.2_R | chr17:37883204-37883350 | AATGGGGTTTCCTTGAGAGG |
| ERBB2.26.2.ERBB2.3_F | chr17:37883002-37883147 | GATGAGTCCAGTATGCCAGG |
| ERBB2.26.2.ERBB2.3_R | chr17:37883002-37883147 | GATACTCCTCAGCATCCACC |
| ERBB2.2.8.ERBB2.1_F | chr17:37856491-37856657 | GAGCCGCAGTGAGCAC |
| ERBB2.2.8.ERBB2.1_R | chr17:37856491-37856657 | TAGGAGAGCTCGTCCGG |
| ERBB2.17.1.ERBB2.1_F | chr17:37875950-37876102 | AATGGGGATAATGACCCAGC |
| ERBB2.17.1.ERBB2.1_R | chr17:37875950-37876102 | GGCAATGAAGGGTACATCCT |
| ERBB2.22.1.ERBB2.1_F | chr17:37881310-37881457 | TCCCATTCCAGGGGATGAG |
| ERBB2.22.1.ERBB2.1_R | chr17:37881310-37881457 | TCCTTGGTCCTTCACCTAAC |
| ERBB2.22.2.ERBB2.2_F | chr17:37881293-37881430 | AAGGTCTACATGGGTGCTTC |
| ERBB2.22.2.ERBB2.2_R | chr17:37881293-37881430 | CCATCTGCATGGTACTCTGT |
| ERBB2.1.1.ERBB2.1_F | chr17:37855709-37855851 | GGGCAACAAGAGCAAAAGTT |
| ERBB2.1.1.ERBB2.1_R | chr17:37855709-37855851 | ATAGTCAAGGAGGGGGATGT |
| ERBB2.18.4.ERBB2.1_F | chr17:37879552-37879692 | AAACTAGCCCTCAATCCCTG |
| ERBB2.18.4.ERBB2.1_R | chr17:37879552-37879692 | TCCGTTTCCTGCAGCAG |
| ERBB2.18.5.ERBB2.2_F | chr17:37879682-37879837 | GCAGAAGATCCGGAAGTACA |
| ERBB2.18.5.ERBB2.2_R | chr17:37879682-37879837 | TCTCTTTCAGGATCCGCATC |
| ERBB2.23.1.ERBB2.1_F | chr17:37881563-37881717 | ACACACAGTTGGAGGACTTC |
| ERBB2.23.1.ERBB2.1_R | chr17:37881563-37881717 | CGTAATTCTCCCCATCCCAG |
| ERBB2.13.1.ERBB2.1_F | chr17:37871993-37872156 | GCCCACCTTTCTCCCATAG |
| ERBB2.13.1.ERBB2.1_R | chr17:37871993-37872156 | GTTGGCAGTGTGGAGCA |
| ERBB2.13.2.ERBB2.2_F | chr17:37872108-37872267 | ATAACACCCACCTCTGCTTC |
| ERBB2.13.2.ERBB2.2_R | chr17:37872108-37872267 | GTCTGCACAAGTCCAAGAAC |
| ERBB2.16.1.ERBB2.1_F | chr17:37873588-37873743 | AGGAGGCTGACCAGTGT |
| ERBB2.16.1.ERBB2.1_R | chr17:37873588-37873743 | CCTTTCTGCAGAAAAGACCG |
| ERBB2.16.2.ERBB2.2_F | chr17:37873442-37873598 | GTGAAGAGCAAGGGTGTTTG |
| ERBB2.16.2.ERBB2.2_R | chr17:37873442-37873598 | AGGGAGGGTCCTTATAGTGG |
| ERBB2.27.1.ERBB2.1_F | chr17:37883647-37883803 | GGCTGGCTCCGATGTATTT |
| ERBB2.27.1.ERBB2.1_R | chr17:37883647-37883803 | TCTGCTTAGACTGGACTCCA |
| ERBB2.27.2.ERBB2.2_F | chr17:37883495-37883646 | CTGTCACCTTCCATGGAGTC |
| ERBB2.27.2.ERBB2.2_R | chr17:37883495-37883646 | CCATTCCCAGGTCACCATC |
| ERBB2.25.1.ERBB2.1_F | chr17:37882805-37882965 | TAGCATGCTGACCTCCCT |
| ERBB2.25.1.ERBB2.1_R | chr17:37882805-37882965 | ATCTCTCCTTCCCACCCTC |
| ERBB2.6.1.ERBB2.1_F | chr17:37866066-37866222 | GTCTCTGGTTCTGTCCTCAG |

| | | |
|---|---|---|
| ERBB2.6.1.ERBB2.1_R | chr17:37866066-37866222 | ACATCCCAAGCCTGTTATCC |
| ERBB2.28.1.ERBB2.1_F | chr17:37883942-37884101 | CACCTTCTCTTGACCTTTCAG |
| ERBB2.28.1.ERBB2.1_R | chr17:37883942-37884101 | CAAGTACTCGGGGTTCTCC |
| ERBB2.28.2.ERBB2.2_F | chr17:37884120-37884283 | TGGAGAACCCCGAGTACTT |
| ERBB2.28.2.ERBB2.2_R | chr17:37884120-37884283 | GGTTCACACTGGCACGT |
| ERBB2.28.3.ERBB2.3_F | chr17:37884277-37884419 | CAGAGAACCCAGAGTACCTG |
| ERBB2.28.3.ERBB2.3_R | chr17:37884277-37884419 | GGTTCCTTAGGACAGGTTCC |
| ERBB2.28.3.ERBB2.4_F | chr17:37884094-37884233 | AAGACGTTTTTGCCTTTGGG |
| ERBB2.28.3.ERBB2.4_R | chr17:37884094-37884233 | TAGGTGTCCCTTTGAAGGTG |
| ERBB2.3.4.ERBB2.1_F | chr17:37863235-37863380 | CTCTGACCCATCTGCTCTCT |
| ERBB2.3.4.ERBB2.1_R | chr17:37863235-37863380 | TCACCTGCAGGAAGGACA |
| ERBB2.3.5.ERBB2.2_F | chr17:37863364-37863502 | AACCTGGAACTCACCTACCT |
| ERBB2.3.5.ERBB2.2_R | chr17:37863364-37863502 | TCCTGGTTGAGAAACCACAA |
| ERBB2.9.1.ERBB2.1_F | chr17:37868210-37868370 | TTCTACGGACGTGGGATCC |
| ERBB2.9.1.ERBB2.1_R | chr17:37868210-37868370 | TGAGGAAGGATAGGACAGGG |
| ERBB2.9.2.ERBB2.2_F | chr17:37868078-37868237 | ATGCGTGGTAGGGCATTTAA |
| ERBB2.9.2.ERBB2.2_R | chr17:37868078-37868237 | ATCCTCTGCTGTCACCTCTT |
| ERBB2.9.2.ERBB2.3_F | chr17:37868401-37868562 | CCTTCCTCAGACCCTCTTG |
| ERBB2.9.2.ERBB2.3_R | chr17:37868401-37868562 | TAGCACACTGAGGAGGTGG |
| ERBB2.9.3.ERBB2.4_F | chr17:37868258-37868413 | AAGAGGTGACAGCAGAGGAT |
| ERBB2.9.3.ERBB2.4_R | chr17:37868258-37868413 | AGGGGTAGAGAGTAGAAGGC |
| ERBB2.12.1.ERBB2.1_F | chr17:37871641-37871789 | TGTCTCTGCATCCTGTTCTG |
| ERBB2.12.1.ERBB2.1_R | chr17:37871641-37871789 | CCCTCTTTCTCAGTGCTCAC |
| ERBB2.14.4.ERBB2.1_F | chr17:37872530-37872676 | CCTCCCCTAAAAGTCCCCT |
| ERBB2.14.4.ERBB2.1_R | chr17:37872530-37872676 | CCCTCATACCCCTGCAGTA |
| ERBB2.14.5.ERBB2.2_F | chr17:37872633-37872773 | CCACCCAGTGTGTCAACTG |
| ERBB2.14.5.ERBB2.2_R | chr17:37872633-37872773 | TGGCATTCACATACTCCCTG |
| ERBB2.20.1.ERBB2.1_F | chr17:37880164-37880323 | TATCCTCCTCTTTCTGCCCA |
| ERBB2.20.1.ERBB2.1_R | chr17:37880164-37880323 | AATGAAGAGAGACCAGAGCC |
| ERBB2.15.1.ERBB2.1_F | chr17:37872767-37872925 | ACCACTGTCCCTTCTCTCA |
| ERBB2.15.1.ERBB2.1_R | chr17:37872767-37872925 | CTACTGCATCTCGCCATCC |
| ERBB2.8.1.ERBB2.1_F | chr17:37866575-37866734 | AAAACAGCACAGTGAAAGCC |
| ERBB2.8.1.ERBB2.1_R | chr17:37866575-37866734 | GTTTCTCCCTGGCACTCAC |
| ERBB2.4.1.ERBB2.1_F | chr17:37864613-37864773 | TACGTGCTCATCGCTCAC |
| ERBB2.4.1.ERBB2.1_R | chr17:37864613-37864773 | CACCTGTGAGGCTTCGA |
| ERBB2.4.2.ERBB2.2_F | chr17:37864737-37864874 | GAACAATACCACCCCTGTCA |
| ERBB2.4.2.ERBB2.2_R | chr17:37864737-37864874 | CCAGAAGGGACACCATTTCT |
| ERBB2.4.2.ERBB2.3_F | chr17:37864557-37864715 | GTTCCTATTTCAGCCCCACT |
| ERBB2.4.2.ERBB2.3_R | chr17:37864557-37864715 | GACAGGGGTGGTATTGTTCA |
| ERBB2.24.4.ERBB2.1_F | chr17:37881959-37882101 | TGACCCTGTCTCTGCCTTA |
| ERBB2.24.4.ERBB2.1_R | chr17:37881959-37882101 | CTCAGCCACGCACATTTG |
| ERBB2.24.5.ERBB2.2_F | chr17:37882088-37882224 | CCATCTGCACCATTGATGTC |
| ERBB2.24.5.ERBB2.2_R | chr17:37882088-37882224 | AAGAAGGTTTTGAGGCTCCT |
| ERBB2.19.6.ERBB2.1_F | chr17:37879739-37879903 | TGAAGTCCTCCCAGCCC |
| ERBB2.19.6.ERBB2.1_R | chr17:37879739-37879903 | GCCCTGACCTTGTAGACT |
| ERBB2.19.7.ERBB2.2_F | chr17:37879889-37880030 | AAGGTGAAGGTGCTTGGATC |
| ERBB2.19.7.ERBB2.2_R | chr17:37879889-37880030 | CTCCCTTCTCCGCTGTAAC |
| ERBB2.10.1.ERBB2.1_F | chr17:37868584-37868739 | CACCTCCTCAGTGTGCTATG |
| ERBB2.10.1.ERBB2.1_R | chr17:37868584-37868739 | GGATCCTCAGGACTCTGTCT |
| ERBB2.10.2.ERBB2.2_F | chr17:37868464-37868623 | AGCCCTACAAGTGTCCCTAT |
| ERBB2.10.2.ERBB2.2_R | chr17:37868464-37868623 | ACTCCTGGATATTGGCACTG |
| ERBB2.5.1.ERBB2.1_F | chr17:37865565-37865722 | TCCTCTGATCATTGCTCACC |
| ERBB2.5.1.ERBB2.1_R | chr17:37865565-37865722 | CTGAGAGAAGAGGCAGCAG |
| PALB2.6.1.PALB2.1_F | chr16:23635293-23635429 | CCAGCTGACAGAGACAAAGA |
| PALB2.6.1.PALB2.1_R | chr16:23635293-23635429 | CCAAGCATAATTTTTGGCTGC |
| PALB2.11.1.PALB2.1_F | chr16:23649161-23649290 | AGCAGGCATAAGTGAATGGT |
| PALB2.11.1.PALB2.1_R | chr16:23649161-23649290 | GAAAACGTATTTCTGGGGCT |
| PALB2.3.1.PALB2.1_F | chr16:23625309-23625439 | TAACACACAAAGTGGTCCCA |
| PALB2.3.1.PALB2.1_R | chr16:23625309-23625439 | CTGGTTTGTTGGAAGAATGTGA |
| PALB2.7.1.PALB2.1_F | chr16:23637557-23637716 | AGCTCGAGATTCCCACTTAC |
| PALB2.7.1.PALB2.1_R | chr16:23637557-23637716 | CTGTTGCCATTGTGTCAGAA |
| PALB2.7.2.PALB2.2_F | chr16:23637580-23637739 | TGCGAAGTGCCAGGTATAAA |
| PALB2.7.2.PALB2.2_R | chr16:23637580-23637739 | ACTCGAGTGCCACTTTAACA |
| PALB2.9.1.PALB2.1_F | chr16:23640961-23641121 | TCACAGAGGAAATGGATTGTAC |
| PALB2.9.1.PALB2.1_R | chr16:23640961-23641121 | AGGCAGCCCAGCAAAAC |
| PALB2.9.2.PALB2.2_F | chr16:23641122-23641281 | ACTTGCAGGGTGGTATGTG |
| PALB2.9.2.PALB2.2_R | chr16:23641122-23641281 | CACCTGCTTTCCCCATCTTA |

| | | |
|---|---|---|
| PALB2.9.3.PALB2.3_F | chr16:23641322-23641481 | AACACATGTCTGTGGTAGGC |
| PALB2.9.3.PALB2.3_R | chr16:23641322-23641481 | ACTGAGTCCTAAACGCATGG |
| PALB2.9.4.PALB2.4_F | chr16:23641237-23641376 | CGTCCAGCAACTTCTGTAGA |
| PALB2.9.4.PALB2.4_R | chr16:23641237-23641376 | TACAAAGACGGGCCTTTCTT |
| PALB2.9.4.PALB2.5_F | chr16:23641541-23641701 | GCTTCCCTCTTTAAGATGTCTC |
| PALB2.9.4.PALB2.5_R | chr16:23641541-23641701 | ATGCTTTCACGGCTCCA |
| PALB2.9.5.PALB2.6_F | chr16:23641696-23641826 | AAACTCAGCATTCCATCCCT |
| PALB2.9.5.PALB2.6_R | chr16:23641696-23641826 | CAGATTGTCTGTTTTGTTGGGT |
| PALB2.9.5.PALB2.7_F | chr16:23641429-23641567 | GAGTTTGGCCTTTTGGGATG |
| PALB2.9.5.PALB2.7_R | chr16:23641429-23641567 | TGCTCAGAAAAACCAGTGGA |
| PALB2.12.1.PALB2.1_F | chr16:23649309-23649466 | ACAGCCCCAGAAATACGTTT |
| PALB2.12.1.PALB2.1_R | chr16:23649309-23649466 | ACTTGCCCAGTATTGTTGGT |
| PALB2.2.1.PALB2.1_F | chr16:23619214-23619365 | GCCCTGGAGGAAGACAGTA |
| PALB2.2.1.PALB2.1_R | chr16:23619214-23619365 | TCCTGACATACTCTTGACAGTC |
| PALB2.2.2.PALB2.2_F | chr16:23619151-23619284 | GCACAGTGCCTTTCAGAATG |
| PALB2.2.2.PALB2.2_R | chr16:23619151-23619284 | CTGTGCCAAAGAGAGTGAGT |
| PALB2.8.1.PALB2.1_F | chr16:23640509-23640647 | AAGACACGAGACACTGGAAG |
| PALB2.8.1.PALB2.1_R | chr16:23640509-23640647 | TGATGAGTGGGTAATGCAGG |
| PALB2.1.8.PALB2.1_F | chr16:23614755-23614910 | AGAGGCCCAATATATCCAGAA |
| PALB2.1.8.PALB2.1_R | chr16:23614755-23614910 | TGCCATTTGGGACTTACTTC |
| PALB2.1.9.PALB2.2_F | chr16:23614907-23615055 | GAGGAGGGCAGTACACTGA |
| PALB2.1.9.PALB2.2_R | chr16:23614907-23615055 | TGGGAACATGGTTTTGACCT |
| PALB2.4.1.PALB2.1_F | chr16:23632681-23632838 | AGGACCTAGAGGGAAAGCTT |
| PALB2.4.1.PALB2.1_R | chr16:23632681-23632838 | GGCTACCTAGAGACTGCTTT |
| PALB2.13.1.PALB2.1_F | chr16:23652337-23652486 | GGGGTGGTCAGATGATACTG |
| PALB2.13.1.PALB2.1_R | chr16:23652337-23652486 | CTGCTCTTTTCGTTCTGTCG |
| PALB2.10.1.PALB2.1_F | chr16:23646283-23646442 | GCTAGAAGTTGGCAAAAGTGG |
| PALB2.10.1.PALB2.1_R | chr16:23646283-23646442 | GCACAGGACAACCAAGTTC |
| PALB2.10.2.PALB2.2_F | chr16:23646443-23646602 | GAGAAGTTTCTGAGAGGTTCTT |
| PALB2.10.2.PALB2.2_R | chr16:23646443-23646602 | TGTCCAATTGCCAGAGGA |
| PALB2.10.2.PALB2.3_F | chr16:23646128-23646285 | AAGGAAGTGCCAGGCAAATA |
| PALB2.10.2.PALB2.3_R | chr16:23646128-23646285 | CCCCAGCATCAGATCATTGT |
| PALB2.10.3.PALB2.4_F | chr16:23646613-23646772 | GGCTACTTTCCTCTGGCA |
| PALB2.10.3.PALB2.4_R | chr16:23646613-23646772 | AGTGACACTCTTGATGGCAG |
| PALB2.10.4.PALB2.5_F | chr16:23646464-23646612 | AACTTGGTTGTCCTGTGCAT |
| PALB2.10.4.PALB2.5_R | chr16:23646464-23646612 | CAACACGAAGCATGTCCAAT |
| PALB2.10.4.PALB2.6_F | chr16:23647003-23647165 | TGCCTTGTGCCTCCAA |
| PALB2.10.4.PALB2.6_R | chr16:23647003-23647165 | CCAACCAGAAAAAGGTGTTGA |
| PALB2.10.5.PALB2.7_F | chr16:23647196-23647358 | CTGGTTGGGCAGTTGGT |
| PALB2.10.5.PALB2.7_R | chr16:23647196-23647358 | CTTTGGCACTGATTCACTCA |
| PALB2.10.6.PALB2.8_F | chr16:23647153-23647291 | GCCCTGGTGAAATTAGGTCT |
| PALB2.10.6.PALB2.8_R | chr16:23647153-23647291 | AATCCTGCTAGATCACCAGT |
| PALB2.10.6.PALB2.9_F | chr16:23647369-23647528 | CCCAGACAATCTGAGTGAATC |
| PALB2.10.6.PALB2.9_R | chr16:23647369-23647528 | TTAACCCTGGAGATGGCCC |
| PALB2.10.7.PALB2.10_F | chr16:23647337-23647477 | GCTACTGATTTCTTCCTGTTCC |
| PALB2.10.7.PALB2.10_R | chr16:23647337-23647477 | ACCCAAGAACATTTTCCCCA |
| PALB2.10.10.PALB2.11_F | chr16:23646758-23646912 | GGTTGACTTAGAATCTCACTTTCC |
| PALB2.10.10.PALB2.11_R | chr16:23646758-23646912 | GCCAACTGCCCACAAGT |
| PALB2.10.11.PALB2.12_F | chr16:23646892-23647037 | GGTGAGTTCATTTAGAGAACATGA |
| PALB2.10.11.PALB2.12_R | chr16:23646892-23647037 | GCAGTGAACTTACTACTCACG |
| PALB2.10.12.PALB2.13_F | chr16:23647498-23647645 | TGGGGAAAATGTTCTTGGGT |
| PALB2.10.12.PALB2.13_R | chr16:23647498-23647645 | TTTTCCTCCTCAGAACCTAAAAA |
| PALB2.10.13.PALB2.14_F | chr16:23647612-23647768 | CAGTTTCTTCATCAAGATGGGT |
| PALB2.10.13.PALB2.14_R | chr16:23647612-23647768 | GCCTGAATGAAATGTCACTGA |
| PALB2.5.1.PALB2.1_F | chr16:23634319-23634470 | TCTGCAAACGTCATGACTTC |
| PALB2.5.1.PALB2.1_R | chr16:23634319-23634470 | CTCCTCACATCACCCCATTT |
| PALB2.5.2.PALB2.2_F | chr16:23634171-23634322 | ACCTAGTGTTGATGCGGTAC |
| PALB2.5.2.PALB2.2_R | chr16:23634171-23634322 | GGGACCCTTTCTGATCAACA |

**Table S3: Universal PCR primers**

| | |
|---|---|
| Illumjna IndexF | AATGATACGGCGACCACCGAGATCTACACTCTTTCCCTACACGACGCTCTTCCGATCTCTG |
| Illumina IndexR* | CAAGCAGAAGACGGCATACGAGATXXXXXXGTGACTGGAGTTCAGACGTGTGCTCTTCCGATCTGAC |
| Sequencing Primer read1 | ACTCTTTCCCTACACGACGCTCTTCCGATCTCTG |
| Sequencing Primer read2 | ACTGGAGTTCAGACGTGTGCTCTTCCGATCTGAC |

* X represents the DNA barcode

**Table S4: Significant copy number gains and losses**

| Gene | Sample | Log2(T/N) | P-value |
|---|---|---|---|
| ABL2 | UCI8965412 | 1.62 | 0 |
| BRAF | UCI1689380 | 1.05 | 3.77E-82 |
| ERBB2 | AA1204 | 1.37 | 6.81E-148 |
| ERBB2 | AA1222 | 1.98 | 0 |
| ERBB2 | AA1247 | 2.66 | 0 |
| ERBB2 | UCI1908503 | 1.78 | 0 |
| ERBB2 | UCI8965412 | 2.42 | 0 |
| FGFR1 | AA1090 | 1.41 | 1.55E-138 |
| FGFR1 | UCI2649875 | 1.54 | 4.06E-133 |
| FGFR1OP | AA1277 | -1.41 | 2.42E-08 |
| FGFR2 | AA1277 | 2.20 | 0 |
| PIK3CA | UCI3564897 | 1.54 | 1.55E-169 |

**Table S5: Read Alignment and Coverage Statistics**

| Patient ID | Sample ID[1] | Run ID | #Reads | #Aligned | Mean Read Coverage[2] | Coverage Uniformity[3] |
|---|---|---|---|---|---|---|
| AA1025 | AA1025B | 120719_M00436_0005 | 2,238,436 | 2,161,009 | 1,049 | 96% |
| AA1025 | AA1025T | 120719_M00436_0005 | 2,749,834 | 2,707,115 | 1,384 | 95% |
| AA1077 | AA1077B | 121025_M00436_0010 | 3,767,118 | 3,374,294 | 1,625 | 94% |
| AA1077 | AA1077T | 121025_M00436_0010 | 3,014,584 | 2,942,237 | 1,529 | 91% |
| AA1090 | AA1090B | 120823_M00436_0009 | 2,049,694 | 2,003,189 | 1,064 | 92% |
| AA1090 | AA1090T | 120823_M00436_0009 | 2,291,814 | 2,232,270 | 1,170 | 85% |
| AA943 | AA943B | 120831_M00436_0012 | 3,208,654 | 3,160,224 | 1,631 | 96% |
| AA943 | AA943T | 120831_M00436_0012 | 3,875,458 | 3,764,254 | 1,869 | 95% |
| AA1188 | AA1188B | 120814_M00436_0008 | 3,621,022 | 3,554,531 | 1,874 | 95% |
| AA1188 | AA1188T | 120814_M00436_0008 | 3,106,796 | 3,005,426 | 1,553 | 95% |
| AA1204 | AA1204B | 120719_M00436_0005 | 4,048,560 | 4,003,089 | 2,105 | 95% |
| AA1204 | AA1204T | 120719_M00436_0005 | 3,070,104 | 3,034,944 | 1,568 | 91% |
| AA1222 | AA1222B | 121025_M00436_0010 | 3,555,052 | 3,171,760 | 1,536 | 93% |
| AA1222 | AA1222T | 121025_M00436_0010 | 3,056,424 | 2,989,223 | 1,569 | 87% |
| AA1247 | AA1247B | 120814_M00436_0008 | 2,980,418 | 2,937,581 | 1,570 | 95% |
| AA1247 | AA1247T | 120814_M00436_0008 | 3,436,018 | 3,389,175 | 1,821 | 86% |
| AA1267 | AA1267B | 120827_M00436_0011 | 2,437,798 | 2,392,933 | 1,249 | 94% |
| AA1267 | AA1267T | 120827_M00436_0011 | 2,803,204 | 2,770,720 | 1,477 | 93% |
| AA1277 | AA1277B | 120825_M00436_0010 | 2,265,368 | 2,206,906 | 1,026 | 94% |
| AA1277 | AA1277T | 120825_M00436_0010 | 2,896,468 | 2,851,861 | 1,412 | 84% |
| AA1307 | AA1307B | 120827_M00436_0011 | 3,828,404 | 3,798,149 | 2,040 | 95% |
| AA1307 | AA1307T | 120827_M00436_0011 | 2,671,612 | 2,626,127 | 1,371 | 92% |
| AA957 | AA957B | 120831_M00436_0012 | 3,404,866 | 3,348,375 | 1,697 | 96% |
| AA957 | AA957T | 120831_M00436_0012 | 1,487,058 | 1,455,284 | 731 | 95% |
| AA926 | AA926B | 120712_M00436_0004 | 2,738,246 | 2,710,403 | 1,462 | 95% |
| AA926 | AA926T | 120712_M00436_0004 | 2,484,200 | 2,434,466 | 1,314 | 91% |
| AA930 | AA930B | 120727_M00436_0007 | 2,628,572 | 2,540,172 | 1,318 | 95% |
| AA930 | AA930T | 120727_M00436_0007 | 3,730,366 | 3,659,703 | 1,950 | 95% |
| AA1106 | AA1106B | 120918_M00436_0001 | 3,586,450 | 3,546,087 | 1,897 | 95% |
| AA1106 | AA1106T | 120918_M00436_0001 | 3,834,160 | 3,788,715 | 2,039 | 94% |
| AA948 | AA948B | 120723_M00436_0006 | 2,760,844 | 2,701,244 | 1,360 | 96% |
| AA948 | AA948T | 120723_M00436_0006 | 3,116,402 | 3,058,760 | 1,539 | 95% |
| AA952 | AA952B | 120723_M00436_0006 | 2,130,702 | 2,039,491 | 922 | 95% |
| AA952 | AA952T | 120723_M00436_0006 | 2,701,764 | 2,629,638 | 1,287 | 95% |
| AA1367 | AA1367B | 120918_M00436_0001 | 4,132,296 | 4,103,434 | 2,225 | 95% |
| AA1367 | AA1367T | 120918_M00436_0001 | 3,575,528 | 3,514,050 | 1,872 | 93% |
| AA960 | AA960B | 120825_M00436_0010 | 2,209,072 | 2,164,943 | 1,025 | 95% |
| AA960 | AA960T | 120825_M00436_0010 | 2,368,496 | 2,318,341 | 1,025 | 92% |
| AA1515 | AA1515B | 120702_M00436_0003 | 4,018,844 | 3,985,206 | 2,194 | 87% |
| AA1515 | AA1515T | 120702_M00436_0003 | 3,170,656 | 3,147,053 | 1,735 | 81% |
| UCI1546879 | UCI1546879B | 121017_M00436_0007 | 2,737,470 | 2,568,080 | 1,152 | 96% |
| UCI1546879 | UCI1546879T | 121017_M00436_0007 | 2,512,712 | 2,458,068 | 1,246 | 96% |
| UCI1689380 | UCI1689380B | 121025_M00436_0010 | 4,325,160 | 4,011,880 | 2,015 | 93% |
| UCI1689380 | UCI1689380T | 121025_M00436_0010 | 3,617,706 | 3,523,676 | 1,784 | 90% |

| Patient ID | Sample ID[1] | Run ID | #Reads | #Aligned | Mean Read Coverage[2] | Coverage Uniformity[3] |
|---|---|---|---|---|---|---|
| UCI1804937 | UCI1804937B | 120927_M00436_0002 | 2,782,872 | 2,645,891 | 1,166 | 92% |
| UCI1804937 | UCI1804937T | 120927_M00436_0002 | 3,666,490 | 3,525,204 | 1,609 | 87% |
| UCI1821517 | UCI1821517B | 121005_M00436_0003 | 2,200,514 | 1,995,831 | 731 | 94% |
| UCI1821517 | UCI1821517T | 121005_M00436_0003 | 2,543,556 | 2,458,056 | 1,085 | 94% |
| UCI1908503 | UCI1908503B | 121005_M00436_0003 | 3,047,486 | 2,839,649 | 1,386 | 94% |
| UCI1908503 | UCI1908503T | 121005_M00436_0003 | 3,497,798 | 3,443,995 | 1,773 | 89% |
| UCI1951813 | UCI1951813B | 120927_M00436_0002 | 2,631,032 | 2,356,453 | 956 | 92% |
| UCI1951813 | UCI1951813T | 120927_M00436_0002 | 3,049,180 | 2,891,836 | 1,253 | 90% |
| UCI2008866 | UCI2008866B | 120727_M00436_0007 | 2,716,066 | 2,515,828 | 1,277 | 95% |
| UCI2008866 | UCI2008866T | 120727_M00436_0007 | 2,711,774 | 2,640,608 | 1,370 | 94% |
| UCI2076630 | UCI2076630B | 121025_M00436_0010 | 2,222,134 | 2,051,676 | 889 | 95% |
| UCI2076630 | UCI2076630T | 121025_M00436_0010 | 3,020,010 | 2,930,941 | 1,453 | 89% |
| UCI2219330 | UCI2219330B | 121019_M00436_0008 | 3,396,468 | 3,245,237 | 1,501 | 96% |
| UCI2219330 | UCI2219330T | 121019_M00436_0008 | 3,518,830 | 3,471,425 | 1,816 | 95% |
| UCI2224680 | UCI2224680B | 121017_M00436_0007 | 4,821,386 | 4,319,722 | 2,026 | 93% |
| UCI2224680 | UCI2224680T | 121017_M00436_0007 | 3,174,400 | 3,067,894 | 1,573 | 90% |
| UCI2231576 | UCI2231576B | 121023_M00436_0009 | 2,284,128 | 1,655,820 | 834 | 95% |
| UCI2231576 | UCI2231576T | 121023_M00436_0009 | 1,841,322 | 1,697,647 | 871 | 94% |
| UCI2564879 | UCI2564879B | 121017_M00436_0007 | 3,805,480 | 3,368,913 | 1,660 | 88% |
| UCI2564879 | UCI2564879T | 121017_M00436_0007 | 3,494,980 | 3,397,756 | 1,776 | 87% |
| UCI2649875 | UCI2649875B | 121008_M00436_0004 | 2,531,122 | 2,300,017 | 720 | 95% |
| UCI2649875 | UCI2649875T | 121008_M00436_0004 | 2,931,292 | 2,857,110 | 1,228 | 91% |
| UCI3165498 | UCI3165498B | 121019_M00436_0008 | 5,049,092 | 4,901,483 | 2,499 | 96% |
| UCI3165498 | UCI3165498T | 121019_M00436_0008 | 3,348,012 | 3,308,405 | 1,741 | 91% |
| UCI3564897 | UCI3564897B | 121019_M00436_0008 | 3,816,162 | 3,622,730 | 1,751 | 96% |
| UCI3564897 | UCI3564897T | 121019_M00436_0008 | 3,390,376 | 3,333,686 | 1,747 | 88% |
| UCI4216548 | UCI4216548B | 121017_M00436_0007 | 2,781,236 | 2,535,992 | 1,181 | 96% |
| UCI4216548 | UCI4216548T | 121017_M00436_0007 | 2,543,478 | 2,485,579 | 1,238 | 90% |
| UCI8965412 | UCI8965412B | 121019_M00436_0008 | 4,590,764 | 4,421,800 | 2,258 | 95% |
| UCI8965412 | UCI8965412T | 121019_M00436_0008 | 3,166,474 | 3,125,612 | 1,658 | 84% |
| UCI9135402 | UCI9135402B | 121008_M00436_0004 | 2,536,108 | 2,371,253 | 839 | 95% |
| UCI9135402 | UCI9135402T | 121008_M00436_0004 | 3,244,088 | 3,168,386 | 1,375 | 93% |

1: B suffix indicates blood germline DNA, T suffix indicates tumor DNA

2: Average number of reads per amplicon

3: Fraction of base pair  within 2 fold of the mean coverage

**Table S6: List of non-silent somatic mutations**

| | | | | | | | | | Allele Fraction (%) | |
|---|---|---|---|---|---|---|---|---|---|---|
| **Sample** | **Chr** | **Coord** | **Ref Allele** | **Alt Allele** | **Gene** | **AA Change*** | **Type** | **Effect** | **Normal** | **Tumor** |
| UCI1804937 | chr1 | 179078209 | G | C | ABL2 | D695E | MISSENSE | NON_SYNONYI | 3.87 | 5.92 |
| UCI2649875 | chr14 | 105246445 | A | C | AKT1 | L52R | MISSENSE | NON_SYNONYI | 0 | 63.21 |
| AA952 | chr5 | 112175825 | G | A | APC | D1512N | MISSENSE | NON_SYNONYI | 0.22 | 13.84 |
| AA952 | chr11 | 108204663 | G | C | ATM | E2660Q | MISSENSE | NON_SYNONYI | 0 | 13.54 |
| UCI4216548 | chr11 | 108124713 | GATCGCTGTC | G | ATM | NA | NONE | FRAME_SHIFT | 0 | 47.3 |
| UCI1908503 | chr7 | 140534596 | C | T | BRAF | G106E | MISSENSE | NON_SYNONYI | 0.29 | 13.58 |
| AA952 | chr17 | 41245012 | C | T | BRCA1 | E550K | MISSENSE | NON_SYNONYI | 0.09 | 12.59 |
| AA952 | chr17 | 41246489 | C | T | BRCA1 | W306* | NONSENSE | STOP_GAINED | 0.08 | 17.65 |
| UCI2224680 | chr13 | 32913979 | G | C | BRCA2 | L1829F | MISSENSE | NON_SYNONYI | 0 | 1.73 |
| AA1106 | chr13 | 32912745 | T | C | BRCA2 | I1418T | MISSENSE | NON_SYNONYI | 0.13 | 3.52 |
| AA1106 | chr16 | 68835732 | G | A | CDH1 | R108K | MISSENSE | NON_SYNONYI | 1.02 | 15.98 |
| AA1106 | chr16 | 68849442 | C | T | CDH1 | Q388* | NONSENSE | STOP_GAINED | 0 | 16.01 |
| AA1188 | chr16 | 68842325 | A | C | CDH1 | NA | NONE | SPLICE_SITE_ | 0.16 | 22.18 |
| AA930 | chr16 | 68846167 | G | A | CDH1 | NA | NONE | SPLICE_SITE_ | 0.17 | 23.79 |
| AA1090 | chr9 | 21971104 | G | T | CDKN2A | A85D | MISSENSE | NON_SYNONYI | 0.17 | 65.93 |
| UCI1908503 | chr10 | 96745907 | A | G | CYP2C9 | K423E | MISSENSE | NON_SYNONYI | 0.33 | 17.92 |
| AA1247 | chr17 | 37880261 | G | C | ERBB2 | D493H | MISSENSE | NON_SYNONYI | 0 | 4.85 |
| AA952 | chr17 | 37883585 | C | G | ERBB2 | S1036C | MISSENSE | NON_SYNONYI | 0 | 16.07 |
| AA1106 | chr17 | 37880220 | T | C | ERBB2 | L479S | MISSENSE | NON_SYNONYI | 0.41 | 16.6 |
| AA952 | chr17 | 37883167 | C | G | ERBB2 | L1009V | MISSENSE | NON_SYNONYI | 0.13 | 17.59 |
| AA952 | chr12 | 56495375 | G | A | ERBB3 | E1130K | MISSENSE | NON_SYNONYI | 0.15 | 16.86 |
| UCI4216548 | chr8 | 38271295 | C | G | FGFR1 | D683H | MISSENSE | NON_SYNONYI | 0.11 | 13.05 |
| UCI1908503 | chr10 | 123239488 | G | C | FGFR2 | Y666* | NONSENSE | STOP_GAINED | 0.37 | 16.86 |
| AA1367 | chr10 | 123256219 | C | G | FGFR2 | V156L | MISSENSE | NON_SYNONYI | 0.47 | 21.53 |
| AA952 | chr10 | 8106058 | T | A | GATA3 | M293K | MISSENSE | NON_SYNONYI | 0 | 12.89 |
| AA1025 | chr10 | 8115874 | C | CT | GATA3 | NA | NONE | FRAME_SHIFT | 0 | 17.49 |
| AA948 | chr10 | 8111478 | A | AC | GATA3 | NA | NONE | FRAME_SHIFT | 0.34 | 29.84 |
| AA960 | chr10 | 8111506 | A | ATGCC | GATA3 | NA | NONE | FRAME_SHIFT | 0 | 34.9 |
| AA926 | chr10 | 8111553 | C | CT | GATA3 | NA | NONE | FRAME_SHIFT | 0 | 40.59 |
| AA1090 | chr10 | 8111513 | T | TG | GATA3 | NA | NONE | FRAME_SHIFT | 0 | 43.46 |
| AA952 | chr9 | 5054787 | C | T | JAK2 | S131L | MISSENSE | NON_SYNONYI | 0 | 15.57 |
| UCI1908503 | chr9 | 5029872 | G | A | JAK2 | E106K | MISSENSE | NON_SYNONYI | 0.06 | 16.11 |
| AA952 | chr19 | 17951135 | G | C | JAK3 | I386M | MISSENSE | NON_SYNONYI | 0 | 12.77 |
| AA952 | chr2 | 47643468 | C | G | MSH2 | L112V | MISSENSE | NON_SYNONYI | 0 | 15.92 |
| UCI2564879 | chr9 | 139391413 | C | A | NOTCH1 | G2260W | MISSENSE | NON_SYNONYI | 0.03 | 35.05 |
| UCI1689380 | chr16 | 23637719 | C | G | PALB2 | NA | NONE | SPLICE_SITE_ | 0.09 | 45.22 |
| AA1204 | chr5 | 149515247 | C | T | PDGFRB | G15S | MISSENSE | NON_SYNONYI | 0.15 | 11.36 |
| UCI1951813 | chr3 | 178936091 | G | A | PIK3CA | E545K | MISSENSE | NON_SYNONYI | 0 | 6.5 |
| AA952 | chr3 | 178936091 | G | A | PIK3CA | E545K | MISSENSE | NON_SYNONYI | 0.18 | 16.47 |
| AA1204 | chr3 | 178952085 | A | G | PIK3CA | H1047R | MISSENSE | NON_SYNONYI | 0.21 | 26.31 |
| AA957 | chr3 | 178936082 | G | A | PIK3CA | E542K | MISSENSE | NON_SYNONYI | 0.2 | 27.97 |
| AA948 | chr3 | 178936091 | G | A | PIK3CA | E545K | MISSENSE | NON_SYNONYI | 0.2 | 34.12 |
| UCI2564879 | chr3 | 178927980 | T | C | PIK3CA | C420R | MISSENSE | NON_SYNONYI | 0.63 | 35.9 |
| AA1267 | chr3 | 178952085 | A | G | PIK3CA | H1047R | MISSENSE | NON_SYNONYI | 0 | 44.68 |
| UCI1908503 | chr3 | 178952085 | A | G | PIK3CA | H1047R | MISSENSE | NON_SYNONYI | 0.13 | 49.04 |
| AA1515 | chr3 | 178936091 | G | A | PIK3CA | E545K | MISSENSE | NON_SYNONYI | 0 | 69.74 |
| UCI1546879 | chr5 | 67591106 | A | G | PIK3R1 | K204E | MISSENSE | NON_SYNONYI | 1.68 | 30.26 |
| AA1106 | chr7 | 6038848 | C | T | PMS2 | R152H | MISSENSE | NON_SYNONYI | 0 | 6.32 |
| UCI2076630 | chr7 | 6029551 | G | C | PMS2 | Q236E | MISSENSE | NON_SYNONYI | 0.5 | 15.97 |
| AA1106 | chr10 | 89692856 | GAAGAT | G | PTEN | NA | NONE | FRAME_SHIFT | 0 | 5.24 |
| UCI1689380 | chr17 | 38512437 | C | A | RARA | P337T | MISSENSE | NON_SYNONYI | 0.08 | 14.35 |
| UCI2008866 | chr13 | 49050847 | AGT | A | RB1 | NA | NONE | FRAME_SHIFT | 0 | 78.52 |
| AA1367 | chr17 | 7578550 | G | T | TP53 | S116Y | MISSENSE | NON_SYNONYI | 0.16 | 36.33 |
| AA1222 | chr17 | 7578526 | C | T | TP53 | C124Y | MISSENSE | NON_SYNONYI | 0.15 | 40.37 |
| AA1204 | chr17 | 7577538 | C | T | TP53 | R116Q | MISSENSE | NON_SYNONYI | 0.23 | 41.52 |
| UCI8965412 | chr17 | 7576867 | AT | A | TP53 | NA | NONE | FRAME_SHIFT | 0 | 42.22 |
| UCI1821517 | chr17 | 7579536 | C | A | TP53 | E51* | NONSENSE | STOP_GAINED | 0 | 45.35 |
| UCI1908503 | chr17 | 7577538 | C | T | TP53 | R116Q | MISSENSE | NON_SYNONYI | 0.27 | 47.82 |
| AA1077 | chr17 | 7578505 | G | A | TP53 | P10L | MISSENSE | NON_SYNONYI | 0.23 | 48.35 |
| AA1077 | chr17 | 7578475 | G | A | TP53 | P141L | MISSENSE | NON_SYNONYI | 0.27 | 48.41 |
| AA1077 | chr17 | 7578458 | G | A | TP53 | R147C | MISSENSE | NON_SYNONYI | 0.11 | 48.53 |
| AA1307 | chr17 | 7577580 | T | C | TP53 | Y102C | MISSENSE | NON_SYNONYI | 0.48 | 56.96 |
| UCI2008866 | chr17 | 7577121 | G | A | TP53 | R141C | MISSENSE | NON_SYNONYI | 0 | 78.5 |
| UCI3165498 | chr17 | 7577094 | G | A | TP53 | R150W | MISSENSE | NON_SYNONYI | 0.2 | 79.04 |
| UCI3564897 | chr17 | 7574003 | G | A | TP53 | R331* | NONSENSE | STOP_GAINED | 0 | 79.33 |
| AA1090 | chr17 | 7578441 | G | T | TP53 | Y152* | NONSENSE | STOP_GAINED | 0 | 82.18 |
| AA1277 | chr17 | 7578382 | G | C | TP53 | S172* | NONSENSE | STOP_GAINED | 0.12 | 85.52 |
| UCI4216548 | chr17 | 7579424 | GC | G | TP53 | NA | NONE | FRAME_SHIFT | 0.12 | 85.55 |

* as reported y SNPEff

**Table S7: List of mutations which segregate into two significantly different groups of alellelic fractions in the tumors of 3 patients.**

| Patient | Gene | AA Change | TAF | Average TAF | P-value |
|---|---|---|---|---|---|
| AA 952 | APC | D1512N | 13.8 | 13.1 | 8.8E-06 |
| | ATM | E1312Q | 13.5 | | |
| | GATA3 | M294K | 12.9 | | |
| | JAK3 | I386M | 12.8 | | |
| | BRCA1 | E550K | 12.6 | | |
| | BRCA1 | W57* | 17.7 | 16.6 | |
| | ERBB2 | L1024V | 17.6 | | |
| | ERBB3 | E1189K | 16.8 | | |
| | PIK3CA | E545K | 16.5 | | |
| | ERBB2 | S1066C | 16.1 | | |
| | MSH2 | L326V | 15.9 | | |
| | JAK2 | S280L | 15.6 | | |
| AA1106 | BRCA2 | I1418T | 3.52 | 5.0 | 1.8E-04 |
| | PTEN | E114fs | 5.24 | | |
| | PMS2 | R199H | 6.32 | | |
| | CDH1 | R108K | 15.98 | 16.2 | |
| | CDH1 | Q449* | 16.01 | | |
| | ERBB2 | L755S | 16.6 | | |
| UCI1908503 | BRAF | G106E | 13.58 | 16.1 | 2.3E-05 |
| | CYP2C9 | K423E | 17.92 | | |
| | FGFR2 | Y783* | 16.86 | | |
| | JAK2 | E106K | 16.11 | | |
| | PIK3CA | H1047R | 49.04 | 48.4 | |
| | TP53 | R248Q | 47.82 | | |

**Table S8: List of non-silent germline variants**

| Sample | Chr | Cord | rsID | Ref Allele | Alt Allele | Allelic Fraction | | Gene | AA change | Type | Effect | Mutation Taster Prediction |
|---|---|---|---|---|---|---|---|---|---|---|---|---|
| | | | | | | Normal | Tumor | | | | | |
| AA1025 | chr1 | 179112145 | rs1318056 | C | G | 46.49 | 65.25 | ABL2 | S12T | MISSENSE | NON_SYNON` | P |
| AA1090 | chr1 | 179112145 | rs1318056 | C | G | 50.77 | 60.16 | ABL2 | S12T | MISSENSE | NON_SYNON` | P |
| AA943 | chr1 | 179112145 | rs1318056 | C | G | 53.16 | 45.83 | ABL2 | S12T | MISSENSE | NON_SYNON` | P |
| AA1188 | chr1 | 179112145 | rs1318056 | C | G | 52.28 | 40.18 | ABL2 | S12T | MISSENSE | NON_SYNON` | P |
| AA1222 | chr1 | 179112145 | rs1318056 | C | G | 51.74 | 44.85 | ABL2 | S12T | MISSENSE | NON_SYNON` | P |
| AA1267 | chr1 | 179112145 | rs1318056 | C | G | 49.79 | 36.8 | ABL2 | S12T | MISSENSE | NON_SYNON` | P |
| AA1307 | chr1 | 179112145 | rs1318056 | C | G | 50 | 50.89 | ABL2 | S12T | MISSENSE | NON_SYNON` | P |
| AA926 | chr1 | 179112145 | rs1318056 | C | G | 51.71 | 28.38 | ABL2 | S12T | MISSENSE | NON_SYNON` | P |
| UCI1821517 | chr1 | 179112145 | rs1318056 | C | G | 45.1 | 81.14 | ABL2 | S12T | MISSENSE | NON_SYNON` | P |
| UCI2008866 | chr1 | 179112145 | rs1318056 | C | G | 51.71 | 54.03 | ABL2 | S12T | MISSENSE | NON_SYNON` | P |
| UCI2076630 | chr1 | 179112145 | rs1318056 | C | G | 43.15 | 69.14 | ABL2 | S12T | MISSENSE | NON_SYNON` | P |
| UCI2649875 | chr1 | 179112145 | rs1318056 | C | G | 45.35 | 17.04 | ABL2 | S12T | MISSENSE | NON_SYNON` | P |
| UCI3564897 | chr1 | 179112145 | rs1318056 | C | G | 53.56 | 10.37 | ABL2 | S12T | MISSENSE | NON_SYNON` | P |
| UCI2231576 | chr14 | 105238734 | rs146483593 | C | T | 43.51 | 55.37 | AKT1 | G105S | MISSENSE | NON_SYNON` | D |
| UCI3564897 | chr7 | 117188715 | rs3034796 | GAAAGC | G | 15.06 | 14.83 | CFTR | NA | NONE | FRAME_SHIF` | NA |
| AA1025 | chr2 | 29416366 | rs1881421 | G | C | 52.74 | 47.01 | ALK | D1529E | MISSENSE | NON_SYNON` | P |
| AA1025 | chr2 | 29416481 | rs1881420 | T | C | 47.17 | 48.57 | ALK | K1491R | MISSENSE | NON_SYNON` | P |
| AA1025 | chr2 | 29416572 | rs1670283 | T | C | 99.83 | 99.85 | ALK | I1461V | MISSENSE | NON_SYNON` | N |
| AA1077 | chr2 | 29416366 | rs1881421 | G | C | 43.57 | 54.35 | ALK | D1529E | MISSENSE | NON_SYNON` | P |
| AA1077 | chr2 | 29416481 | rs1881420 | T | C | 50.09 | 52.43 | ALK | K1491R | MISSENSE | NON_SYNON` | P |
| AA1090 | chr2 | 29416572 | rs1670283 | T | C | 100 | 99.93 | ALK | I1461V | MISSENSE | NON_SYNON` | N |
| AA943 | chr2 | 29416572 | rs1670283 | T | C | 99.93 | 99.94 | ALK | I1461V | MISSENSE | NON_SYNON` | N |
| AA1188 | chr2 | 29416572 | rs1670283 | T | C | 99.95 | 99.93 | ALK | I1461V | MISSENSE | NON_SYNON` | N |
| AA1204 | chr2 | 29416572 | rs1670283 | T | C | 99.96 | 99.73 | ALK | I1461V | MISSENSE | NON_SYNON` | N |
| AA1222 | chr2 | 29416572 | rs1670283 | T | C | 99.81 | 99.88 | ALK | I1461V | MISSENSE | NON_SYNON` | N |
| AA1247 | chr2 | 29416572 | rs1670283 | T | C | 100 | 99.9 | ALK | I1461V | MISSENSE | NON_SYNON` | N |
| AA1267 | chr2 | 29416366 | rs1881421 | G | C | 46.95 | 44.18 | ALK | D1529E | MISSENSE | NON_SYNON` | P |
| AA1267 | chr2 | 29416481 | rs1881420 | T | C | 46.23 | 52.19 | ALK | K1491R | MISSENSE | NON_SYNON` | P |
| AA1267 | chr2 | 29416572 | rs1670283 | T | C | 99.93 | 99.87 | ALK | I1461V | MISSENSE | NON_SYNON` | N |
| AA1277 | chr2 | 29416366 | rs1881421 | G | C | 100 | 99.79 | ALK | D1529E | MISSENSE | NON_SYNON` | P |
| AA1277 | chr2 | 29416481 | rs1881420 | T | C | 100 | 99.6 | ALK | K1491R | MISSENSE | NON_SYNON` | P |
| AA1277 | chr2 | 29416572 | rs1670283 | T | C | 99.83 | 99.78 | ALK | I1461V | MISSENSE | NON_SYNON` | N |
| AA1307 | chr2 | 29416572 | rs1670283 | T | C | 99.87 | 99.87 | ALK | I1461V | MISSENSE | NON_SYNON` | N |
| AA957 | chr2 | 29416366 | rs1881421 | G | C | 99.75 | 99.71 | ALK | D1529E | MISSENSE | NON_SYNON` | P |
| AA957 | chr2 | 29416481 | rs1881420 | T | C | 46.98 | 47.03 | ALK | K1491R | MISSENSE | NON_SYNON` | P |
| AA957 | chr2 | 29416572 | rs1670283 | T | C | 99.71 | 99.74 | ALK | I1461V | MISSENSE | NON_SYNON` | N |
| AA926 | chr2 | 29416366 | rs1881421 | G | C | 99.93 | 99.83 | ALK | D1529E | MISSENSE | NON_SYNON` | P |
| AA926 | chr2 | 29416481 | rs1881420 | T | C | 99.56 | 99.68 | ALK | K1491R | MISSENSE | NON_SYNON` | P |
| AA926 | chr2 | 29416572 | rs1670283 | T | C | 99.82 | 99.93 | ALK | I1461V | MISSENSE | NON_SYNON` | N |
| AA930 | chr2 | 29416366 | rs1881421 | G | C | 99.7 | 99.88 | ALK | D1529E | MISSENSE | NON_SYNON` | P |
| AA930 | chr2 | 29416481 | rs1881420 | T | C | 99.33 | 99.22 | ALK | K1491R | MISSENSE | NON_SYNON` | P |
| AA930 | chr2 | 29416572 | rs1670283 | T | C | 99.62 | 99.91 | ALK | I1461V | MISSENSE | NON_SYNON` | N |
| AA1106 | chr2 | 29416366 | rs1881421 | G | C | 53.4 | 42.74 | ALK | D1529E | MISSENSE | NON_SYNON` | P |
| AA1106 | chr2 | 29416481 | rs1881420 | T | C | 46.55 | 37.58 | ALK | K1491R | MISSENSE | NON_SYNON` | P |
| AA1106 | chr2 | 29416572 | rs1670283 | T | C | 99.89 | 99.95 | ALK | I1461V | MISSENSE | NON_SYNON` | N |
| AA948 | chr2 | 29416366 | rs1881421 | G | C | 50.86 | 52.3 | ALK | D1529E | MISSENSE | NON_SYNON` | P |
| AA948 | chr2 | 29416481 | rs1881420 | T | C | 58.7 | 48.91 | ALK | K1491R | MISSENSE | NON_SYNON` | P |
| AA948 | chr2 | 29416572 | rs1670283 | T | C | 100 | 100 | ALK | I1461V | MISSENSE | NON_SYNON` | N |
| UCI8965412 | chr2 | 29474026 | rs147858673 | C | T | 55.52 | 52.9 | ALK | E717K | MISSENSE | NON_SYNON` | D |
| AA952 | chr2 | 29416366 | rs1881421 | G | C | 51.83 | 49.14 | ALK | D1529E | MISSENSE | NON_SYNON` | P |
| AA952 | chr2 | 29416572 | rs1670283 | T | C | 100 | 99.91 | ALK | I1461V | MISSENSE | NON_SYNON` | N |
| AA1367 | chr2 | 29416366 | rs1881421 | G | C | 49.42 | 50.85 | ALK | D1529E | MISSENSE | NON_SYNON` | P |
| AA1367 | chr2 | 29416481 | rs1881420 | T | C | 51.07 | 49.16 | ALK | K1491R | MISSENSE | NON_SYNON` | P |
| AA1367 | chr2 | 29416572 | rs1670283 | T | C | 99.89 | 99.92 | ALK | I1461V | MISSENSE | NON_SYNON` | N |
| AA960 | chr2 | 29416366 | rs1881421 | G | C | 48.64 | 63.2 | ALK | D1529E | MISSENSE | NON_SYNON` | P |
| AA960 | chr2 | 29416481 | rs1881420 | T | C | 46.98 | 58.39 | ALK | K1491R | MISSENSE | NON_SYNON` | P |
| AA960 | chr2 | 29416572 | rs1670283 | T | C | 100 | 99.68 | ALK | I1461V | MISSENSE | NON_SYNON` | N |
| AA1515 | chr2 | 29416366 | rs1881421 | G | C | 51.74 | 49.39 | ALK | D1529E | MISSENSE | NON_SYNON` | P |
| AA1515 | chr2 | 29416481 | rs1881420 | T | C | 46.98 | 50.02 | ALK | K1491R | MISSENSE | NON_SYNON` | P |
| AA1515 | chr2 | 29416572 | rs1670283 | T | C | 99.86 | 99.96 | ALK | I1461V | MISSENSE | NON_SYNON` | N |
| UCI1546879 | chr2 | 29416572 | rs1670283 | T | C | 100 | 100 | ALK | I1461V | MISSENSE | NON_SYNON` | N |
| UCI1689380 | chr2 | 29416366 | rs1881421 | G | C | 55.72 | 58.02 | ALK | D1529E | MISSENSE | NON_SYNON` | P |
| UCI1689380 | chr2 | 29416481 | rs1881420 | T | C | 43.3 | 63.5 | ALK | K1491R | MISSENSE | NON_SYNON` | P |
| UCI1689380 | chr2 | 29416572 | rs1670283 | T | C | 99.53 | 100 | ALK | I1461V | MISSENSE | NON_SYNON` | N |
| UCI8965412 | chr2 | 29446394 | . | A | G | 50.42 | 49.28 | ALK | V1058A | MISSENSE | NON_SYNON` | D |
| UCI1804937 | chr2 | 29416572 | rs1670283 | T | C | 99.38 | 99.55 | ALK | I1461V | MISSENSE | NON_SYNON` | N |
| UCI1821517 | chr7 | 117188715 | rs3034796 | GAAAGC | G | 15.18 | 11.65 | CFTR | NA | NONE | FRAME_SHIF` | NA |
| UCI1821517 | chr2 | 29416572 | rs1670283 | T | C | 99.34 | 99.55 | ALK | I1461V | MISSENSE | NON_SYNON` | N |
| AA1204 | chr2 | 29497967 | rs35228363 | G | A | 50.58 | 47.05 | ALK | T680I | MISSENSE | NON_SYNON` | D |
| AA930 | chr22 | 42524820 | rs3831704 | T | TC | 15.26 | 12.19 | CYP2D6 | NA | NONE | FRAME_SHIF` | NA |
| UCI1908503 | chr2 | 29416366 | rs1881421 | G | C | 44.43 | 45.69 | ALK | D1529E | MISSENSE | NON_SYNON` | P |
| UCI1908503 | chr2 | 29416481 | rs1881420 | T | C | 40.82 | 49.39 | ALK | K1491R | MISSENSE | NON_SYNON` | P |
| UCI1908503 | chr2 | 29416572 | rs1670283 | T | C | 99.18 | 99.94 | ALK | I1461V | MISSENSE | NON_SYNON` | N |
| UCI2649875 | chr2 | 29497967 | rs35228363 | G | A | 56.91 | 46.5 | ALK | T680I | MISSENSE | NON_SYNON` | D |
| UCI1951813 | chr2 | 29416366 | rs1881421 | G | C | 48.01 | 49.55 | ALK | D1529E | MISSENSE | NON_SYNON` | P |
| UCI1951813 | chr2 | 29416481 | rs1881420 | T | C | 44.92 | 51.16 | ALK | K1491R | MISSENSE | NON_SYNON` | P |
| UCI1951813 | chr2 | 29416572 | rs1670283 | T | C | 99.32 | 99.28 | ALK | I1461V | MISSENSE | NON_SYNON` | N |
| UCI2008866 | chr2 | 29416572 | rs1670283 | T | C | 100 | 99.76 | ALK | I1461V | MISSENSE | NON_SYNON` | N |
| UCI2076630 | chr2 | 29416572 | rs1670283 | T | C | 100 | 99.47 | ALK | I1461V | MISSENSE | NON_SYNON` | N |
| UCI2076630 | chr2 | 29543736 | rs35093491 | A | G | 47.82 | 60.98 | ALK | V476A | MISSENSE | NON_SYNON` | N |
| UCI2219330 | chr2 | 29416572 | rs1670283 | T | C | 45.72 | 47.34 | ALK | I1461V | MISSENSE | NON_SYNON` | N |
| UCI8965412 | chr7 | 117188715 | rs3034796 | GAAAGC | G | 15.27 | 11.71 | CFTR | NA | NONE | FRAME_SHIF` | NA |
| UCI2224680 | chr2 | 29416366 | rs1881421 | G | C | 99.67 | 99.54 | ALK | D1529E | MISSENSE | NON_SYNON` | P |
| UCI2224680 | chr2 | 29416481 | rs1881420 | T | C | 99.89 | 99.66 | ALK | K1491R | MISSENSE | NON_SYNON` | P |
| UCI2224680 | chr2 | 29416572 | rs1670283 | T | C | 99.7 | 99.96 | ALK | I1461V | MISSENSE | NON_SYNON` | N |
| UCI1951813 | chr8 | 38277052 | . | GT | G | 15.36 | 10.42 | FGFR1 | NA | NONE | FRAME_SHIF` | NA |
| UCI2231576 | chr2 | 29416366 | rs1881421 | G | C | 99.37 | 99.85 | ALK | D1529E | MISSENSE | NON_SYNON` | P |
| UCI2231576 | chr2 | 29416481 | rs1881420 | T | C | 47.35 | 50.13 | ALK | K1491R | MISSENSE | NON_SYNON` | P |
| UCI2231576 | chr2 | 29416572 | rs1670283 | T | C | 100 | 99.57 | ALK | I1461V | MISSENSE | NON_SYNON` | N |
| UCI1546879 | chr2 | 29497967 | rs35228363 | G | A | 43.74 | 36.75 | ALK | T680I | MISSENSE | NON_SYNON` | D |
| UCI2564879 | chr2 | 29416366 | rs1881421 | G | C | 53.41 | 64.06 | ALK | D1529E | MISSENSE | NON_SYNON` | P |
| UCI2564879 | chr2 | 29416481 | rs1881420 | T | C | 48.33 | 60.73 | ALK | K1491R | MISSENSE | NON_SYNON` | P |
| UCI2564879 | chr2 | 29416572 | rs1670283 | T | C | 100 | 100 | ALK | I1461V | MISSENSE | NON_SYNON` | N |
| UCI2649875 | chr2 | 29416366 | rs1881421 | G | C | 99.15 | 98.8 | ALK | D1529E | MISSENSE | NON_SYNON` | P |
| UCI2649875 | chr2 | 29416481 | rs1881420 | T | C | 98.91 | 99.56 | ALK | K1491R | MISSENSE | NON_SYNON` | P |
| UCI2649875 | chr2 | 29416572 | rs1670283 | T | C | 99.79 | 99.24 | ALK | I1461V | MISSENSE | NON_SYNON` | N |
| UCI3165498 | chr2 | 29416366 | rs1881421 | G | C | 99.76 | 99.86 | ALK | D1529E | MISSENSE | NON_SYNON` | P |
| UCI3165498 | chr2 | 29416481 | rs1881420 | T | C | 99.81 | 99.55 | ALK | K1491R | MISSENSE | NON_SYNON` | P |
| UCI3165498 | chr2 | 29416572 | rs1670283 | T | C | 99.96 | 99.69 | ALK | I1461V | MISSENSE | NON_SYNON` | N |
| UCI3564897 | chr2 | 29416572 | rs1670283 | T | C | 99.88 | 99.95 | ALK | I1461V | MISSENSE | NON_SYNON` | N |

**Table S8: List of non-silent germline variants**

| Sample | Chr | Cord | rsID | Ref Allele | Alt Allele | Allelic Fraction | | Gene | AA change | Type | Effect | Mutation Taster Prediction |
|---|---|---|---|---|---|---|---|---|---|---|---|---|
| | | | | | | Normal | Tumor | | | | | |
| UCI4216548 | chr2 | 29416572 | rs1670283 | T | C | 99.82 | 99.21 | ALK | I1461V | MISSENSE | NON_SYNON | N |
| UCI8965412 | chr2 | 29416366 | rs1881421 | G | C | 46.47 | 46.63 | ALK | D1529E | MISSENSE | NON_SYNON | P |
| UCI8965412 | chr2 | 29416481 | rs1881420 | T | C | 52.43 | 50.36 | ALK | K1491R | MISSENSE | NON_SYNON | P |
| UCI8965412 | chr2 | 29416572 | rs1670283 | T | C | 99.55 | 100 | ALK | I1461V | MISSENSE | NON_SYNON | N |
| UCI9135402 | chr9 | 139407486 | . | CA | C | 15.38 | 15 | NOTCH1 | NA | NONE | FRAME_SHIF | NA |
| UCI9135402 | chr2 | 29416366 | rs1881421 | G | C | 99.17 | 98.98 | ALK | D1529E | MISSENSE | NON_SYNON | P |
| UCI9135402 | chr2 | 29416572 | rs1670283 | T | C | 99.63 | 99.44 | ALK | I1461V | MISSENSE | NON_SYNON | N |
| AA1025 | chr5 | 112176756 | rs459552 | T | A | 52.65 | 70.77 | APC | V1822D | MISSENSE | NON_SYNON | P |
| AA1077 | chr5 | 112176756 | rs459552 | T | A | 99.85 | 99.65 | APC | V1822D | MISSENSE | NON_SYNON | P |
| AA1090 | chr5 | 112176756 | rs459552 | T | A | 54.3 | 39.75 | APC | V1822D | MISSENSE | NON_SYNON | P |
| AA943 | chr5 | 112176756 | rs459552 | T | A | 99.31 | 99.61 | APC | V1822D | MISSENSE | NON_SYNON | P |
| AA1188 | chr5 | 112176756 | rs459552 | T | A | 99.72 | 99.73 | APC | V1822D | MISSENSE | NON_SYNON | P |
| AA1204 | chr5 | 112176756 | rs459552 | T | A | 99.48 | 99.48 | APC | V1822D | MISSENSE | NON_SYNON | P |
| AA1222 | chr5 | 112176756 | rs459552 | T | A | 99.68 | 100 | APC | V1822D | MISSENSE | NON_SYNON | P |
| AA1247 | chr5 | 112176756 | rs459552 | T | A | 99.78 | 99.78 | APC | V1822D | MISSENSE | NON_SYNON | P |
| AA1247 | chr5 | 112178795 | rs2229995 | G | A | 48.9 | 37.51 | APC | G2502S | MISSENSE | NON_SYNON | N |
| AA1267 | chr5 | 112176756 | rs459552 | T | A | 99.69 | 99.71 | APC | V1822D | MISSENSE | NON_SYNON | P |
| AA1277 | chr5 | 112176756 | rs459552 | T | A | 58.49 | 9.98 | APC | V1822D | MISSENSE | NON_SYNON | P |
| AA1307 | chr5 | 112176756 | rs459552 | T | A | 54.23 | 71.24 | APC | V1822D | MISSENSE | NON_SYNON | P |
| AA957 | chr5 | 112176756 | rs459552 | T | A | 45.91 | 53.97 | APC | V1822D | MISSENSE | NON_SYNON | P |
| AA926 | chr5 | 112176756 | rs459552 | T | A | 99.81 | 99.7 | APC | V1822D | MISSENSE | NON_SYNON | P |
| AA930 | chr5 | 112176756 | rs459552 | T | A | 98.49 | 99.91 | APC | V1822D | MISSENSE | NON_SYNON | P |
| AA1106 | chr5 | 112176756 | rs459552 | T | A | 99.35 | 99.19 | APC | V1822D | MISSENSE | NON_SYNON | P |
| AA948 | chr5 | 112176756 | rs459552 | T | A | 98.73 | 99.51 | APC | V1822D | MISSENSE | NON_SYNON | P |
| AA952 | chr5 | 112176756 | rs459552 | T | A | 99.85 | 100 | APC | V1822D | MISSENSE | NON_SYNON | P |
| AA952 | chr5 | 112178795 | rs2229995 | G | A | 52.77 | 49.44 | APC | G2502S | MISSENSE | NON_SYNON | N |
| AA1367 | chr5 | 112176756 | rs459552 | T | A | 52.33 | 27.44 | APC | V1822D | MISSENSE | NON_SYNON | P |
| AA960 | chr5 | 112176756 | rs459552 | T | A | 100 | 99.76 | APC | V1822D | MISSENSE | NON_SYNON | P |
| AA1515 | chr5 | 112176756 | rs459552 | T | A | 99.73 | 99.92 | APC | V1822D | MISSENSE | NON_SYNON | P |
| UCI1546879 | chr5 | 112176756 | rs459552 | T | A | 99.88 | 99.75 | APC | V1822D | MISSENSE | NON_SYNON | P |
| UCI1689380 | chr5 | 112102960 | rs139196838 | C | T | 49.52 | 92.48 | APC | R109W | MISSENSE | NON_SYNON | N |
| UCI1689380 | chr5 | 112176756 | rs459552 | T | A | 52.28 | 10.79 | APC | V1822D | MISSENSE | NON_SYNON | P |
| UCI1804937 | chr5 | 112176756 | rs459552 | T | A | 98.42 | 97.89 | APC | V1822D | MISSENSE | NON_SYNON | P |
| UCI1821517 | chr5 | 112176756 | rs459552 | T | A | 98.9 | 99.62 | APC | V1822D | MISSENSE | NON_SYNON | P |
| UCI1908503 | chr5 | 112176756 | rs459552 | T | A | 99.86 | 99.22 | APC | V1822D | MISSENSE | NON_SYNON | P |
| UCI1951813 | chr5 | 112176756 | rs459552 | T | A | 96.98 | 97.64 | APC | V1822D | MISSENSE | NON_SYNON | P |
| UCI2008866 | chr5 | 112176756 | rs459552 | T | A | 100 | 99.74 | APC | V1822D | MISSENSE | NON_SYNON | P |
| UCI2076630 | chr5 | 112176756 | rs459552 | T | A | 52.85 | 46.38 | APC | V1822D | MISSENSE | NON_SYNON | P |
| UCI2219330 | chr5 | 112176756 | rs459552 | T | A | 41.36 | 46.76 | APC | V1822D | MISSENSE | NON_SYNON | P |
| UCI2224680 | chr5 | 112176756 | rs459552 | T | A | 99.51 | 99.65 | APC | V1822D | MISSENSE | NON_SYNON | P |
| UCI2231576 | chr5 | 112176756 | rs459552 | T | A | 98.73 | 100 | APC | V1822D | MISSENSE | NON_SYNON | P |
| UCI2564879 | chr5 | 112176756 | rs459552 | T | A | 99.24 | 100 | APC | V1822D | MISSENSE | NON_SYNON | P |
| UCI2649875 | chr5 | 112176756 | rs459552 | T | A | 48.63 | 48.27 | APC | V1822D | MISSENSE | NON_SYNON | P |
| UCI3165498 | chr5 | 112176756 | rs459552 | T | A | 99.7 | 98.89 | APC | V1822D | MISSENSE | NON_SYNON | P |
| UCI3165498 | chr5 | 112178795 | rs2229995 | G | A | 45.24 | 19.29 | APC | G2502S | MISSENSE | NON_SYNON | N |
| UCI3564897 | chr5 | 112176756 | rs459552 | T | A | 99.62 | 99.79 | APC | V1822D | MISSENSE | NON_SYNON | P |
| UCI4216548 | chr5 | 112176756 | rs459552 | T | A | 100 | 99.71 | APC | V1822D | MISSENSE | NON_SYNON | P |
| UCI8965412 | chr5 | 112176756 | rs459552 | T | A | 60.31 | 56.37 | APC | V1822D | MISSENSE | NON_SYNON | P |
| UCI9135402 | chr5 | 112176756 | rs459552 | T | A | 98.97 | 98.9 | APC | V1822D | MISSENSE | NON_SYNON | P |
| AA1025 | chr11 | 108183167 | rs659243 | A | G | 100 | 99.92 | ATM | N1983S | MISSENSE | NON_SYNON | N |
| AA1077 | chr11 | 108106443 | rs2234997 | T | A | 48.8 | 17.8 | ATM | D126E | MISSENSE | NON_SYNON | N |
| AA1077 | chr11 | 108175462 | rs1801516 | G | A | 55.74 | 81.53 | ATM | D1853N | MISSENSE | NON_SYNON | P |
| AA1077 | chr11 | 108183167 | rs659243 | A | G | 99.89 | 99.76 | ATM | N1983S | MISSENSE | NON_SYNON | N |
| AA1090 | chr11 | 108175462 | rs1801516 | G | A | 99.7 | 99.69 | ATM | D1853N | MISSENSE | NON_SYNON | P |
| AA1090 | chr11 | 108183167 | rs659243 | A | G | 99.89 | 99.6 | ATM | N1983S | MISSENSE | NON_SYNON | N |
| AA943 | chr11 | 108183167 | rs659243 | A | G | 99.74 | 99.79 | ATM | N1983S | MISSENSE | NON_SYNON | N |
| AA1188 | chr11 | 108183167 | rs659243 | A | G | 100 | 100 | ATM | N1983S | MISSENSE | NON_SYNON | N |
| AA1204 | chr11 | 108183167 | rs659243 | A | G | 99.88 | 99.89 | ATM | N1983S | MISSENSE | NON_SYNON | N |
| AA1222 | chr11 | 108183167 | rs659243 | A | G | 99.92 | 99.93 | ATM | N1983S | MISSENSE | NON_SYNON | N |
| AA1247 | chr11 | 108183167 | rs659243 | A | G | 99.89 | 100 | ATM | N1983S | MISSENSE | NON_SYNON | N |
| AA1267 | chr11 | 108183167 | rs659243 | A | G | 99.61 | 100 | ATM | N1983S | MISSENSE | NON_SYNON | N |
| AA1277 | chr11 | 108183167 | rs659243 | A | G | 99.64 | 99.63 | ATM | N1983S | MISSENSE | NON_SYNON | N |
| AA1307 | chr11 | 108183167 | rs659243 | A | G | 100 | 99.83 | ATM | N1983S | MISSENSE | NON_SYNON | N |
| AA957 | chr11 | 108183167 | rs659243 | A | G | 99.8 | 99.68 | ATM | N1983S | MISSENSE | NON_SYNON | N |
| AA926 | chr11 | 108175462 | rs1801516 | G | A | 50.13 | 44.73 | ATM | D1853N | MISSENSE | NON_SYNON | P |
| AA926 | chr11 | 108183167 | rs659243 | A | G | 99.81 | 99.76 | ATM | N1983S | MISSENSE | NON_SYNON | N |
| AA930 | chr11 | 108183167 | rs659243 | A | G | 100 | 99.95 | ATM | N1983S | MISSENSE | NON_SYNON | N |
| AA1106 | chr11 | 108175462 | rs1801516 | G | A | 40.38 | 22.21 | ATM | D1853N | MISSENSE | NON_SYNON | P |
| AA1106 | chr11 | 108183167 | rs659243 | A | G | 99.72 | 99.74 | ATM | N1983S | MISSENSE | NON_SYNON | N |
| AA948 | chr11 | 108175462 | rs1801516 | G | A | 52.64 | 47.82 | ATM | D1853N | MISSENSE | NON_SYNON | P |
| AA948 | chr11 | 108183167 | rs659243 | A | G | 100 | 100 | ATM | N1983S | MISSENSE | NON_SYNON | N |
| AA952 | chr11 | 108183167 | rs659243 | A | G | 99.81 | 99.93 | ATM | N1983S | MISSENSE | NON_SYNON | N |
| AA1367 | chr11 | 108168053 | rs55870064 | A | G | 49.69 | 55.14 | ATM | N1650S | MISSENSE | NON_SYNON | N |
| AA1367 | chr11 | 108183167 | rs659243 | A | G | 100 | 99.57 | ATM | N1983S | MISSENSE | NON_SYNON | N |
| AA960 | chr11 | 108183167 | rs659243 | A | G | 99.6 | 99.74 | ATM | N1983S | MISSENSE | NON_SYNON | N |
| AA1515 | chr11 | 108175462 | rs1801516 | G | A | 52.22 | 45.24 | ATM | D1853N | MISSENSE | NON_SYNON | P |
| AA1515 | chr11 | 108183167 | rs659243 | A | G | 99.89 | 100 | ATM | N1983S | MISSENSE | NON_SYNON | N |
| UCI1546879 | chr11 | 108183167 | rs659243 | A | G | 100 | 99.71 | ATM | N1983S | MISSENSE | NON_SYNON | N |
| UCI1689380 | chr11 | 108183167 | rs659243 | A | G | 99.77 | 99.93 | ATM | N1983S | MISSENSE | NON_SYNON | N |
| AA930 | chr11 | 108098576 | rs1800054 | C | G | 42.89 | 48.56 | ATM | S49C | MISSENSE | NON_SYNON | D |
| UCI1804937 | chr11 | 108183167 | rs659243 | A | G | 96.07 | 93.39 | ATM | N1983S | MISSENSE | NON_SYNON | N |
| UCI1821517 | chr11 | 108183167 | rs659243 | A | G | 98.41 | 98.02 | ATM | N1983S | MISSENSE | NON_SYNON | N |
| UCI1908503 | chr11 | 108183167 | rs659243 | A | G | 98.69 | 98.03 | ATM | N1983S | MISSENSE | NON_SYNON | N |
| UCI1951813 | chr11 | 108183167 | rs659243 | A | G | 95.41 | 93.59 | ATM | N1983S | MISSENSE | NON_SYNON | N |
| UCI2008866 | chr11 | 108175462 | rs1801516 | G | A | 47.07 | 63.17 | ATM | D1853N | MISSENSE | NON_SYNON | P |
| UCI2008866 | chr11 | 108183167 | rs659243 | A | G | 99.81 | 100 | ATM | N1983S | MISSENSE | NON_SYNON | N |
| UCI2076630 | chr11 | 108183167 | rs659243 | A | G | 100 | 99.88 | ATM | N1983S | MISSENSE | NON_SYNON | N |
| UCI2219330 | chr11 | 108175462 | rs1801516 | G | A | 48.28 | 47.62 | ATM | D1853N | MISSENSE | NON_SYNON | P |
| UCI2219330 | chr11 | 108183167 | rs659243 | A | G | 99.71 | 99.8 | ATM | N1983S | MISSENSE | NON_SYNON | N |
| UCI2219330 | chr11 | 108196896 | rs56009889 | C | T | 54.18 | 48.72 | ATM | L2307F | MISSENSE | NON_SYNON | N |
| UCI2224680 | chr11 | 108183167 | rs659243 | A | G | 100 | 99.94 | ATM | N1983S | MISSENSE | NON_SYNON | N |
| UCI2231576 | chr11 | 108119823 | rs56128736 | T | C | 56.88 | 45.68 | ATM | V410A | MISSENSE | NON_SYNON | N |
| UCI2231576 | chr11 | 108183167 | rs659243 | A | G | 100 | 100 | ATM | N1983S | MISSENSE | NON_SYNON | N |
| UCI2564879 | chr11 | 108183167 | rs659243 | A | G | 99.8 | 99.91 | ATM | N1983S | MISSENSE | NON_SYNON | N |
| UCI2649875 | chr11 | 108183167 | rs659243 | A | G | 97.93 | 97.95 | ATM | N1983S | MISSENSE | NON_SYNON | N |
| UCI3165498 | chr11 | 108183167 | rs659243 | A | G | 99.78 | 99.72 | ATM | N1983S | MISSENSE | NON_SYNON | N |
| UCI3564897 | chr11 | 108175462 | rs1801516 | G | A | 54.1 | 12.9 | ATM | D1853N | MISSENSE | NON_SYNON | P |
| UCI3564897 | chr11 | 108183167 | rs659243 | A | G | 99.82 | 99.83 | ATM | N1983S | MISSENSE | NON_SYNON | N |
| UCI4216548 | chr11 | 108183167 | rs659243 | A | G | 99.82 | 99.92 | ATM | N1983S | MISSENSE | NON_SYNON | N |
| UCI8965412 | chr11 | 108183167 | rs659243 | A | G | 99.92 | 100 | ATM | N1983S | MISSENSE | NON_SYNON | N |

**Table S8: List of non-silent germline variants**

| Sample | Chr | Cord | rsID | Ref Allele | Alt Allele | Allelic Fraction | | Gene | AA change | Type | Effect | Mutation Taster Prediction |
|---|---|---|---|---|---|---|---|---|---|---|---|---|
| | | | | | | Normal | Tumor | | | | | |
| AA1277 | chr11 | 108235879 | rs139379666 | C | T | 49.94 | 11.35 | ATM | P2974L | MISSENSE | NON_SYNON` | D |
| UCI9135402 | chr11 | 108175462 | rs1801516 | G | A | 99.25 | 98.82 | ATM | D1853N | MISSENSE | NON_SYNON` | P |
| UCI9135402 | chr11 | 108183167 | rs659243 | A | G | 98.66 | 98.15 | ATM | N1983S | MISSENSE | NON_SYNON` | N |
| AA957 | chr7 | 117188715 | rs3034796 | GAAAGC | G | 15.61 | 12.61 | CFTR | NA | NONE | FRAME_SHIF` | NA |
| AA943 | chr14 | 105243085 | . | CT | C | 16.04 | 16.27 | AKT1 | NA | NONE | FRAME_SHIF` | NA |
| AA943 | chr17 | 41223094 | rs1799966 | T | C | 99.87 | 98.98 | BRCA1 | S1317G | MISSENSE | NON_SYNON` | P |
| AA943 | chr17 | 41244000 | rs16942 | T | C | 100 | 98.73 | BRCA1 | K1136R | MISSENSE | NON_SYNON` | P |
| AA943 | chr17 | 41244435 | rs16941 | T | C | 99.75 | 98.13 | BRCA1 | E1038G | MISSENSE | NON_SYNON` | P |
| AA943 | chr17 | 41244936 | rs799917 | G | A | 99.85 | 98.81 | BRCA1 | P575L | MISSENSE | NON_SYNON` | P |
| AA943 | chr17 | 41245471 | rs4986850 | C | T | 52.67 | 51.88 | BRCA1 | D397N | MISSENSE | NON_SYNON` | P |
| AA1188 | chr17 | 41243840 | rs28897687 | A | C | 47.55 | 48.74 | BRCA1 | N1189K | MISSENSE | NON_SYNON` | N |
| UCI1804937 | chr2 | 29451760 | . | TCC | T | 16.25 | 13.62 | ALK | NA | NONE | FRAME_SHIF` | NA |
| AA1204 | chr17 | 41223094 | rs1799966 | T | C | 53.4 | 27.2 | BRCA1 | S1317G | MISSENSE | NON_SYNON` | P |
| AA1204 | chr17 | 41244000 | rs16942 | T | C | 54.36 | 22.04 | BRCA1 | K1136R | MISSENSE | NON_SYNON` | P |
| AA1204 | chr17 | 41244435 | rs16941 | T | C | 46.83 | 27.07 | BRCA1 | E1038G | MISSENSE | NON_SYNON` | P |
| AA1204 | chr17 | 41244936 | rs799917 | G | A | 52.24 | 28.2 | BRCA1 | P575L | MISSENSE | NON_SYNON` | P |
| AA1222 | chr17 | 41223094 | rs1799966 | T | C | 49.16 | 70.16 | BRCA1 | S1317G | MISSENSE | NON_SYNON` | P |
| AA1222 | chr17 | 41244000 | rs16942 | T | C | 60.9 | 67.99 | BRCA1 | K1136R | MISSENSE | NON_SYNON` | P |
| AA1222 | chr17 | 41244435 | rs16941 | T | C | 55.42 | 66.88 | BRCA1 | E1038G | MISSENSE | NON_SYNON` | P |
| AA1222 | chr17 | 41244936 | rs799917 | G | A | 48.32 | 71.71 | BRCA1 | P575L | MISSENSE | NON_SYNON` | P |
| AA1247 | chr17 | 41223094 | rs1799966 | T | C | 49.28 | 17.48 | BRCA1 | S1317G | MISSENSE | NON_SYNON` | P |
| AA1247 | chr17 | 41244000 | rs16942 | T | C | 46.76 | 15.93 | BRCA1 | K1136R | MISSENSE | NON_SYNON` | P |
| AA1247 | chr17 | 41244435 | rs16941 | T | C | 48.71 | 18.46 | BRCA1 | E1038G | MISSENSE | NON_SYNON` | P |
| AA1247 | chr17 | 41244936 | rs799917 | G | A | 44.73 | 17.29 | BRCA1 | P575L | MISSENSE | NON_SYNON` | P |
| AA1267 | chr17 | 41223094 | rs1799966 | T | C | 48.75 | 50.75 | BRCA1 | S1317G | MISSENSE | NON_SYNON` | P |
| AA1267 | chr17 | 41244000 | rs16942 | T | C | 50.03 | 52.61 | BRCA1 | K1136R | MISSENSE | NON_SYNON` | P |
| AA1267 | chr17 | 41244435 | rs16941 | T | C | 52.95 | 50.17 | BRCA1 | E1038G | MISSENSE | NON_SYNON` | P |
| AA1267 | chr17 | 41244936 | rs799917 | G | A | 48.17 | 53.43 | BRCA1 | P575L | MISSENSE | NON_SYNON` | P |
| AA957 | chr17 | 41223094 | rs1799966 | T | C | 51.21 | 51.27 | BRCA1 | S1317G | MISSENSE | NON_SYNON` | P |
| AA957 | chr17 | 41244000 | rs16942 | T | C | 45.22 | 47.22 | BRCA1 | K1136R | MISSENSE | NON_SYNON` | P |
| AA957 | chr17 | 41244435 | rs16941 | T | C | 43.58 | 44.31 | BRCA1 | E1038G | MISSENSE | NON_SYNON` | P |
| AA957 | chr17 | 41244936 | rs799917 | G | A | 46.99 | 51.38 | BRCA1 | P575L | MISSENSE | NON_SYNON` | P |
| AA926 | chr17 | 41244936 | rs799917 | G | A | 48.11 | 51.95 | BRCA1 | P575L | MISSENSE | NON_SYNON` | P |
| AA930 | chr17 | 41223094 | rs1799966 | T | C | 54.35 | 51.38 | BRCA1 | S1317G | MISSENSE | NON_SYNON` | P |
| AA930 | chr17 | 41244000 | rs16942 | T | C | 54.2 | 52.49 | BRCA1 | K1136R | MISSENSE | NON_SYNON` | P |
| AA930 | chr17 | 41244435 | rs16941 | T | C | 46.78 | 51.74 | BRCA1 | E1038G | MISSENSE | NON_SYNON` | P |
| AA930 | chr17 | 41244936 | rs799917 | G | A | 42.81 | 52.41 | BRCA1 | P575L | MISSENSE | NON_SYNON` | P |
| AA1106 | chr17 | 41243509 | rs28897689 | T | C | 50.79 | 48.94 | BRCA1 | R1051G | MISSENSE | NON_SYNON` | N |
| AA948 | chr17 | 41244710 | . | G | C | 47.33 | 45.26 | BRCA1 | I650M | MISSENSE | NON_SYNON` | N |
| AA952 | chr17 | 41246481 | rs1799950 | T | C | 46.18 | 49.73 | BRCA1 | Q309R | MISSENSE | NON_SYNON` | N |
| AA1515 | chr17 | 41219694 | rs80357087 | C | A | 51.47 | 52 | BRCA1 | A1373S | MISSENSE | NON_SYNON` | D |
| AA1367 | chr17 | 41244982 | rs80356892 | A | G | 45.23 | 70.69 | BRCA1 | Y560H | MISSENSE | NON_SYNON` | N |
| UCI1546879 | chr17 | 41222975 | rs1799967 | C | T | 51.31 | 60.25 | BRCA1 | M1356I | MISSENSE | NON_SYNON` | N |
| UCI1546879 | chr17 | 41223094 | rs1799966 | T | C | 99.62 | 99.47 | BRCA1 | S1317G | MISSENSE | NON_SYNON` | P |
| UCI1546879 | chr17 | 41244000 | rs16942 | T | C | 99.78 | 99.69 | BRCA1 | K1136R | MISSENSE | NON_SYNON` | P |
| UCI1546879 | chr17 | 41244435 | rs16941 | T | C | 99.56 | 99.35 | BRCA1 | E1038G | MISSENSE | NON_SYNON` | P |
| UCI1546879 | chr17 | 41244936 | rs799917 | G | A | 99.71 | 99.61 | BRCA1 | P575L | MISSENSE | NON_SYNON` | P |
| UCI1821517 | chr17 | 41223094 | rs1799966 | T | C | 98.58 | 98 | BRCA1 | S1317G | MISSENSE | NON_SYNON` | P |
| UCI1821517 | chr17 | 41244000 | rs16942 | T | C | 98.58 | 98.77 | BRCA1 | K1136R | MISSENSE | NON_SYNON` | P |
| UCI1821517 | chr17 | 41244435 | rs16941 | T | C | 99.24 | 98.88 | BRCA1 | E1038G | MISSENSE | NON_SYNON` | P |
| UCI1821517 | chr17 | 41244936 | rs799917 | G | A | 99.24 | 98.7 | BRCA1 | P575L | MISSENSE | NON_SYNON` | P |
| UCI1908503 | chr17 | 41244429 | rs4986852 | C | T | 50.63 | 46.93 | BRCA1 | S1040N | MISSENSE | NON_SYNON` | N |
| UCI2076630 | chr17 | 41223094 | rs1799966 | T | C | 99.46 | 99.35 | BRCA1 | S1317G | MISSENSE | NON_SYNON` | P |
| UCI2076630 | chr17 | 41244000 | rs16942 | T | C | 98.38 | 98.1 | BRCA1 | K1136R | MISSENSE | NON_SYNON` | P |
| UCI2076630 | chr17 | 41244435 | rs16941 | T | C | 99.6 | 98.88 | BRCA1 | E1038G | MISSENSE | NON_SYNON` | P |
| UCI2076630 | chr17 | 41244936 | rs799917 | G | A | 98.83 | 98.78 | BRCA1 | P575L | MISSENSE | NON_SYNON` | P |
| UCI2219330 | chr17 | 41246481 | rs1799950 | T | C | 49.02 | 49.12 | BRCA1 | Q309R | MISSENSE | NON_SYNON` | N |
| UCI2224680 | chr17 | 41223094 | rs1799966 | T | C | 46.06 | 74.04 | BRCA1 | S1317G | MISSENSE | NON_SYNON` | P |
| UCI2224680 | chr17 | 41244000 | rs16942 | T | C | 49.49 | 79.81 | BRCA1 | K1136R | MISSENSE | NON_SYNON` | P |
| UCI2224680 | chr17 | 41244435 | rs16941 | T | C | 47.7 | 72.07 | BRCA1 | E1038G | MISSENSE | NON_SYNON` | P |
| UCI2224680 | chr17 | 41244936 | rs799917 | G | A | 50.19 | 71.74 | BRCA1 | P575L | MISSENSE | NON_SYNON` | P |
| UCI2224680 | chr17 | 41245471 | rs4986850 | C | T | 42.86 | 73.57 | BRCA1 | D397N | MISSENSE | NON_SYNON` | P |
| UCI2231576 | chr17 | 41244252 | rs80357201 | G | A | 50.83 | 51.41 | BRCA1 | P1052L | MISSENSE | NON_SYNON` | N |
| UCI2564879 | chr17 | 41223094 | rs1799966 | T | C | 47.68 | 49.99 | BRCA1 | S1317G | MISSENSE | NON_SYNON` | P |
| UCI2564879 | chr17 | 41244000 | rs16942 | T | C | 55.6 | 53.77 | BRCA1 | K1136R | MISSENSE | NON_SYNON` | P |
| UCI2564879 | chr17 | 41244435 | rs16941 | T | C | 52.62 | 50.35 | BRCA1 | E1038G | MISSENSE | NON_SYNON` | P |
| UCI2564879 | chr17 | 41244936 | rs799917 | G | A | 49.52 | 52.51 | BRCA1 | P575L | MISSENSE | NON_SYNON` | P |
| UCI2649875 | chr17 | 41244936 | rs799917 | G | A | 49.09 | 53.03 | BRCA1 | P575L | MISSENSE | NON_SYNON` | P |
| UCI2649875 | chr17 | 41256956 | rs80357209 | G | C | 43.87 | 48.01 | BRCA1 | T30R | MISSENSE | NON_SYNON` | N |
| UCI9135402 | chr17 | 41223094 | rs1799966 | T | C | 47.08 | 50.99 | BRCA1 | S1317G | MISSENSE | NON_SYNON` | P |
| UCI9135402 | chr17 | 41244000 | rs16942 | T | C | 45.14 | 54.11 | BRCA1 | K1136R | MISSENSE | NON_SYNON` | P |
| UCI9135402 | chr17 | 41244435 | rs16941 | T | C | 53.65 | 49.5 | BRCA1 | E1038G | MISSENSE | NON_SYNON` | P |
| UCI9135402 | chr17 | 41244936 | rs799917 | G | A | 53.7 | 44.4 | BRCA1 | P575L | MISSENSE | NON_SYNON` | P |
| AA1025 | chr13 | 32914236 | rs4987117 | C | T | 56.52 | 33.93 | BRCA2 | T1915M | MISSENSE | NON_SYNON` | N |
| AA1025 | chr13 | 32929387 | rs169547 | T | C | 99.92 | 99.94 | BRCA2 | V2466A | MISSENSE | NON_SYNON` | N |
| AA1077 | chr13 | 32929387 | rs169547 | T | C | 99.86 | 100 | BRCA2 | V2466A | MISSENSE | NON_SYNON` | N |
| AA1090 | chr13 | 32912928 | rs80358678 | G | C | 51.36 | 47.54 | BRCA2 | S1479T | MISSENSE | NON_SYNON` | N |
| AA1090 | chr13 | 32929387 | rs169547 | T | C | 100 | 99.94 | BRCA2 | V2466A | MISSENSE | NON_SYNON` | N |
| AA943 | chr13 | 32929387 | rs169547 | T | C | 99.81 | 100 | BRCA2 | V2466A | MISSENSE | NON_SYNON` | N |
| AA1188 | chr13 | 32929387 | rs169547 | T | C | 99.74 | 99.66 | BRCA2 | V2466A | MISSENSE | NON_SYNON` | N |
| AA1204 | chr13 | 32929387 | rs169547 | T | C | 99.92 | 99.88 | BRCA2 | V2466A | MISSENSE | NON_SYNON` | N |
| AA1222 | chr13 | 32906729 | rs144848 | A | C | 57.57 | 80.48 | BRCA2 | N370H | MISSENSE | NON_SYNON` | P |
| AA1222 | chr13 | 32929387 | rs169547 | T | C | 99.78 | 99.48 | BRCA2 | V2466A | MISSENSE | NON_SYNON` | N |
| AA1247 | chr13 | 32929387 | rs169547 | T | C | 99.8 | 99.78 | BRCA2 | V2466A | MISSENSE | NON_SYNON` | N |
| AA1267 | chr13 | 32929387 | rs169547 | T | C | 99.81 | 100 | BRCA2 | V2466A | MISSENSE | NON_SYNON` | N |
| AA1277 | chr13 | 32910842 | rs11571653 | A | G | 45.82 | 67.44 | BRCA2 | M784V | MISSENSE | NON_SYNON` | N |
| AA1277 | chr13 | 32929387 | rs169547 | T | C | 99.35 | 100 | BRCA2 | V2466A | MISSENSE | NON_SYNON` | N |
| AA1307 | chr13 | 32929387 | rs169547 | T | C | 99.96 | 100 | BRCA2 | V2466A | MISSENSE | NON_SYNON` | N |
| AA957 | chr13 | 32929387 | rs169547 | T | C | 99.96 | 100 | BRCA2 | V2466A | MISSENSE | NON_SYNON` | N |
| AA926 | chr13 | 32914236 | rs4987117 | C | T | 50 | 62.88 | BRCA2 | T1915M | MISSENSE | NON_SYNON` | N |
| AA926 | chr13 | 32929387 | rs169547 | T | C | 100 | 100 | BRCA2 | V2466A | MISSENSE | NON_SYNON` | N |
| AA930 | chr13 | 32906729 | rs144848 | A | C | 48.09 | 52.48 | BRCA2 | N370H | MISSENSE | NON_SYNON` | P |
| AA930 | chr13 | 32929387 | rs169547 | T | C | 99.74 | 99.88 | BRCA2 | V2466A | MISSENSE | NON_SYNON` | N |
| AA1106 | chr13 | 32914592 | rs1799954 | C | T | 51.79 | 46.64 | BRCA2 | R2034C | MISSENSE | NON_SYNON` | N |
| AA1106 | chr13 | 32929387 | rs169547 | T | C | 99.6 | 99.85 | BRCA2 | V2466A | MISSENSE | NON_SYNON` | N |
| AA948 | chr13 | 32906729 | rs144848 | A | C | 46.76 | 50.25 | BRCA2 | N370H | MISSENSE | NON_SYNON` | P |
| AA948 | chr13 | 32914947 | rs80358881 | C | A | 48.15 | 46.74 | BRCA2 | S2152Y | MISSENSE | NON_SYNON` | N |
| AA948 | chr13 | 32929387 | rs169547 | T | C | 100 | 100 | BRCA2 | V2466A | MISSENSE | NON_SYNON` | N |
| AA952 | chr13 | 32906729 | rs144848 | A | C | 99.85 | 99.41 | BRCA2 | N370H | MISSENSE | NON_SYNON` | P |
| AA952 | chr13 | 32929387 | rs169547 | T | C | 99.83 | 99.87 | BRCA2 | V2466A | MISSENSE | NON_SYNON` | N |

**Table S8: List of non-silent germline variants**

| Sample | Chr | Cord | rsID | Ref Allele | Alt Allele | Allelic Fraction | | Gene | AA change | Type | Effect | Mutation Taster Prediction |
|---|---|---|---|---|---|---|---|---|---|---|---|---|
| | | | | | | Normal | Tumor | | | | | |
| AA1367 | chr13 | 32906729 | rs144848 | A | C | 50.73 | 70.94 | BRCA2 | N370H | MISSENSE | NON_SYNON | P |
| AA1367 | chr13 | 32929387 | rs169547 | T | C | 99.93 | 99.9 | BRCA2 | V2466A | MISSENSE | NON_SYNON | N |
| AA960 | chr13 | 32906729 | rs144848 | A | C | 48.67 | 49.6 | BRCA2 | N370H | MISSENSE | NON_SYNON | P |
| AA960 | chr13 | 32914236 | rs4987117 | C | T | 46.43 | 45.87 | BRCA2 | T1915M | MISSENSE | NON_SYNON | N |
| AA960 | chr13 | 32929387 | rs169547 | T | C | 99.49 | 99.86 | BRCA2 | V2466A | MISSENSE | NON_SYNON | N |
| AA960 | chr13 | 32930598 | rs11571707 | T | C | 46.28 | 53.85 | BRCA2 | I2490T | MISSENSE | NON_SYNON | N |
| AA1515 | chr13 | 32906480 | rs766173 | A | C | 59.11 | 50.69 | BRCA2 | N287H | MISSENSE | NON_SYNON | N |
| AA1515 | chr13 | 32911463 | rs1799944 | A | G | 50.89 | 53.88 | BRCA2 | N991D | MISSENSE | NON_SYNON | P |
| AA1515 | chr13 | 32929387 | rs169547 | T | C | 99.91 | 99.85 | BRCA2 | V2466A | MISSENSE | NON_SYNON | N |
| UCI1546879 | chr13 | 32906729 | rs144848 | A | C | 44.8 | 31.14 | BRCA2 | N370H | MISSENSE | NON_SYNON | P |
| UCI1546879 | chr13 | 32929387 | rs169547 | T | C | 99.58 | 100 | BRCA2 | V2466A | MISSENSE | NON_SYNON | N |
| UCI1689380 | chr13 | 32906729 | rs144848 | A | C | 41.44 | 61.95 | BRCA2 | N370H | MISSENSE | NON_SYNON | P |
| UCI1689380 | chr13 | 32929387 | rs169547 | T | C | 99.42 | 99.72 | BRCA2 | V2466A | MISSENSE | NON_SYNON | N |
| UCI1804937 | chr13 | 32929387 | rs169547 | T | C | 99.68 | 99.78 | BRCA2 | V2466A | MISSENSE | NON_SYNON | N |
| UCI1821517 | chr13 | 32929387 | rs169547 | T | C | 99.62 | 99.63 | BRCA2 | V2466A | MISSENSE | NON_SYNON | N |
| UCI1908503 | chr13 | 32929387 | rs169547 | T | C | 99.49 | 99.55 | BRCA2 | V2466A | MISSENSE | NON_SYNON | N |
| UCI1951813 | chr13 | 32906729 | rs144848 | A | C | 99.59 | 99.75 | BRCA2 | N370H | MISSENSE | NON_SYNON | P |
| UCI1951813 | chr13 | 32929387 | rs169547 | T | C | 99.77 | 99.72 | BRCA2 | V2466A | MISSENSE | NON_SYNON | N |
| UCI2008866 | chr13 | 32906729 | rs144848 | A | C | 54.32 | 10.51 | BRCA2 | N370H | MISSENSE | NON_SYNON | P |
| UCI2008866 | chr13 | 32929387 | rs169547 | T | C | 99.93 | 99.82 | BRCA2 | V2466A | MISSENSE | NON_SYNON | N |
| UCI2076630 | chr13 | 32929387 | rs169547 | T | C | 100 | 99.69 | BRCA2 | V2466A | MISSENSE | NON_SYNON | N |
| UCI2219330 | chr13 | 32906729 | rs144848 | A | C | 99.56 | 99.62 | BRCA2 | N370H | MISSENSE | NON_SYNON | P |
| UCI2219330 | chr13 | 32929387 | rs169547 | T | C | 99.79 | 99.91 | BRCA2 | V2466A | MISSENSE | NON_SYNON | N |
| UCI2224680 | chr13 | 32929387 | rs169547 | T | C | 99.76 | 99.52 | BRCA2 | V2466A | MISSENSE | NON_SYNON | N |
| UCI2224680 | chr13 | 32972884 | rs1801426 | A | G | 53.2 | 34.16 | BRCA2 | I3412V | MISSENSE | NON_SYNON | P |
| UCI2231576 | chr13 | 32906480 | rs766173 | A | C | 55.74 | 47.48 | BRCA2 | N287H | MISSENSE | NON_SYNON | N |
| UCI2231576 | chr13 | 32911463 | rs1799944 | A | G | 52.76 | 54.43 | BRCA2 | N991D | MISSENSE | NON_SYNON | P |
| UCI2231576 | chr13 | 32929387 | rs169547 | T | C | 99.65 | 99.91 | BRCA2 | V2466A | MISSENSE | NON_SYNON | N |
| UCI2564879 | chr13 | 32929387 | rs169547 | T | C | 100 | 100 | BRCA2 | V2466A | MISSENSE | NON_SYNON | N |
| UCI2649875 | chr13 | 32929387 | rs169547 | T | C | 99.31 | 99.67 | BRCA2 | V2466A | MISSENSE | NON_SYNON | N |
| UCI3165498 | chr13 | 32929387 | rs169547 | T | C | 99.88 | 99.85 | BRCA2 | V2466A | MISSENSE | NON_SYNON | N |
| UCI3564897 | chr13 | 32906480 | rs766173 | A | C | 53.57 | 53.7 | BRCA2 | N287H | MISSENSE | NON_SYNON | N |
| UCI3564897 | chr13 | 32906729 | rs144848 | A | C | 52.27 | 49.3 | BRCA2 | N370H | MISSENSE | NON_SYNON | P |
| UCI3564897 | chr13 | 32911463 | rs1799944 | A | G | 51.3 | 48.31 | BRCA2 | N991D | MISSENSE | NON_SYNON | P |
| UCI3564897 | chr13 | 32929387 | rs169547 | T | C | 99.9 | 100 | BRCA2 | V2466A | MISSENSE | NON_SYNON | N |
| UCI4216548 | chr13 | 32929387 | rs169547 | T | C | 99.72 | 99.72 | BRCA2 | V2466A | MISSENSE | NON_SYNON | N |
| UCI8965412 | chr13 | 32929387 | rs169547 | T | C | 63 | 71.34 | BRCA2 | V2466A | MISSENSE | NON_SYNON | N |
| UCI2008866 | chr13 | 32972626 | rs11571833 | A | T | 45.99 | 12.16 | BRCA2 | K3326* | NONSENSE | STOP_GAINE | D |
| UCI9135402 | chr13 | 32906729 | rs144848 | A | C | 51.08 | 51.13 | BRCA2 | N370H | MISSENSE | NON_SYNON | P |
| UCI9135402 | chr13 | 32929387 | rs169547 | T | C | 99.24 | 99.58 | BRCA2 | V2466A | MISSENSE | NON_SYNON | N |
| AA1247 | chr16 | 68855966 | rs35187787 | G | A | 54.15 | 59.36 | CDH1 | A531T | MISSENSE | NON_SYNON | N |
| UCI3165498 | chr16 | 68842734 | . | C | T | 45.15 | 37.66 | CDH1 | R224C | MISSENSE | NON_SYNON | N |
| UCI2224680 | chr9 | 21970916 | rs3731249 | C | T | 52.5 | 51.15 | CDKN2A | A148T | MISSENSE | NON_SYNON | N |
| AA1025 | chr7 | 117199533 | rs213950 | G | A | 99.32 | 99.33 | CFTR | V409M | MISSENSE | NON_SYNON | P |
| AA1025 | chr7 | 117227832 | rs113993959 | G | T | 50.84 | 55.83 | CFTR | G481* | NONSENSE | STOP_GAINE | A |
| AA1077 | chr7 | 117199533 | rs213950 | G | A | 44.47 | 81.3 | CFTR | V409M | MISSENSE | NON_SYNON | P |
| AA1090 | chr7 | 117199533 | rs213950 | G | A | 49.29 | 9.52 | CFTR | V409M | MISSENSE | NON_SYNON | P |
| AA943 | chr7 | 117199533 | rs213950 | G | A | 53.95 | 45.71 | CFTR | V409M | MISSENSE | NON_SYNON | P |
| AA1188 | chr7 | 117199533 | rs213950 | G | A | 49.63 | 45.96 | CFTR | V409M | MISSENSE | NON_SYNON | P |
| AA1204 | chr7 | 117199533 | rs213950 | G | A | 50.51 | 59.27 | CFTR | V409M | MISSENSE | NON_SYNON | P |
| AA1222 | chr7 | 117199533 | rs213950 | G | A | 53.5 | 50.22 | CFTR | V409M | MISSENSE | NON_SYNON | P |
| AA1247 | chr7 | 117199533 | rs213950 | G | A | 99.94 | 99.1 | CFTR | V409M | MISSENSE | NON_SYNON | P |
| UCI1821517 | chr7 | 117182116 | rs143860237 | C | T | 54.27 | 79.84 | CFTR | T358M | MISSENSE | NON_SYNON | D |
| AA1204 | chr7 | 117267812 | rs34911792 | T | G | 47.92 | 60.22 | CFTR | S1174R | MISSENSE | NON_SYNON | D |
| AA930 | chr7 | 117199533 | rs213950 | G | A | 55.13 | 48.58 | CFTR | V409M | MISSENSE | NON_SYNON | P |
| UCI2224680 | chr7 | 117267812 | rs34911792 | T | G | 58.03 | 50.95 | CFTR | S1174R | MISSENSE | NON_SYNON | D |
| AA1367 | chr7 | 117199533 | rs213950 | G | A | 52.05 | 47.22 | CFTR | V409M | MISSENSE | NON_SYNON | P |
| AA960 | chr7 | 117199533 | rs213950 | G | A | 47.27 | 47.84 | CFTR | V409M | MISSENSE | NON_SYNON | P |
| UCI1546879 | chr7 | 117199533 | rs213950 | G | A | 54.22 | 42.18 | CFTR | V409M | MISSENSE | NON_SYNON | P |
| UCI9135402 | chr7 | 117175323 | rs138338446 | G | A | 50.05 | 49.49 | CFTR | V171M | MISSENSE | NON_SYNON | D |
| UCI1689380 | chr7 | 117199533 | rs213950 | G | A | 57.08 | 34.56 | CFTR | V409M | MISSENSE | NON_SYNON | P |
| UCI1804937 | chr7 | 117199533 | rs213950 | G | A | 46.94 | 46.81 | CFTR | V409M | MISSENSE | NON_SYNON | P |
| UCI9135402 | chr7 | 117282582 | rs11971167 | G | A | 45.16 | 47.4 | CFTR | D1209N | MISSENSE | NON_SYNON | D |
| UCI1821517 | chr7 | 117199533 | rs213950 | G | A | 44.39 | 76.49 | CFTR | V409M | MISSENSE | NON_SYNON | P |
| UCI1908503 | chr7 | 117199533 | rs213950 | G | A | 99.59 | 99.73 | CFTR | V409M | MISSENSE | NON_SYNON | P |
| UCI9135402 | chr7 | 117149143 | rs115545701 | C | T | 51.85 | 45.86 | CFTR | R74W | MISSENSE | NON_SYNON | D |
| UCI1951813 | chr7 | 117199533 | rs213950 | G | A | 39.17 | 44.43 | CFTR | V409M | MISSENSE | NON_SYNON | P |
| UCI2008866 | chr7 | 117199533 | rs213950 | G | A | 50.12 | 35.77 | CFTR | V409M | MISSENSE | NON_SYNON | P |
| UCI3564897 | chr7 | 117171122 | rs35516286 | T | C | 45.19 | 43.52 | CFTR | I148T | MISSENSE | NON_SYNON | D |
| UCI2076630 | chr7 | 117199533 | rs213950 | G | A | 55.72 | 21.12 | CFTR | V409M | MISSENSE | NON_SYNON | P |
| UCI1546879 | chr7 | 117267812 | rs34911792 | T | G | 47.89 | 42.15 | CFTR | S1174R | MISSENSE | NON_SYNON | D |
| UCI2219330 | chr7 | 117199533 | rs213950 | G | A | 48.79 | 54.26 | CFTR | V409M | MISSENSE | NON_SYNON | P |
| UCI2224680 | chr7 | 117199533 | rs213950 | G | A | 41.5 | 55.67 | CFTR | V409M | MISSENSE | NON_SYNON | P |
| AA926 | chr7 | 117149147 | rs1800076 | G | A | 44.33 | 37.99 | CFTR | R75Q | MISSENSE | NON_SYNON | D |
| UCI2231576 | chr7 | 117199533 | rs213950 | G | A | 55.08 | 49.57 | CFTR | V409M | MISSENSE | NON_SYNON | P |
| UCI2564879 | chr7 | 117199533 | rs213950 | G | A | 99.53 | 99.43 | CFTR | V409M | MISSENSE | NON_SYNON | P |
| UCI2649875 | chr7 | 117199533 | rs213950 | G | A | 99.87 | 99.63 | CFTR | V409M | MISSENSE | NON_SYNON | P |
| AA943 | chr7 | 140481383 | . | CT | C | 16.89 | 16.78 | BRAF | NA | NONE | FRAME_SHIF | NA |
| UCI1908503 | chr7 | 6018228 | . | AT | A | 16.96 | 17.35 | PMS2 | NA | NONE | FRAME_SHIF | NA |
| UCI3165498 | chr7 | 117199533 | rs213950 | G | A | 55.95 | 49.08 | CFTR | V409M | MISSENSE | NON_SYNON | P |
| UCI3564897 | chr7 | 117199533 | rs213950 | G | A | 45.85 | 45.91 | CFTR | V409M | MISSENSE | NON_SYNON | P |
| UCI1951813 | chr5 | 149500767 | . | GT | G | 17.33 | 18.31 | PDGFRB | NA | NONE | FRAME_SHIF | NA |
| UCI1908503 | chr9 | 139391529 | . | GA | G | 17.34 | 18.78 | NOTCH1 | NA | NONE | FRAME_SHIF | NA |
| UCI1821517 | chr7 | 6018228 | . | AT | A | 17.47 | 17.88 | PMS2 | NA | NONE | FRAME_SHIF | NA |
| AA943 | chr7 | 117188715 | rs3034796 | GAAAGC | G | 18.03 | 19.82 | CFTR | NA | NONE | FRAME_SHIF | NA |
| AA1277 | chr7 | 117188715 | rs3034796 | GAAAGC | G | 18.34 | 13.2 | CFTR | NA | NONE | FRAME_SHIF | NA |
| AA1025 | chr7 | 117188715 | rs3034796 | GAAAGC | G | 18.54 | 15 | CFTR | NA | NONE | FRAME_SHIF | NA |
| UCI2224680 | chr7 | 117188715 | rs3034796 | GAAAGC | G | 18.72 | 17.4 | CFTR | NA | NONE | FRAME_SHIF | NA |
| UCI1951813 | chr9 | 139390569 | . | GT | G | 19.17 | 17.98 | NOTCH1 | NA | NONE | FRAME_SHIF | NA |
| AA1367 | chr9 | 139390569 | . | GT | G | 19.93 | 17.48 | NOTCH1 | NA | NONE | FRAME_SHIF | NA |
| UCI1804937 | chr3 | 37090032 | . | CT | C | 20.3 | 19.94 | MLH1 | NA | NONE | FRAME_SHIF | NA |
| UCI9135402 | chr7 | 117199533 | rs213950 | G | A | 54.77 | 50.16 | CFTR | V409M | MISSENSE | NON_SYNON | P |
| AA960 | chr22 | 29121087 | rs17879961 | A | G | 46.53 | 62.59 | CHEK2 | I157T | MISSENSE | NON_SYNON | NA |
| UCI2649875 | chr22 | 29121087 | rs17879961 | A | G | 57.08 | 49.87 | CHEK2 | I157T | MISSENSE | NON_SYNON | NA |
| UCI2076630 | chr22 | 29126440 | . | A | G | 59.77 | 43.31 | CHEK2 | V139A | MISSENSE | NON_SYNON | NA |
| AA960 | chr5 | 138160248 | rs150893072 | G | C | 47.84 | 28.06 | CTNAA1 | Q103H | MISSENSE | NON_SYNON | D |
| AA1077 | chr10 | 96702066 | rs7900194 | G | A | 46.19 | 24.91 | CYP2C9 | R150H | MISSENSE | NON_SYNON | N |
| UCI3564897 | chr10 | 96702047 | rs1799853 | C | T | 53.16 | 81.91 | CYP2C9 | R144C | MISSENSE | NON_SYNON | D |
| UCI2649875 | chr10 | 96702047 | rs1799853 | C | T | 43.89 | 61.77 | CYP2C9 | R144C | MISSENSE | NON_SYNON | D |
| AA1307 | chr10 | 96741053 | rs1057910 | A | C | 45.58 | 51.45 | CYP2C9 | I359L | MISSENSE | NON_SYNON | D |

**Table S8: List of non-silent germline variants**

| Sample | Chr | Cord | rsID | Ref Allele | Alt Allele | Allelic Fraction | | Gene | AA change | Type | Effect | Mutation Taster Prediction |
|---|---|---|---|---|---|---|---|---|---|---|---|---|
| | | | | | | Normal | Tumor | | | | | |
| AA948 | chr10 | 96748777 | rs9332239 | C | T | 48.21 | 51.16 | CYP2C9 | P489S | MISSENSE | NON_SYNON | D |
| UCI2219330 | chr10 | 96741053 | rs1057910 | A | C | 46.05 | 50.95 | CYP2C9 | I359L | MISSENSE | NON_SYNON | D |
| AA948 | chr10 | 96702047 | rs1799853 | C | T | 55.15 | 50.79 | CYP2C9 | R144C | MISSENSE | NON_SYNON | D |
| AA1515 | chr10 | 96702047 | rs1799853 | C | T | 52.95 | 50.26 | CYP2C9 | R144C | MISSENSE | NON_SYNON | D |
| UCI1689380 | chr10 | 96741053 | rs1057910 | A | C | 49.72 | 49.81 | CYP2C9 | I359L | MISSENSE | NON_SYNON | D |
| AA926 | chr10 | 96702047 | rs1799853 | C | T | 47.71 | 49.78 | CYP2C9 | R144C | MISSENSE | NON_SYNON | D |
| UCI1689380 | chr10 | 96702047 | rs1799853 | C | T | 54.96 | 49.46 | CYP2C9 | R144C | MISSENSE | NON_SYNON | D |
| AA1267 | chr10 | 96702047 | rs1799853 | C | T | 51.72 | 47.7 | CYP2C9 | R144C | MISSENSE | NON_SYNON | D |
| AA952 | chr10 | 96741053 | rs1057910 | A | C | 52.58 | 47.03 | CYP2C9 | I359L | MISSENSE | NON_SYNON | D |
| AA1025 | chr10 | 96702047 | rs1799853 | C | T | 48.83 | 45.36 | CYP2C9 | R144C | MISSENSE | NON_SYNON | D |
| UCI8965412 | chr10 | 96740981 | rs28371685 | C | T | 49.46 | 72.69 | CYP2C9 | R335W | MISSENSE | NON_SYNON | P |
| AA1188 | chr22 | 42522613 | rs1135840 | G | C | 100 | 100 | CYP2D6 | T432S | MISSENSE | NON_SYNON | NA |
| UCI1821517 | chr22 | 42523943 | rs16947 | A | G | 99.89 | 100 | CYP2D6 | C242R | MISSENSE | NON_SYNON | NA |
| UCI2231576 | chr22 | 42522613 | rs1135840 | G | C | 97.63 | 100 | CYP2D6 | T432S | MISSENSE | NON_SYNON | NA |
| UCI9135402 | chr22 | 42523943 | rs16947 | A | G | 99.85 | 100 | CYP2D6 | C242R | MISSENSE | NON_SYNON | NA |
| UCI1951813 | chr22 | 42523943 | rs16947 | A | G | 99.36 | 99.94 | CYP2D6 | C242R | MISSENSE | NON_SYNON | NA |
| AA952 | chr22 | 42523943 | rs16947 | A | G | 99.51 | 99.93 | CYP2D6 | C242R | MISSENSE | NON_SYNON | NA |
| AA1188 | chr22 | 42523943 | rs16947 | A | G | 99.91 | 99.86 | CYP2D6 | C242R | MISSENSE | NON_SYNON | NA |
| AA1267 | chr22 | 42522613 | rs1135840 | G | C | 99.93 | 99.86 | CYP2D6 | T432S | MISSENSE | NON_SYNON | NA |
| UCI2231576 | chr22 | 42523943 | rs16947 | A | G | 99.02 | 99.81 | CYP2D6 | C242R | MISSENSE | NON_SYNON | NA |
| AA1090 | chr22 | 42523943 | rs16947 | A | G | 99.86 | 99.8 | CYP2D6 | C242R | MISSENSE | NON_SYNON | NA |
| UCI2564879 | chr22 | 42523943 | rs16947 | A | G | 99.66 | 99.79 | CYP2D6 | C242R | MISSENSE | NON_SYNON | NA |
| AA1106 | chr22 | 42523943 | rs16947 | A | G | 99.96 | 99.77 | CYP2D6 | C242R | MISSENSE | NON_SYNON | NA |
| AA1367 | chr22 | 42523943 | rs16947 | A | G | 99.84 | 99.77 | CYP2D6 | C242R | MISSENSE | NON_SYNON | NA |
| UCI1908503 | chr22 | 42523943 | rs16947 | A | G | 99.41 | 99.76 | CYP2D6 | C242R | MISSENSE | NON_SYNON | NA |
| AA1025 | chr22 | 42523943 | rs16947 | A | G | 99.71 | 99.72 | CYP2D6 | C242R | MISSENSE | NON_SYNON | NA |
| UCI2649875 | chr22 | 42522613 | rs1135840 | G | C | 99.2 | 99.68 | CYP2D6 | T432S | MISSENSE | NON_SYNON | NA |
| AA1267 | chr22 | 42523943 | rs16947 | A | G | 99.87 | 99.63 | CYP2D6 | C242R | MISSENSE | NON_SYNON | NA |
| UCI2008866 | chr22 | 42523943 | rs16947 | A | G | 99.89 | 99.57 | CYP2D6 | C242R | MISSENSE | NON_SYNON | NA |
| UCI3165498 | chr22 | 42523943 | rs16947 | A | G | 99.53 | 99.54 | CYP2D6 | C242R | MISSENSE | NON_SYNON | NA |
| AA960 | chr22 | 42523943 | rs16947 | A | G | 99.84 | 99.4 | CYP2D6 | C242R | MISSENSE | NON_SYNON | NA |
| UCI2649875 | chr22 | 42523943 | rs16947 | A | G | 99.21 | 99.39 | CYP2D6 | C242R | MISSENSE | NON_SYNON | NA |
| UCI2564879 | chr22 | 42526694 | rs1065852 | G | A | 99.55 | 99.36 | CYP2D6 | P34S | MISSENSE | NON_SYNON | NA |
| AA1247 | chr22 | 42523943 | rs16947 | A | G | 99.59 | 99.35 | CYP2D6 | C242R | MISSENSE | NON_SYNON | NA |
| AA1204 | chr22 | 42523943 | rs16947 | A | G | 99.59 | 99.28 | CYP2D6 | C242R | MISSENSE | NON_SYNON | NA |
| UCI3165498 | chr22 | 42522613 | rs1135840 | G | C | 99.59 | 99.27 | CYP2D6 | T432S | MISSENSE | NON_SYNON | NA |
| AA1106 | chr22 | 42522613 | rs1135840 | G | C | 99.73 | 98.95 | CYP2D6 | T432S | MISSENSE | NON_SYNON | NA |
| AA1515 | chr22 | 42522613 | rs1135840 | G | C | 91.42 | 94.89 | CYP2D6 | T432S | MISSENSE | NON_SYNON | NA |
| UCI9135402 | chr22 | 42526694 | rs1065852 | G | A | 95.62 | 94.83 | CYP2D6 | P34S | MISSENSE | NON_SYNON | NA |
| UCI2008866 | chr22 | 42526694 | rs1065852 | G | A | 68.22 | 93.02 | CYP2D6 | P34S | MISSENSE | NON_SYNON | NA |
| UCI1689380 | chr22 | 42523943 | rs16947 | A | G | 46.82 | 92.63 | CYP2D6 | C242R | MISSENSE | NON_SYNON | NA |
| UCI1689380 | chr22 | 42522613 | rs1135840 | G | C | 50.25 | 90.03 | CYP2D6 | T432S | MISSENSE | NON_SYNON | NA |
| UCI3165498 | chr22 | 42526712 | rs138100349 | G | A | 47.01 | 88.58 | CYP2D6 | R28C | MISSENSE | NON_SYNONYMOUS_CODING | |
| UCI1821517 | chr22 | 42526694 | rs1065852 | G | A | 60.65 | 85.04 | CYP2D6 | P34S | MISSENSE | NON_SYNON | NA |
| AA1090 | chr22 | 42522613 | rs1135840 | G | C | 53.3 | 84.61 | CYP2D6 | T432S | MISSENSE | NON_SYNON | NA |
| AA1367 | chr22 | 42526694 | rs1065852 | G | A | 64.72 | 75.39 | CYP2D6 | P34S | MISSENSE | NON_SYNON | NA |
| AA1247 | chr22 | 42526694 | rs1065852 | G | A | 64.51 | 73.07 | CYP2D6 | P34S | MISSENSE | NON_SYNON | NA |
| UCI1951813 | chr22 | 42526694 | rs1065852 | G | A | 63.7 | 67.61 | CYP2D6 | P34S | MISSENSE | NON_SYNON | NA |
| UCI2008866 | chr22 | 42523528 | rs1058172 | C | T | 50.33 | 64.96 | CYP2D6 | R311H | MISSENSE | NON_SYNON | NA |
| AA1025 | chr22 | 42526694 | rs1065852 | G | A | 48.1 | 63.65 | CYP2D6 | P34S | MISSENSE | NON_SYNON | NA |
| UCI1908503 | chr22 | 42522613 | rs1135840 | G | C | 51.17 | 61.43 | CYP2D6 | T432S | MISSENSE | NON_SYNON | NA |
| UCI9135402 | chr22 | 42523528 | rs1058172 | C | T | 64.95 | 60.28 | CYP2D6 | R311H | MISSENSE | NON_SYNON | NA |
| AA926 | chr22 | 42523943 | rs16947 | A | G | 52.72 | 59.91 | CYP2D6 | C242R | MISSENSE | NON_SYNON | NA |
| UCI1821517 | chr22 | 42523528 | rs1058172 | C | T | 56.49 | 59.07 | CYP2D6 | R311H | MISSENSE | NON_SYNON | NA |
| AA926 | chr22 | 42526694 | rs1065852 | G | A | 50.36 | 58.85 | CYP2D6 | P34S | MISSENSE | NON_SYNON | NA |
| UCI1804937 | chr22 | 42522613 | rs1135840 | G | C | 54.98 | 55.44 | CYP2D6 | T432S | MISSENSE | NON_SYNON | NA |
| AA960 | chr22 | 42522613 | rs1135840 | G | C | 44.94 | 55.2 | CYP2D6 | T432S | MISSENSE | NON_SYNON | NA |
| UCI1804937 | chr22 | 42523943 | rs16947 | A | G | 48.75 | 55.15 | CYP2D6 | C242R | MISSENSE | NON_SYNON | NA |
| UCI2219330 | chr22 | 42523943 | rs16947 | A | G | 47.83 | 54.82 | CYP2D6 | C242R | MISSENSE | NON_SYNON | NA |
| AA948 | chr22 | 42522613 | rs1135840 | G | C | 46.24 | 54.58 | CYP2D6 | T432S | MISSENSE | NON_SYNON | NA |
| AA1515 | chr22 | 42523943 | rs16947 | A | G | 49.61 | 54.12 | CYP2D6 | C242R | MISSENSE | NON_SYNON | NA |
| AA1204 | chr22 | 42522613 | rs1135840 | G | C | 49.38 | 53.99 | CYP2D6 | T432S | MISSENSE | NON_SYNON | NA |
| UCI1908503 | chr22 | 42526694 | rs1065852 | G | A | 64.81 | 52.97 | CYP2D6 | P34S | MISSENSE | NON_SYNON | NA |
| AA952 | chr22 | 42522613 | rs1135840 | G | C | 49.52 | 52.78 | CYP2D6 | T432S | MISSENSE | NON_SYNON | NA |
| AA1367 | chr9 | 139391529 | . | GA | G | 20.96 | 21.41 | NOTCH1 | NA | NONE | FRAME_SHIF | NA |
| UCI2219330 | chr22 | 42522613 | rs1135840 | G | C | 51.69 | 50.76 | CYP2D6 | T432S | MISSENSE | NON_SYNON | NA |
| UCI1951813 | chr22 | 42523528 | rs1058172 | C | T | 48.49 | 50.34 | CYP2D6 | R311H | MISSENSE | NON_SYNON | NA |
| AA1307 | chr22 | 42523943 | rs16947 | A | G | 47.81 | 49.93 | CYP2D6 | C242R | MISSENSE | NON_SYNON | NA |
| AA1204 | chr22 | 42526694 | rs1065852 | G | A | 50.98 | 49.5 | CYP2D6 | P34S | MISSENSE | NON_SYNON | NA |
| AA957 | chr22 | 42523943 | rs16947 | A | G | 55.47 | 49.1 | CYP2D6 | C242R | MISSENSE | NON_SYNON | NA |
| AA943 | chr22 | 42523943 | rs16947 | A | G | 47.32 | 48.95 | CYP2D6 | C242R | MISSENSE | NON_SYNON | NA |
| AA952 | chr22 | 42526694 | rs1065852 | G | A | 48.47 | 48.9 | CYP2D6 | P34S | MISSENSE | NON_SYNON | NA |
| AA957 | chr22 | 42526694 | rs1065852 | G | A | 48.89 | 48.76 | CYP2D6 | P34S | MISSENSE | NON_SYNON | NA |
| UCI2649875 | chr7 | 116371802 | . | TG | T | 21.12 | 21.01 | MET | NA | NONE | FRAME_SHIF | NA |
| AA1307 | chr22 | 42522613 | rs1135840 | G | C | 44.56 | 48.16 | CYP2D6 | T432S | MISSENSE | NON_SYNON | NA |
| AA948 | chr22 | 42523943 | rs16947 | A | G | 49.13 | 48.08 | CYP2D6 | C242R | MISSENSE | NON_SYNON | NA |
| AA1077 | chr22 | 42526694 | rs1065852 | G | A | 51.19 | 47.85 | CYP2D6 | P34S | MISSENSE | NON_SYNON | NA |
| UCI1908503 | chr22 | 42523528 | rs1058172 | C | T | 47.83 | 47.85 | CYP2D6 | R311H | MISSENSE | NON_SYNON | NA |
| AA1247 | chr22 | 42523528 | rs1058172 | C | T | 50.24 | 47.84 | CYP2D6 | R311H | MISSENSE | NON_SYNON | NA |
| AA943 | chr22 | 42522613 | rs1135840 | G | C | 46.7 | 46.83 | CYP2D6 | T432S | MISSENSE | NON_SYNON | NA |
| AA1077 | chr22 | 42523943 | rs16947 | A | G | 50.18 | 46.7 | CYP2D6 | C242R | MISSENSE | NON_SYNON | NA |
| AA1222 | chr22 | 42523943 | rs16947 | A | G | 53.04 | 45.92 | CYP2D6 | C242R | MISSENSE | NON_SYNON | NA |
| UCI1951813 | chr22 | 42522613 | rs1135840 | G | C | 48.69 | 43.55 | CYP2D6 | T432S | MISSENSE | NON_SYNON | NA |
| UCI4216548 | chr22 | 42522613 | rs1135840 | G | C | 44.3 | 43.53 | CYP2D6 | T432S | MISSENSE | NON_SYNON | NA |
| AA1222 | chr22 | 42522613 | rs1135840 | G | C | 48.15 | 43.33 | CYP2D6 | T432S | MISSENSE | NON_SYNON | NA |
| UCI2076630 | chr22 | 42523943 | rs16947 | A | G | 46.39 | 43.05 | CYP2D6 | C242R | MISSENSE | NON_SYNON | NA |
| AA1025 | chr22 | 42523528 | rs1058172 | C | T | 32.27 | 42.14 | CYP2D6 | R311H | MISSENSE | NON_SYNON | NA |
| UCI2076630 | chr22 | 42522613 | rs1135840 | G | C | 58.98 | 41.69 | CYP2D6 | T432S | MISSENSE | NON_SYNON | NA |
| AA1247 | chr22 | 42522613 | rs1135840 | G | C | 49.4 | 41.33 | CYP2D6 | T432S | MISSENSE | NON_SYNON | NA |
| AA1367 | chr22 | 42522613 | rs1135840 | G | C | 50.73 | 40.72 | CYP2D6 | T432S | MISSENSE | NON_SYNON | NA |
| UCI4216548 | chr22 | 42523943 | rs16947 | A | G | 52.92 | 39.74 | CYP2D6 | C242R | MISSENSE | NON_SYNON | NA |
| AA948 | chr7 | 117188715 | rs3034796 | GAAAGC | G | 22.29 | 14.68 | CFTR | NA | NONE | FRAME_SHIF | NA |
| AA960 | chr22 | 42526694 | rs1065852 | G | A | 50.98 | 35.29 | CYP2D6 | P34S | MISSENSE | NON_SYNON | NA |
| AA926 | chr22 | 42523528 | rs1058172 | C | T | 32.07 | 35.02 | CYP2D6 | R311H | MISSENSE | NON_SYNON | NA |
| AA957 | chr22 | 42523528 | rs1058172 | C | T | 31.75 | 34.62 | CYP2D6 | R311H | MISSENSE | NON_SYNON | NA |
| AA1025 | chr22 | 42522613 | rs1135840 | G | C | 42.71 | 34.35 | CYP2D6 | T432S | MISSENSE | NON_SYNON | NA |
| AA943 | chr22 | 42524817 | rs139779104 | C | T | 29.99 | 33.74 | CYP2D6 | G158E | MISSENSE | NON_SYNON | NA |
| AA1077 | chr22 | 42523528 | rs1058172 | C | T | 30.18 | 32.85 | CYP2D6 | R311H | MISSENSE | NON_SYNON | NA |
| UCI2224680 | chr22 | 42522613 | rs1135840 | G | C | 46.51 | 30.36 | CYP2D6 | T432S | MISSENSE | NON_SYNON | NA |
| UCI2224680 | chr22 | 42523943 | rs16947 | A | G | 50.52 | 29.59 | CYP2D6 | C242R | MISSENSE | NON_SYNON | NA |

**Table S8: List of non-silent germline variants**

| Sample | Chr | Cord | rsID | Ref Allele | Alt Allele | Allelic Fraction | | Gene | AA change | Type | Effect | Mutation Taster Prediction |
|---|---|---|---|---|---|---|---|---|---|---|---|---|
| | | | | | | Normal | Tumor | | | | | |
| AA1204 | chr22 | 42523528 | rs1058172 | C | T | 31.48 | 27.85 | CYP2D6 | R311H | MISSENSE | NON_SYNON` | NA |
| UCI1821517 | chr22 | 42522613 | rs1135840 | G | C | 48.37 | 23.26 | CYP2D6 | T432S | MISSENSE | NON_SYNON` | NA |
| AA1090 | chr22 | 42526694 | rs1065852 | G | A | 47.13 | 15.31 | CYP2D6 | P34S | MISSENSE | NON_SYNON` | NA |
| UCI2564879 | chr7 | 117188715 | rs3034796 | GAAAGC | G | 22.39 | 17.15 | CFTR | NA | NONE | FRAME_SHIF` | NA |
| AA1090 | chr22 | 42523528 | rs1058172 | C | T | 33.49 | 10.91 | CYP2D6 | R311H | MISSENSE | NON_SYNON` | NA |
| UCI2008866 | chr22 | 42522613 | rs1135840 | G | C | 51.51 | 7.63 | CYP2D6 | T432S | MISSENSE | NON_SYNON` | NA |
| AA1367 | chr1 | 97547962 | . | A | G | 50.98 | 49.64 | DPYD | V944A | MISSENSE | NON_SYNON` | D |
| UCI2008866 | chr1 | 97770920 | rs1801160 | C | T | 42.18 | 49.77 | DPYD | V732I | MISSENSE | NON_SYNON` | D |
| UCI1804937 | chr1 | 97770920 | rs1801160 | C | T | 48.73 | 48.91 | DPYD | V732I | MISSENSE | NON_SYNON` | D |
| AA952 | chr1 | 97770920 | rs1801160 | C | T | 48.21 | 47.18 | DPYD | V732I | MISSENSE | NON_SYNON` | D |
| UCI1546879 | chr1 | 98144726 | rs45589337 | T | C | 49.13 | 45.82 | DPYD | K259E | MISSENSE | NON_SYNON` | D |
| AA948 | chr1 | 98144726 | rs45589337 | T | C | 48.18 | 44.7 | DPYD | K259E | MISSENSE | NON_SYNON` | D |
| AA1025 | chr1 | 98348885 | rs1801265 | G | A | 53.3 | 53.25 | DPYD | R29C | MISSENSE | NON_SYNON` | None |
| AA1077 | chr1 | 98348885 | rs1801265 | G | A | 50.87 | 69.92 | DPYD | R29C | MISSENSE | NON_SYNON` | None |
| AA1090 | chr1 | 98348885 | rs1801265 | G | A | 44.09 | 51.46 | DPYD | R29C | MISSENSE | NON_SYNON` | None |
| AA943 | chr1 | 98348885 | rs1801265 | G | A | 99.67 | 99.85 | DPYD | R29C | MISSENSE | NON_SYNON` | None |
| AA1188 | chr1 | 98348885 | rs1801265 | G | A | 99.84 | 99.64 | DPYD | R29C | MISSENSE | NON_SYNON` | None |
| AA1204 | chr1 | 98348885 | rs1801265 | G | A | 99.73 | 99.32 | DPYD | R29C | MISSENSE | NON_SYNON` | None |
| AA1222 | chr1 | 98348885 | rs1801265 | G | A | 99.72 | 99.57 | DPYD | R29C | MISSENSE | NON_SYNON` | None |
| AA1247 | chr1 | 98348885 | rs1801265 | G | A | 99.82 | 99.9 | DPYD | R29C | MISSENSE | NON_SYNON` | None |
| AA1267 | chr1 | 98348885 | rs1801265 | G | A | 99.81 | 99.8 | DPYD | R29C | MISSENSE | NON_SYNON` | None |
| AA1277 | chr1 | 98348885 | rs1801265 | G | A | 99.59 | 98.89 | DPYD | R29C | MISSENSE | NON_SYNON` | None |
| AA1307 | chr1 | 98348885 | rs1801265 | G | A | 48.69 | 57.06 | DPYD | R29C | MISSENSE | NON_SYNON` | None |
| AA957 | chr1 | 98348885 | rs1801265 | G | A | 99.64 | 99.58 | DPYD | R29C | MISSENSE | NON_SYNON` | None |
| AA926 | chr1 | 98348885 | rs1801265 | G | A | 46.27 | 61.67 | DPYD | R29C | MISSENSE | NON_SYNON` | None |
| AA930 | chr1 | 98348885 | rs1801265 | G | A | 99.69 | 100 | DPYD | R29C | MISSENSE | NON_SYNON` | None |
| AA1106 | chr1 | 98348885 | rs1801265 | G | A | 46.9 | 41.07 | DPYD | R29C | MISSENSE | NON_SYNON` | None |
| AA952 | chr1 | 98348885 | rs1801265 | G | A | 99.05 | 98.43 | DPYD | R29C | MISSENSE | NON_SYNON` | None |
| AA1367 | chr1 | 98348885 | rs1801265 | G | A | 49.68 | 48.17 | DPYD | R29C | MISSENSE | NON_SYNON` | None |
| AA960 | chr1 | 98348885 | rs1801265 | G | A | 99.18 | 99.67 | DPYD | R29C | MISSENSE | NON_SYNON` | None |
| AA1515 | chr1 | 98348885 | rs1801265 | G | A | 99.68 | 100 | DPYD | R29C | MISSENSE | NON_SYNON` | None |
| UCI1546879 | chr1 | 98348885 | rs1801265 | G | A | 54.6 | 46.67 | DPYD | R29C | MISSENSE | NON_SYNON` | None |
| UCI1689380 | chr1 | 98348885 | rs1801265 | G | A | 49.26 | 7.82 | DPYD | R29C | MISSENSE | NON_SYNON` | None |
| UCI1804937 | chr1 | 98348885 | rs1801265 | G | A | 99.56 | 99.72 | DPYD | R29C | MISSENSE | NON_SYNON` | None |
| UCI1821517 | chr1 | 98348885 | rs1801265 | G | A | 99.85 | 99.74 | DPYD | R29C | MISSENSE | NON_SYNON` | None |
| UCI1908503 | chr1 | 98348885 | rs1801265 | G | A | 99.16 | 99.44 | DPYD | R29C | MISSENSE | NON_SYNON` | None |
| UCI1951813 | chr1 | 98348885 | rs1801265 | G | A | 99.36 | 99.46 | DPYD | R29C | MISSENSE | NON_SYNON` | None |
| UCI2008866 | chr1 | 98348885 | rs1801265 | G | A | 100 | 99.81 | DPYD | R29C | MISSENSE | NON_SYNON` | None |
| UCI2076630 | chr1 | 98348885 | rs1801265 | G | A | 99.74 | 99.63 | DPYD | R29C | MISSENSE | NON_SYNON` | None |
| UCI2219330 | chr1 | 98348885 | rs1801265 | G | A | 49.84 | 51.54 | DPYD | R29C | MISSENSE | NON_SYNON` | None |
| UCI2224680 | chr1 | 98348885 | rs1801265 | G | A | 99.76 | 99.62 | DPYD | R29C | MISSENSE | NON_SYNON` | None |
| UCI2231576 | chr1 | 98348885 | rs1801265 | G | A | 99.04 | 100 | DPYD | R29C | MISSENSE | NON_SYNON` | None |
| UCI2564879 | chr1 | 98348885 | rs1801265 | G | A | 99.52 | 99.52 | DPYD | R29C | MISSENSE | NON_SYNON` | None |
| UCI2649875 | chr1 | 98348885 | rs1801265 | G | A | 99.66 | 99.61 | DPYD | R29C | MISSENSE | NON_SYNON` | None |
| UCI3165498 | chr1 | 98348885 | rs1801265 | G | A | 99.54 | 99.55 | DPYD | R29C | MISSENSE | NON_SYNON` | None |
| UCI3564897 | chr1 | 98348885 | rs1801265 | G | A | 56.6 | 62.3 | DPYD | R29C | MISSENSE | NON_SYNON` | None |
| UCI4216548 | chr1 | 98348885 | rs1801265 | G | A | 42.97 | 34.24 | DPYD | R29C | MISSENSE | NON_SYNON` | None |
| UCI8965412 | chr1 | 98348885 | rs1801265 | G | A | 50 | 28.43 | DPYD | R29C | MISSENSE | NON_SYNON` | None |
| UCI9135402 | chr1 | 98348885 | rs1801265 | G | A | 99.32 | 99.29 | DPYD | R29C | MISSENSE | NON_SYNON` | None |
| AA1025 | chr1 | 98165091 | rs2297595 | T | C | 50.68 | 52.45 | DPYD | M166V | MISSENSE | NON_SYNON` | P |
| AA1277 | chr1 | 97981395 | rs1801159 | T | C | 50.7 | 51.41 | DPYD | I543V | MISSENSE | NON_SYNON` | P |
| AA1307 | chr1 | 97981395 | rs1801159 | T | C | 55.5 | 58 | DPYD | I543V | MISSENSE | NON_SYNON` | P |
| AA926 | chr1 | 97981395 | rs1801159 | T | C | 49.05 | 65.39 | DPYD | I543V | MISSENSE | NON_SYNON` | P |
| AA1106 | chr1 | 98165091 | rs2297595 | T | C | 51.31 | 56.62 | DPYD | M166V | MISSENSE | NON_SYNON` | P |
| AA1515 | chr1 | 97981395 | rs1801159 | T | C | 55.66 | 23.34 | DPYD | I543V | MISSENSE | NON_SYNON` | P |
| UCI1689380 | chr1 | 97981395 | rs1801159 | T | C | 50.67 | 8.63 | DPYD | I543V | MISSENSE | NON_SYNON` | P |
| UCI1821517 | chr1 | 97981395 | rs1801159 | T | C | 52.06 | 37.27 | DPYD | I543V | MISSENSE | NON_SYNON` | P |
| UCI1908503 | chr1 | 97981395 | rs1801159 | T | C | 53.61 | 51.82 | DPYD | I543V | MISSENSE | NON_SYNON` | P |
| UCI1951813 | chr1 | 97981395 | rs1801159 | T | C | 50.22 | 45.62 | DPYD | I543V | MISSENSE | NON_SYNON` | P |
| UCI2008866 | chr1 | 97981395 | rs1801159 | T | C | 50.47 | 48.61 | DPYD | I543V | MISSENSE | NON_SYNON` | P |
| UCI2076630 | chr1 | 97981395 | rs1801159 | T | C | 52.51 | 40.7 | DPYD | I543V | MISSENSE | NON_SYNON` | P |
| UCI2224680 | chr1 | 97981395 | rs1801159 | T | C | 49.21 | 51.76 | DPYD | I543V | MISSENSE | NON_SYNON` | P |
| UCI2231576 | chr1 | 97981395 | rs1801159 | T | C | 46.64 | 46.7 | DPYD | I543V | MISSENSE | NON_SYNON` | P |
| UCI2564879 | chr1 | 97981395 | rs1801159 | T | C | 56.11 | 58.89 | DPYD | I543V | MISSENSE | NON_SYNON` | P |
| UCI8965412 | chr1 | 98165091 | rs2297595 | T | C | 44.19 | 65.79 | DPYD | M166V | MISSENSE | NON_SYNON` | P |
| AA1222 | chr7 | 55214348 | rs2072454 | C | T | 99.76 | 99.94 | EGFR | NA | NONE | SPLICE_SITE_ACCEPTOR | |
| AA1077 | chr7 | 55229255 | rs2227983 | G | A | 47.74 | 26.73 | EGFR | R315K | MISSENSE | NON_SYNON` | N |
| UCI2076630 | chr7 | 55214348 | rs2072454 | C | T | 99.43 | 99.8 | EGFR | NA | NONE | SPLICE_SITE_ACCEPTOR | |
| UCI2564879 | chr7 | 55214348 | rs2072454 | C | T | 99.66 | 99.74 | EGFR | NA | NONE | SPLICE_SITE_ACCEPTOR | |
| UCI2008866 | chr7 | 55214348 | rs2072454 | C | T | 99.83 | 99.63 | EGFR | NA | NONE | SPLICE_SITE_ACCEPTOR | |
| UCI2231576 | chr7 | 55214348 | rs2072454 | C | T | 99.24 | 99.55 | EGFR | NA | NONE | SPLICE_SITE_ACCEPTOR | |
| UCI3165498 | chr7 | 55214348 | rs2072454 | C | T | 99.62 | 99.43 | EGFR | NA | NONE | SPLICE_SITE_ACCEPTOR | |
| AA1277 | chr7 | 55214348 | rs2072454 | C | T | 98.96 | 99.39 | EGFR | NA | NONE | SPLICE_SITE_ACCEPTOR | |
| AA1367 | chr7 | 55214348 | rs2072454 | C | T | 98.96 | 98.71 | EGFR | NA | NONE | SPLICE_SITE_ACCEPTOR | |
| UCI1951813 | chr7 | 55214348 | rs2072454 | C | T | 88.82 | 88.5 | EGFR | NA | NONE | SPLICE_SITE_ACCEPTOR | |
| AA1277 | chr7 | 55229255 | rs2227983 | G | A | 51.62 | 49.69 | EGFR | R315K | MISSENSE | NON_SYNON` | N |
| UCI2649875 | chr7 | 55214348 | rs2072454 | C | T | 50.78 | 58.97 | EGFR | NA | NONE | SPLICE_SITE_ACCEPTOR | |
| UCI1546879 | chr7 | 55211102 | . | T | A | 51.88 | 58.71 | EGFR | N115K | MISSENSE | NON_SYNON` | D |
| UCI1546879 | chr7 | 55214348 | rs2072454 | C | T | 46.21 | 57.54 | EGFR | NA | NONE | SPLICE_SITE_ACCEPTOR | |
| AA1204 | chr7 | 55214348 | rs2072454 | C | T | 50.15 | 56.03 | EGFR | NA | NONE | SPLICE_SITE_ACCEPTOR | |
| UCI1908503 | chr7 | 55214348 | rs2072454 | C | T | 39.01 | 55.59 | EGFR | NA | NONE | SPLICE_SITE_ACCEPTOR | |
| AA1106 | chr7 | 55214348 | rs2072454 | C | T | 48.9 | 53.49 | EGFR | NA | NONE | SPLICE_SITE_ACCEPTOR | |
| AA1188 | chr7 | 55214348 | rs2072454 | C | T | 49.73 | 53.42 | EGFR | NA | NONE | SPLICE_SITE_ACCEPTOR | |
| AA952 | chr7 | 55229255 | rs2227983 | G | A | 54.89 | 40.55 | EGFR | R315K | MISSENSE | NON_SYNON` | N |
| UCI8965412 | chr7 | 55214348 | rs2072454 | C | T | 51.71 | 52.08 | EGFR | NA | NONE | SPLICE_SITE_ACCEPTOR | |
| AA1367 | chr7 | 55229255 | rs2227983 | G | A | 49.66 | 47.64 | EGFR | R315K | MISSENSE | NON_SYNON` | N |
| AA943 | chr7 | 55214348 | rs2072454 | C | T | 49.79 | 50.81 | EGFR | NA | NONE | SPLICE_SITE_ACCEPTOR | |
| AA960 | chr7 | 55229255 | rs2227983 | G | A | 50.73 | 51.64 | EGFR | R315K | MISSENSE | NON_SYNON` | N |
| UCI1546879 | chr7 | 55229255 | rs2227983 | G | A | 38.63 | 55.89 | EGFR | R315K | MISSENSE | NON_SYNON` | N |
| AA1025 | chr7 | 55214348 | rs2072454 | C | T | 52.34 | 49.34 | EGFR | NA | NONE | SPLICE_SITE_ACCEPTOR | |
| UCI2224680 | chr7 | 55214348 | rs2072454 | C | T | 49.53 | 49.17 | EGFR | NA | NONE | SPLICE_SITE_ACCEPTOR | |
| AA948 | chr7 | 55214348 | rs2072454 | C | T | 45.64 | 48.17 | EGFR | NA | NONE | SPLICE_SITE_ACCEPTOR | |
| UCI1804937 | chr7 | 55229255 | rs2227983 | G | A | 48.2 | 44.07 | EGFR | R315K | MISSENSE | NON_SYNON` | N |
| UCI1821517 | chr7 | 55229255 | rs2227983 | G | A | 97.35 | 96.5 | EGFR | R315K | MISSENSE | NON_SYNON` | N |
| UCI2219330 | chr7 | 55214348 | rs2072454 | C | T | 47.07 | 46.7 | EGFR | NA | NONE | SPLICE_SITE_ACCEPTOR | |
| UCI1951813 | chr7 | 55229255 | rs2227983 | G | A | 48.27 | 47.56 | EGFR | R315K | MISSENSE | NON_SYNON` | N |
| AA930 | chr7 | 55214348 | rs2072454 | C | T | 49.59 | 45.55 | EGFR | NA | NONE | SPLICE_SITE_ACCEPTOR | |
| UCI1804937 | chr7 | 55223641 | . | T | G | 45.2 | 45.1 | EGFR | NA | NONE | SPLICE_SITE_ | NA |
| AA957 | chr7 | 55214348 | rs2072454 | C | T | 45.85 | 43.73 | EGFR | NA | NONE | SPLICE_SITE_ACCEPTOR | |
| AA1247 | chr7 | 55214348 | rs2072454 | C | T | 51.16 | 43.64 | EGFR | NA | NONE | SPLICE_SITE_ACCEPTOR | |

**Table S8: List of non-silent germline variants**

| Sample | Chr | Cord | rsID | Ref Allele | Alt Allele | Allelic Fraction | | Gene | AA change | Type | Effect | Mutation Taster Prediction |
|---|---|---|---|---|---|---|---|---|---|---|---|---|
| | | | | | | Normal | Tumor | | | | | |
| UCI2076630 | chr7 | 55229255 | rs2227983 | G | A | 57.79 | 58.63 | EGFR | R315K | MISSENSE | NON_SYNON | N |
| UCI1804937 | chr7 | 55214348 | rs2072454 | C | T | 46.47 | 43.15 | EGFR | NA | NONE | SPLICE_SITE_ACCEPTOR | |
| AA952 | chr7 | 55214348 | rs2072454 | C | T | 56.13 | 42.31 | EGFR | NA | NONE | SPLICE_SITE_ACCEPTOR | |
| UCI2219330 | chr7 | 55229255 | rs2227983 | G | A | 53.58 | 53.89 | EGFR | R315K | MISSENSE | NON_SYNON | N |
| UCI1804937 | chr7 | 140481383 | . | CT | C | 23.36 | 25.83 | BRAF | NA | NONE | FRAME_SHIF | NA |
| UCI1804937 | chr7 | 55223636 | . | A | C | 25.69 | 29.4 | EGFR | K129Q | MISSENSE | NON_SYNON | D |
| UCI1689380 | chr7 | 55214348 | rs2072454 | C | T | 47.24 | 29.24 | EGFR | NA | NONE | SPLICE_SITE_ACCEPTOR | |
| UCI1821517 | chr7 | 55214348 | rs2072454 | C | T | 42.77 | 27.13 | EGFR | NA | NONE | SPLICE_SITE_ACCEPTOR | |
| UCI2649875 | chr7 | 55229255 | rs2227983 | G | A | 47.93 | 63.59 | EGFR | R315K | MISSENSE | NON_SYNON | N |
| UCI3564897 | chr7 | 55229255 | rs2227983 | G | A | 47.49 | 37.08 | EGFR | R315K | MISSENSE | NON_SYNON | N |
| UCI4216548 | chr7 | 55229255 | rs2227983 | G | A | 48.25 | 92.38 | EGFR | R315K | MISSENSE | NON_SYNON | N |
| UCI8965412 | chr7 | 55229255 | rs2227983 | G | A | 49.66 | 46.37 | EGFR | R315K | MISSENSE | NON_SYNON | N |
| UCI9135402 | chr7 | 55238227 | rs10258568 | C | T | 48.74 | 69.88 | EGFR | S703F | MISSENSE | NON_SYNON | N |
| AA1025 | chr17 | 37884037 | rs61552325 | C | G | 48.64 | 35.13 | ERBB2 | P1140A | MISSENSE | NON_SYNON | P |
| AA1077 | chr17 | 37884037 | rs61552325 | C | G | 58.22 | 22.31 | ERBB2 | P1140A | MISSENSE | NON_SYNON | P |
| AA1090 | chr17 | 37884037 | rs61552325 | C | G | 99.83 | 99.63 | ERBB2 | P1140A | MISSENSE | NON_SYNON | P |
| AA943 | chr17 | 37884037 | rs61552325 | C | G | 57.08 | 53.89 | ERBB2 | P1140A | MISSENSE | NON_SYNON | P |
| AA1188 | chr17 | 37884037 | rs61552325 | C | G | 99.85 | 99.94 | ERBB2 | P1140A | MISSENSE | NON_SYNON | P |
| UCI1821517 | chr17 | 37879588 | rs1136201 | A | G | 97.02 | 97.4 | ERBB2 | I379V | MISSENSE | NON_SYNON | D |
| UCI8965412 | chr17 | 37879588 | rs1136201 | A | G | 52.19 | 95.5 | ERBB2 | I379V | MISSENSE | NON_SYNON | D |
| AA1247 | chr17 | 37855834 | rs4252596 | C | A | 45.24 | 94.81 | ERBB2 | P8T | MISSENSE | NON_SYNON | NA |
| UCI2649875 | chr17 | 37855834 | rs4252596 | C | A | 95.49 | 94.34 | ERBB2 | P8T | MISSENSE | NON_SYNON | NA |
| AA1204 | chr17 | 37884037 | rs61552325 | C | G | 46.8 | 15.08 | ERBB2 | P1140A | MISSENSE | NON_SYNON | P |
| AA1222 | chr17 | 37879588 | rs1136201 | A | G | 51.36 | 92.33 | ERBB2 | I379V | MISSENSE | NON_SYNON | D |
| AA1222 | chr17 | 37884037 | rs61552325 | C | G | 99.67 | 99.83 | ERBB2 | P1140A | MISSENSE | NON_SYNON | P |
| UCI3165498 | chr17 | 37855834 | rs4252596 | C | A | 51.28 | 86.35 | ERBB2 | P8T | MISSENSE | NON_SYNON | NA |
| AA1247 | chr17 | 37884037 | rs61552325 | C | G | 51.93 | 96.2 | ERBB2 | P1140A | MISSENSE | NON_SYNON | P |
| AA960 | chr17 | 37879588 | rs1136201 | A | G | 52.54 | 78.4 | ERBB2 | I379V | MISSENSE | NON_SYNON | D |
| AA1267 | chr17 | 37872589 | . | C | T | 23.75 | 2.45 | ERBB2 | R241* | NONSENSE | STOP_GAINE | A |
| AA1267 | chr17 | 37884037 | rs61552325 | C | G | 99.91 | 99.74 | ERBB2 | P1140A | MISSENSE | NON_SYNON | P |
| AA1277 | chr17 | 37884037 | rs61552325 | C | G | 52.24 | 95.93 | ERBB2 | P1140A | MISSENSE | NON_SYNON | P |
| AA1106 | chr17 | 37879588 | rs1136201 | A | G | 51.63 | 58.36 | ERBB2 | I379V | MISSENSE | NON_SYNON | D |
| UCI1546879 | chr17 | 37879588 | rs1136201 | A | G | 50.6 | 58.31 | ERBB2 | I379V | MISSENSE | NON_SYNON | D |
| AA926 | chr17 | 37884037 | rs61552325 | C | G | 99.94 | 99.52 | ERBB2 | P1140A | MISSENSE | NON_SYNON | P |
| AA1515 | chr17 | 37855834 | rs4252596 | C | A | 53.28 | 56.17 | ERBB2 | P8T | MISSENSE | NON_SYNON | NA |
| AA930 | chr17 | 37884037 | rs61552325 | C | G | 50.29 | 47.57 | ERBB2 | P1140A | MISSENSE | NON_SYNON | P |
| AA930 | chr17 | 37884070 | . | G | A | 49.96 | 47.38 | ERBB2 | V1151I | MISSENSE | NON_SYNON | N |
| AA1106 | chr17 | 37884037 | rs61552325 | C | G | 47.33 | 61.23 | ERBB2 | P1140A | MISSENSE | NON_SYNON | P |
| AA948 | chr17 | 37884037 | rs61552325 | C | G | 50.62 | 51.51 | ERBB2 | P1140A | MISSENSE | NON_SYNON | P |
| UCI1804937 | chr17 | 37855834 | rs4252596 | C | A | 41.34 | 52.48 | ERBB2 | P8T | MISSENSE | NON_SYNON | NA |
| AA952 | chr17 | 37884037 | rs61552325 | C | G | 99.79 | 98.66 | ERBB2 | P1140A | MISSENSE | NON_SYNON | P |
| AA926 | chr17 | 37855834 | rs4252596 | C | A | 51.02 | 52.25 | ERBB2 | P8T | MISSENSE | NON_SYNON | NA |
| AA1267 | chr17 | 37855834 | rs4252596 | C | A | 46.64 | 51.83 | ERBB2 | P8T | MISSENSE | NON_SYNON | NA |
| AA1367 | chr17 | 37884037 | rs61552325 | C | G | 99.82 | 99.61 | ERBB2 | P1140A | MISSENSE | NON_SYNON | P |
| AA960 | chr17 | 37884037 | rs61552325 | C | G | 49.07 | 83.65 | ERBB2 | P1140A | MISSENSE | NON_SYNON | P |
| AA1188 | chr17 | 37879588 | rs1136201 | A | G | 48.97 | 50.3 | ERBB2 | I379V | MISSENSE | NON_SYNON | D |
| AA1515 | chr17 | 37884037 | rs61552325 | C | G | 99.68 | 99.64 | ERBB2 | P1140A | MISSENSE | NON_SYNON | P |
| AA1267 | chr17 | 37879588 | rs1136201 | A | G | 48.02 | 49.39 | ERBB2 | I379V | MISSENSE | NON_SYNON | D |
| UCI1546879 | chr17 | 37884037 | rs61552325 | C | G | 99.9 | 99.75 | ERBB2 | P1140A | MISSENSE | NON_SYNON | P |
| UCI1689380 | chr17 | 37884037 | rs61552325 | C | G | 50.64 | 44.94 | ERBB2 | P1140A | MISSENSE | NON_SYNON | P |
| UCI9135402 | chr17 | 37884176 | rs55943169 | C | A | 46.3 | 48.18 | ERBB2 | A1186D | MISSENSE | NON_SYNON | D |
| AA1188 | chr17 | 37855834 | rs4252596 | C | A | 50.81 | 48.08 | ERBB2 | P8T | MISSENSE | NON_SYNON | NA |
| UCI1804937 | chr17 | 37884037 | rs61552325 | C | G | 47.36 | 63.41 | ERBB2 | P1140A | MISSENSE | NON_SYNON | P |
| UCI1821517 | chr17 | 37884037 | rs61552325 | C | G | 54.64 | 27.07 | ERBB2 | P1140A | MISSENSE | NON_SYNON | P |
| AA930 | chr17 | 37855834 | rs4252596 | C | A | 46.46 | 46.74 | ERBB2 | P8T | MISSENSE | NON_SYNON | NA |
| UCI9135402 | chr17 | 37879588 | rs1136201 | A | G | 54.95 | 46.14 | ERBB2 | I379V | MISSENSE | NON_SYNON | D |
| AA957 | chr17 | 37879588 | rs1136201 | A | G | 54.25 | 46.1 | ERBB2 | I379V | MISSENSE | NON_SYNON | D |
| UCI1951813 | chr17 | 37884037 | rs61552325 | C | G | 53.69 | 53.72 | ERBB2 | P1140A | MISSENSE | NON_SYNON | P |
| UCI2231576 | chr17 | 37855834 | rs4252596 | C | A | 44.96 | 45.28 | ERBB2 | P8T | MISSENSE | NON_SYNON | NA |
| AA948 | chr17 | 37855834 | rs4252596 | C | A | 44.04 | 44.94 | ERBB2 | P8T | MISSENSE | NON_SYNON | NA |
| UCI2008866 | chr17 | 37884037 | rs61552325 | C | G | 99.87 | 99.71 | ERBB2 | P1140A | MISSENSE | NON_SYNON | P |
| UCI2076630 | chr17 | 37855834 | rs4252596 | C | A | 41.68 | 43.29 | ERBB2 | P8T | MISSENSE | NON_SYNON | NA |
| UCI2076630 | chr17 | 37884037 | rs61552325 | C | G | 99.79 | 99.71 | ERBB2 | P1140A | MISSENSE | NON_SYNON | P |
| AA943 | chr17 | 37879588 | rs1136201 | A | G | 48.67 | 41.78 | ERBB2 | I379V | MISSENSE | NON_SYNON | D |
| UCI2219330 | chr17 | 37884037 | rs61552325 | C | G | 52.16 | 51.99 | ERBB2 | P1140A | MISSENSE | NON_SYNON | P |
| UCI1689380 | chr17 | 37879588 | rs1136201 | A | G | 52.07 | 40.2 | ERBB2 | I379V | MISSENSE | NON_SYNON | D |
| UCI1546879 | chr17 | 37855834 | rs4252596 | C | A | 48.24 | 38.62 | ERBB2 | P8T | MISSENSE | NON_SYNON | NA |
| UCI2224680 | chr17 | 37884037 | rs61552325 | C | G | 99.64 | 99.5 | ERBB2 | P1140A | MISSENSE | NON_SYNON | P |
| AA1025 | chr17 | 37855834 | rs4252596 | C | A | 53.14 | 35.16 | ERBB2 | P8T | MISSENSE | NON_SYNON | NA |
| UCI2231576 | chr17 | 37884037 | rs61552325 | C | G | 57.2 | 49.22 | ERBB2 | P1140A | MISSENSE | NON_SYNON | P |
| UCI1821517 | chr17 | 37884176 | rs55943169 | C | A | 42.72 | 25.95 | ERBB2 | A1186D | MISSENSE | NON_SYNON | D |
| AA1077 | chr17 | 37879588 | rs1136201 | A | G | 55.01 | 23.24 | ERBB2 | I379V | MISSENSE | NON_SYNON | D |
| UCI2649875 | chr17 | 37884037 | rs61552325 | C | G | 98.01 | 99.02 | ERBB2 | P1140A | MISSENSE | NON_SYNON | P |
| AA1307 | chr17 | 37879588 | rs1136201 | A | G | 46.85 | 19.79 | ERBB2 | I379V | MISSENSE | NON_SYNON | D |
| UCI3165498 | chr17 | 37884037 | rs61552325 | C | G | 99.87 | 99.86 | ERBB2 | P1140A | MISSENSE | NON_SYNON | P |
| UCI3564897 | chr17 | 37884037 | rs61552325 | C | G | 99.14 | 100 | ERBB2 | P1140A | MISSENSE | NON_SYNON | P |
| AA1090 | chr17 | 37879588 | rs1136201 | A | G | 48.91 | 13.64 | ERBB2 | I379V | MISSENSE | NON_SYNON | D |
| UCI8965412 | chr17 | 37884037 | rs61552325 | C | G | 51.25 | 94.65 | ERBB2 | P1140A | MISSENSE | NON_SYNON | P |
| UCI9135402 | chr17 | 37884037 | rs61552325 | C | G | 49.81 | 47.18 | ERBB2 | P1140A | MISSENSE | NON_SYNON | P |
| AA1222 | chr17 | 37855834 | rs4252596 | C | A | 47.87 | 7.86 | ERBB2 | P8T | MISSENSE | NON_SYNON | NA |
| UCI1689380 | chr12 | 56494998 | rs773123 | A | T | 99.28 | 99.33 | ERBB3 | S1060C | MISSENSE | NON_SYNON | D |
| UCI2008866 | chr12 | 56494998 | rs773123 | A | T | 53.41 | 89.18 | ERBB3 | S1060C | MISSENSE | NON_SYNON | D |
| UCI3564897 | chr12 | 56494998 | rs773123 | A | T | 46.53 | 88.63 | ERBB3 | S1060C | MISSENSE | NON_SYNON | D |
| UCI3165498 | chr12 | 56494998 | rs773123 | A | T | 50.54 | 64.31 | ERBB3 | S1060C | MISSENSE | NON_SYNON | D |
| AA957 | chr12 | 56495339 | rs55699040 | C | A | 46.98 | 53.19 | ERBB3 | L1118I | MISSENSE | NON_SYNON | D |
| UCI2649875 | chr12 | 56494998 | rs773123 | A | T | 53.88 | 50.94 | ERBB3 | S1060C | MISSENSE | NON_SYNON | D |
| UCI2231576 | chr12 | 56494998 | rs773123 | A | T | 45.66 | 50.59 | ERBB3 | S1060C | MISSENSE | NON_SYNON | D |
| AA1267 | chr12 | 56494998 | rs773123 | A | T | 52.05 | 50.09 | ERBB3 | S1060C | MISSENSE | NON_SYNON | D |
| UCI2219330 | chr12 | 56493677 | rs56259600 | A | G | 50.79 | 47.27 | ERBB3 | K118R | MISSENSE | NON_SYNON | D |
| UCI1804937 | chr12 | 56494998 | rs773123 | A | T | 46.09 | 47.14 | ERBB3 | S1060C | MISSENSE | NON_SYNON | D |
| AA1025 | chr12 | 56494998 | rs773123 | A | T | 52.82 | 44.5 | ERBB3 | S1060C | MISSENSE | NON_SYNON | D |
| AA1106 | chr12 | 56494998 | rs773123 | A | T | 17.66 | 38.69 | ERBB3 | S1060C | MISSENSE | NON_SYNON | D |
| AA1204 | chr12 | 56494998 | rs773123 | A | T | 52.5 | 31.69 | ERBB3 | S1060C | MISSENSE | NON_SYNON | D |
| AA1247 | chr8 | 38275808 | . | C | A | 46.39 | 82.95 | FGFR1 | M365I | MISSENSE | NON_SYNON | D |
| UCI2076630 | chr8 | 38285913 | rs138489552 | G | GTCA | 48.57 | 75.33 | FGFR1 | D125VN | NONE | CODON_CHA | NA |
| UCI2564879 | chr8 | 38287238 | rs140382957 | G | A | 54.75 | 55.7 | FGFR1 | S107L | MISSENSE | NON_SYNON | D |
| UCI2219330 | chr7 | 117188715 | rs3034796 | GAAAGC | G | 23.48 | 16.42 | CFTR | NA | NONE | FRAME_SHIF | NA |
| UCI1546879 | chr8 | 38282083 | . | C | T | 15.33 | 2.22 | FGFR1 | E134K | MISSENSE | NON_SYNON | D |
| AA957 | chr10 | 123260337 | . | CA | C | 43.25 | 43.76 | FGFR2 | NA | NONE | SPLICE_SITE_ | NA |
| UCI2224680 | chr10 | 123310871 | rs755793 | A | G | 45.05 | 31.04 | FGFR2 | M186T | MISSENSE | NON_SYNON | P |

**Table S8: List of non-silent germline variants**

| Sample | Chr | Cord | rsID | Ref Allele | Alt Allele | Allelic Fraction | | Gene | AA change | Type | Effect | Mutation Taster Prediction |
|---|---|---|---|---|---|---|---|---|---|---|---|---|
| | | | | | | Normal | Tumor | | | | | |
| UCI2564879 | chr10 | 123310871 | rs755793 | A | G | 48.26 | 57.51 | FGFR2 | M186T | MISSENSE | NON_SYNON` | P |
| AA1267 | chr10 | 8100506 | rs143627754 | C | G | 55.42 | 45.93 | GATA3 | D160E | MISSENSE | NON_SYNON` | D |
| AA1025 | chr9 | 5126715 | rs142269166 | A | G | 45.85 | 63.61 | JAK2 | N1108S | MISSENSE | NON_SYNON` | D |
| AA943 | chr9 | 5126343 | rs41316003 | G | A | 50.45 | 50.54 | JAK2 | R1063H | MISSENSE | NON_SYNON` | D |
| UCI1821517 | chr9 | 5072561 | rs139504737 | G | A | 50.59 | 26.71 | JAK2 | G422S | MISSENSE | NON_SYNON` | D |
| AA943 | chr19 | 17945696 | rs3213409 | C | T | 48.03 | 48.45 | JAK3 | V722I | MISSENSE | NON_SYNON` | None |
| AA1267 | chr19 | 17943490 | . | G | A | 53.27 | 53.54 | JAK3 | R840C | MISSENSE | NON_SYNON` | None |
| AA1277 | chr19 | 17953321 | . | C | T | 50.84 | 31.16 | JAK3 | R222H | MISSENSE | NON_SYNON` | None |
| AA1090 | chr7 | 116364823 | rs34822187 | CA | C | 99.82 | 100 | MET | NA | NONE | SPLICE_SITE_ | NA |
| AA960 | chr7 | 116364823 | rs34822187 | CA | C | 99.7 | 100 | MET | NA | NONE | SPLICE_SITE_ | NA |
| AA1277 | chr7 | 116364823 | rs34822187 | CA | C | 99.91 | 99.86 | MET | NA | NONE | SPLICE_SITE_ | NA |
| AA1106 | chr7 | 116364823 | rs34822187 | CA | C | 99.84 | 99.81 | MET | NA | NONE | SPLICE_SITE_ | NA |
| UCI3165498 | chr7 | 116364823 | rs34822187 | CA | C | 100 | 99.69 | MET | NA | NONE | SPLICE_SITE_ | NA |
| UCI1689380 | chr7 | 116364823 | rs34822187 | CA | C | 99.67 | 99.68 | MET | NA | NONE | SPLICE_SITE_ | NA |
| AA1077 | chr7 | 116335811 | rs80153920 | A | T | 45.71 | 82.03 | MET | M1L | MISSENSE | NON_SYNON` | NA |
| UCI1821517 | chr7 | 116335811 | rs80153920 | A | T | 53.76 | 76.42 | MET | M1L | MISSENSE | NON_SYNON` | NA |
| UCI8965412 | chr7 | 116335811 | rs80153920 | A | T | 50.93 | 68.94 | MET | M1L | MISSENSE | NON_SYNON` | NA |
| AA1307 | chr7 | 116339642 | rs55985569 | G | T | 43.12 | 63.01 | MET | E168D | MISSENSE | NON_SYNON` | N |
| AA926 | chr7 | 116339600 | . | A | G | 49.98 | 63.77 | MET | I154M | MISSENSE | NON_SYNON` | D |
| AA1204 | chr7 | 116364823 | rs34822187 | CA | C | 47.2 | 60.56 | MET | NA | NONE | SPLICE_SITE_ | NA |
| AA1515 | chr7 | 116364823 | rs34822187 | CA | C | 53.63 | 58.35 | MET | NA | NONE | SPLICE_SITE_ | NA |
| UCI1546879 | chr7 | 116364823 | rs34822187 | CA | C | 47.17 | 56.82 | MET | NA | NONE | SPLICE_SITE_ | NA |
| UCI1908503 | chr7 | 116364823 | rs34822187 | CA | C | 51.3 | 54.44 | MET | NA | NONE | SPLICE_SITE_ | NA |
| AA957 | chr7 | 116364823 | rs34822187 | CA | C | 50.35 | 54.24 | MET | NA | NONE | SPLICE_SITE_ | NA |
| UCI1951813 | chr7 | 116364823 | rs34822187 | CA | C | 48.31 | 52.53 | MET | NA | NONE | SPLICE_SITE_ | NA |
| UCI2224680 | chr7 | 116364823 | rs34822187 | CA | C | 54.31 | 51.18 | MET | NA | NONE | SPLICE_SITE_ | NA |
| AA1188 | chr7 | 116364823 | rs34822187 | CA | C | 44.72 | 50.76 | MET | NA | NONE | SPLICE_SITE_ | NA |
| AA943 | chr7 | 116364823 | rs34822187 | CA | C | 48.73 | 50.49 | MET | NA | NONE | SPLICE_SITE_ | NA |
| AA948 | chr7 | 116364823 | rs34822187 | CA | C | 52.62 | 50.22 | MET | NA | NONE | SPLICE_SITE_ | NA |
| UCI2231576 | chr7 | 116364823 | rs34822187 | CA | C | 50.91 | 50.15 | MET | NA | NONE | SPLICE_SITE_ | NA |
| UCI2219330 | chr7 | 116411990 | rs56391007 | C | T | 54.2 | 48.74 | MET | T1010I | MISSENSE | NON_SYNON` | D |
| AA1367 | chr7 | 116436122 | . | G | C | 47.29 | 48.61 | MET | D1373H | MISSENSE | NON_SYNON` | D |
| AA930 | chr7 | 116364823 | rs34822187 | CA | C | 48.55 | 48.39 | MET | NA | NONE | SPLICE_SITE_ | NA |
| AA1367 | chr7 | 116364823 | rs34822187 | CA | C | 54.35 | 46.87 | MET | NA | NONE | SPLICE_SITE_ | NA |
| UCI2219330 | chr7 | 116364823 | rs34822187 | CA | C | 62.82 | 45.96 | MET | NA | NONE | SPLICE_SITE_ | NA |
| UCI4216548 | chr7 | 116364823 | rs34822187 | CA | C | 47.51 | 45.77 | MET | NA | NONE | SPLICE_SITE_ | NA |
| UCI1804937 | chr7 | 116340262 | rs33917957 | A | G | 48.64 | 44.95 | MET | N375S | MISSENSE | NON_SYNON` | D |
| UCI2224680 | chr7 | 116335811 | rs80153920 | A | T | 52.77 | 44.75 | MET | M1L | MISSENSE | NON_SYNON` | NA |
| AA1025 | chr7 | 116364823 | rs34822187 | CA | C | 50.61 | 41.75 | MET | NA | NONE | SPLICE_SITE_ | NA |
| AA1307 | chr7 | 116364823 | rs34822187 | CA | C | 53.04 | 39.38 | MET | NA | NONE | SPLICE_SITE_ | NA |
| UCI2649875 | chr7 | 116335811 | rs80153920 | A | T | 49.57 | 39.05 | MET | M1L | MISSENSE | NON_SYNON` | NA |
| UCI2564879 | chr7 | 116364823 | rs34822187 | CA | C | 49.12 | 38.22 | MET | NA | NONE | SPLICE_SITE_ | NA |
| AA926 | chr7 | 116364823 | rs34822187 | CA | C | 54.9 | 36.06 | MET | NA | NONE | SPLICE_SITE_ | NA |
| AA1247 | chr7 | 116364823 | rs34822187 | CA | C | 55.77 | 34.4 | MET | NA | NONE | SPLICE_SITE_ | NA |
| UCI8965412 | chr7 | 116364823 | rs34822187 | CA | C | 47.46 | 30.65 | MET | NA | NONE | SPLICE_SITE_ | NA |
| UCI1951813 | chr7 | 117188715 | rs3034796 | GAAAGC | G | 26.08 | 20.38 | CFTR | NA | NONE | FRAME_SHIF` | NA |
| UCI1821517 | chr7 | 116364823 | rs34822187 | CA | C | 45.87 | 18.78 | MET | NA | NONE | SPLICE_SITE_ | NA |
| AA1077 | chr7 | 116364823 | rs34822187 | CA | C | 49.59 | 17.71 | MET | NA | NONE | SPLICE_SITE_ | NA |
| AA1025 | chr3 | 37053568 | rs1799977 | A | G | 48.58 | 47.59 | MLH1 | I121V | MISSENSE | NON_SYNON` | P |
| AA1090 | chr3 | 37053568 | rs1799977 | A | G | 47.8 | 49.69 | MLH1 | I121V | MISSENSE | NON_SYNON` | P |
| AA1188 | chr3 | 37053568 | rs1799977 | A | G | 50.06 | 49.05 | MLH1 | I121V | MISSENSE | NON_SYNON` | P |
| AA1204 | chr3 | 37053568 | rs1799977 | A | G | 54.18 | 53.05 | MLH1 | I121V | MISSENSE | NON_SYNON` | P |
| AA1267 | chr3 | 37053568 | rs1799977 | A | G | 45.84 | 47.46 | MLH1 | I121V | MISSENSE | NON_SYNON` | P |
| AA1307 | chr3 | 37053568 | rs1799977 | A | G | 54.39 | 40.77 | MLH1 | I121V | MISSENSE | NON_SYNON` | P |
| AA926 | chr3 | 37053568 | rs1799977 | A | G | 51.54 | 42.17 | MLH1 | I121V | MISSENSE | NON_SYNON` | P |
| AA1106 | chr3 | 37053568 | rs1799977 | A | G | 53.29 | 56.87 | MLH1 | I121V | MISSENSE | NON_SYNON` | P |
| AA948 | chr3 | 37053568 | rs1799977 | A | G | 45.2 | 50.27 | MLH1 | I121V | MISSENSE | NON_SYNON` | P |
| AA952 | chr3 | 37053568 | rs1799977 | A | G | 99.67 | 98.46 | MLH1 | I121V | MISSENSE | NON_SYNON` | P |
| AA960 | chr3 | 37053568 | rs1799977 | A | G | 59.42 | 48.51 | MLH1 | I121V | MISSENSE | NON_SYNON` | P |
| AA1515 | chr3 | 37053568 | rs1799977 | A | G | 38.87 | 41.96 | MLH1 | I121V | MISSENSE | NON_SYNON` | P |
| UCI1689380 | chr3 | 37053568 | rs1799977 | A | G | 99.72 | 99.46 | MLH1 | I121V | MISSENSE | NON_SYNON` | P |
| UCI1908503 | chr3 | 37053568 | rs1799977 | A | G | 99.78 | 99.32 | MLH1 | I121V | MISSENSE | NON_SYNON` | P |
| UCI1951813 | chr3 | 37053568 | rs1799977 | A | G | 48.8 | 52.74 | MLH1 | I121V | MISSENSE | NON_SYNON` | P |
| UCI2008866 | chr3 | 37053568 | rs1799977 | A | G | 51.78 | 62.92 | MLH1 | I121V | MISSENSE | NON_SYNON` | P |
| UCI2076630 | chr3 | 37053568 | rs1799977 | A | G | 99.76 | 98.94 | MLH1 | I121V | MISSENSE | NON_SYNON` | P |
| UCI2219330 | chr3 | 37053568 | rs1799977 | A | G | 52.72 | 45.46 | MLH1 | I121V | MISSENSE | NON_SYNON` | P |
| UCI2224680 | chr3 | 37053568 | rs1799977 | A | G | 53.73 | 62.69 | MLH1 | I121V | MISSENSE | NON_SYNON` | P |
| UCI2649875 | chr3 | 37053568 | rs1799977 | A | G | 41.73 | 46.78 | MLH1 | I121V | MISSENSE | NON_SYNON` | P |
| AA930 | chr7 | 55266424 | . | GA | G | 39.25 | 38.46 | EGFR | NA | NONE | FRAME_SHIF` | NA |
| UCI4216548 | chr3 | 37053568 | rs1799977 | A | G | 59.31 | 5.83 | MLH1 | I121V | MISSENSE | NON_SYNON` | P |
| UCI8965412 | chr3 | 37053568 | rs1799977 | A | G | 48.51 | 43.51 | MLH1 | I121V | MISSENSE | NON_SYNON` | P |
| UCI2008866 | chr2 | 47739551 | rs2303424 | A | G | 100 | 99.8 | MSH2 | Q915R | MISSENSE | NON_SYNON` | NA |
| AA1367 | chr2 | 47739551 | rs2303424 | A | G | 99.78 | 99.77 | MSH2 | Q915R | MISSENSE | NON_SYNON` | NA |
| AA957 | chr2 | 47739551 | rs2303424 | A | G | 99.79 | 99.76 | MSH2 | Q915R | MISSENSE | NON_SYNON` | NA |
| AA1090 | chr2 | 47739551 | rs2303424 | A | G | 100 | 99.75 | MSH2 | Q915R | MISSENSE | NON_SYNON` | NA |
| UCI2564879 | chr2 | 47739551 | rs2303424 | A | G | 99.7 | 99.6 | MSH2 | Q915R | MISSENSE | NON_SYNON` | NA |
| UCI2008866 | chr2 | 47643457 | rs4987188 | G | A | 47.73 | 92.67 | MSH2 | G108D | MISSENSE | NON_SYNON` | D |
| UCI4216548 | chr2 | 47739551 | rs2303424 | A | G | 39.23 | 89.66 | MSH2 | Q915R | MISSENSE | NON_SYNON` | NA |
| AA960 | chr2 | 47739551 | rs2303424 | A | G | 48.48 | 68.14 | MSH2 | Q915R | | NON_SYNON` | NA |
| UCI1689380 | chr2 | 47739551 | rs2303424 | A | G | 53.65 | 65.47 | MSH2 | Q915R | MISSENSE | NON_SYNON` | NA |
| UCI2224680 | chr2 | 47739551 | rs2303424 | A | G | 42.5 | 57.51 | MSH2 | Q915R | MISSENSE | NON_SYNON` | NA |
| UCI1804937 | chr2 | 47739551 | rs2303424 | A | G | 45.67 | 56.44 | MSH2 | Q915R | MISSENSE | NON_SYNON` | NA |
| AA1222 | chr2 | 47739551 | rs2303424 | A | G | 58 | 54.4 | MSH2 | Q915R | MISSENSE | NON_SYNON` | NA |
| UCI9135402 | chr2 | 47739533 | rs116117580 | G | A | 52.52 | 52.37 | MSH2 | R909Q | MISSENSE | NON_SYNON` | NA |
| AA943 | chr2 | 47739551 | rs2303424 | A | G | 52.21 | 50.81 | MSH2 | Q915R | MISSENSE | NON_SYNON` | NA |
| AA1090 | chr2 | 47643457 | rs4987188 | G | A | 49.97 | 50.33 | MSH2 | G108D | MISSENSE | NON_SYNON` | D |
| AA1204 | chr2 | 47739551 | rs2303424 | A | G | 48.19 | 50.18 | MSH2 | Q915R | MISSENSE | NON_SYNON` | NA |
| UCI1821517 | chr2 | 47739551 | rs2303424 | A | G | 52.99 | 50.07 | MSH2 | Q915R | MISSENSE | NON_SYNON` | NA |
| AA1367 | chr2 | 47656972 | rs17224367 | C | T | 48.51 | 48.42 | MSH2 | L176F | MISSENSE | NON_SYNON` | D |
| AA926 | chr2 | 47739551 | rs2303424 | A | G | 49.53 | 47.98 | MSH2 | Q915R | MISSENSE | NON_SYNON` | NA |
| UCI2231576 | chr2 | 47635690 | . | A | G | 52.55 | 46.97 | MSH2 | I7V | MISSENSE | NON_SYNON` | D |
| UCI2649875 | chr2 | 47739551 | rs2303424 | A | G | 55.36 | 46.31 | MSH2 | Q915R | MISSENSE | NON_SYNON` | NA |
| UCI1951813 | chr2 | 47739551 | rs2303424 | A | G | 53.3 | 46.27 | MSH2 | Q915R | MISSENSE | NON_SYNON` | NA |
| UCI1546879 | chr2 | 47739533 | rs116117580 | G | A | 46.58 | 42.3 | MSH2 | R909Q | MISSENSE | NON_SYNON` | NA |
| UCI3564897 | chr2 | 47637301 | rs63750124 | T | G | 49.04 | 48.36 | MSH2 | I145M | MISSENSE | NON_SYNON` | N |
| AA1025 | chr2 | 48010488 | rs1042821 | G | A | 47.32 | 40.53 | MSH6 | G39E | MISSENSE | NON_SYNON` | N |
| AA1204 | chr2 | 48010488 | rs1042821 | G | A | 49.11 | 46.97 | MSH6 | G39E | MISSENSE | NON_SYNON` | N |
| AA1267 | chr2 | 48010488 | rs1042821 | G | A | 50.47 | 53.06 | MSH6 | G39E | MISSENSE | NON_SYNON` | N |
| AA1277 | chr2 | 48010488 | rs1042821 | G | A | 51.36 | 47.75 | MSH6 | G39E | MISSENSE | NON_SYNON` | N |
| AA1307 | chr2 | 48010488 | rs1042821 | G | A | 98.83 | 98.76 | MSH6 | G39E | MISSENSE | NON_SYNON` | N |

**Table S8: List of non-silent germline variants**

| Sample | Chr | Cord | rsID | Ref Allele | Alt Allele | Allelic Fraction | | Gene | AA change | Type | Effect | Mutation Taster Prediction |
|---|---|---|---|---|---|---|---|---|---|---|---|---|
| | | | | | | Normal | Tumor | | | | | |
| AA930 | chr2 | 48010488 | rs1042821 | G | A | 49.16 | 49.35 | MSH6 | G39E | MISSENSE | NON_SYNON | N |
| AA1106 | chr2 | 48010488 | rs1042821 | G | A | 48.88 | 49.94 | MSH6 | G39E | MISSENSE | NON_SYNON | N |
| UCI2219330 | chr2 | 48026648 | rs63751005 | T | C | 54.02 | 52.44 | MSH6 | V207A | MISSENSE | NON_SYNON | D |
| UCI9135402 | chr2 | 48018236 | rs3211299 | G | T | 49.44 | 49.25 | MSH6 | S142I | MISSENSE | NON_SYNON | D |
| AA1204 | chr2 | 48018236 | rs3211299 | G | T | 48.46 | 47.02 | MSH6 | S142I | MISSENSE | NON_SYNON | D |
| UCI1908503 | chr2 | 48010488 | rs1042821 | G | A | 47.68 | 45.73 | MSH6 | G39E | MISSENSE | NON_SYNON | N |
| UCI3564897 | chr2 | 48027755 | rs2020912 | T | C | 52.14 | 46.15 | MSH6 | V576A | MISSENSE | NON_SYNON | D |
| UCI1951813 | chr2 | 48010488 | rs1042821 | G | A | 50.88 | 45.49 | MSH6 | G39E | MISSENSE | NON_SYNON | N |
| AA1025 | chr2 | 48025785 | rs41557217 | A | C | 48.88 | 45.19 | MSH6 | E122D | MISSENSE | NON_SYNON | D |
| UCI2219330 | chr2 | 48010488 | rs1042821 | G | A | 49.94 | 50.04 | MSH6 | G39E | MISSENSE | NON_SYNON | N |
| UCI2564879 | chr2 | 48010488 | rs1042821 | G | A | 45.93 | 38.52 | MSH6 | G39E | MISSENSE | NON_SYNON | N |
| UCI3564897 | chr2 | 48010488 | rs1042821 | G | A | 98.75 | 98.85 | MSH6 | G39E | MISSENSE | NON_SYNON | N |
| UCI8965412 | chr2 | 48010488 | rs1042821 | G | A | 43.39 | 43.02 | MSH6 | G39E | MISSENSE | NON_SYNON | N |
| AA1090 | chr9 | 139401233 | rs61751543 | C | T | 99.61 | 99.8 | NOTCH1 | R1279H | MISSENSE | NON_SYNON | D |
| AA1267 | chr9 | 139400219 | rs61751542 | G | A | 47.25 | 50.16 | NOTCH1 | P1377S | MISSENSE | NON_SYNON | N |
| AA1307 | chr9 | 139400219 | rs61751542 | G | A | 45.34 | 51.8 | NOTCH1 | P1377S | MISSENSE | NON_SYNON | N |
| UCI1689380 | chr9 | 139407858 | . | C | T | 49.8 | 61.97 | NOTCH1 | R780Q | MISSENSE | NON_SYNON | D |
| UCI1546879 | chr9 | 139400219 | rs61751542 | G | A | 50.26 | 39.83 | NOTCH1 | P1377S | MISSENSE | NON_SYNON | N |
| UCI2231576 | chr9 | 139399320 | rs76371972 | C | T | 46.27 | 48.66 | NOTCH1 | R1608H | MISSENSE | NON_SYNON | D |
| UCI2224680 | chr9 | 139391338 | rs61751489 | C | T | 47.32 | 55.38 | NOTCH1 | V2285I | MISSENSE | NON_SYNON | N |
| UCI2564879 | chr9 | 139391219 | . | G | T | 52.4 | 59.37 | NOTCH1 | N2324K | MISSENSE | NON_SYNON | None |
| UCI1908503 | chr2 | 29519780 | . | CA | C | 39.49 | 39.08 | ALK | NA | NONE | FRAME_SHIF | NA |
| UCI1951813 | chr22 | 42524175 | rs5030656 | CCTT | C | 42.41 | 36.7 | CYP2D6 | K227- | NONE | CODON_DEL | NA |
| UCI1908503 | chr2 | 29451759 | . | CT | C | 43.93 | 42.01 | ALK | NA | NONE | FRAME_SHIF | NA |
| AA1204 | chr2 | 29451759 | . | CT | C | 46 | 46.83 | ALK | NA | NONE | FRAME_SHIF | NA |
| AA1025 | chr2 | 29451759 | . | CT | C | 48.54 | 48.36 | ALK | NA | NONE | FRAME_SHIF | NA |
| AA1077 | chr16 | 23646191 | rs152451 | T | C | 47.01 | 51.74 | PALB2 | Q559R | MISSENSE | NON_SYNON | N |
| AA1090 | chr16 | 23646857 | rs45494092 | A | G | 51.56 | 50.8 | PALB2 | L337S | MISSENSE | NON_SYNON | N |
| AA1188 | chr16 | 23646857 | rs45494092 | A | G | 50.23 | 45.98 | PALB2 | L337S | MISSENSE | NON_SYNON | N |
| AA1204 | chr16 | 23646191 | rs152451 | T | C | 46.88 | 48.56 | PALB2 | Q559R | MISSENSE | NON_SYNON | N |
| AA1277 | chr16 | 23646191 | rs152451 | T | C | 58.07 | 63.29 | PALB2 | Q559R | MISSENSE | NON_SYNON | N |
| AA1307 | chr16 | 23646191 | rs152451 | T | C | 48.98 | 35.78 | PALB2 | Q559R | MISSENSE | NON_SYNON | N |
| UCI2231576 | chr16 | 23634293 | rs45551636 | C | T | 50.11 | 54.81 | PALB2 | G998E | MISSENSE | NON_SYNON | D |
| AA1277 | chr17 | 41243479 | rs80357508 | CTTGA | C | 49.14 | 94.57 | BRCA1 | NA | NONE | FRAME_SHIF | NA |
| UCI1908503 | chr16 | 23635370 | rs45624036 | C | T | 33.05 | 59.23 | PALB2 | V932M | MISSENSE | NON_SYNON | N |
| UCI2008866 | chr16 | 23646191 | rs152451 | T | C | 99.66 | 99.88 | PALB2 | Q559R | MISSENSE | NON_SYNON | N |
| UCI2076630 | chr16 | 23641461 | rs45532440 | C | G | 59.13 | 44.88 | PALB2 | E672Q | MISSENSE | NON_SYNON | N |
| UCI2076630 | chr16 | 23646191 | rs152451 | T | C | 56.24 | 42.98 | PALB2 | Q559R | MISSENSE | NON_SYNON | N |
| UCI2076630 | chr16 | 23634293 | rs45551636 | C | T | 46.2 | 42.35 | PALB2 | G998E | MISSENSE | NON_SYNON | D |
| UCI2219330 | chr16 | 23646191 | rs152451 | T | C | 42.53 | 51.6 | PALB2 | Q559R | MISSENSE | NON_SYNON | N |
| UCI2231576 | chr16 | 23641461 | rs45532440 | C | G | 53.23 | 53.75 | PALB2 | E672Q | MISSENSE | NON_SYNON | N |
| UCI2231576 | chr16 | 23646191 | rs152451 | T | C | 51.75 | 47.52 | PALB2 | Q559R | MISSENSE | NON_SYNON | N |
| UCI3165498 | chr16 | 23646191 | rs152451 | T | C | 99.65 | 99.68 | PALB2 | Q559R | MISSENSE | NON_SYNON | N |
| AA1277 | chr5 | 149515397 | rs17110944 | T | A | 54.74 | 9.29 | PDGFRB | I29F | MISSENSE | NON_SYNON | P |
| AA1307 | chr5 | 149495354 | rs151236133 | C | T | 52.15 | 58.89 | PDGFRB | R1098Q | MISSENSE | NON_SYNON | N |
| AA926 | chr5 | 149497358 | rs35731372 | C | T | 51.38 | 50.47 | PDGFRB | R657Q | MISSENSE | NON_SYNON | D |
| UCI1951813 | chr5 | 149497347 | rs140261309 | G | A | 59.93 | 48.65 | PDGFRB | R661C | MISSENSE | NON_SYNON | D |
| UCI1804937 | chr5 | 149515397 | rs17110944 | T | A | 52.59 | 37.15 | PDGFRB | I29F | MISSENSE | NON_SYNON | P |
| UCI2231576 | chr5 | 149509446 | rs41287110 | C | T | 49.4 | 50.22 | PDGFRB | E155K | MISSENSE | NON_SYNON | N |
| UCI2231576 | chr22 | 42524175 | rs5030656 | CCTT | C | 51.5 | 52.49 | CYP2D6 | K227- | NONE | CODON_DEL | NA |
| AA1025 | chr3 | 178927410 | rs3729680 | A | G | 50.42 | 57.7 | PIK3CA | I391M | MISSENSE | NON_SYNON | P |
| AA1090 | chr3 | 178927410 | rs3729680 | A | G | 46.7 | 54.49 | PIK3CA | I391M | MISSENSE | NON_SYNON | P |
| AA952 | chr3 | 178927410 | rs3729680 | A | G | 45.64 | 53.97 | PIK3CA | I391M | MISSENSE | NON_SYNON | P |
| UCI1951813 | chr3 | 178927410 | rs3729680 | A | G | 58.49 | 50.45 | PIK3CA | I391M | MISSENSE | NON_SYNON | P |
| UCI3564897 | chr3 | 178927410 | rs3729680 | A | G | 52.49 | 7.11 | PIK3CA | I391M | MISSENSE | NON_SYNON | P |
| AA1025 | chr5 | 67588148 | rs3730089 | G | A | 48.3 | 67.94 | PIK3R1 | M26I | MISSENSE | NON_SYNON | P |
| AA1090 | chr5 | 67588148 | rs3730089 | G | A | 48.31 | 57.8 | PIK3R1 | M26I | MISSENSE | NON_SYNON | P |
| AA943 | chr5 | 67588148 | rs3730089 | G | A | 49.17 | 43.67 | PIK3R1 | M26I | MISSENSE | NON_SYNON | P |
| AA1188 | chr5 | 67588148 | rs3730089 | G | A | 50.44 | 47.13 | PIK3R1 | M26I | MISSENSE | NON_SYNON | P |
| AA1247 | chr5 | 67588148 | rs3730089 | G | A | 50.39 | 59.94 | PIK3R1 | M26I | MISSENSE | NON_SYNON | P |
| AA1267 | chr5 | 67588148 | rs3730089 | G | A | 51.34 | 47.99 | PIK3R1 | M26I | MISSENSE | NON_SYNON | P |
| AA1277 | chr5 | 67588148 | rs3730089 | G | A | 49.56 | 88.46 | PIK3R1 | M26I | MISSENSE | NON_SYNON | P |
| AA926 | chr5 | 67588148 | rs3730089 | G | A | 51.03 | 47.79 | PIK3R1 | M26I | MISSENSE | NON_SYNON | P |
| AA930 | chr5 | 67588148 | rs3730089 | G | A | 42.32 | 49.51 | PIK3R1 | M26I | MISSENSE | NON_SYNON | P |
| UCI1951813 | chr5 | 67588148 | rs3730089 | G | A | 40.93 | 44.23 | PIK3R1 | M26I | MISSENSE | NON_SYNON | P |
| UCI2076630 | chr5 | 67588148 | rs3730089 | G | A | 50.6 | 34.71 | PIK3R1 | M26I | MISSENSE | NON_SYNON | P |
| UCI2219330 | chr5 | 67588148 | rs3730089 | G | A | 43.51 | 44.24 | PIK3R1 | M26I | MISSENSE | NON_SYNON | P |
| UCI2564879 | chr5 | 67588148 | rs3730089 | G | A | 52.3 | 52.28 | PIK3R1 | M26I | MISSENSE | NON_SYNON | P |
| UCI8965412 | chr5 | 67588148 | rs3730089 | G | A | 55.19 | 40.09 | PIK3R1 | M26I | MISSENSE | NON_SYNON | P |
| UCI1689380 | chr5 | 67593246 | rs114163757 | C | G | 51.24 | 6.55 | PIK3R1 | D301E | MISSENSE | NON_SYNON | D |
| AA1025 | chr7 | 6026988 | rs1805321 | G | A | 99.22 | 98.53 | PMS2 | P364S | MISSENSE | NON_SYNON | N |
| AA1090 | chr7 | 6026988 | rs1805321 | G | A | 49.21 | 83.05 | PMS2 | P364S | MISSENSE | NON_SYNON | N |
| AA943 | chr7 | 6026988 | rs1805321 | G | A | 51.2 | 51.53 | PMS2 | P364S | MISSENSE | NON_SYNON | N |
| AA943 | chr7 | 6045627 | rs10254120 | C | T | 47.29 | 50.4 | PMS2 | R20Q | MISSENSE | NON_SYNON | N |
| AA1188 | chr7 | 6026988 | rs1805321 | G | A | 52.02 | 49.46 | PMS2 | P364S | MISSENSE | NON_SYNON | N |
| AA1204 | chr7 | 6026942 | rs1805323 | G | T | 47.61 | 38.83 | PMS2 | T379K | MISSENSE | NON_SYNON | N |
| AA1204 | chr7 | 6045627 | rs10254120 | C | T | 51.38 | 57.01 | PMS2 | R20Q | MISSENSE | NON_SYNON | N |
| AA1222 | chr7 | 6026942 | rs1805323 | G | T | 53.97 | 51.62 | PMS2 | T379K | MISSENSE | NON_SYNON | N |
| AA1247 | chr7 | 6026988 | rs1805321 | G | A | 98.71 | 98.43 | PMS2 | P364S | MISSENSE | NON_SYNON | N |
| AA1267 | chr7 | 6026708 | rs63750668 | C | A | 42.49 | 46.15 | PMS2 | R457L | MISSENSE | NON_SYNON | N |
| AA1267 | chr7 | 6026988 | rs1805321 | G | A | 48.8 | 48.73 | PMS2 | P364S | MISSENSE | NON_SYNON | N |
| AA1277 | chr7 | 6026942 | rs1805323 | G | T | 45.49 | 83.37 | PMS2 | T379K | MISSENSE | NON_SYNON | N |
| AA1277 | chr7 | 6045627 | rs10254120 | C | T | 50.78 | 11.63 | PMS2 | R20Q | MISSENSE | NON_SYNON | N |
| AA1367 | chr7 | 6029571 | . | T | C | 51.88 | 61.95 | PMS2 | N229S | MISSENSE | NON_SYNON | D |
| AA957 | chr7 | 6026942 | rs1805323 | G | T | 99.04 | 99.09 | PMS2 | T379K | MISSENSE | NON_SYNON | N |
| AA926 | chr7 | 6026988 | rs1805321 | G | A | 39.91 | 59.4 | PMS2 | P364S | MISSENSE | NON_SYNON | N |
| AA930 | chr7 | 6026530 | rs1805324 | C | T | 53.47 | 49.05 | PMS2 | M516I | MISSENSE | NON_SYNON | N |
| AA1106 | chr7 | 6026942 | rs1805323 | G | T | 42.28 | 18.4 | PMS2 | T379K | MISSENSE | NON_SYNON | N |
| AA1106 | chr7 | 6026988 | rs1805321 | G | A | 43.83 | 41.8 | PMS2 | P364S | MISSENSE | NON_SYNON | N |
| AA948 | chr7 | 6026988 | rs1805321 | G | A | 50.59 | 40.49 | PMS2 | P364S | MISSENSE | NON_SYNON | N |
| AA948 | chr7 | 6045627 | rs10254120 | C | T | 49.12 | 50.89 | PMS2 | R20Q | MISSENSE | NON_SYNON | N |
| AA1367 | chr7 | 6026988 | rs1805321 | G | A | 50.61 | 54.41 | PMS2 | P364S | MISSENSE | NON_SYNON | N |
| AA960 | chr7 | 6026988 | rs1805321 | G | A | 46.44 | 49.21 | PMS2 | P364S | MISSENSE | NON_SYNON | N |
| AA1515 | chr7 | 6026988 | rs1805321 | G | A | 43.76 | 32.54 | PMS2 | P364S | MISSENSE | NON_SYNON | N |
| UCI1546879 | chr7 | 6026988 | rs1805321 | G | A | 98.55 | 98.5 | PMS2 | P364S | MISSENSE | NON_SYNON | N |
| UCI1689380 | chr7 | 6026942 | rs1805323 | G | T | 50.64 | 23.56 | PMS2 | T379K | MISSENSE | NON_SYNON | N |
| UCI1804937 | chr7 | 6026942 | rs1805323 | G | T | 44.69 | 44.56 | PMS2 | T379K | MISSENSE | NON_SYNON | N |
| AA952 | chr7 | 6045634 | rs63750123 | T | C | 43.72 | 47.96 | PMS2 | I18V | MISSENSE | NON_SYNON | D |
| UCI1821517 | chr7 | 6026988 | rs1805321 | G | A | 45.3 | 19.44 | PMS2 | P364S | MISSENSE | NON_SYNON | N |
| UCI1908503 | chr7 | 6026865 | rs2228007 | T | C | 53.19 | 57.81 | PMS2 | T405A | MISSENSE | NON_SYNON | N |

**Table S8: List of non-silent germline variants**

| Sample | Chr | Cord | rsID | Ref Allele | Alt Allele | Allelic Fraction | | Gene | AA change | Type | Effect | Mutation Taster Prediction |
|---|---|---|---|---|---|---|---|---|---|---|---|---|
| | | | | | | Normal | Tumor | | | | | |
| UCI1951813 | chr7 | 6026607 | rs1805318 | T | A | 52.73 | 54.34 | PMS2 | T491S | MISSENSE | NON_SYNON | N |
| UCI1951813 | chr7 | 6026988 | rs1805321 | G | A | 39.28 | 40.8 | PMS2 | P364S | MISSENSE | NON_SYNON | N |
| UCI2076630 | chr7 | 6026942 | rs1805323 | G | T | 53.01 | 37.03 | PMS2 | T379K | MISSENSE | NON_SYNON | N |
| UCI2224680 | chr7 | 6026988 | rs1805321 | G | A | 48.62 | 44.58 | PMS2 | P364S | MISSENSE | NON_SYNON | N |
| UCI2231576 | chr7 | 6026988 | rs1805321 | G | A | 97.81 | 99.34 | PMS2 | P364S | MISSENSE | NON_SYNON | N |
| UCI2564879 | chr7 | 6026942 | rs1805323 | G | T | 50.11 | 59.79 | PMS2 | T379K | MISSENSE | NON_SYNON | N |
| UCI2564879 | chr7 | 6045627 | rs10254120 | C | T | 53.21 | 33.11 | PMS2 | R20Q | MISSENSE | NON_SYNON | N |
| UCI2649875 | chr7 | 6026988 | rs1805321 | G | A | 43.24 | 59.2 | PMS2 | P364S | MISSENSE | NON_SYNON | N |
| UCI3165498 | chr7 | 6026988 | rs1805321 | G | A | 48.1 | 48.54 | PMS2 | P364S | MISSENSE | NON_SYNON | N |
| AA1307 | chr22 | 42524243 | rs35742686 | CT | C | 52.02 | 48.38 | CYP2D6 | NA | NONE | FRAME_SHIF | NA |
| UCI3564897 | chr7 | 6026988 | rs1805321 | G | A | 99.12 | 99.14 | PMS2 | P364S | MISSENSE | NON_SYNON | N |
| UCI1689380 | chr16 | 23619289 | . | ACT | A | 53.98 | 46.5 | PALB2 | E1082fs | NONE | FRAME_SHIF | NA |
| UCI8965412 | chr7 | 6026988 | rs1805321 | G | A | 98.2 | 99.21 | PMS2 | P364S | MISSENSE | NON_SYNON | N |
| UCI9135402 | chr7 | 6026988 | rs1805321 | G | A | 41.23 | 57.86 | PMS2 | P364S | MISSENSE | NON_SYNON | N |
| AA1077 | chr17 | 38512480 | rs2229773 | C | T | 43.75 | 67.7 | RARA | R456C | MISSENSE | NON_SYNON | NA |
| AA1025 | chr10 | 43595999 | rs145633958 | C | A | 51.99 | 51.04 | RET | L56M | MISSENSE | NON_SYNON | N |
| AA1090 | chr10 | 43610119 | rs1799939 | G | A | 50.94 | 52.84 | RET | G691S | MISSENSE | NON_SYNON | P |
| AA1307 | chr10 | 43610119 | rs1799939 | G | A | 54.07 | 79.21 | RET | G691S | MISSENSE | NON_SYNON | P |
| AA957 | chr10 | 43610119 | rs1799939 | G | A | 55.76 | 52.14 | RET | G691S | MISSENSE | NON_SYNON | P |
| AA952 | chr10 | 43610119 | rs1799939 | G | A | 59.37 | 50.71 | RET | G691S | MISSENSE | NON_SYNON | P |
| UCI1546879 | chr10 | 43610119 | rs1799939 | G | A | 47.28 | 57.23 | RET | G691S | MISSENSE | NON_SYNON | P |
| UCI1821517 | chr10 | 43610119 | rs1799939 | G | A | 42.51 | 52 | RET | G691S | MISSENSE | NON_SYNON | P |
| UCI1908503 | chr10 | 43610119 | rs1799939 | G | A | 94.98 | 96.12 | RET | G691S | MISSENSE | NON_SYNON | P |
| UCI2224680 | chr10 | 43610119 | rs1799939 | G | A | 54.42 | 50.43 | RET | G691S | MISSENSE | NON_SYNON | P |
| UCI2564879 | chr10 | 43610119 | rs1799939 | G | A | 49.82 | 44.45 | RET | G691S | MISSENSE | NON_SYNON | P |
| UCI3564897 | chr10 | 43610119 | rs1799939 | G | A | 99.51 | 99.29 | RET | G691S | MISSENSE | NON_SYNON | P |
| UCI3564897 | chr10 | 43620335 | rs17158558 | C | T | 50.79 | 32.14 | RET | R982C | MISSENSE | NON_SYNON | P |
| UCI4216548 | chr10 | 43610119 | rs1799939 | G | A | 44.22 | 47.64 | RET | G691S | MISSENSE | NON_SYNON | P |
| UCI4216548 | chr10 | 43620335 | rs17158558 | C | T | 48.88 | 46.29 | RET | R982C | MISSENSE | NON_SYNON | P |
| UCI9135402 | chr10 | 43610119 | rs1799939 | G | A | 56.33 | 69.69 | RET | G691S | MISSENSE | NON_SYNON | P |
| AA1025 | chr17 | 7579472 | rs1042522 | G | C | 47.54 | 35.9 | TP53 | P72R | MISSENSE | NON_SYNON | P |
| AA1077 | chr17 | 7579472 | rs1042522 | G | C | 99.62 | 99.73 | TP53 | P72R | MISSENSE | NON_SYNON | P |
| AA1090 | chr17 | 7579472 | rs1042522 | G | C | 99.68 | 99.32 | TP53 | P72R | MISSENSE | NON_SYNON | P |
| AA943 | chr17 | 7579472 | rs1042522 | G | C | 99.29 | 99.27 | TP53 | P72R | MISSENSE | NON_SYNON | P |
| AA1188 | chr17 | 7579472 | rs1042522 | G | C | 99.37 | 99.56 | TP53 | P72R | MISSENSE | NON_SYNON | P |
| AA1204 | chr17 | 7579472 | rs1042522 | G | C | 99.67 | 99.83 | TP53 | P72R | MISSENSE | NON_SYNON | P |
| AA1222 | chr17 | 7579472 | rs1042522 | G | C | 50.35 | 26.09 | TP53 | P72R | MISSENSE | NON_SYNON | P |
| AA1247 | chr17 | 7579472 | rs1042522 | G | C | 49.16 | 87.85 | TP53 | P72R | MISSENSE | NON_SYNON | P |
| AA1267 | chr17 | 7579472 | rs1042522 | G | C | 99.26 | 99.42 | TP53 | P72R | MISSENSE | NON_SYNON | P |
| AA960 | chr17 | 7578146 | rs34949160 | T | C | 50.2 | 71.17 | TP53 | R89G | MISSENSE | NON_SYNONYMOUS_CODING | |
| UCI2219330 | chr17 | 7578146 | rs34949160 | T | C | 42.59 | 70.49 | TP53 | R89G | MISSENSE | NON_SYNONYMOUS_CODING | |
| AA1277 | chr17 | 7579472 | rs1042522 | G | C | 51 | 7.27 | TP53 | P72R | MISSENSE | NON_SYNON | P |
| AA1307 | chr17 | 7579472 | rs1042522 | G | C | 99.4 | 99.48 | TP53 | P72R | MISSENSE | NON_SYNON | P |
| AA957 | chr17 | 7579472 | rs1042522 | G | C | 99.21 | 99 | TP53 | P72R | MISSENSE | NON_SYNON | P |
| AA926 | chr17 | 7579472 | rs1042522 | G | C | 52.15 | 55.16 | TP53 | P72R | MISSENSE | NON_SYNON | P |
| AA930 | chr17 | 7579472 | rs1042522 | G | C | 51.98 | 51.78 | TP53 | P72R | MISSENSE | NON_SYNON | P |
| AA948 | chr17 | 7579472 | rs1042522 | G | C | 99.8 | 99.81 | TP53 | P72R | MISSENSE | NON_SYNON | P |
| AA952 | chr17 | 7579472 | rs1042522 | G | C | 99.41 | 99.69 | TP53 | P72R | MISSENSE | NON_SYNON | P |
| AA1367 | chr17 | 7579472 | rs1042522 | G | C | 99.3 | 98.83 | TP53 | P72R | MISSENSE | NON_SYNON | P |
| AA960 | chr17 | 7579472 | rs1042522 | G | C | 47.12 | 16.84 | TP53 | P72R | MISSENSE | NON_SYNON | P |
| AA1515 | chr17 | 7579472 | rs1042522 | G | C | 52.78 | 50.98 | TP53 | P72R | MISSENSE | NON_SYNON | P |
| UCI1546879 | chr17 | 7579472 | rs1042522 | G | C | 47.12 | 41.31 | TP53 | P72R | MISSENSE | NON_SYNON | P |
| UCI1689380 | chr17 | 7579472 | rs1042522 | G | C | 99.55 | 99.45 | TP53 | P72R | MISSENSE | NON_SYNON | P |
| UCI1821517 | chr17 | 7579472 | rs1042522 | G | C | 44.83 | 25.58 | TP53 | P72R | MISSENSE | NON_SYNON | P |
| UCI2008866 | chr17 | 7579472 | rs1042522 | G | C | 99.35 | 99.39 | TP53 | P72R | MISSENSE | NON_SYNON | P |
| UCI2076630 | chr17 | 7579472 | rs1042522 | G | C | 51.26 | 14.78 | TP53 | P72R | MISSENSE | NON_SYNON | P |
| UCI2219330 | chr17 | 7579472 | rs1042522 | G | C | 47.78 | 30.92 | TP53 | P72R | MISSENSE | NON_SYNON | P |
| UCI2224680 | chr17 | 7579472 | rs1042522 | G | C | 99.55 | 99.04 | TP53 | P72R | MISSENSE | NON_SYNON | P |
| UCI2231576 | chr17 | 7579472 | rs1042522 | G | C | 96.6 | 99.33 | TP53 | P72R | MISSENSE | NON_SYNON | P |
| UCI2649875 | chr17 | 7579472 | rs1042522 | G | C | 97.34 | 97.23 | TP53 | P72R | MISSENSE | NON_SYNON | P |
| UCI3165498 | chr17 | 7579472 | rs1042522 | G | C | 49.92 | 87.05 | TP53 | P72R | MISSENSE | NON_SYNON | P |
| UCI3564897 | chr17 | 7579472 | rs1042522 | G | C | 99.68 | 99.67 | TP53 | P72R | MISSENSE | NON_SYNON | P |
| UCI8965412 | chr17 | 7579472 | rs1042522 | G | C | 99.59 | 99.56 | TP53 | P72R | MISSENSE | NON_SYNON | P |
| UCI9135402 | chr17 | 7579472 | rs1042522 | G | C | 97.15 | 96.46 | TP53 | P72R | MISSENSE | NON_SYNON | P |
| UCI9135402 | chr6 | 18139228 | rs1800460 | C | T | 52.39 | 50.13 | TPMT | A154T | MISSENSE | NON_SYNON | D |
| UCI9135402 | chr6 | 18130918 | rs1142345 | T | C | 53.78 | 44.31 | TPMT | Y240C | MISSENSE | NON_SYNON | D |

**Table S9: list of predicted deleterious inherited variants in BRCA1 or BRCA2**

| | | | | | Allelic Fraction | |
|---|---|---|---|---|---|---|
| **Sample** | **rsID** | **Gene** | **AA change** | **Type** | **Normal** | **Tumor** |
| AA1277 | rs80357508 | BRCA1 | Q1355_E1356fs | FRAMESHIFT | 49.14 | 94.57 |
| AA1515 | rs80357087 | BRCA1 | A1373S | MISSENSE | 51.47 | 52 |
| UCI2008866 | rs11571833 | BRCA2 | K3326* | NONSENSE | 45.99 | 12.16 |
