# Supplementary material for: Evaluation of ultra-deep targeted sequencing for personalized breast cancer care
Source: Breast Cancer Res. 2013 Dec 10;15(6):R115. doi: 10.1186/bcr3584 (PMC3978701; doi:10.1186/bcr3584)
Supplement: Additional file 2 — Supplemental text. [file bcr3584-S2.pdf]

Supplemental Text

Clinical Specimen

<u>Collection:</u> All subjects provided informed consent with written documentation prior to sample collection or assay. Remnant tissue samples were taken for research use at the time of clinically indicated surgeries under the direct supervision of licensed members of the pathology department at the respective institutions. Each patient who consented to donating a tissue sample was also requested to give either a blood or saliva sample. Blood was obtained by venipuncture of the median cubital vein into an uncoated serum tube and allowed to clot at room temperature. Saliva was collected in an Oragene saliva kit (OGR-500) (DNA Genotek, Ottawa, Canada). Detailed standard pathologic characteristics are provided in Table S1. ASCO/CAP guidelines [1, 2] were followed for evaluation of estrogen receptor, progesterone receptor and Her2. The cellularity of the frozen sample was estimated as the average cellularity of the tumor tissue from two adjacent H&E stained paraffin-embedded sections. Most specimens were collected from untreated primary tumors but our cohort included patients with regional or distant disease treated with neoadjuvant therapy prior to surgery or biopsy.

<u>Description</u>: We included 38 patients from our breast cancer clinics for which frozen tissue specimen was available from the biorepository and properly consented for genetic analysis (Table S1). Three specimens were from distant sites (lymph node, liver and brain metastasis), two were from primary invasive lobular carcinomas, and 33 were from primary invasive ductal carcinoma including 5 with mixed ductal/lobular features. The majority were diagnosed with Nottingham histologic grade II (N=16) and grade III (N=12). All specimens underwent routine molecular testing for receptor status (ER and PR using immunohistochemical staining). Her2 status was assayed using either immunostaining (N=19), in situ hybridization (N=13), or both (N=5), resulting in the identification of 6 specimens that were Her2-overexpressed by ASCO/CAP standards. Histological inspection revealed a broad range of cellularity (Figure 1). More than half of the tumors contained fewer than 60% malignant cells in the section studied for diagnosis. These samples would have failed criteria for inclusion in most cancer genomic studies such as the TCGA study. Ten samples had less than 40% tumor cells, and therefore failed the criteria for analysis by classical Sanger sequencing. Finally, 4 tumors had less than 20% cellularity failing to meet the criteria for genetic profiling at most diagnostic laboratories, including the ones using next generation sequencing. We

anticipated that the sensitivity of our assay would overcome this difficulty and permit the identification of actionable mutations in the majority of the samples collected.

UDT-Seq assay design

Gene selection We assembled a panel of 47 genes to be analyzed by UDT-Seq. The genes were selected for their clinical importance or their relevance to breast cancer genetics and treatment (Table 1). Thirteen genes were selected because of the availability of an FDA-approved drug (e.g. *ABL, EGFR, ERBB2*) targeting the genes or their pathway, with the rationale of repurposing approved drugs based on mutational profile. An additional ten genes were selected by the same rational for drugs that were in clinical trial at the time of the study (*PTEN*, *PIK3CA* and *KRAS*). Importantly, for some of the trials, the presence of a genetic alteration is an eligibility criterion. We included 12 additional genes known to be mutated in at least 1% of breast cancer [3]. Finally we added 11 genes for their importance in the germline only; 5 of them in the DNA repair pathway (e.g. *MLH1, MSH6, CHK2)*, 5 pharmacogenomic (PKG) genes (*CY2D6, DPYD, CYP2C9, TPMT, VKORC1*), and *CFTR*. Of note, 3 PKG genes and *CFTR* were not selected for their relevance to breast cancer, but rather to explore the feasibility of including additional types of genes in a clinical setting.

Primer design**:** The custom primer library was designed using the Primer3 v2.3.0 algorithm and the primers aligned back to the genome using isPCR v33, selecting only primer pairs resulting in one predicted amplicon. Our primer design pipeline included exhaustive primer selection across the targeted intervals with the following ideal parameters: 200 bp product length, 16-20 bp primer length, 40-60% GC, and avoiding sequences containing SNPs (dbSNP135). When no primer was designed under ideal parameters, the automated pipeline altered the parameters in the following order – product length, primer length and GC content – until the entire target region was covered (Table S2). After designing the locus-specific portion of the primers, sequence tails corresponding to a portion of the Illumina adaptor sequence were added to the sequence-specific portion of the primers prior to synthesis. The sequence added to the forward primers was CGCTCTTCCGATCT**CTG** and the sequence added to the reverse primer was CGCTCTTCCGATCT**GAC**. The tri-nucleotide sequence in bold was inserted between the specific target and the universal primer to confer adapter-strand specificity so that only the reads originating from the same end of the amplicon would be sequenced simultaneously. This ensured that the sequencing error rate could be

computed as a function of the read direction (forward or reverse). All primer pairs were used to prepare a primer droplet library (Raindance Technologies) using 5 pairs per droplet. While alternative approaches are frequently designed to examine only mutational hotspots [4], we chose to sequence the entire coding regions of the genes in the panel since hotspots continue to be identified and an increasing number of studies report functional mutations outside of hotspots. We designed 1,736 pairs of primers that targeted 99% (889/894) of the selected exons. Remarkably, 83% of the primer pairs were designed with the most stringent criteria controlling the presence of SNP, GC content and amplicon size, to ensure a more uniform amplification. The panel of amplicons had a total size of 228 kb, of which 154 kb were covering the targeted exons.

Germline Variation Summary

We identified a total of 586 inherited variants in the 38 patients, with a median of 140 per patient, of which, 498 were present in dbSNP (482 SNVs and 16 indels) and 88 were novel (61 SNVs and 27 indels). A total of 253 variants were unique to one patient (175 in dbSNP, 78 novel). 182 variants were non-silent (Table S8), and of those, 57 were predicted to be deleterious missense, 3 nonsense, 19 frameshift and 7 other mutations (splice sites, codon indels, start loss) by MutationTaster [5]. In total, 92% (79/86) of these predicted deleterious variants were seen in 3 or fewer patients and affected 28 genes. These figures are in agreement with the current understanding of human population genetics [6, 7].

References

1. Hammond MEH, Hayes DF, Dowsett M, Allred DC, Hagerty KL, Badve S, Fitzgibbons PL, Francis G, Goldstein NS, Hayes M, Hicks DG, Lester S, Love R, Mangu PB, McShane L, Miller K, Osborne CK, Paik S, Perlmutter J, Rhodes A, Sasano H, Schwartz JN, Sweep FCG, Taube S, Torlakovic EE, Valenstein P, Viale G, Visscher D, Wheeler T, Williams RB, et al.: **American Society of Clinical Oncology/College Of American Pathologists guideline recommendations for immunohistochemical testing of estrogen and progesterone receptors in breast cancer.** *Journal of clinical oncology : official journal of the American Society of Clinical Oncology* 2010, **28**:2784–95.

2. Wolff AC, Hammond MEH, Schwartz JN, Hagerty KL, Allred DC, Cote RJ, Dowsett M, Fitzgibbons PL, Hanna WM, Langer A, McShane LM, Paik S, Pegram MD, Perez EA, Press MF, Rhodes A, Sturgeon C, Taube SE, Tubbs R, Vance GH, van de Vijver M, Wheeler TM, Hayes DF: **American Society of Clinical Oncology/College of American Pathologists guideline recommendations for human epidermal growth factor receptor 2 testing in breast cancer.** *Journal of clinical oncology : official journal of the American Society of Clinical Oncology* 2007, **25**:118–45.

3. Forbes SA, Bindal N, Bamford S, Cole C, Kok CY, Beare D, Jia M, Shepherd R, Leung K, Menzies A, Teague JW, Campbell PJ, Stratton MR, Futreal PA: **COSMIC: mining complete cancer genomes in the Catalogue of Somatic Mutations in Cancer.** *Nucleic acids research* 2011, **39**(Database issue):D945–50.

4. Beadling C, Neff TL, Heinrich MC, Rhodes K, Thornton M, Leamon J, Andersen M, Corless CL: **Combining Highly Multiplexed PCR with Semiconductor-Based Sequencing for Rapid Cancer Genotyping**. *The Journal of Molecular Diagnostics* 2013, **15**:171–176.

5. Schwarz JM, Rödelsperger C, Schuelke M, Seelow D: **MutationTaster evaluates disease-causing potential of sequence alterations.** *Nature methods* 2010, **7**:575–6.

6. Durbin RM, Altshuler DL, Abecasis GR, Bentley DR, Chakravarti A, Clark AG, Collins FS, De La Vega FM, Donnelly P, Egholm M, Flicek P, Gabriel SB, Gibbs RA, Knoppers BM, Lander ES, Lehrach H, Mardis ER, McVean GA, Nickerson DA, Peltonen L, Schafer AJ, Sherry ST, Wang J, Wilson RK, Deiros D, Metzker M, Muzny D, Reid J, Wheeler D, Li J, et al.: **A map of human genome variation from population-scale sequencing**. *Nature* 2010, **467**:1061–1073.

7. MacArthur DG, Balasubramanian S, Frankish A, Huang N, Morris J, Walter K, Jostins L, Habegger L, Pickrell JK, Montgomery SB, Albers CA, Zhang ZD, Conrad DF, Lunter G, Zheng H, Ayub Q, DePristo MA, Banks E, Hu M, Handsaker RE, Rosenfeld JA, Fromer M, Jin M, Mu XJ, Khurana E, Ye K, Kay M, Saunders GI, Suner M-M, Hunt T, et al.: **A Systematic Survey of Loss-of-Function Variants in Human Protein-Coding Genes**. *Science* 2012, **335**:823–828.
